# Identification of Bioactive Metabolites of *Capirona macrophylla* by Metabolomic Analysis, Molecular Docking, and In Vitro Antiparasitic Assays

**DOI:** 10.3390/metabo15030157

**Published:** 2025-02-26

**Authors:** Joseph Evaristo, Elise de Laia, Bruna Tavares, Esdras Mendonça, Larissa Grisostenes, Caroline Rodrigues, Welington do Nascimento, Carolina Garcia, Sheila Guterres, Fábio Nogueira, Fernando Zanchi, Geisa Evaristo

**Affiliations:** 1 Center for the Study of Biomolecules Applied to Health (Cebio), Oswaldo Cruz Foundation Rondônia Unity (Fiocruz/RO), Porto Velho 76812-245, Rondônia, Brazil; joseph.am.evaristo@gmail.com (J.E.); elisebitencourte@gmail.com (E.d.L.); brunatavares13@outlook.com (B.T.); larissahbg@gmail.com (L.G.); carolrodrigues.bio@gmail.com (C.R.); 2 Bioinformatic and Medicinal Chemistry Laboratory, Fiocruz/RO, Porto Velho 76812-245, Rondônia, Brazil; esdrasmaia23@gmail.com (E.M.); fernando.zanchi@fiocruz.br (F.Z.); 3 National Institute of Epidemiology in the Western Amazon (INCT-EPIAMO), Porto Velho 76812-245, Rondônia, Brazil; 4 Malaria and Leishmaniasis Bioassays Platform Laboratory, Fiocruz/RO, Porto Velho 76812-245, Rondônia, Brazil; welington1717@gmail.com (W.d.N.); carolina.teles@fiocruz.br (C.G.); 5 Chemistry Department, Federal University of Rondônia (UNIR), Rio de Janeiro City 21941-598, Rio de Janeiro State, Brazil; sheila@unir.br; 6 Laboratory of Proteomics (LabProt), LADETEC, Institute of Chemistry, Federal University of Rio de Janeiro (UFRJ), Rio de Janeiro City 21941-598, Rio de Janeiro State, Brazil; fabiocsn@gmail.com; 7 Laboratory of Protein Chemistry-Proteomic Unit, Center for Research in Precision Medicine (CPMP), Carlos Chagas Filho Biophysics Institute, UFRJ, Rio de Janeiro City 21941-902, Rio de Janeiro State, Brazil

**Keywords:** mulateiro, tropical diseases, UHPLC/HRMS^n^, GNPS, in silico assay

## Abstract

*Capirona macrophylla* is a Rubiaceae known as “mulateiro”. Ethnobotanical extracts have been used for skin treatment and in the management of leishmaniasis and malaria. Objectives: The metabolites in aqueous extracts from wood bark, leaves, and stems were identified, and their in silico docking and in vitro cellular efficacy against *Leishmania amazonensis* and *Plasmodium falciparum* were evaluated. Methods: The extracts were analyzed by UHPLC/HRMS^n^ using untargeted metabolomics approach with MSDial, MSFinder, and GNPS software for metabolite identification and spectra clustering. The most abundant metabolites underwent molecular docking using AutoDock via PyRx, targeting the dihydroorotate dehydrogenase from *Leishmania* and *P. falciparum*, and evaluated through molecular dynamics simulations using Gromacs. In vitro biological assays were conducted on 60 HPLC-fractions against these parasites. Results: Metabolomics analysis identified 5100 metabolites in ESI+ and 2839 in ESI− spectra among the “mulateiro” samples. GNPS clustering highlighted large clusters of quercetin and chlorogenic acid groups. The most abundant metabolites were isofraxidin, scopoletin, 5(S)-5-carboxystrictosidine, loliolide, quercetin, quinic acid, caffeoylquinic acid (and isomers), chlorogenic acid, neochlorogenic acid, tryptophan, N-acetyltryptophan, epicatechin, procyanidin, and kaempferol-3-O-robinoside-7-O-rhamnoside. Molecular docking pointed to 3,4-dicaffeoylquinic acid and kaempferol as promising inhibitors. The in vitro assays yielded four active HPLC-fractions against *L. amazonensis* with IC50 values ranging from 175.2 μg/mL to 194.8 μg/mL, and fraction G29 showed an IC50 of 119.8 μg/mL against *P. falciparum*. Conclusions: The ethnobotanical use of “mulateiro” wood bark tea as an antimalarial and antileishmanial agent was confirmed through in vitro assays. We speculate that these activities are attributed to linoleic acids and quinic acids.

## 1. Introduction

*Capirona macrophylla* (Poeppig) Delprete (in the past classified as *Capirona decorticans* Spruce) is a tree species from the Rubiaceae family that can reach up to 40 m high and is native to floodplain areas in Amazonia Forest [1,2,3]. It is popularly known as “mulateiro”, “pau-mulato”, “mamalu”, “mamaluco, “perna-de-moça”, “capirona branca”, and “escorrega-macaco” in Brazil, and some local population also call it the “tree of youth” due to its potent antioxidant activity and use in dermatological formulas and cosmetic treatments to revitalize the skin.

*Calycophyllum spruceanum* (Benth.) K.Schum. is another Rubiaceae tree that is often mistaken with *Capirona macrophylla* because they share similar taxonomic characteristics, popular names, and traditional uses. Both trees are widely spread in the Amazonian biome, belong to the subfamily Ixoroideae, have a cylindrical trunk that breaks off into circular plates, have a dense crown, and their wood is widely used for furniture, fuel, ornamental, medicinal, and cosmetic purposes. The main differences are the size of the leaves and *Capirona macrophylla* has flowers with a pinkish corolla, while *Calycophyllum spruceanum* has greenish white flowers with eight petals.

These two species have (nine) patents related to hair and skin products based on “Mulateiro” extracts [4,5,6,7,8,9,10,11,12]. Ethnobotanical extracts of “Mulateiro” from *Capirona macrophylla* is used to treat skin diseases, acariosis, psoriasis, leishmaniasis and malaria [13,14], while “Mulateiro” from *Calycophyllum spruceanum* have been used as wound healing, photoprotector sunscreen, antidiabetic, antimicrobial, anti-inflammatory, and to treat parasitic diseases [15,16,17].

The literature has reported that ethanolic extracts of wood bark from *Capirona decorticans* have in vitro activity with IC50 23 µg/mL against *Leishmania donovani* LV9 amastigotes, IC50 > 10 µg/mL to *Plasmodium falciparum* 3D7, and IC50 > 100 µg/mL against amastigotes forms of *Leishmania amazonensis* [13,14]. A phytochemical composition of ethanolic extract from *Capirona decorticans* wood bark has described seven flavonoids: apigenin, rutin, luteolin, myricetin, quercetin, quercetin-3-β-D-glucoside, and quercitrin [18]. Additionally, the extract contains iridoids, indole alkaloids, terpenoids, anthraquinones and flavonoids as Rubiaceae chemotaxonomic metabolites [19].

In a molecular approach, two studies by Chibli et al. [20,21] identified several classes of molecules such as phenolic acid, alkaloids, flavonoids, and terpenes present in extracts from more than 50 Asteraceae species that inhibited the dihydroorotate dehydrogenase enzyme of *Leishmania major*.

These Amazonian metabolites, either from mulateiro or other plants, can be the key to the development of a pharmaceutical product against tropical diseases. The actual scenario of the current medical treatment against malaria and leishmaniasis, such as chloroquine, quinine, pentavalent and trivalent antimony and all its derivatives, is a decreased activity due to the resistance of many strains of the protozoa, summed their strong toxicity against human cells, side effects, and unknown mechanism of action [22,23,24,25,26]. Therefore, we have used the ethnopharmacological popular knowledge from the state of Rondônia in Brazil to research a promising plant against the protozoan parasites causing leishmaniasis and malaria, diseases that affected millions of people locally and worldwide every year. This research led to the discovery of *Capirona macrophylla* wood bark tea, a plant easily identified in the native forest by its height, straight and smooth bark, as well as its extensive ethnobotanical use.

This research has analyzed the metabolites present in aqueous extracts of *Capirona macrophylla* wood bark, as well as the ethnopharmacological uses by the local population. It also examined metabolites from leaves and branches to quantitatively compare metabolites’ biosynthesis and possible exploration of other parts of the plant without compromising tree survival. In silico interaction of the identified metabolites was performed by molecular docking using the available three-dimensional model target enzymes dihydroorotate dehydrogenase from *Plasmodium falciparum* and *Leishmania major*, which are essential for the survival of both parasites. Additionally, the in vitro parasiticidal molecular activity of 60 HPLC wood bark fractions was tested against *Leishmania amazonensis* and *Plasmodium falciparum*.

## 2. Materials and Methods

### 2.1. Plant Material

**Figure 1:**
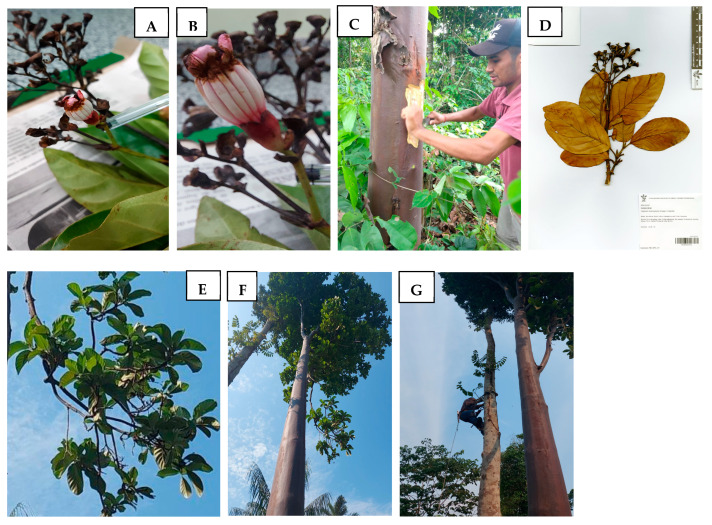
Photos of *Capirona macrophylla* inflorescence (**A**), flower (**B**), wood bark sampling (**C**), exsiccate voucher (**D**), leaves and branches (**E**), tree (**F**), and tree with the taxonomist climbing a tree next to the young mulateiro tree (**G**).

Leaf, stem, and wood bark material from *Capirona macrophylla* were collected in October 2019 at the coordinates 8°51″56.4′ S and 63°45″26′ W, in the city of Porto Velho/Rondônia/Brazil (Figure 1). A sample was identified and deposited at the “Herbário Rondoniense João Geraldo Kuhlmann (RON)” from Federal University of Rondônia (UNIR) under the code RON 22537. Access to genetic heritage was registered in the National System for the Management of Genetic Heritage and Associated Traditional Knowledge (SisGen) with the code A5E7ED4.

### 2.2. Extraction

The fresh leaf, stem, and wood bark tissues of *C. macrophylla* were cleaned with water, dried at 40 °C for 72 h, and pulverized.

Ten grams of each plant tissue were extracted through three rounds of decoction with 150 mL of water in the magnetic stirrer heated at 80 °C for 20 min, followed by an ultrasonic bath for 1 h, then subjected to vacuum filtration and lyophilization.

### 2.3. Analysis by UHPLC/HRMS^n^

Dried samples from leaves, stem, and wood bark were diluted in water:methanol (95:5) to reach a concentration of 10 mg/mL, then submitted to sonication for 20 min, centrifugation for 15 min at 14,000× *g*, and the supernatants were transferred to vials for analysis.

Five µL of each sample was injected into the ultra-performance liquid chromatography coupled with high-resolution tandem mass spectrometry (UHPLC/HRMS^n^, UHPLC Dionex Ultimate 3000/Q Exactive™ hybrid quadrupole-Orbitrap Plus, Thermo Fisher Scientific, Bremen, GE, USA), controlled by Xcalibur™ 3.2 software. Chromatography analysis was performed through a C18 column (2.1 × 50 mm (diameter × length), 1.8 μm, 100 Å, Zorbax Agilent, Santa Clara, CA, USA), a flow rate of 0.5 mL/min at 40 °C and mobile phase A composed of water with ammonium formate (5 mM) and formic acid 0.1%, and mobile phase B of methanol with formic acid 0.1%. The elution method followed the gradient: 10% B at 0 min, 10–100% B from 1 to 11 min, 100% B at 16 min, and re-equilibration with 10% B from 17 to 20 min. Heated Electrospray Ionization (HESI) source was settled with gas flow rates of 60 for sheath gas, 20 for auxiliar gas, and 10 for sweep gas, a spray voltage of 3.9 KV (positive) and 2.9 KV (negative), a current spray of 1.2 µA, and a capillary and auxiliar gas heater temperature of 325 °C. The S-lens RF level was set to 80. FullMS was acquired with a resolution of 70.000, an AGC target of 106, a maximum IT of 100 ms, a scan range of 67–1000 *m*/*z*, and profile mode. Fragmentation spectra were triggered in DDA (data-dependent acquisition) mode with a resolution of 17.500; an AGC target of 105; a maximum IT of 50 ms; Top 20; an isolation window of 2.0 *m*/*z*; isolation offset of 0.5 *m*/*z*; stepped NCE of 10, 15, and 30; dynamic exclusion of 6 s; and centroid mode. Positive and negative ionization acquisitions were acquired in individual run analysis and the samples were analyzed in technical triplicate.

HRMS calibrations were conducted prior to the acquisition analysis using a solution of caffeine, MRFA, and Ultramark 1621 for positive heated electrospray ionization (HESI) mode, covering the range of [M + H]+ from 195 to 1522 *m*/*z*. For negative calibration, a solution containing SDS, sodium taurocholate, and Ultramark 1621 was used, spanning [M-H]− from 265 to 1680 *m*/*z*. Both calibration solutions were purchased by Thermo Fisher Scientific.

### 2.4. HPLC Fractionation

Next, 0.5 g of wood bark dried sample was diluted in 5 mL of water:methanol (90:10), sonicated for 20 min, centrifuged for 15 min at 10,000× *g*, and the supernatant was filtered through a sterile 0.22 µm pore size. This sample was fractionated by HPLC with a diode array detector (Shimadzu Prominence UFLC, Kyoto, Japan) using a C18 column (10 × 250 mm (diameter × length), 5 μm, 300 Å, Jupiter Phenomenex, Torrance, CA, USA) and mobile phase A composed of water with acetic acid 0.1% and mobile phase B of methanol with acetic acid 0.1%. The gradient method followed the following steps: 10% B from the beginning until 2 min; 65% B at 47 min; 100% B at 49 min; maintaining 100% B until 54 min; and re-equilibrating with 10% B from 55 to 60 min. The flow rate was 5 mL/min at room temperature, and the fractions were collected manually every minute (F1 to F60). This procedure was repeated five times, and the equivalent of 394 mg of sample was fractionated. The corresponding fractions from these five fractionation procedures were summed and dried using a speed vacuum and lyophilization.

### 2.5. Metabolomic Analysis

UHPLC/HRMS^n^ raw data were converted to “.ibf” format and processed by the software MSDial (version 5.1.0, openly available) using the following parameters: MS1 and MS2 tolerances: 0.005 and 0.05 Da, respectively; minimum peak height: 50,000; mass slice width: 0.05; sigma window value for deconvolution: 0.5; retention time and MS1 tolerances for peak alignment: 0.2 min and 0.025 Da, respectively. All databases available from MSDIAL regarding positive and negative charged MS/MS spectra were downloaded (in December 2022) and used for metabolite identification: authentic standards and standards + bio + in silico libraries, MassBank, MassBank-EU, ReSpect, RIKEN IMS oxidized phospholipids, GNPS, Fiehn HILIC, CASMI2016, MetaboBASE, RIKEN PlaSMA authentic standards, RIKEN PlaSMA bio-MS/MS from plant tissues, Karolinska Institute and Gunma (GIAR) zic-HILIC deconvoluted MS2 spectra in data-independent acquisition, Fiehn Pathogen Box, Fiehn/Vaniya natural product library, BMDMS-NP, PFAS and MoNA.

For confirming the identification of metabolites previously reported in the literature, only the fragmentation spectra (MS^2^) and/or full molecular mass with errors below +/−5 ppm, as determined by MSDIAL (Supplementary Material S3), were considered valid.

Data processed via MSDIAL 5.1.0 software were also converted into MSP and MGF files in order to export the list of masses of interest from MSDIAL to MSFinder 3.52 (http://prime.psc.riken.jp/compms/msfinder/main.html, accessed on 1 December 2022) and Global Natural Products Social Molecular Networking (GNPS, [27] V.1.2.16, (https://gnps.ucsd.edu/ProteoSAFe/static/gnps-splash.jsp, accessed on 1 December 2022). The GNPS method of analysis was Feature-Based Molecular Networking (FBMN), with the parameters of ion precursor tolerance and ion fragmented set to 0.02 Da, a coseno minimum value of 0.68, and a combination of at least 6 ion fragments to create a molecular network. The results were exported as a graphml extension into Cytoscape 3.9.1 software [28].

### 2.6. Virtual Screening by Molecular Docking

Motivated by the studies conducted by Chibli et al. [20,21], which demonstrated the inhibition of dihydroorotate dehydrogenase in *Leishmania major* by various molecules found in natural products, a virtual screening protocol was established using molecular docking. The goal was to investigate whether the molecules identified in this study had the potential to inhibit the same enzymes in *Leishmania amazonensis* and *Plasmodium falciparum*. Therefore, a virtual screening by docking was used to determine which compounds might be candidates for inhibitors for the dihydroorotate dehydrogenase of *Leishmania major* (*Lm*DHODH) and dihydroorotate dehydrogenase of *Plasmodium falciparum* (*Pf*DHODH). The 3D structures of metabolites identified from UHPLC/HRMS^n^ were downloaded from PubChem [29]; subsequently, these structures were minimized using the software Open Babel [30], and then converted to .pdbqt format in the interface PyRx 0.9.8 [31]. The energy calculation of the molecular docking was performed by the Autodock4 4.2.6 software using interface PyRx 0.9.8. The simulation grid configuration varies according to the targets, so the following configuration was utilized: (a) for *Lm*DHODH, PDB Id: 3GZ3, the center of the grid was positioned at the geometric center of the crystallized substrate orotate (ORO) at the following coordinates: X: 52.502, Y: −21.782, and Z: −15.089. The number of grid points was as follows: 47 × 47 × 47, 0.375 Å of spacing; (b) for *Pf*DHODH, PDB Id: 5BOO [32], the center of the grid was positioned at the geometric center of the crystallized ligand DSM265 at the following coordinates: X: −28.841, Y: −8.252, and Z: −12.991, with the number of points as follows: 47 × 47 × 47, and 0.375 Å of spacing. The Lamarckian genetic algorithm and standard semi-flexible docking were used with a protocol consisting of 50 independent runs per ligand and the maximum number of energy evaluations was set to medium, all the other settings were left at their defaults. Interaction analyses were performed using PoseView from ProteinsPlus [33].

### 2.7. Molecular Dynamics

The structural behavior of the protein and ligands were analyzed by Molecular Dynamics (MD) simulations for *Pf*DODH and *Lm*DHODH. In total, six simulations were conducted: three for *Pf*DODH and three for *Lm*DHODH. For each enzyme, these three simulations were based on [a] the best ligands docked in the presence of flavin mononucleotide (FMN), [b] the systems with their respective ligands crystallized in the presence of FMN, and finally, [c] the systems with the enzymes in their free (APO) form.

MD were performed using Gromacs 2024.1 [34] with interface Visual Dynamics [35] for generating scripts such as Amber99 force field [36]. The partial charges and ligand topologies were obtained by Acpype [37] using the ANTECHAMBER [38] module. Electrostatic interactions were treated using the particle mesh Ewald (PME) algorithm with a cut-off of 12 Å. Each system was simulated under periodic boundary conditions in a cubic box, whose dimensions were automatically defined, considering 2 Å from the outermost protein atoms in all Cartesian directions. The simulation box was filled with TIP3P water molecules [39]. Subsequently, a two-step energy minimization procedure was performed: 2000 steps of steepest descent and 2000 steps of conjugate-gradient or until the system reaches a resistance force lower than 1000 kJ·mol^−1^·nm^−1^. Next, initial atomic velocities were assigned using the Maxwell-Boltzmann distribution corresponding to a temperature of 300 K. All systems were subsequently equilibrated during two successive NVT and NPT equilibration simulations with 200 ps for each. After this period, all the systems were simulated with no restraints at 300 K in the Gibbs ensemble with a 1 atm pressure using isotropic coupling. All chemical bonds containing hydrogen atoms were restricted using the SHAKE algorithm [40] and the time step was set to 2 fs. Finally, we simulated independent MD runs of 150 ns for each system. Simulation trajectories were analyzed with GROMACS package tools [34]. Root-mean-square deviation (RMSD) was calculated separately for each system fitting their heavy atoms, taking the initial structure of the production dynamics as a reference. Hydrogen bonds (H-bond) were calculated between protein + cofactor (FMN) and ligand complexes. We considered one to be a hit when the distance between two polar heavy atoms, with at least one hydrogen atom attached, was less than 3.5 Å and the H-donor angle was higher than 120°.

### 2.8. In Vitro Biological Activity

#### 2.8.1. Antiplasmodial Activity

Strain *Plasmodium falciparum* clone W2, resistant to chloroquine, was cultured in human red blood cells (type O, Rh+; hematocrit in 2%) as described by Trager and Jensen [41]. Asexual blood forms of *Plasmodium* were maintained in 10.4 g/L RPMI 1640 medium (Sigma-Aldrich), supplemented with 25 mM HEPES (Sigma-Aldrich, St. Louis, MI, USA), 0.3 mM hypoxanthine (Sigma-Aldrich), 11 mM glucose (Thermo-Fisher Scientific, Waltham, MA, USA), 40 mg/L gentamicin (Novafarma, Anápolis, Brazil), and 10% (*v*/*v*) O+ plasma or 2.5 mg/L Albumax (Thermo-Fisher Scientific, Waltham, MA, USA). The culture bottles were stored at 37 °C in a gaseous mix made up of 5% CO_2_, 5% O_2_, and 90% N2. Parasitemia was monitored daily by optical microscopy with an immersion objective (1000×) through the thin smear method on slides stained with the panoptic kit (Newprov, Irvine, CA, USA).

After the culture reached parasitemia above 8% in young trophozoites, it was synchronized [42] and an antiplasmodial assay was started. Compounds were solubilized in DMSO and adjusted to a concentration of 2000 µg/mL (20 μL) in triplicate in 96-well U-bottom microplates. A volume of 180 µL of the synchronized culture with a 2% hematocrit value and 0.5% parasitemia was added to the plate. Therefore, each of the 60 HPLC wood bark fractions from *C. macrophylla* were diluted 10-fold. Starting from a concentration of 200 µg/mL, the following serial dilutions were evaluated: 100 µg/mL, 50 µg/mL, and 25 µg/mL. Artemisinin (Art) at a final concentration of 0.2 μg/mL was used as a death control. For growth control, red blood cells infected with parasites with no treatment were employed, while non-infected red blood cells, whose fluorescence was subtracted from the values obtained, were used as blanks. The experimental culture was incubated under the same conditions described above in the bottles for 72 h. After the end of this period, the supernatant was removed without suspending the red blood cell precipitate. RBCs were washed with 1× PBS and centrifuged at 700× *g* for 10 min. Then, the supernatant was discarded, and the antimalarial evaluation was performed using SYBR Green I (Thermo Fisher). Next, 100 µL of lysis buffer with SYBR Green were added, and the contents were transferred to microplates containing 100 μL of 1× PBS. The plates were incubated for 30 min at 24 °C; fluorescence was determined by a multi-detection system (Synergy HT BioTek, Hampton, United States), which was obtained at an excitation of 485 nm and an emission of 590 nm [43,44]. The results were expressed as a percentage of growth inhibition (IC).

#### 2.8.2. Antileishmanial Activity

The *Leishmania* strain used in this study was provided by the CLIOC (IOC-Instituto Oswaldo Cruz, Rio de Janeiro). *Leishmania (Leishmania) amazonensis* (IFLA/BR/67/PH8) promastigotes were routinely cultured in vitro at 1 × 10^6^ promastigotes/mL in RPMI 1640, supplemented with 10% inactivated fetal bovine serum (FBS), 2 mM L-glutamine, 20 nM HEPES (N-2-hydroxyethylpiperazine-N’-2-ethanesulfonic acid), 21 mM sodium bicarbonate, 11 mM glucose, and 50 mg/mL of gentamycin. The parasites were kept in a BOD incubator at 24 °C and subcultured every 120 h.

For antileishmanial activity, promastigotes were centrifuged at room temperature at 1500 rpm (478× *g*) for 15 min. The supernatant was discarded, and the pellet was resuspended in complete RPMI-1640 medium. So, *L. (L.) amazonensis* concentration was adjusted to 1×106 parasites/mL with the aid of a Neubauer chamber by optical microscopy at 400× magnification, and the parasites were plated in 96-well microplates and incubated at 24 °C for 72 h with 200 μg/mL, 100 µg/mL, 50 µg/mL, and 25 µg/mL in DMSO (0.6%) of each 60 HPLC wood bark fractions from *C. macrophylla*. Subsequently, 10 μL of a 2 mM solution of resazurin (dissolved in PBS) were added to each well, and the plates were incubated for another 5 h. Then the plates were read with a fluorescence plate reader (Synergy HT BioTek, Hampton, VA, USA) using an excitation wavelength of 530 nm and an emission wavelength of 590 nm. Positive controls were parasites with no treatment; and the death control was amphotericin B (3 µg/mL). Each concentration was screened in triplicate (adapted from [45]).

Data was transferred into the graphic software Prism^®^8.4.3, and the results were expressed as the percentage of growth inhibition, which was determined by the following formula:

IC (%) = 100 − (test fluorescence − fluorescence of Medium RPMI) × 100 (fluorescence of control with no treatment − fluorescence of Medium RPMI)

#### 2.8.3. Hemolysis Assay

The possible hemolytic activity of the 60 HPLC wood bark fractions from *C. macrophylla* were determined according to Wang et al. [46]. Briefly, human blood was collected from a healthy O+ donor, and 180 μL of these erythrocytes at 1% hematocrit were added in U-bottomed microplates. After that, 20 µL of the HPLC fractions were added individually to the red blood cell suspension (final concentrations of 100 µg/mL, 50 µg/mL, 25 µg/mL and 12.5 µg/mL). The plates were incubated for 30 min at 37 °C with agitation after every 5 min, followed by centrifugation for 10 min at 478× *g*. The supernatant was analyzed using a microplate spectrophotometer ( model: Expert Plus, Biochrom^®^, Midland, ON, Canada) at 540 nm. Saponin (0.05%) was used as a positive control for hemolysis, whereas non-treated red blood cells were used as a negative control. The blank (RPMI) was used to subtract the absorbance of that medium in relation to all samples by the end of the test. The results obtained were expressed as optical density. To assess significance, the ANOVA test was performed (*p* < 0.05), followed by Tukey’s post-test. The absorbance values of the test compounds were compared in relation to the control of untreated erythrocytes.

## 3. Results and Discussion

### 3.1. Untargeted Metabolomic Analysis

**Figure 2:**
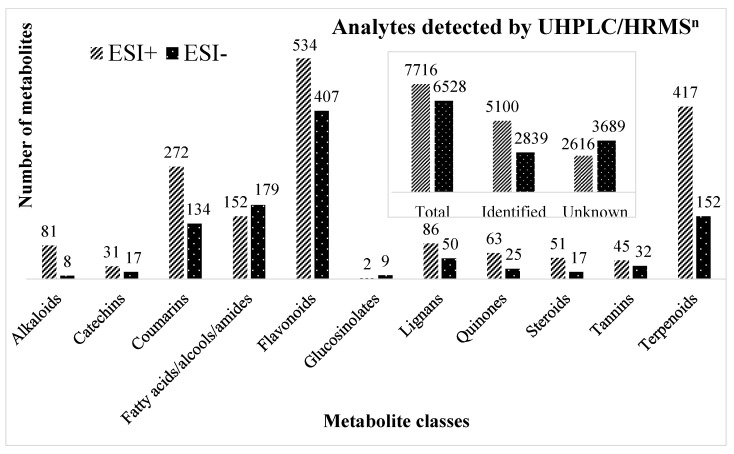
UHPLC/HRMS^n^ analysis detected features and identified metabolites classes by MSDIAL software from *Capirona macrophylla* leaf, stem, and wood bark samples, acquired separately in electrospray positive (ESI+) and negative (ESI−) ionization modes.

An untargeted approach was used to analyze the metabolome and 7.716 features acquired on ESI+ and 6.528 features on ESI− were obtained (Figure 2). From them, 5.100 metabolites on ESI+ and 2.839 on ESI− were identified by FullMS and/or MS2 spectra with high resolution. An important characteristic is that many metabolites can be ionized and detected on both polarities. Flavonoids and terpenoids were the most abundant secondary metabolite classes among the identified metabolites.

**Figure 3:**
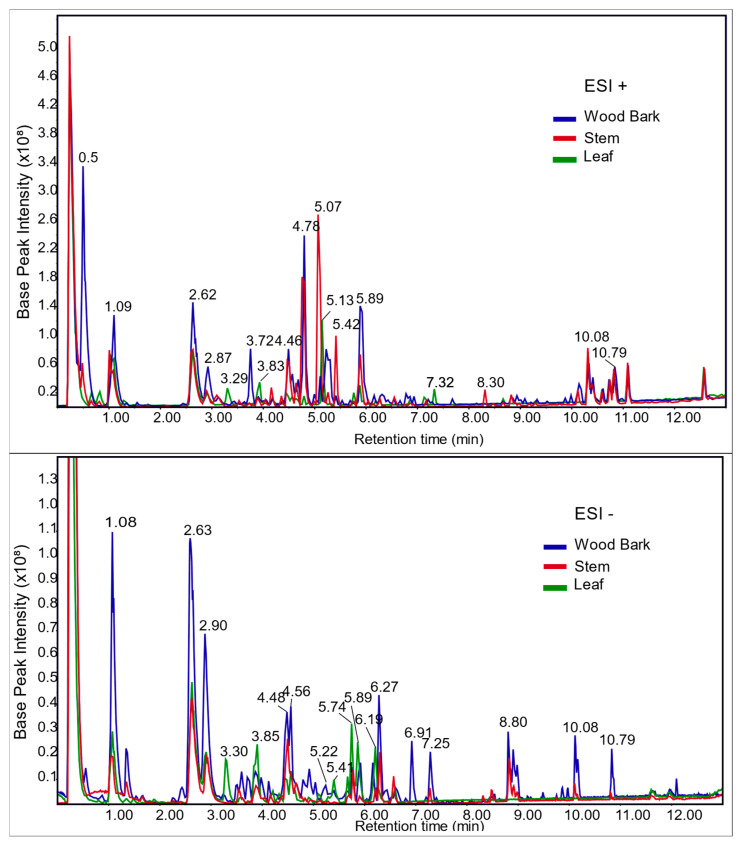
Chromatograms based on HRMS^n^ analysis of water extracts from leaf, stem, and wood bark of *Capirona macrophylla* acquired independently in electrospray positive (ESI+) and negative (ESI−) ionization mode analysis.

The chromatograms based on high-resolution mass spectrometry (HRMS^n^) analysis of water extracts from *C. macrophylla* leaf, stem, and wood bark, acquired on positive (ESI+) and negative (ESI−) ionization modes, showed that the stem and wood bark samples had more similarities in qualitative composition among the three group samples (Figure 3). The wood bark crude sample had a richer number of peaks and higher relative concentration UHPLC/HRMS profiles than the other samples. Some unique metabolites found in the wood bark sample were: xanthoquinodin B2, a xanthene with reported activities against microbial, fungi, various types of cancer and protozoa, including *Plasmodium falciparum* [47,48,49]; tubocurarine (alkaloid), a neuromuscular blocker, activator of neurohormonal pathways, analgesic and anti-inflammatory agent [50,51]; gossypol, a sesquiterpene with antineoplastic activity for breast and prostate cancers, as well as a contraceptive effect for men [52,53,54]; and genistein 8-C-glucoside, an isoflavone common in plants with antioxidant, phytoestrogenic and anticarcinogenic activities through the induction of mitochondrial membrane depolarization [55,56]. Vincristine, a widely used chemotherapy drug [57], was also detected in the wood bark and stem in the same relative concentration but was not present in the leaf samples. Several other metabolites with several anticancer activities in the literature were identified in this research within the three types of samples, such as myricetin and derivatives glycosylated [58].

Table 1 and Table 2 listed and described the most intense peaks from the ESI+ and ESI− chromatograms (Figure 3), respectively. Precursor and fragmentation ions from mass spectra were reported for these analytes, as well as for the highly abundant metabolites that could not be identified by database matches. Supplementary Materials S1 (ESI+) and S2 (ESI−) provided the FullMS and MS^2^ mass spectra of all metabolites listed in these tables. The most abundant metabolites identified in the leaf sample were the flavonoids quercetin, epicatechin, procyanidin, and kaempferol-3-O-robinoside-7-O-rhamnoside, as well as the monoterpene loliolide. All these compounds were also found in the stem and wood bark samples, except for procyanidin, which was absent in the wood bark, and present in low concentration in the stem sample. The coumarins fraxidin, isofraxidin, and scopoletin, which have known anticancer, neuroprotective, cardioprotective, anti-inflammatory, and antioxidant effects [59,60,61,62], were found in higher concentrations in the stem sample. The amino acids tryptophan, malonyltryptophan, and N-acetyltryptophan, along with quinic acids, caffeoylquinic acids (and their isomers), chlorogenic acid, neochlorogenic acid, 5(S)-5-carboxystrictosidine, and fatty acids such as linolenic acid, decanedioic acid, FA 18:2 + 20, and FA18:1 + 30, were present in higher concentrations in the wood bark sample.

The seven flavonoids from *Capirona decorticans* described in the literature are as follows: apigenin, rutin, luteolin, myricetin, quercetin, quercetin-3-β-D-glucoside, and quercitrin [18]. These flavonoids were found in the aqueous extract of leaf, steam, and wood bark samples by ESI+ and/or ESI− analysis (Supplementary Material S3). Moreover, glycosylated versions of these flavonoids were also identified, such as the following: apigenin 6,8-digalactoside, apigenin-7-O-(2G-rhamnosyl)gentiobioside, apigenin-7-O-glucoside, apigenin-6-C-glucoside-7-O-glucoside, luteolin 7-glucoside, luteolin C-glucoside C-xyloside, quercetin-3-ramnoside, quercetin-3-O-vicianoside, 3-Glu-7-Rha quercetin, 3-O-beta-(6″-trans-caffeoyl)-galactopyranosyl quercetin, 3-O-beta-(6″-trans-caffeoyl)-galactopyranosyl quercetin, 6-methoxyluteolin, dihydroquercetin, quercetin 3,7-dimethyl ether, and peracetate quercetin.

Interestingly, there is another Rubiaceae tree known as Mulateiro, belonging to the species *Callycophyllum spruceanum*, that is widely traded in local herbal markets in the Amazonian region. It is often confused with the species *Capirona macrophylla*. Three studies have described the metabolites from the wood bark of *Calycoplyllum spruceanum*: Zuleta et al. [63] identified secoxyloganin, kingiside, gardenoside, loganin, loganetin, diderroside, 7-methoxydiderroside, 6-O-acetyldiderroside, and 8-O-tigloyldiderroside; Peixoto et al. [16] reported gardenoside, 5-hydroxymorin, cyanidin, taxifolin, and 5-hydroxy-6-methoxycoumarin-7-glucoside; and Da Silva et al. [64] described quinic acid, 3-O-caffeoylquinic acid, 4-O-caffeoylquinic acid, 3,4-O-dicaffeoylquinic acid, 5-hydroxymorin, and taxifolin. Curiously, all these metabolites identified from the wood bark of *Calycoplyllum spruceanum,* along with the seven flavonoids reported in *Capirona decorticans* [18], were also found in the aqueous samples of *Capirona macrophylla*. This shared chemical composition among these plant species, collectively known as “Mulateiro”, may serve as a chemotaxonomic marker to define the “Mulateiro” trees and their ethnopharmacology effects.

The identification of secoxyloganin, kingiside, and gardenoside, that share the molecular formula C17H24O11, but have slightly different structures, was peculiar because the MS-DIAL/MS-FINDER recognized the MS^2^ spectra of five different retention times (RT) of the ions [427.1213 + Na]+ and [403.1274 − H]−, as well as the other three different RT with *m*/*z* [405.1394 + H]+ without MS^2^, as secoxyloganin with a high score and gardenoside as a second identification choice. Moreover, because the fragmentation spectra showed different ions, we have concluded that some of these compounds might be kingiside and gardenoside. In fact, the occurrence of repeated *m*/*z* values and identifications on different RT and fragmentation ion profiles on MS^2^ suggests the presence of isomers with different structural formulas, such as loganin, that were identified by MS-DIAL/MS-FINDER on twelve different RT, and the fragmentation mass spectra have different ions among them. Additionally, when the MS2 profile shows the same ions and equivalent relative abundance, it is plausible that occurrences are enantiomers in the sample, as we can observe for cyanidin-3-O-glucoside with 5.88 and 6.23 min (Supplementary Material S4).

Cyanidin as the theoretical molecular weight was not found, even after searching for raw data for each sample (Supplementary Material S3). However, other cyanidin-family metabolites were identified, such as cyanidin-3-O-glucoside, cyanidin-3-O-galactoside, cyanidine-3-O-sambubioside, cyanidin-3-O-sophoroside, cyanidin-3-O-(2″-O-beta-xylopyranosyl-beta-glucopyranoside), cyanidin-3-O-rutinoside, cyanidin-3,5-di-O-glucoside, procyanidin, procyanidin A1, procyanidin A2, procyanidin B1, procyanidin B2, procyanidin C1, and procyanidin trimer, all of them either exclusively or in more abundance on the leaf sample. Cyanidin is a pigment characteristic of red berries, leaves, and flowers, and it plays a function as a neuroprotective agent, antioxidant, and protectant against breast, liver, lung, prostate, and thyroid cancers [65].

**Figure 4:**
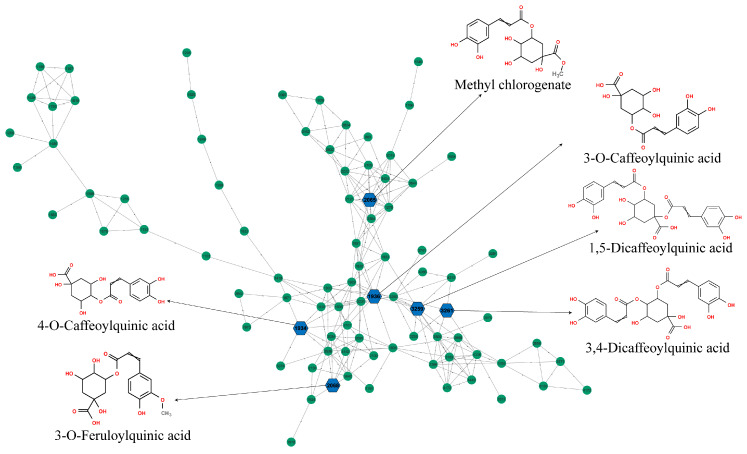
A cluster of caffeoylquinic acids and isomers via Cytoscape view from Feature-Based Molecular Networking analysis generated by GNPS software from MS/MS spectra acquired on negative ionization mode from the *Capirona macrophylla* samples: leaves, stem, and wood bark. The full analysis is available in the following link: https://gnps.ucsd.edu/ProteoSAFe/status.jsp?task=527162782caa4f79a2f3ee3f7e12fe59, accessed on 15 February 2023.

By the Feature-Based Molecular Networking provided by GNPS online software, it was possible to classify other compounds and the phytochemical profile similarity between them, even the ones that were not identified, in both ESI+ and ESI− (https://gnps.ucsd.edu/ProteoSAFe/status.jsp?task=1c821747b908401d82e1184b8b9292f8, accessed on 06 March 2023, and https://gnps.ucsd.edu/ProteoSAFe/status.jsp?task=527162782caa4f79a2f3ee3f7e12fe59, accessed on 15 February 2023). The major cluster of the ESI− molecular network consisted of 87 compounds with spectra similar to those of caffeoylquinic acids (Figure 4). Identifications were performed by comparison of the fragment ions and their intensities on MS^2^ spectra with the literature reference of [66,67]. Therefore, 3-O-caffeoylquinic acid (3-O-CQA), 4-O-caffeoylquinic acid (4-O-CQA), 5-O-caffeoylquinic acid (5-O-CQA), 1,5-dicaffeoylquinic acid, 3,4-dicaffeoylquinic acid, and 4,5-dicaffeoylquinic acid could be identified and were present in high concentrations in the samples (as can be observed in the ESI− chromatogram of Figure 3, and Table 2). They also showed relevant results in the molecular docking analysis (Table 3).

### 3.2. Virtual Screening by Molecular Docking

Dihydroorotate dehydrogenase is a crucial enzyme involved in the biosynthesis of pyrimidines that is a validated target for anti-infective drug research [20]. In addition, some studies have recently emerged showing the potential in the use of natural products against *Lm*DHODH [68] and *Pf*DHODH [69]. The structures of *Lm*DHODH and *La*DHODH have 92% global similarity between them (Clustal 2.1 analysis) and a highly similar space in the active site. The molecular docking with *Lm*DHODH was based on the original substrate orotate (ORO) (RMSD of redocking = 0.11 Angstroms), and with *Pf*DHODH with the ligand inhibitor DSM265 (RMSD of redocking = 0.67 Angstroms). Both substrate and ligand are present in the PDB files of their respective enzymes, and they were used as a positive control.

The energies of the in silico molecular interactions of metabolites from *C. macrophylla* with *Lm*DHODH and *Pf*DHODH are listed in Table 3. The test with *Lm*DHODH revealed that the 3,4-dicaffeoylquinic acid (CID: 5281780), 3-feruloylquinic acid (CID: 9799386), and 1,4-dicaffeoylquinic acid (CID: 12358846) showed highly competitive energies of −8.78, −8.35, and −8.08 kcal/mol when compared to the literature substrate ORO of −8.56 kcal/mol. The interactions with *Pf*DHODH showed that kaempferol (CID: 5280863), quercetin (CID: 5280343), and anabasamine (CID: 161313) are promising metabolites with competitive energy of −8.82, −8.62, and −8.57 kcal/mol, compared to −9.82 kcal/mol of the ligand inhibitor DSM265. Due to the error variation of ±2.00 kcal/mol, the metabolites from Table 1 that showed docking energy until −7.82 kcal/mol are also considered competitive inhibitors to *Pf*DHODH.

**Figure 5:**
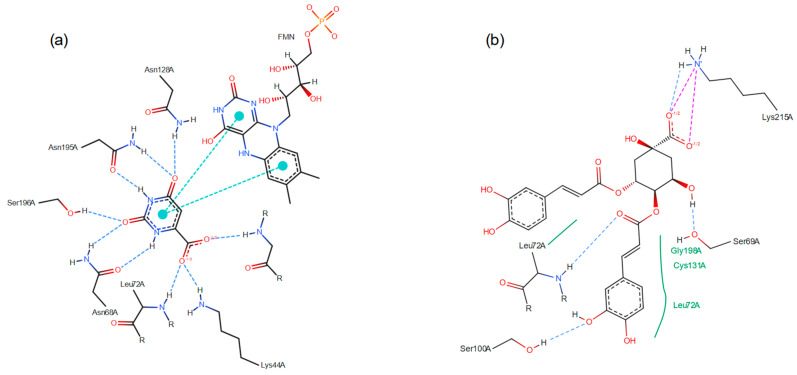
Molecular docking showing the chemical bonds of the enzyme dihydroorotate dehydrogenase (DHODH) of *Leishmania major* and the metabolite (**a**) Orotate; (**b**) 3,4-O-DiCQA. The types of interactions are separated by colors: in blue are conventional hydrogen bonds; in green are van der Waals; in cyan are π-π stacking; in red is the attractive charge.

The chemical bonds between *Lm*DHODH and ORO (Figure 5a) presented nine hydrogen bonds, two π-π stacking interactions. In contrast, the 3,4-dicaffeoylquinic acid (Figure 5b) presented four hydrogen bonds, two attractive charges, and three Van Der Waals interactions. Therefore, 3,4-dicaffeoylquinic acid had a more intense interaction with the *Lm*DHODH when compared with the original ligand substrate, ORO.

### 3.3. Molecular Dynamics Simulations

After 150 ns of simulation, the best docked candidates for *Lm*DHODH and *Pf*DHODH—3,4-Dicaffeoylquinic acid (CID: 5281780) and Kaempferol (CID: 5280863), respectively—demonstrated stabilization of DHODH based on the RMSD analysis of the enzymes (Supplementary Figures S6 and S7). In the case of Kaempferol for *Pf*DHODH, a significant improvement was observed compared to DSM265, which is a widely tested in vitro inhibitor against *Pf*DHODH (Supplementary Figure S6). Notably, despite producing fewer hydrogen interactions than DSM265, KaemPferol outperforms it significantly in terms of van der Waals interactions (Supplementary Figure S6). As for *Lm*DHODH, it is well-established that the substrate Orotate (ORO) confers substantial stability to the enzyme, primarily through hydrogen bonding. However, the van der Waals interactions of the candidate 3,4-Dicaffeoylquinic acid indicate that this ligand remains sufficiently bound to the enzyme to compete with ORO (Supplementary Figure S7).

### 3.4. Biological Activity

**Figure 6:**
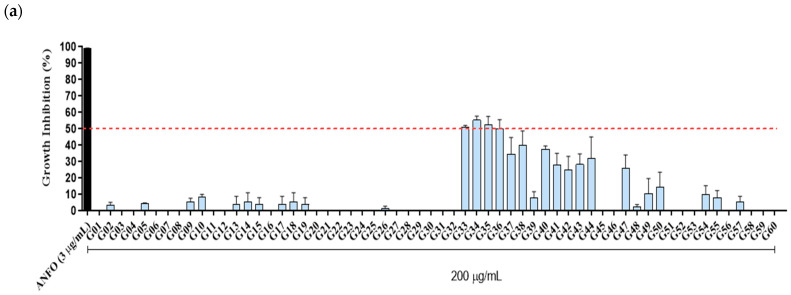

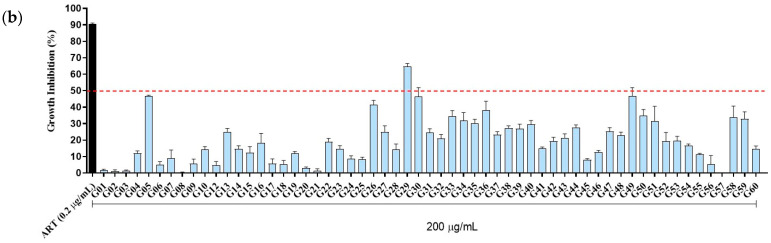
Biological Activity of 60 HPLC wood bark fractions from *Capirona macrophylla* (G01–G60). (**a**) Leishmanicidal effects of metabolites (fractions) on promastigotes forms of *L. (L.) amazonensis* treated for 72 h. (**b**) Antimalarial effects of metabolites (fractions) in inhibiting the growth of *Plasmodium falciparum* W2 treated for 72 h. Results are expressed as the mean percentage in treated parasites compared to the control (no treatment) ± SD of two independent experiments.

Among the 60 HPLC fractions tested (G01–G60), fractions G33, G34, G35, and G36 inhibited the growth of promastigotes *L. (L.) amazonensis* by 51%, 55.3%, 52.5%, and 50.3%, respectively, at a concentration of 200 μg/mL (Figure 6a). The IC50 values for these fractions, calculated over a range of 200 to 25 μg/mL, were 180.2 μg/mL, 175.2 μg/mL, 194.8 μg/mL, and 183.8 μg/mL, respectively. Four other fractions, G37, G38, G40, and G44, showed lower inhibition (30–40%) at the same concentrations for these protozoans. Fractions G41, G42, G43, and G47 inhibited parasite growth by about 20% at 200 μg/mL, while the remaining fractions showed minimal or no inhibition. The death control used in the anti-Leishmania bioassays, Amphotericin B, was able to inhibit 100% at 3 μg/mL.

In antimalarial tests, although a smaller number of HPLC fractions at a concentration of 200 μg/mL was observed inhibiting the growth of *P. falciparum* W2 by up to 50% (only G29 = 65% inhibition), there was a greater number of HPLC fractions that showed inhibition between 20 and 40%. Twelve of the 60 HPLC fractions mentioned above showed in vitro activity, inhibiting about 30–46% of the cultured strains: G05, G26, G30, G33, G34, G35, G36, G49, G50, G51, G58, and G59. Eleven of them showed little antimalarial activity, with a percentage value of 20%: G13, G31, G32, G37, G38, G39, G40, G43, G44, G47, and G48 (Figure 6b). In these results, it is emphasized that the metabolite G29 was active only for *P. falciparum*, with a calculated IC50 value of 119.8 μg/mL. The artemisinin control at 0.2 μg/mL inhibited the growth of 100% of the asexual forms of *Plasmodium* in culture.

By comparing the HPLC (Supplementary Figure S5) and UHPLC chromatograms (Figure 3), we identified correspondences among the peak elutions. Specifically, the peaks corresponding to the most active fractions—G05, G29–30, and G33–38—align with retention time of 1.09, 10.08, and 10.79 min from UHPLC chromatogram, respectively. Despite the co-elution of various metabolites, our observations indicate the most abundant metabolites during these retention times are quinic acids, linoleic acids, and medium to long-chain fatty acids.

None of the compounds showed hemolytic rates for human erythrocytes at the highest concentration tested (200 μg/mL); that is, it was observed that the absorbance values obtained from the erythrocytes treated with the metabolites were not statistically different (*p* > 0.05) from the negative control values (erythrocytes in incomplete RPMI-1640 medium).

## 4. Conclusions

The untargeted metabolomics by UPLC/HRMS^n^ of *Capirona macrophylla* extracts have disclosed 9.595 metabolite features; however, only 393 were properly identified by MS^2^ spectra, and those were tested for in silico screening against *Leishmania amazonensis* and *Plasmodium falciparum*. Molecular docking was the strategy to search for potential inhibitors and indicate the biological assays prior to the in vitro assay, in which 27 metabolites tested against *La*DHODH showed that all caffeoylquinic acids were promising inhibitor competitors. In vitro antiparasitic assays also revealed that quinic acids, in addition to linoleic acids, may be the active metabolites against the *P. falciparum* W2. Furthermore, the co-elution of various medium to long-chain fatty acids in active fractions suggests their potential role in the antiparasitic activity against *L. amazonensis*. GNPS has clustered similar mass spectra and therefore can be used as another strategy to interpret the untargeted metabolomic data without the need to identify all the metabolites in the sample. In addition, they could predict chemotaxonomy markers that can indicate bioactivity, such as flavonoids, terpenes, coumarins, alkaloids, and cholines in *C. macrophylla*, granting antioxidant, anti-inflammatory, bronchodilator, vasodilator, diuretic, healing, antidopaminergics, anticholinesterases, and antitumor activities. The detailed characterization of these bioactive compounds present in *Capirona macrophylla* could lead to the development of new pharmaceutical and herbal products. Exploring the medicinal properties of these compounds may result in innovative treatments for tropical diseases, leveraging the traditional knowledge of Amazonian communities.

The strategy of this research to find bioactive metabolites, that can be a pipeline, was to analyze the crude extract of different parts of a plant by an untargeted metabolomic approach, the identified metabolites were subjected to in silico screening against specific (parasite) enzymes and followed by the related in vitro test.

## Figures and Tables

**Table 1:** Characterization of the major chromatogram peaks from leaf, stem, and wood bark water extracts of *Capirona macrophylla* by UHPLC/HRMS^n^ on ESI positive acquisition mode. The compounds were identified by MS-DIAL and GNPS software.

ID	t_R_ (min) Average	Formula [M + H]^+^	Experimental Mass (*m*/*z)*	Error (ppm)	Peak Area Leaf/Stem/Wood Bark	Name	CID PubChem	MS^2^ Ions and Percentages (*m*/*z,* %)
1	0.5	-	254.1606	-	9.2 × 10^6^/1.5 × 10^8^/8.8 × 10^8^	Unknown	-	254.1609 (100%), 237.1342 (3.5%), 196.1163 (3.3%), and 195.1127 (32.0%)
2	1.09	C_11_H_13_N_2_O_2_	205.0971	−2.75	1.7 × 10^8^/1.7 × 10^8^/3.8 × 10^8^	Tryptophan	6305	188.0705 (100%), 146.06 (32.7%), 189.0740 (13.0%), 144.0807 (5.4%) and 159.0916 (5.3%)
3	2.62	C_16_H_19_O_9_	355.1026	−0.96	1.6 × 10^8^/2 × 10^8^/4.6 × 10^8^	Chlorogenic acid	1794427	163.0389 (100%), and 355.1026 (44.9%)
4	2.87	-	289.1182	-	2.3 × 10^6^/1.1 × 10^7^/3 × 10^7^	Unknown	-	216.1017 (100%), 272.0914 (53.7%), 289.1182 (44.9%), 188.0706 (20.5%) and 180.0803 (7.7%)
5	3.29	C_30_H_27_O_12_	579.1484	−3.24	3 × 10^7^/9.8 × 10^3^/0	Procyanidin	130556	127.0389 (100%), 579.1385 (88.9%), 291.0858 (78.8%), 427.1019 (78.7%), and 409.0906 (55.3%)
6	3.72	-	496.2035	-	8.2 × 10^5^/8.7 × 10^6^/8.6 × 10^7^	Unknown	-	185.0808 (100%), 308.8935 (44.1%), 371.3235 (42.4%), and 330.3095 (37.6%)
7	3.83	C_15_H_15_O_6_	291.0855	−4.55	5.5 × 10^7^/7 × 10^5^/5.5 × 10^4^	Epicatechin	72276	139.0388 (100%), 123.0440 (52.5%), 291.0857 (28.8%), 165.0544 (26.2%), and 147.0438 (55.3%)
8	4.46	C_24_H_15_N_2_O_5_	291.0974	−2.36	2.4 × 10^6^/1.5 × 10^8^/1.5 × 10^8^	Malonyltryptophan	5199636	188.0867 (100%), 245.0919 (91.2%), 130.0652 (40.7%), 227.0814 (27.9%), 273.0867 (18.6%), and 291.0979 (17.4%)
9	4.78	C_10_H_9_O_4_	193.0497	−2.1	1.9 × 10^7^/4.5 × 10^8^/3.3 × 10^8^	Scopoletin	5280460	193.0495 (100%), 194.0528 (10.6%), 133.0284 (4.4%), and 178.0260 (1.9%)
10	5.07/5.42	C_11_H_11_O_5_	223.0597	−4.48	4.4 × 10^6^/5.3 × 10^8^/6 × 10^7^/1.2 × 10^6^/1.3 × 10^8^/1.2 × 10^7^	Isofraxidin	5318565	223.0599 (100%), 224.0631 (11.2%), 208.0363 (4.6%), and 163.0387 (2.3%)
11	5.13	C_11_H_17_O_3_	197.1170	−4.11	1.4 × 10^8^/3.3 × 10^7^/1.1 × 10^7^	Loliolide	14334	197.1169 (100%), 179.1064 (83.7%), 135.1167 (44.4%), 104.0857 (19.9%), and 161.0958 (18.7%)
12	5.89	C_28_H_35_N_2_O_11_	575.2247	1.01	7.7 × 10^6^/1 × 10^8^/1.9 × 10^8^	5-Carboxystrictosidine	10483216	575.2227 (100%), 413.1701 (32.6%), 188.0704 (14%), 395.1589 (8.4%), and 165. 0811 (7.3%)
13	7.32	-	181.1223	-	2.8 × 10^7^/5.8 × 10^6^/5.2 × 10^6^	Unknown	-	181.1221 (100%), 135.1167 (15.4%), 163.1115 (13.5%), 139.0752 (4.1), 121.1012 (4.1%), and 95.0858 (3.76)
14	8.30	-	367.1180	-	1.2 × 10^4^/2.9 × 10^7^/4.9 × 10^6^	Unknown	-	367.1172 (100%), 336.0986 (54.3%), 337.1018 (7.9%), 321.0753 (1.4%), and 352.0924 (1.1%)
15	10.08	-	335.2184	-	1.6 × 10^6^/4 × 10^7^/7.6 × 10^7^	Unknown	-	335.2193 (100%), 195.0990 (0.3%), 71.17 (0.2%), and 203.3772 (0.1%)
16	10.79	C_18_H_31_O_2_	279.2315	−3.31	1.7 × 10^6^/2.7 × 10^7^/7.3 × 10^7^	Linolenic acid	5280934	279.2315 (100%), 95.0859 (44.1%), 81.0704 (43.9%), 67.0548 (26.7%), 109.1014 (23.5%), and 123.1169 (20.8%)

**Table 2:** Characterization of the major chromatogram peaks from leaf, stem and wood bark water extracts of *Capirona macrophylla* by UHPLC/HRMS^n^ on ESI negative acquisition mode. The compounds were identified by MS-DIAL and GNPS software.

ID	t_R_ (min) Average	Formula [M-H]−	Experimental Mass *m*/*z*	Error (ppm)	Peak Area Leaf/Stem/Wood Bark	Name	CID PubChem	MS^2^ Ions and Percentages (*m*/*z,* %)
1	0.28 2.63	C_7_H_11_O_6_	191.0551	−2.54	5.9 × 10^7^/7.3 × 10^7^/1.5 × 10^8^/5.4 × 10^8^/1.3 × 10^9^/1.2 × 10^9^	Quinic acid	6508	191.0546 (100%), 85.0278 (2.5%), 173.0440 (0.9%), 127.0385 (0.9%) and 93.0329 (0.7%)
2	1.08	C_16_H_17_O_9_	353.0870	−0.62	6 × 10^7^/7.3 × 10^7^/2.4 × 10^8^	5-O-CQA *	5280633	353.0867 (100%), 191.0546 (72.3%), 179.0334 (49.2%) and 135.0434 (8.03%)
3	2.63	C_16_H_17_O_9_	353.0873	0.06	1.4 × 10^8^/1.8 × 10^8^/3.7 × 10^8^	3-O-CQA *	1794427	191.0545 (100%), 353.0864 (19.9%), 179.0337 (4.5%), 192.0579 (3.5%) and 161.7553 (1%)
4	2.90	C_16_H_17_O_9_	353.0867	−1.5	7.1 × 10^7^/7.9 × 10^7^/2.2 × 10^8^	4-O-CQA *	9798666	173.0438 (100%), 353.0864 (89.8%), 179.0333 (68.3%), 191.0545 (39%) and 135.0434 (13.5%)
5	3.30	C_30_H_25_O_12_	577.1356	1.76	3.1 × 10^7^/3 × 10^3^/2.1 × 10^3^	Procyanidin B2	122738	577.1346 (100%), 425.0876 (50.1%), 289.0715 (40.8%), 407.0769 (37.4%) and 125.0230 (33%)
6	3.85	C_15_H_13_O6	289.0716	−0.81	3.6 × 10^7^/4.1 × 10^4^/5.5 × 10^3^	Epicatechin	1203	289.0715 (100%), 245.0813 (20.3%), 125.0229 (5.6%), 205.0496 (5.5%) and 179.0339 (5%)
7	4.48	C_13_H_13_N_2_O_3_	245.0923	−1.42	1.7 × 10^7^/6.2 × 10^7^/6.8 × 10^7^	N-Acetyltryptophan	2002	245.0921 (100%), 203.0811 (64%), 74.0231 (27.3%) and 201.1022 (8.1%)
8	4.56	C_17_H_19_O_9_	367.1026	−0.84	1.8 × 10^7^/2.2 × 10^7^/6.1 × 10^7^	3-O-Feruloylquinic acid	9799386	173.0443 (100%), 191.0546 (76.7%), 367.1020 (50%), 193.0491 (18.7%) and 93.0329 (7%)
9	5.22	-	581.2208	-	5.6 × 10^4^/7.8 × 10^5^/8.3 × 10^6^	Unknown	-	581.2225 (100%), 419.1700 (12.9%), 101.0227 (4.3%), 153.0542 (3.3%)
10	5.41	C_26_H_27_O_16_	595.1309	1.69	8.4 × 10^6^/4.8 × 10^5^/1 × 10^4^	Quercetin-3-O-vicianoside	13887800	595.1301 (100%), 300.0271 (52.2%), 301.0329 (10.9%), 178.9975 (0.9%) and 302.0391 (0.6%)
11	5.74	C_25_H_23_O_12_	515.1192	0.48	2.5 × 10^7^/1.6 × 10^7^/9.1 × 10^6^	1,5-DiCQA **	122685	515.1188 (100%), 353.0874 (60%), 179.0338 (32.3%), 173.0443 (25.3%) and 191.0550 (24.9%)
12	5.89	C_27_H_29_O_16_	609.1456	0.11	2.4 × 10^7^/8.6 × 10^5^/2.2 × 10^5^	Quercetin-3-O-rutinose	5293655	609.1452 (100%), 300.0271 (32.8%), 301.0343 (32.8%), 67.4327 (2.4%) and 302.0387 (1.1%)
13	6.19	C_25_H_23_O_12_	515.1193	0.71	2.2 × 10^7^/8.7 × 10^6^/3.5 × 10^6^	3,4-DiCQA **	3802778	515.1189 (100%), 353.0874 (72.1%), 173.0443 (41.8%), 179.0338 (29.2%) and 191.0549 (9.4%)
14	6.27	-	187.0964	-	3.6 × 10^6^/2.4 × 10^7^/6.8 × 10^7^	Unknown	-	125.0957 (100%), 126.0991 (5.4%), 169.0857 (5.0%) and 143.1065 (1.8%)
15	6.30	C_33_H_39_O_19_	739.2066	−2.65	4.8 × 10^5^/2.2 × 10^6^/3.7 × 10^6^	Kaempferol-3-O-robinoside-7-O-rhamnoside	5351997	739.2043 (100%), 577.1774 (75.3%), 173.0442 (30.2%), 578.1737 (21.1%) 191.0555 (20.3)
16	6.91	-	241.1077	-	4.2 × 10^5^/7.2 × 10^5^/3.2 × 10^7^	Unknown	-	241.1065 (100%), 197.1165 (74.5%), 67.5990 (6.8%) and 179.1060 (6.6%)
17	7.25	C_10_H_17_O_4_	201.1123	−1.96	1.4 × 10^6^/1.1 × 10^7^/2.3 × 10^7^	Decanedioic acid	5192	201.1118 (100%), 67.3854 (24.8%), 139.1109 (8.7%), 183.1008 (7.7%%) and 111.0799 (5.4%)
18	8.80	C_18_H_33_O_5_	329.2322	−1.79	5.4 × 10^6^/5.5 × 10^7^/6.3 × 10^7^	FA 18:1 + 3O	153001	329.2323 (100%), 171.1010 (6.6%), 211.1326 (4.6%), 229.1432 (3.86%) and 311.2209 (0.5%)
19	10.08	C_18_H_31_O_4_	311.2223	0.18	1.3 × 10^6^/2.9 × 10^7^/7.2 × 10^7^	FA 18:2 + 2O	1928	311.2218 (100%), 171.1011 (24.9%), 293.2113 (10.9%), 185.1168 (5.9%) and 201.1118 (4.7%)
20	10.79	-	295.2260	-	4.8 × 10^5^/1.5 × 10^7^/4.2 × 10^7^	Unknown	-	183.1373 (100%), 277.2157 (71.4%), 68.0670 (20.5%) and 171.1007 (19.6%)

* CQA (Caffeoylquinic Acid); ** DiCQA—Dicaffeoylquinic Acid.

**Table 3:** Docking energies of the metabolites present in the samples on the DHODH of *Leishmania major* and *Plasmodium falciparum*.

DHODH *Leishmania major*			DHODH *Plasmodium falciparum*		
CID	Name	kcal/mol	CID	Name	kcal/mol
5281780	3,4-Dicaffeoylquinic acid	−8.78	**51347395**	DSM265 *	**−9.82**
**1492348**	Orotate (ORO*)	**−8.56**	5280863	Kaempferol	−8.82
9799386	3-Feruloylquinic acid	−8.35	5280343	Quercetin	−8.62
12358846	1,4-Dicaffeoylquinic acid	−8.08	161313	Anabasamine	−8.57
10466307	Loganetin	−7.41	6476139	Methyl chlorogenate	−7.78
6476139	Methyl chlorogenate	−7.34	5282944	9-HODE	−7.59
14334	Loliolide	−7.08	1794427	3-Caffeoylquinic acid	−7.5
1794427	3-O-Caffeoylquinic acid	−7.02	348159	(-)-5-Caffeoylquinic acid	−7.43
348159	(-)-5-Caffeoylquinic acid	−6.77	9799386	3-Feruloylquinic acid	−7.38
58427569	4-O-Caffeoylquinic acid	−6.67	14334	Loliolide	−7.01
5280460	Scopoletin	−6.58	10466307	Loganetin	−6.99
5199636	N-Malonyltryptophan	−6.54	5199636	N-Malonyltryptophan	−6.95
23760099	Diderroside	−6.51	101756906	Diderroside methyl ester	−6.77
5280633	5-Caffeoylquinic acid	−6.28	58427569	4-Caffeoylquinic acid	−6.72
14158101	3-p-Coumaroylquinic acid	−6.18	5281780	3,4-Dicaffeoylquinic acid	−6.67
5280863	Kaempferol	−6.16	1148	DL-Tryptophan	−6.45
5280343	Quercetin	−6.12	5460026	beta-Gentiobiose	−5.66
161313	Anabasamine	−5.98	12358846	1,4-Dicaffeoylquinic acid	−5.65
6508	Quinic acid	−5.75	5429	Theobromine	−5.59
1148	DL-Tryptophan	−5.52	798	Indole	−5.37
10483216	5(S)-5-Carboxystrictosidine	−5.51	23760099	Diderroside	−5.07
101756906	Diderroside methyl ester	−5.14	22563	Desethylatrazine	−5.07
5282944	9-HODE	−5.06	998	Phenylacetaldehyde	−4.9
5429	Theobromine	−4.96	135398660	Pterin	−4.69
5281769	1,5-Dicaffeoylqunic acid	−4.8	6508	Quinic acid	−4.65
5460026	beta-Gentiobiose	−4.69	75368781	4,5-Dycaffeoylquinic acid	−4.63
798	Indole	−4.65	5281769	1,5-Dicaffeoylquinic acid	−3.10
998	Phenylacetaldehyde	−4.51	10483216	5(S)-5-Carboxystrictosidine	−2.17

CID: PubChem compound identification; *: Redocking Value; The table is in order from the lowest to the biggest docking energy. ORO and DSM265 docking values (in bold) serve as references because they are the crystallized substrate and inhibitory ligand for *Lm*DHODH and *Pf*DHODH, respectively.

## Data Availability

The original contributions presented in the study are included in the article and Supplementary Material, further inquiries can be directed to the corresponding author.

## Supplementary Materials

The following supporting information can be downloaded at: https://www.mdpi.com/article/10.3390/metabo15030157/s1, Supplementary Material S1: Mass Spectra of the compounds identified in Table 1—ESI Positive; Supplementary Material S2: Mass Spectra of the compounds identified in Table 2—ESI Negative; Supplementary Material S3: Tables of previously identified metabolites in Mulateiro species with confirmation of identification in *Capirona macrophylla* samples.; Supplementary Material S4: Metabolites described in *Calycophyllum spruceanum* (mulateiro) found in *Capirona macrophylla* (mulateiro) samples; Supplementary Material S5: HPLC chromatogram with diode array detector showing the fractionation profile of the aqueous wood bark sample of *C. macrophylla.*; Supplementary Material S6: RMSD of the systems and number of interactions between the *Pf*DHODH and ligands 5280863 and DSM265; Supplementary Material S7: RMSD of the systems and number of interactions between the *Lm*DHODH and ligands 5281780 and ORO.

## Abbreviations

CID	PubChem Compound Identification
CQA	Caffeoylquinic Acid
D.E.	Docking Energy
DHODH	Dihydroorotate Dehydrogenase enzyme
DiCQA	Di Caffeoylquinic Acid
HESI	Heated Electrospray Ionization
GNPS	Global Natural Products Social Molecular Networking
HPLC	High Performance Liquid Chromatography
IC50	Half maximal inhibitory concentration
MeOH	Methanol
ORO	Orotate substrate
SisGen	Brazilian National System of Genetic Heritage Management and Associated Traditional Knowledge
UHPLC/HRMS^n^	Ultra High-Performance Liquid Chromatography/High Resolution Tandem Mass Spectrometry
