# Supplementary material for: Identification of Bioactive Metabolites of Capirona macrophylla by Metabolomic Analysis, Molecular Docking, and In Vitro Antiparasitic Assays
Source: Metabolites. 2025 Feb 26;15(3):157. doi: 10.3390/metabo15030157 (PMC11943490; doi:10.3390/metabo15030157)
Supplement: Supplementary file 1 [file metabolites-15-00157-s001.zip › Supplementary material S1.pptx]

## Slide 1
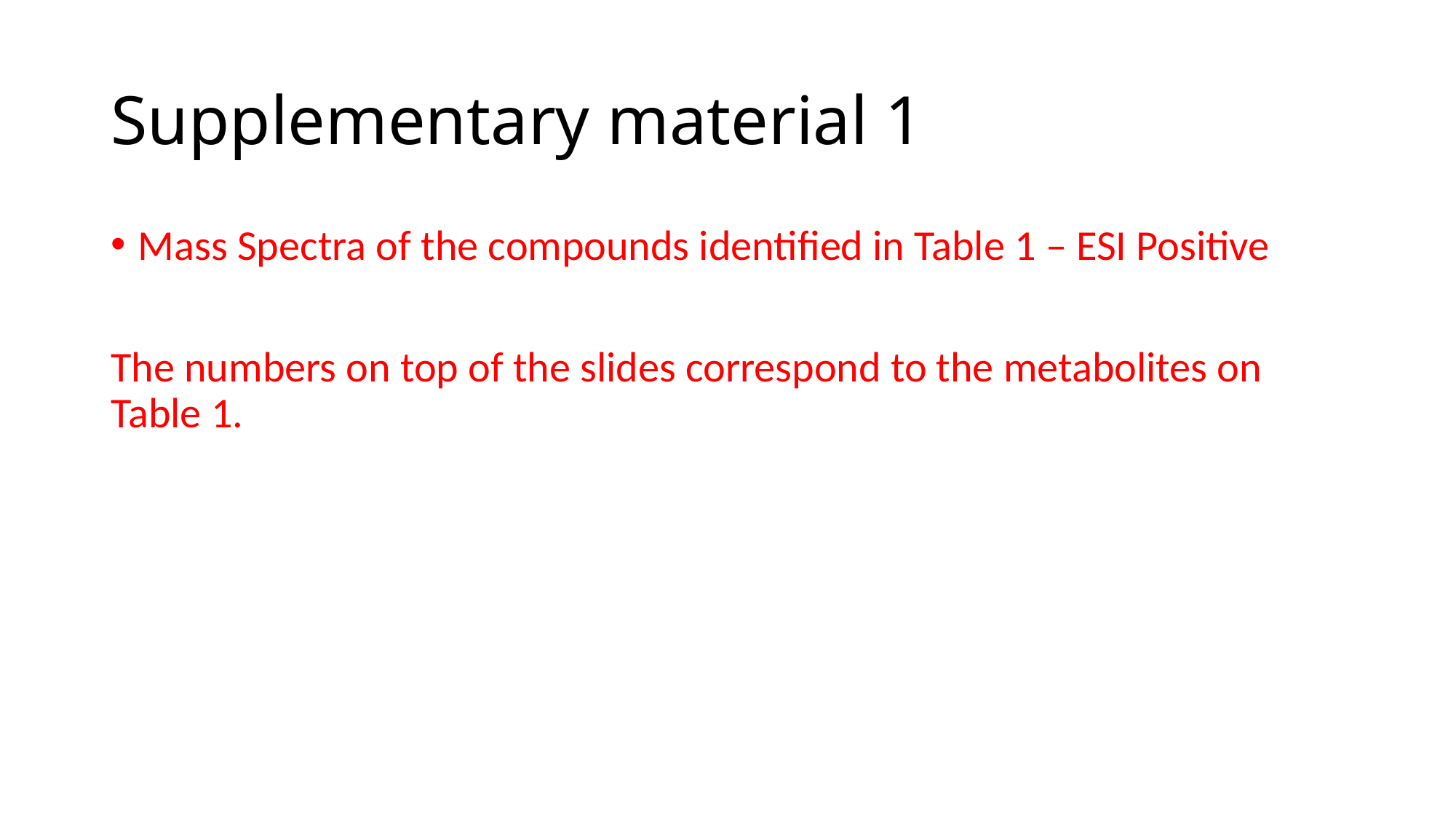

# Supplementary material 1
Mass Spectra of the compounds identified in Table 1 – ESI Positive
The numbers on top of the slides correspond to the metabolites on Table 1.

## Slide 2
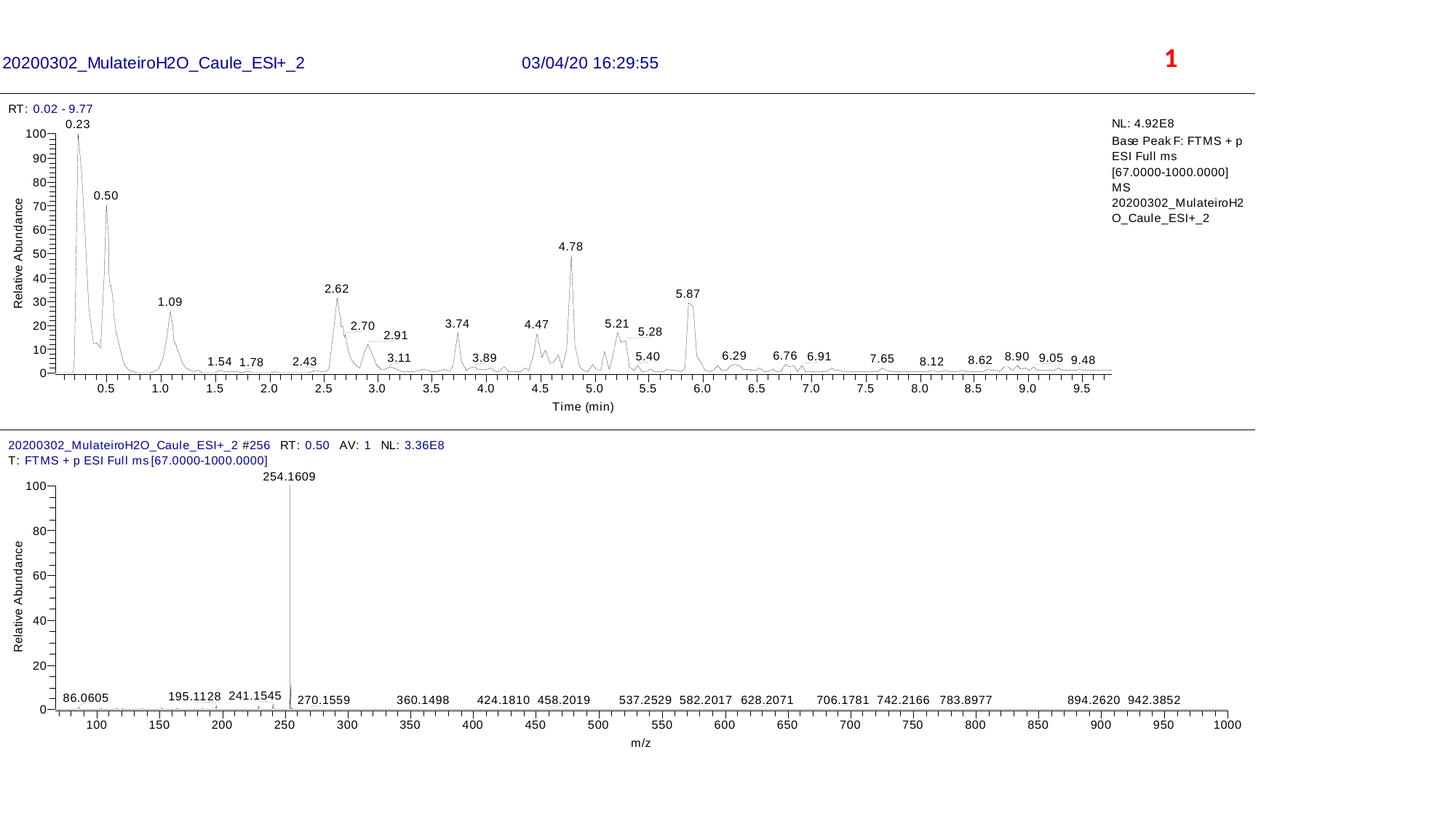

1

## Slide 3
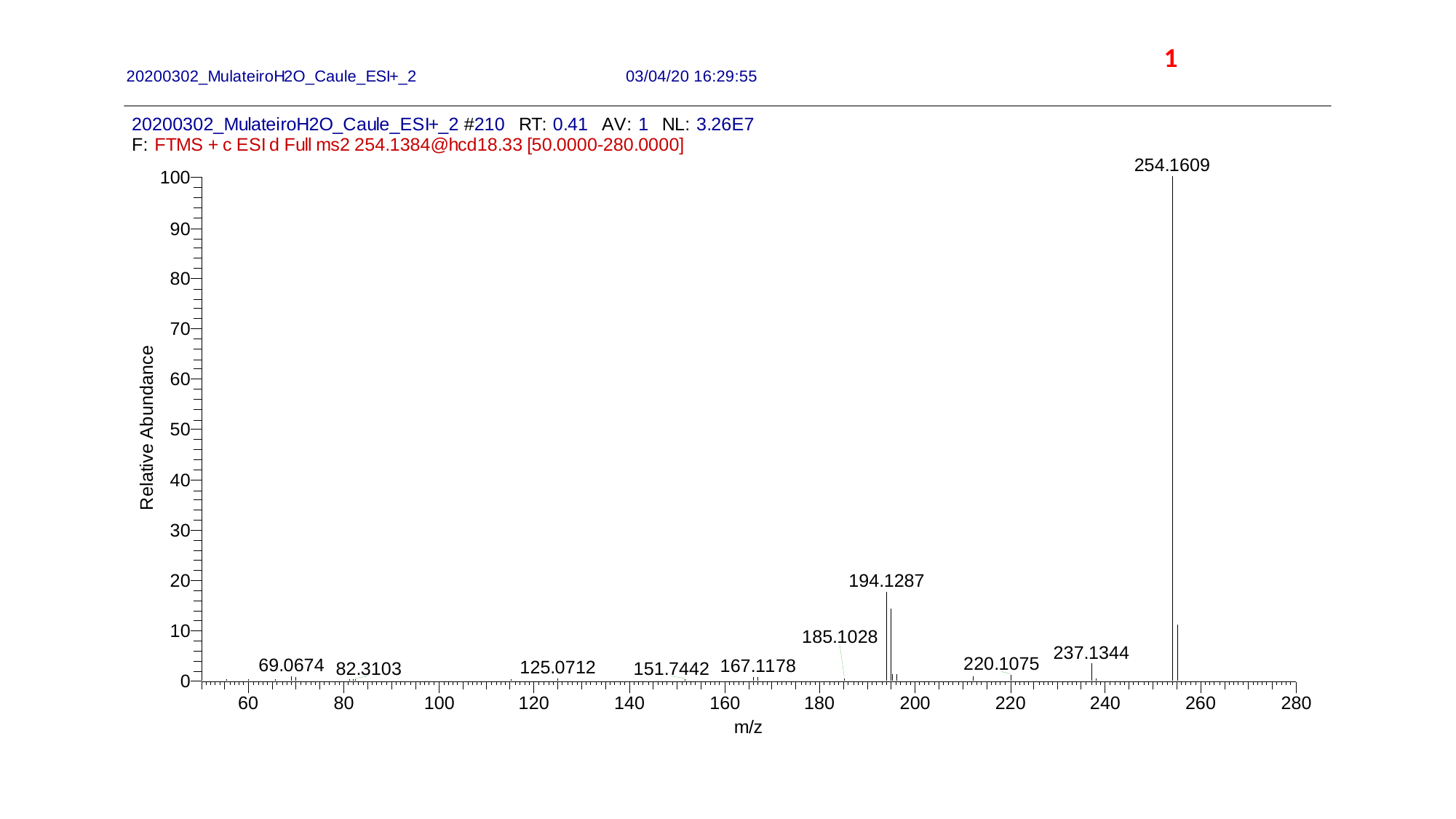

1

## Slide 4
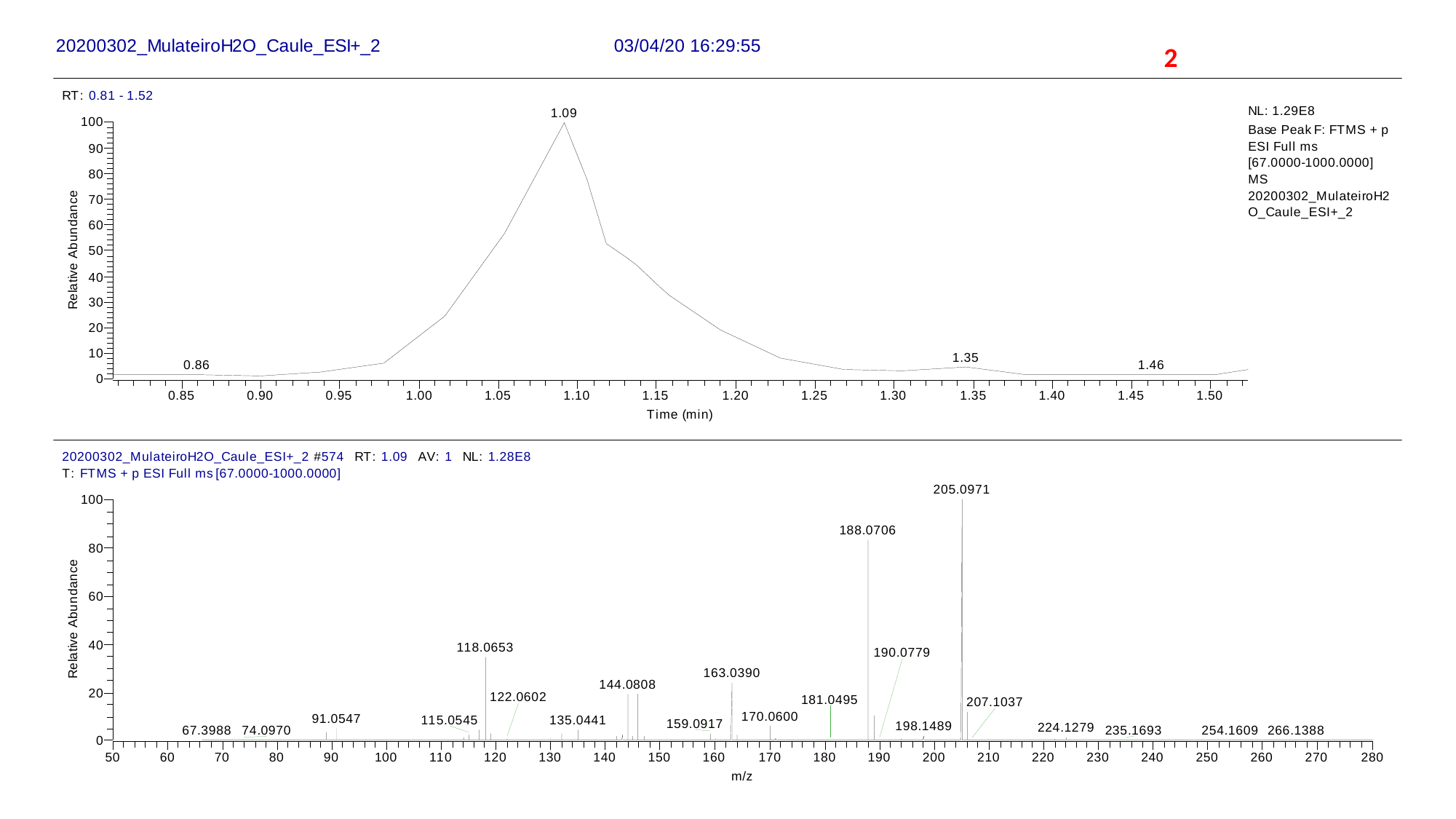

2

## Slide 5
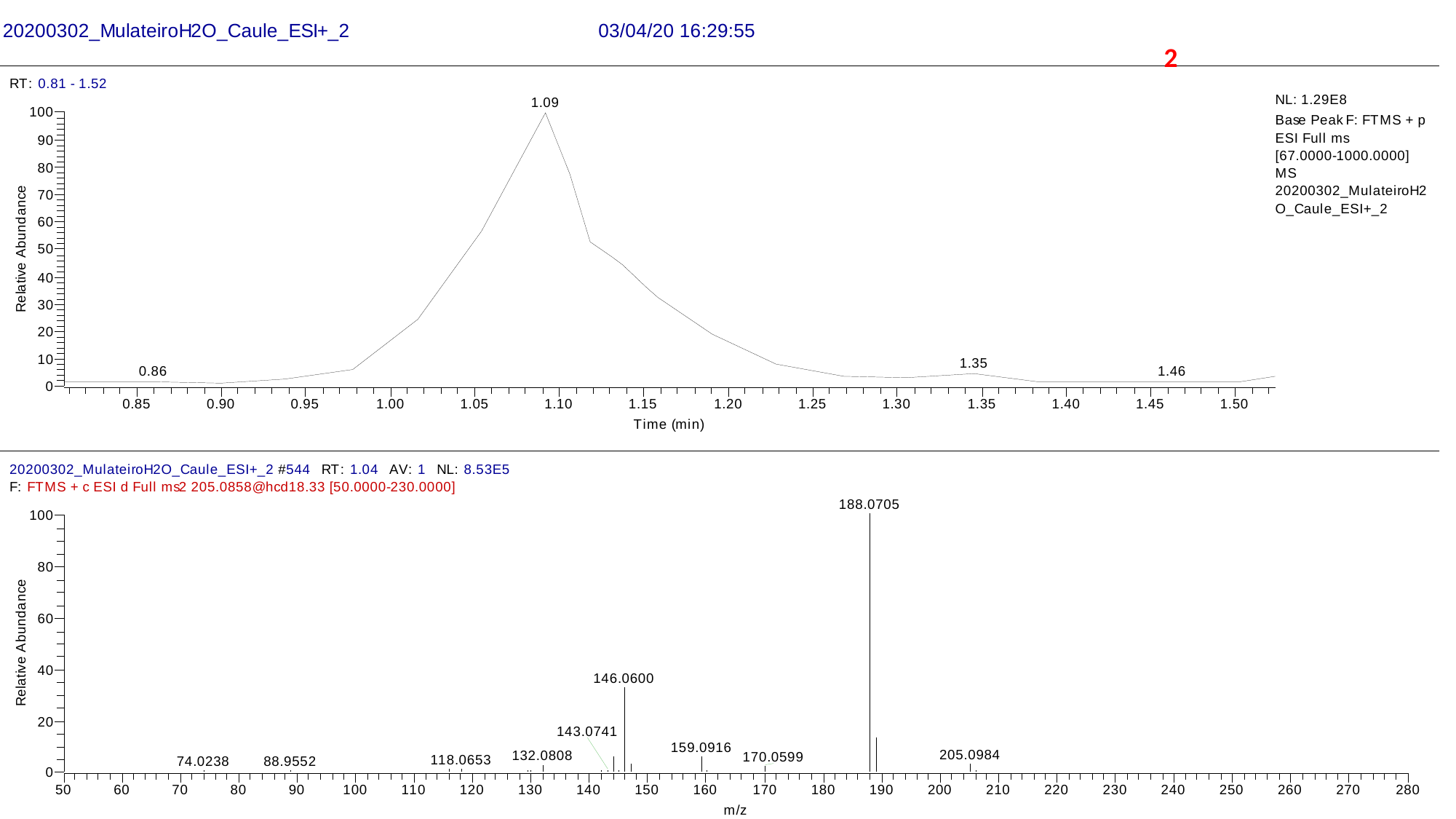

2

## Slide 6
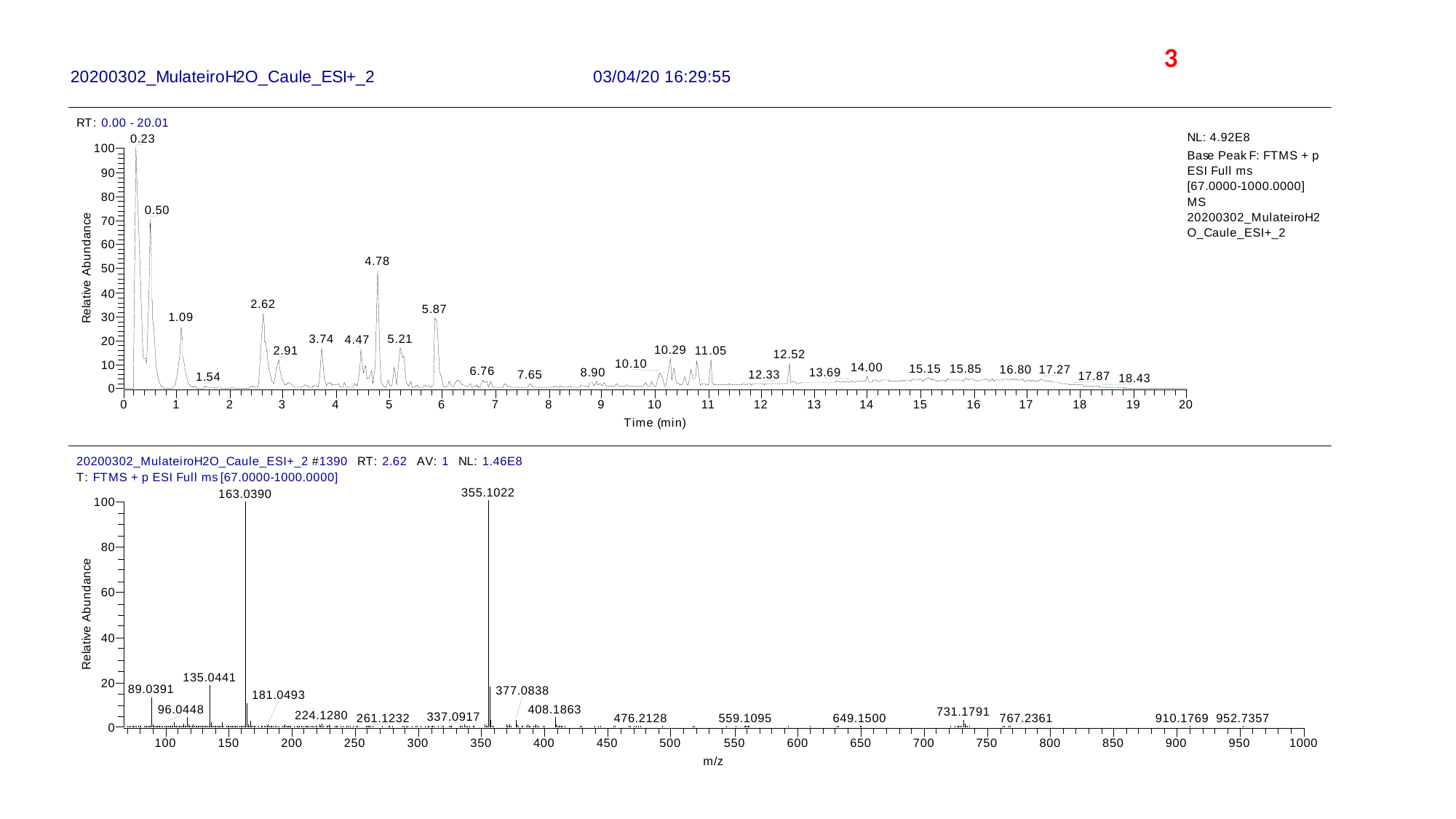

3

## Slide 7
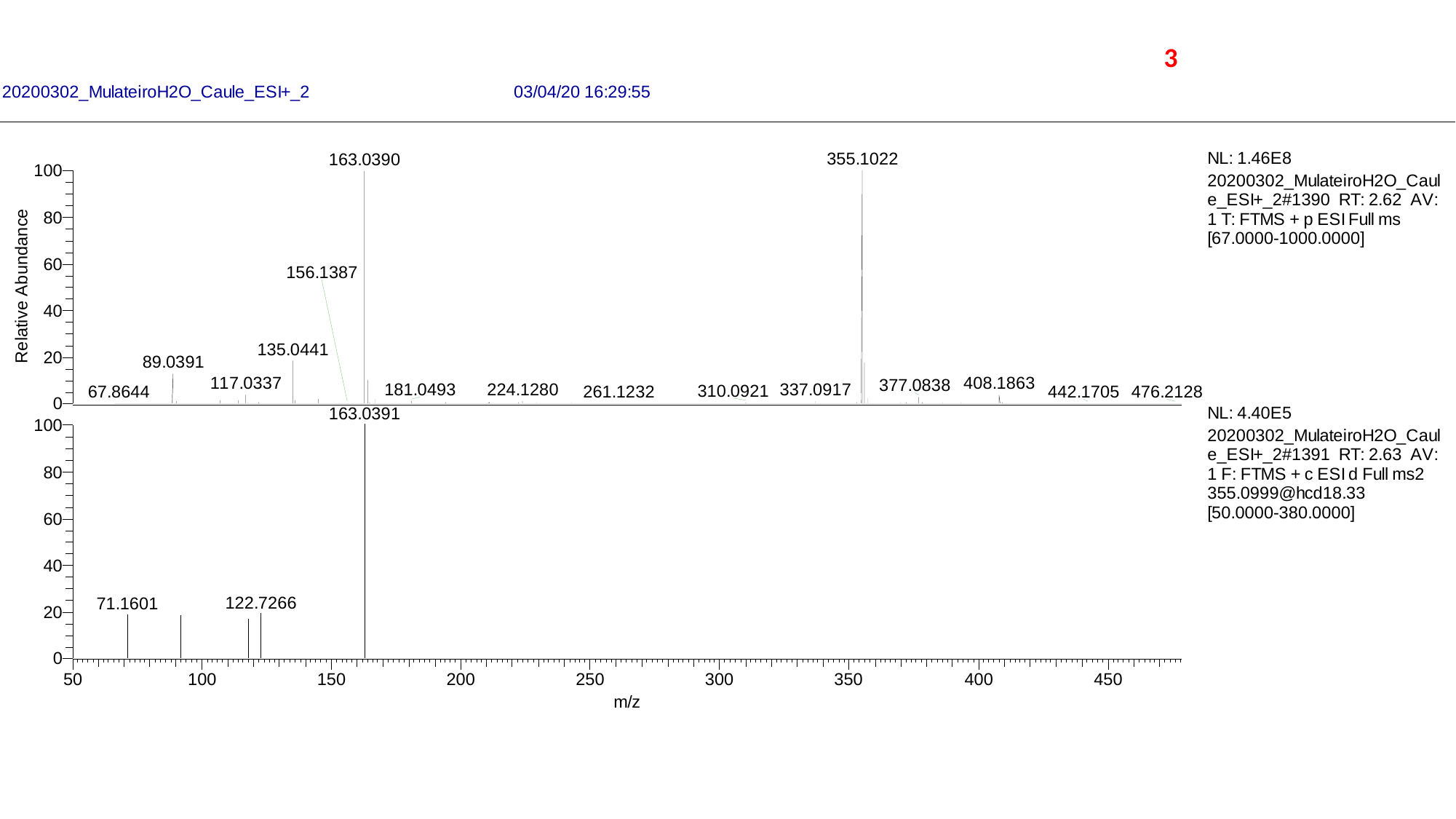

3

## Slide 8
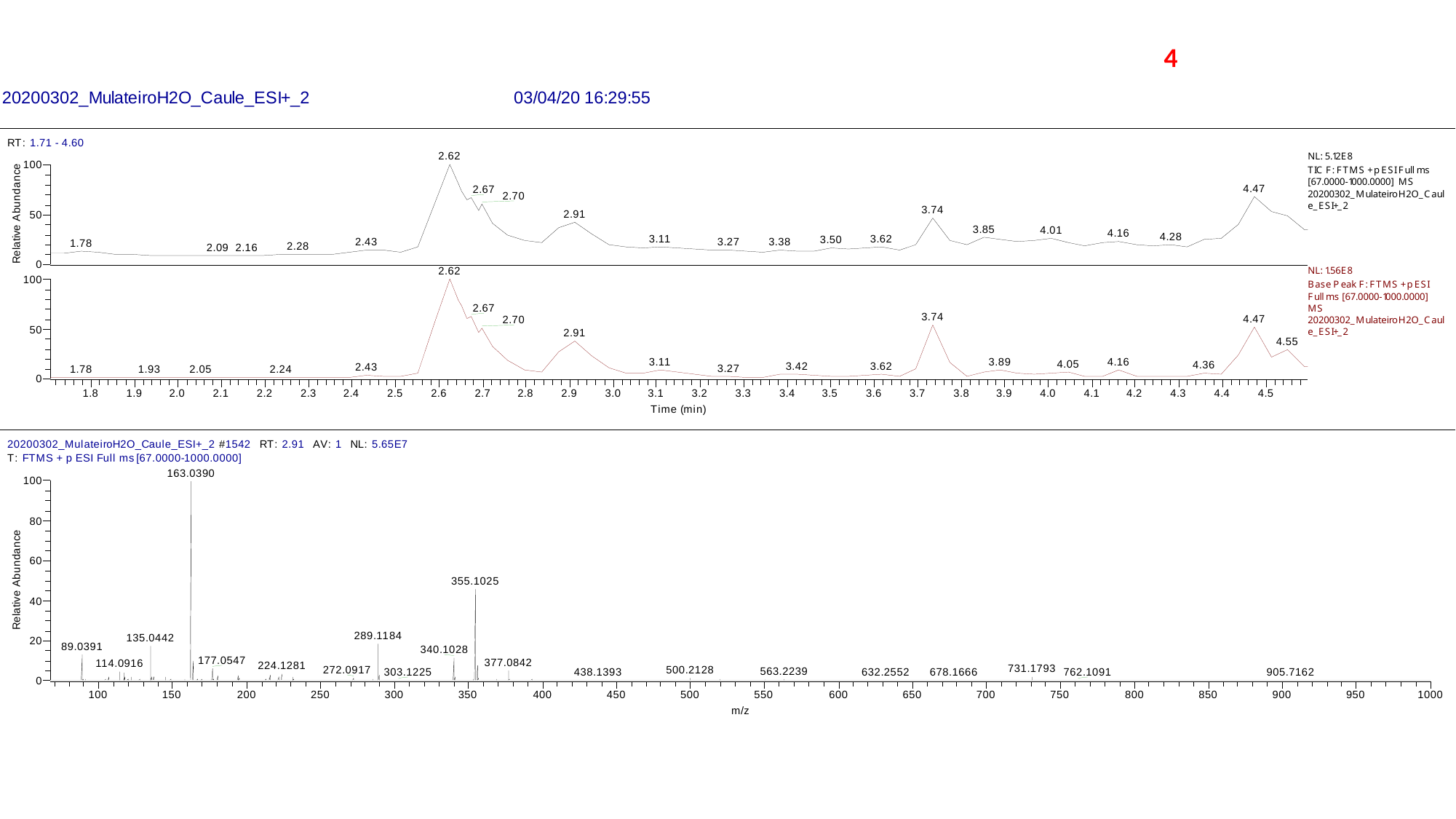

4

## Slide 9
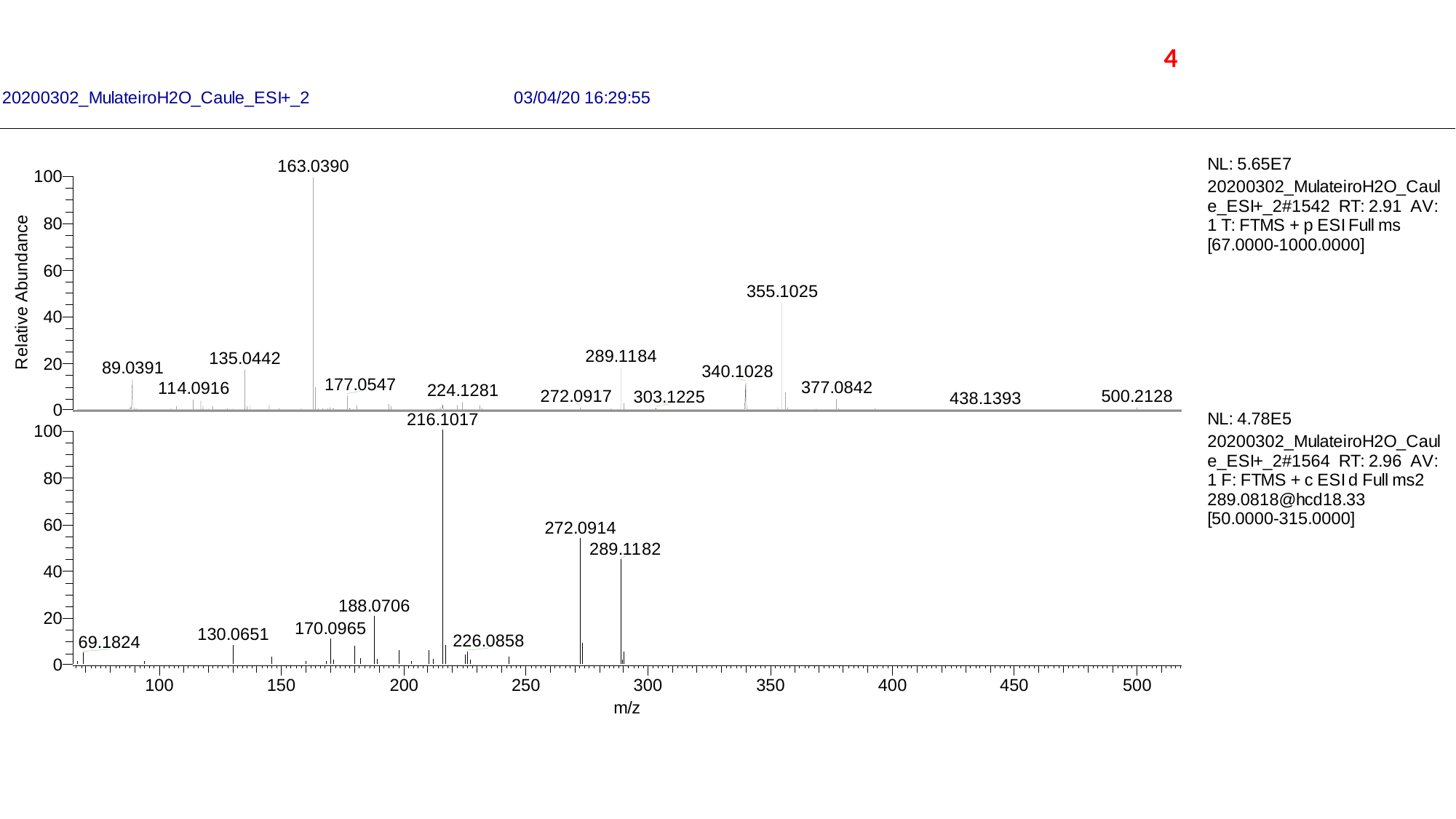

4

## Slide 10
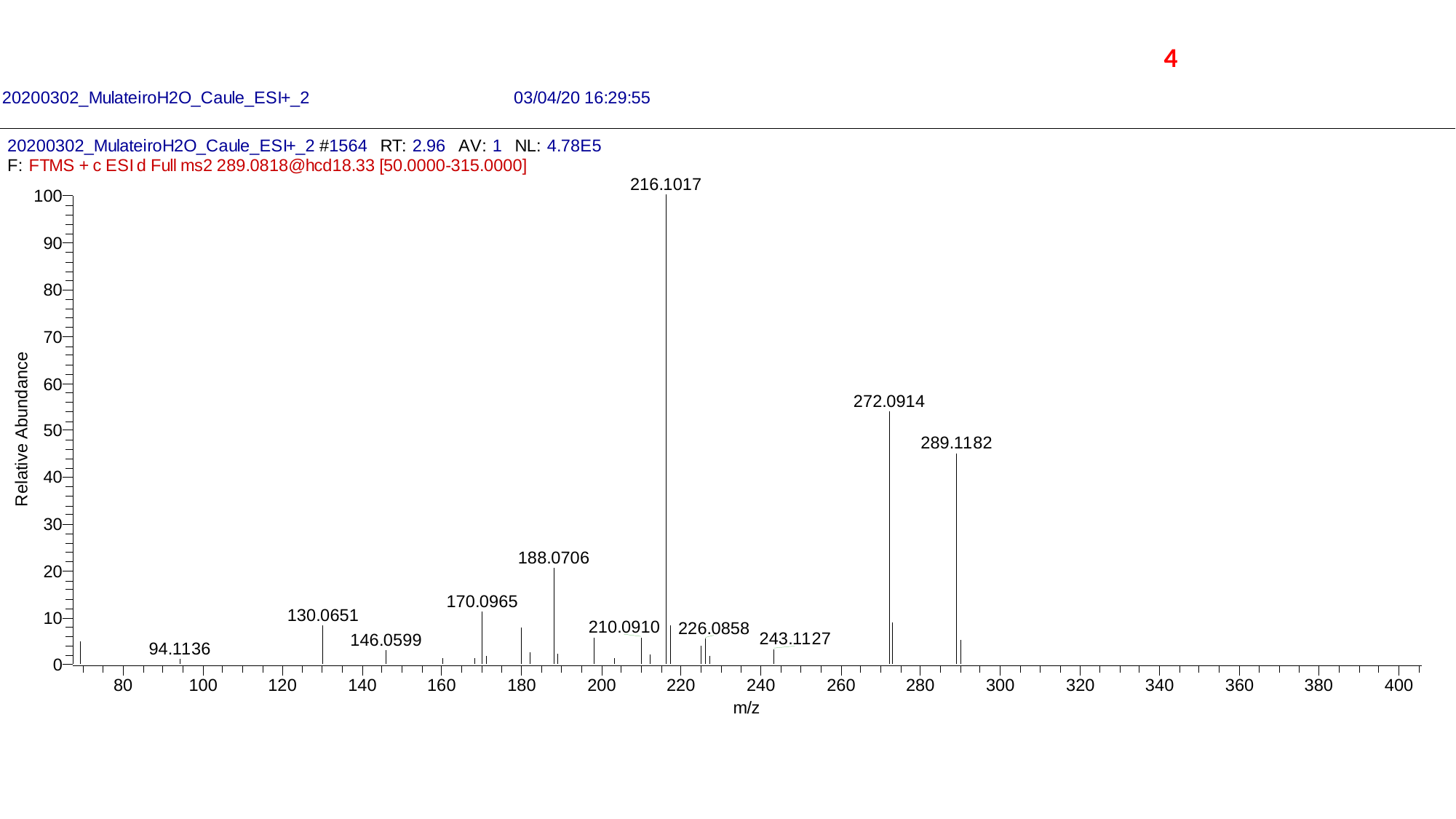

4

## Slide 11
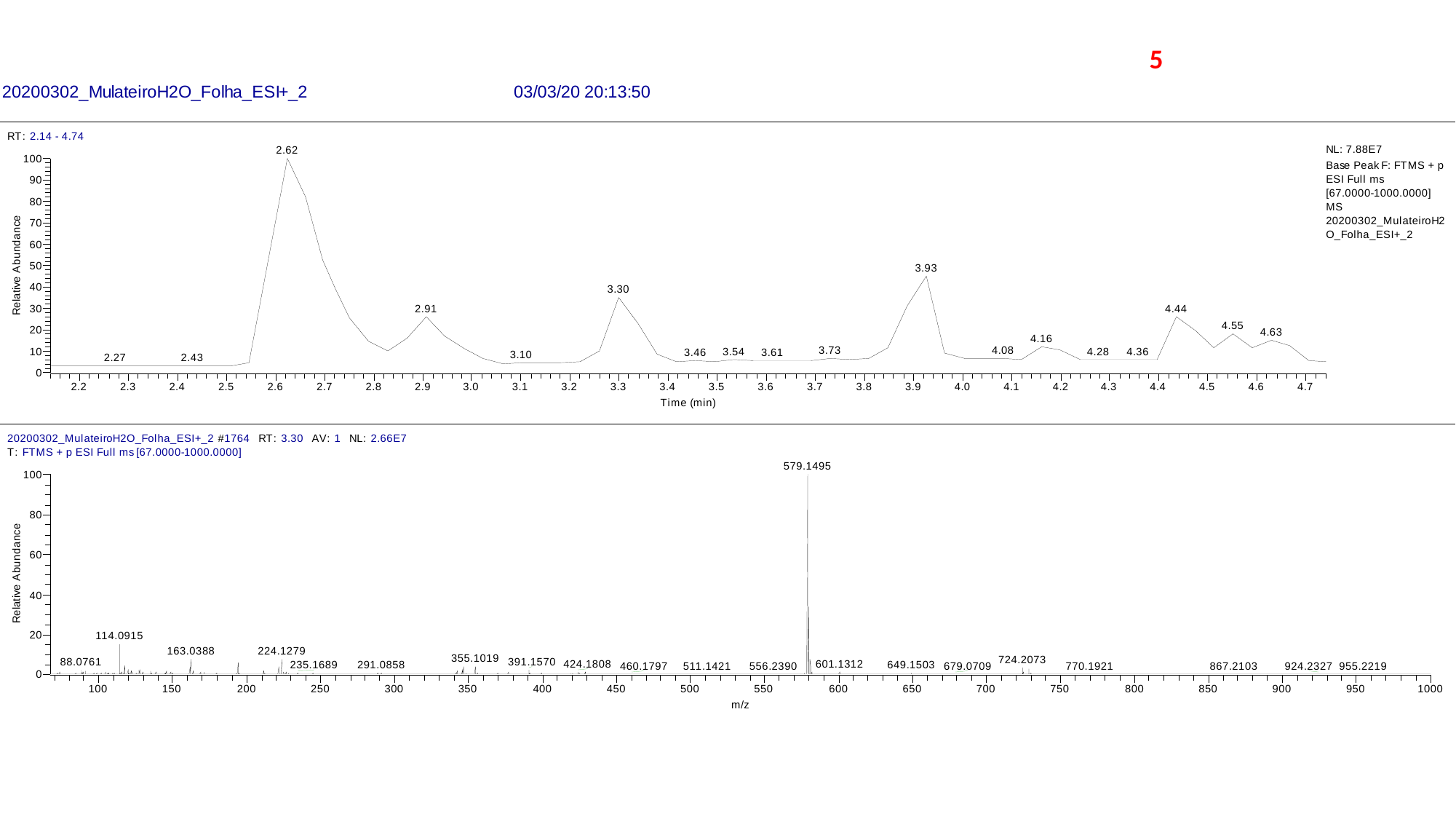

5

## Slide 12
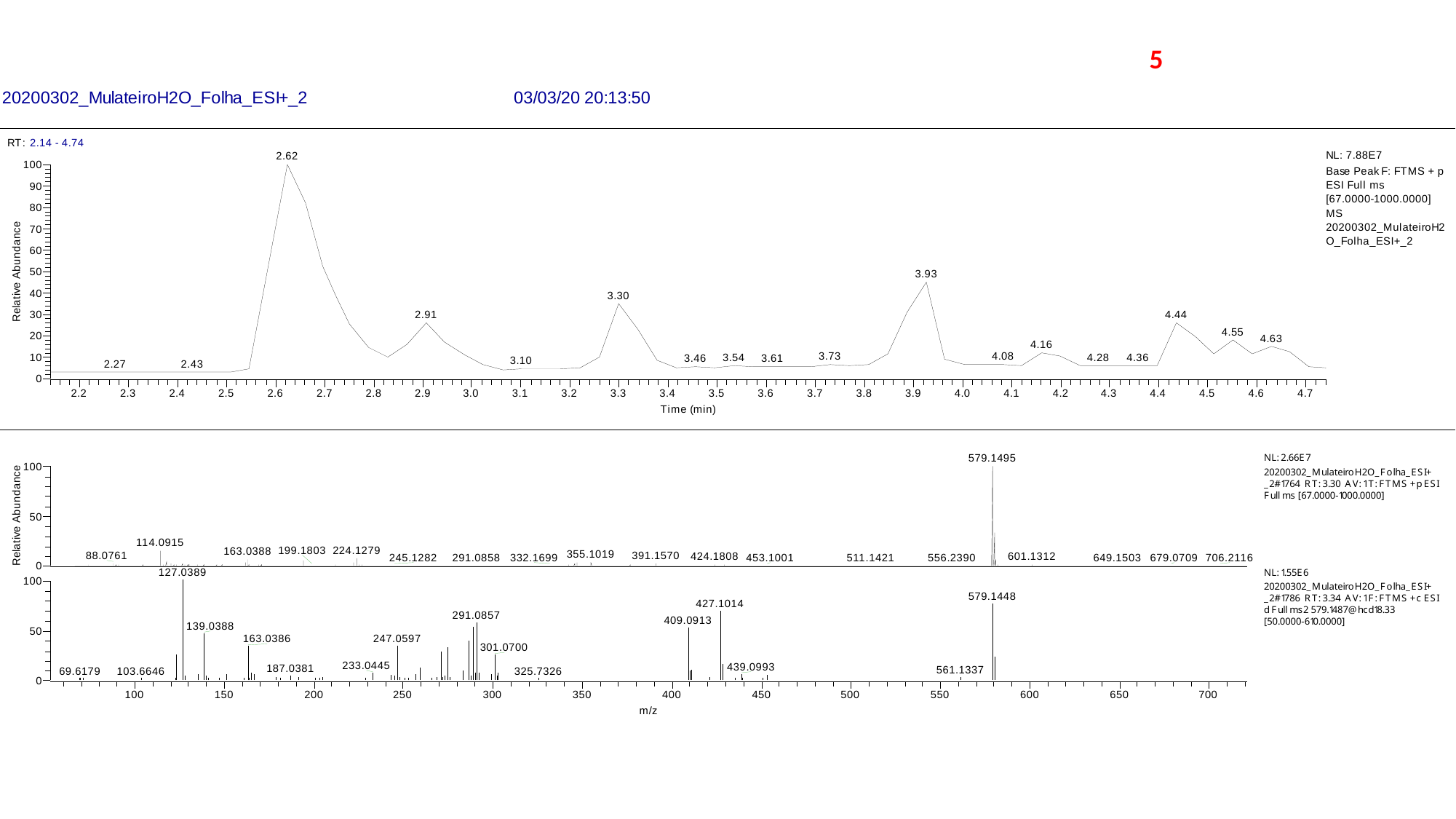

5

## Slide 13
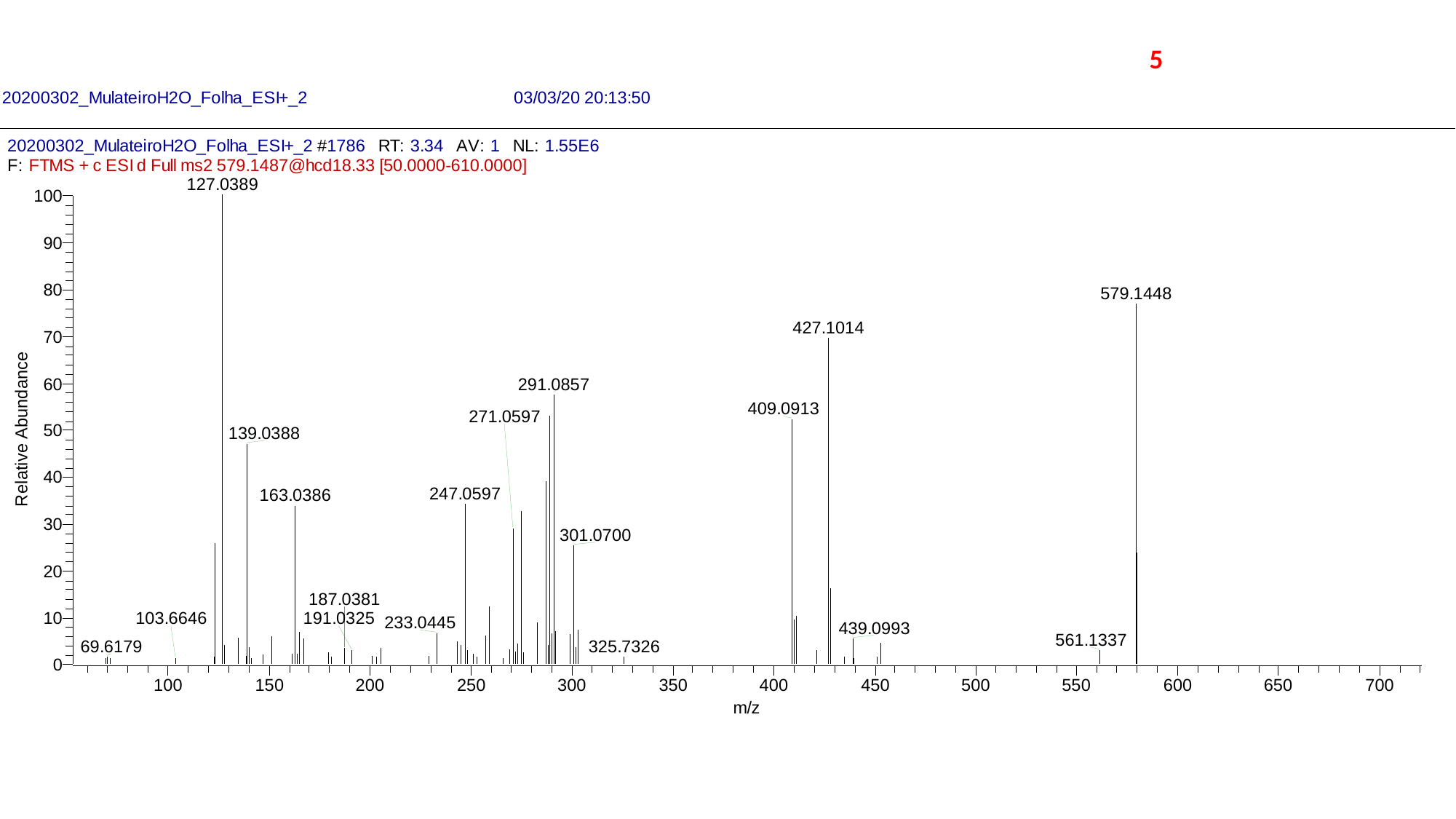

5

## Slide 14
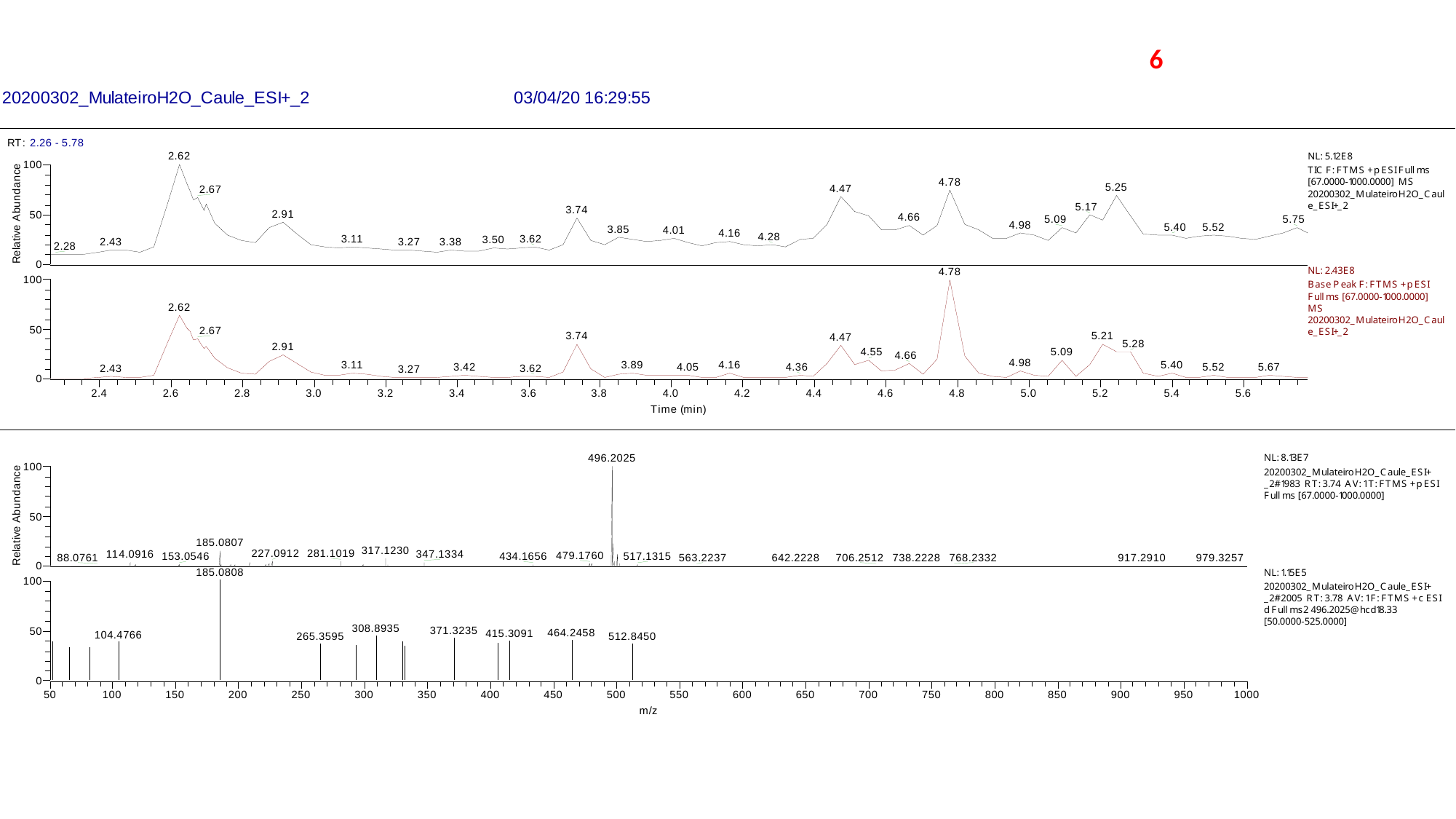

6

## Slide 15
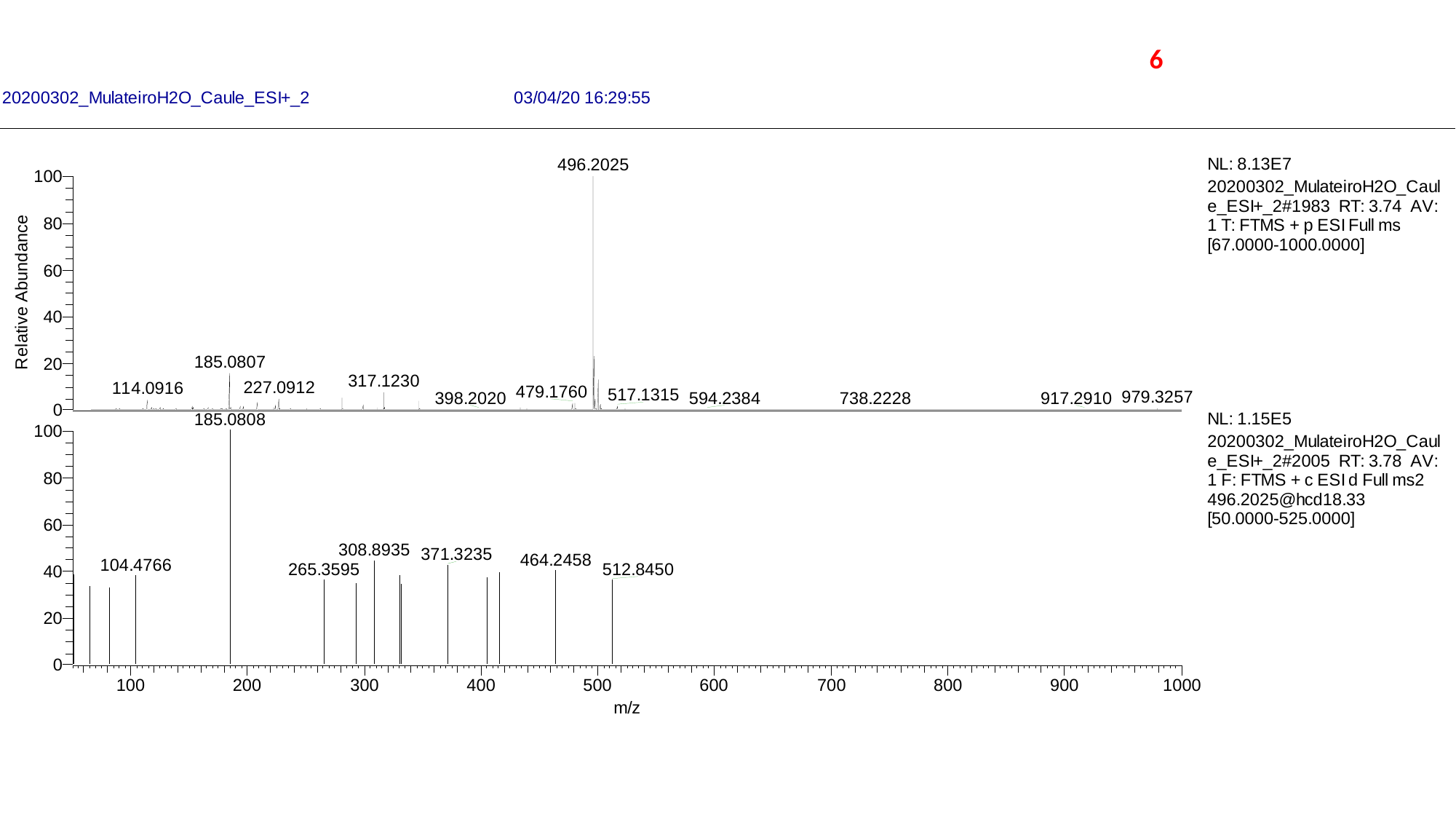

6

## Slide 16
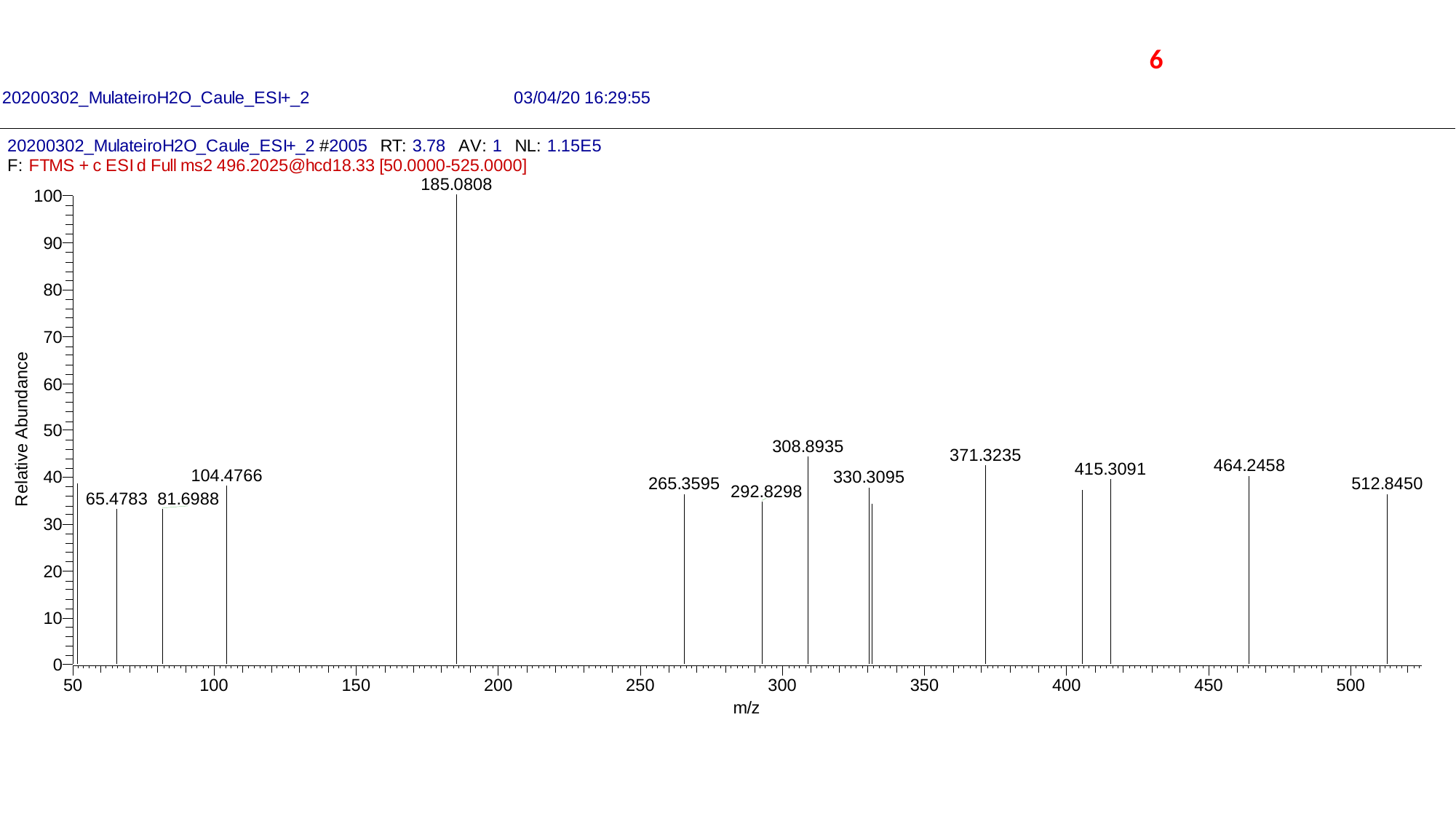

6

## Slide 17
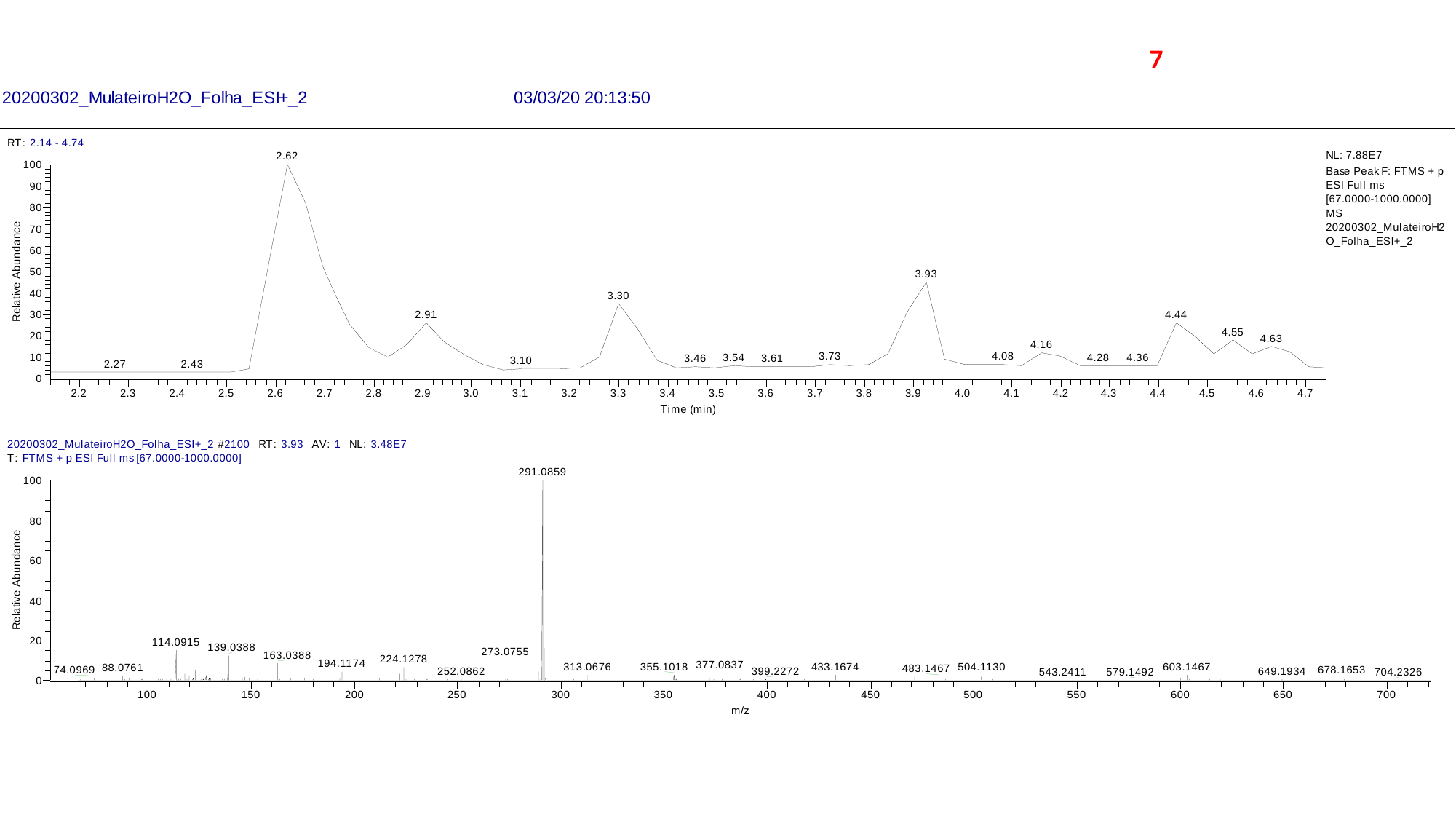

7

## Slide 18
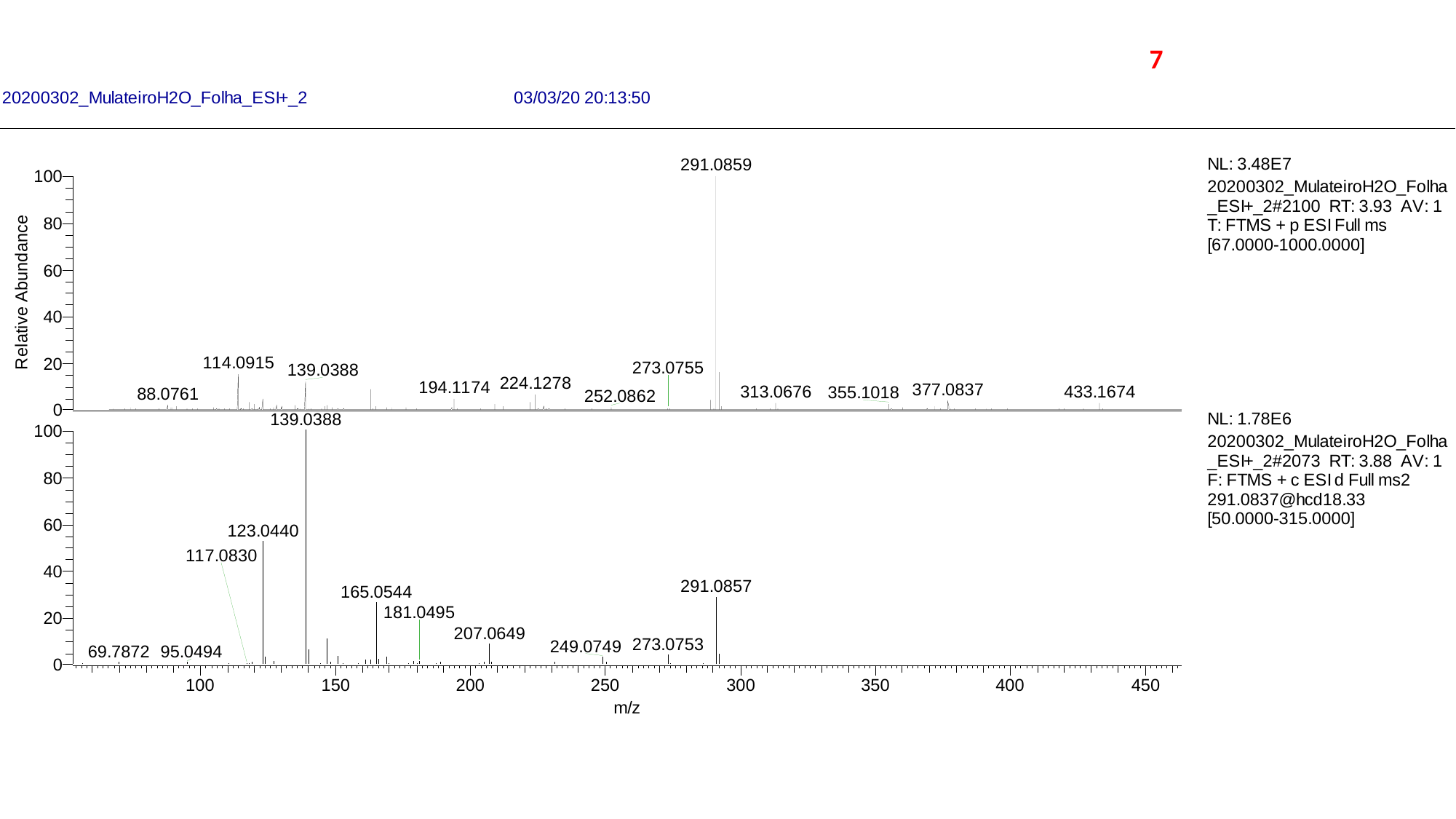

7

## Slide 19
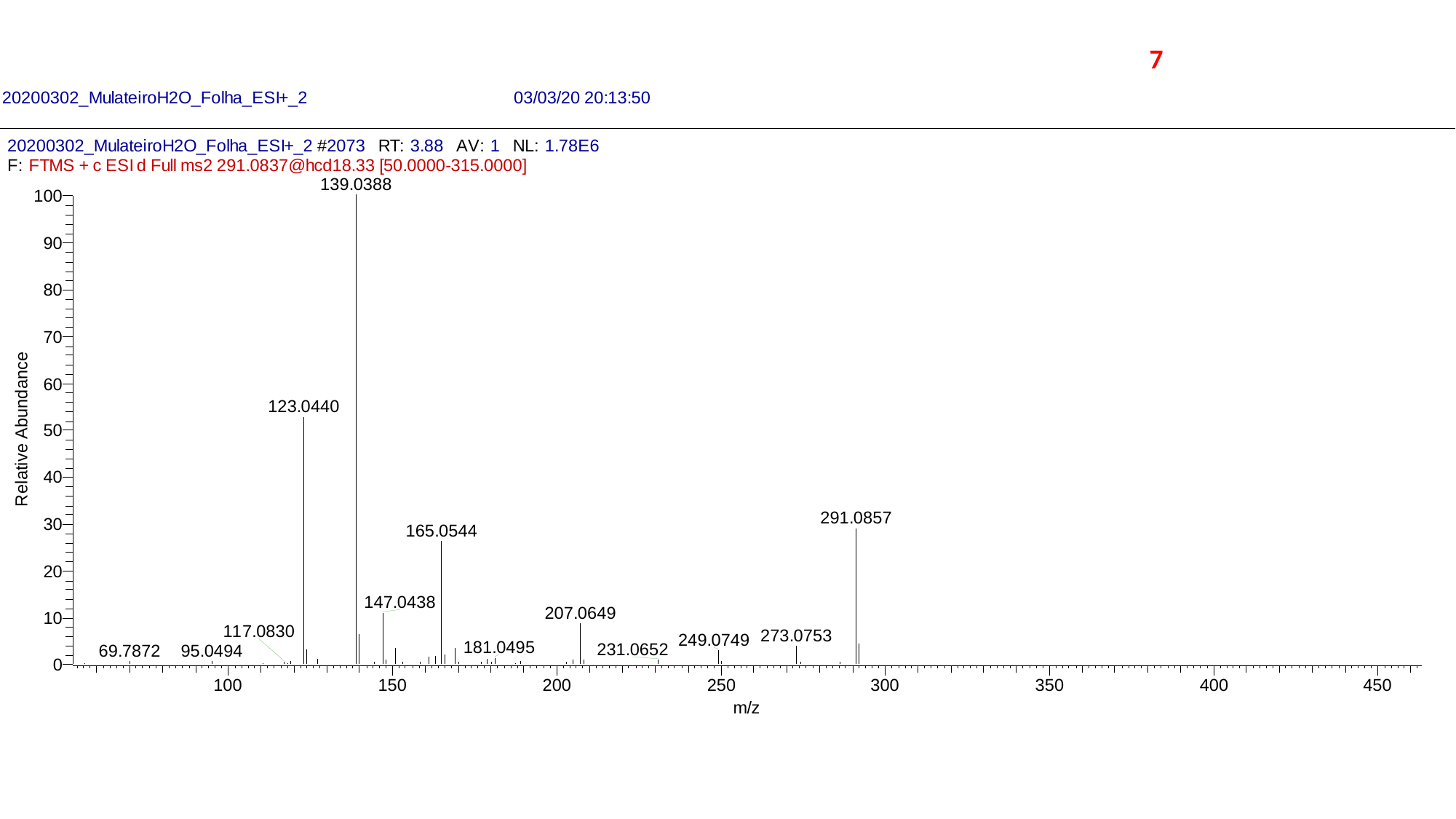

7

## Slide 20
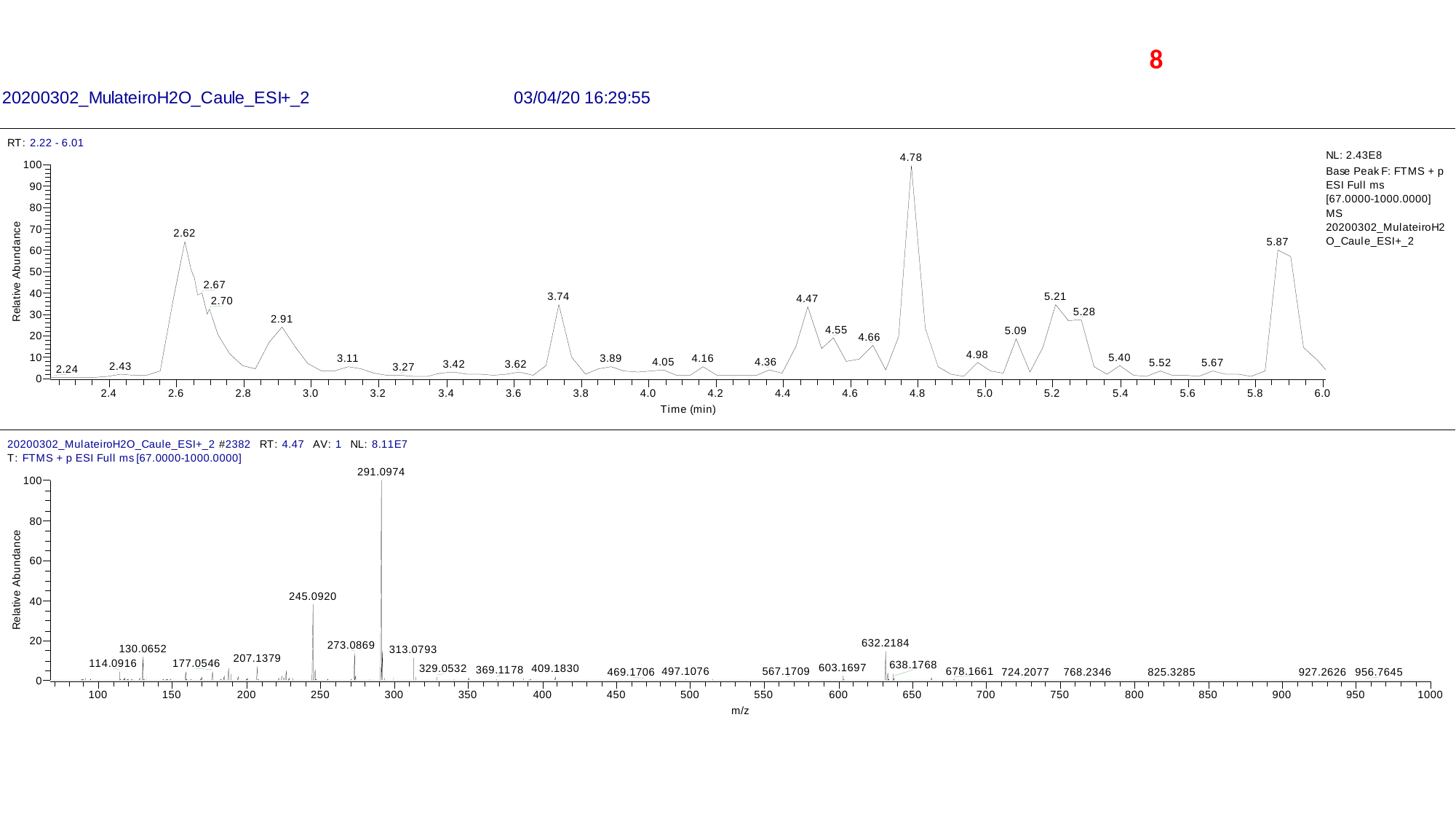

8

## Slide 21
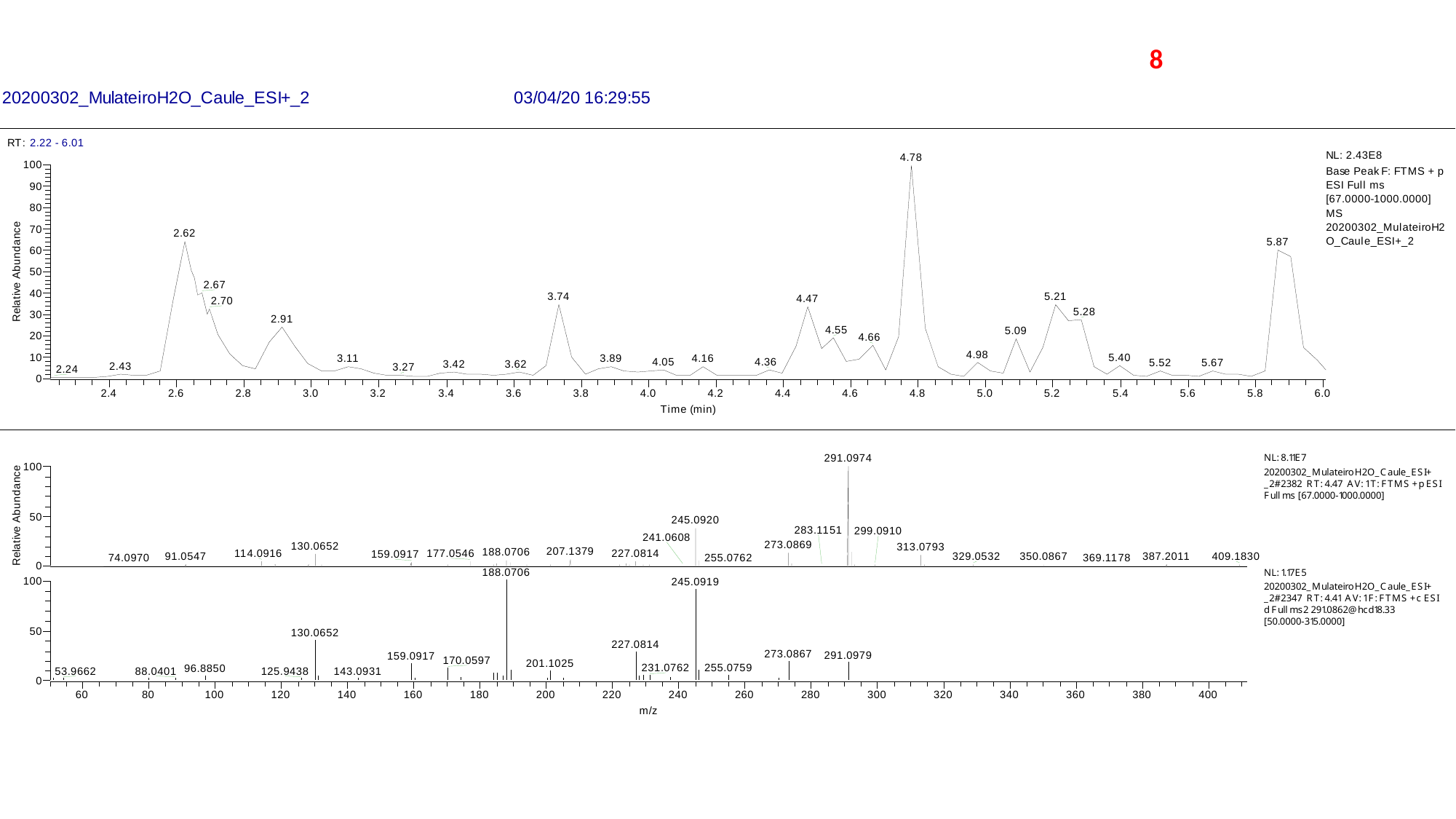

8

## Slide 22
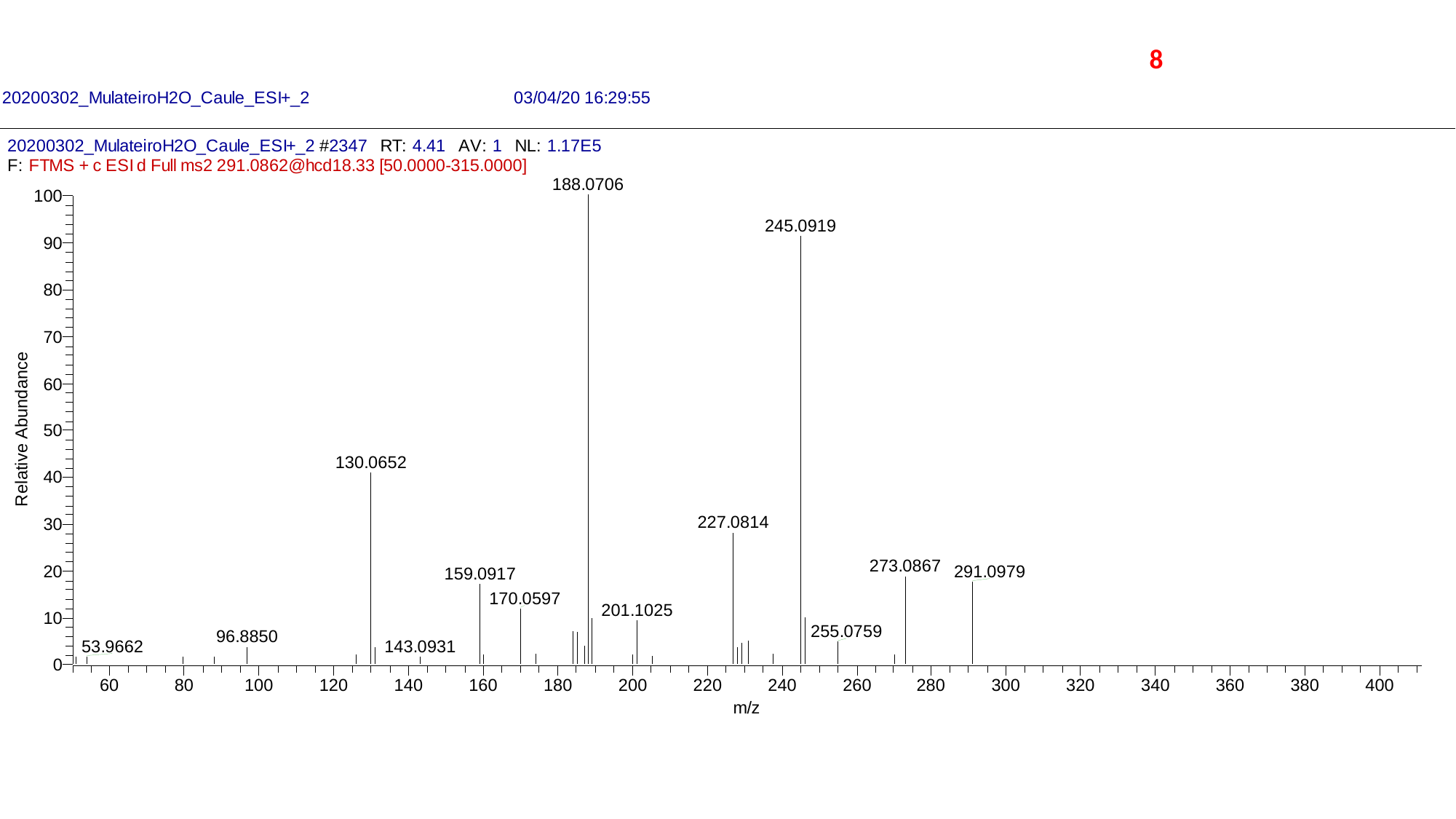

8

## Slide 23
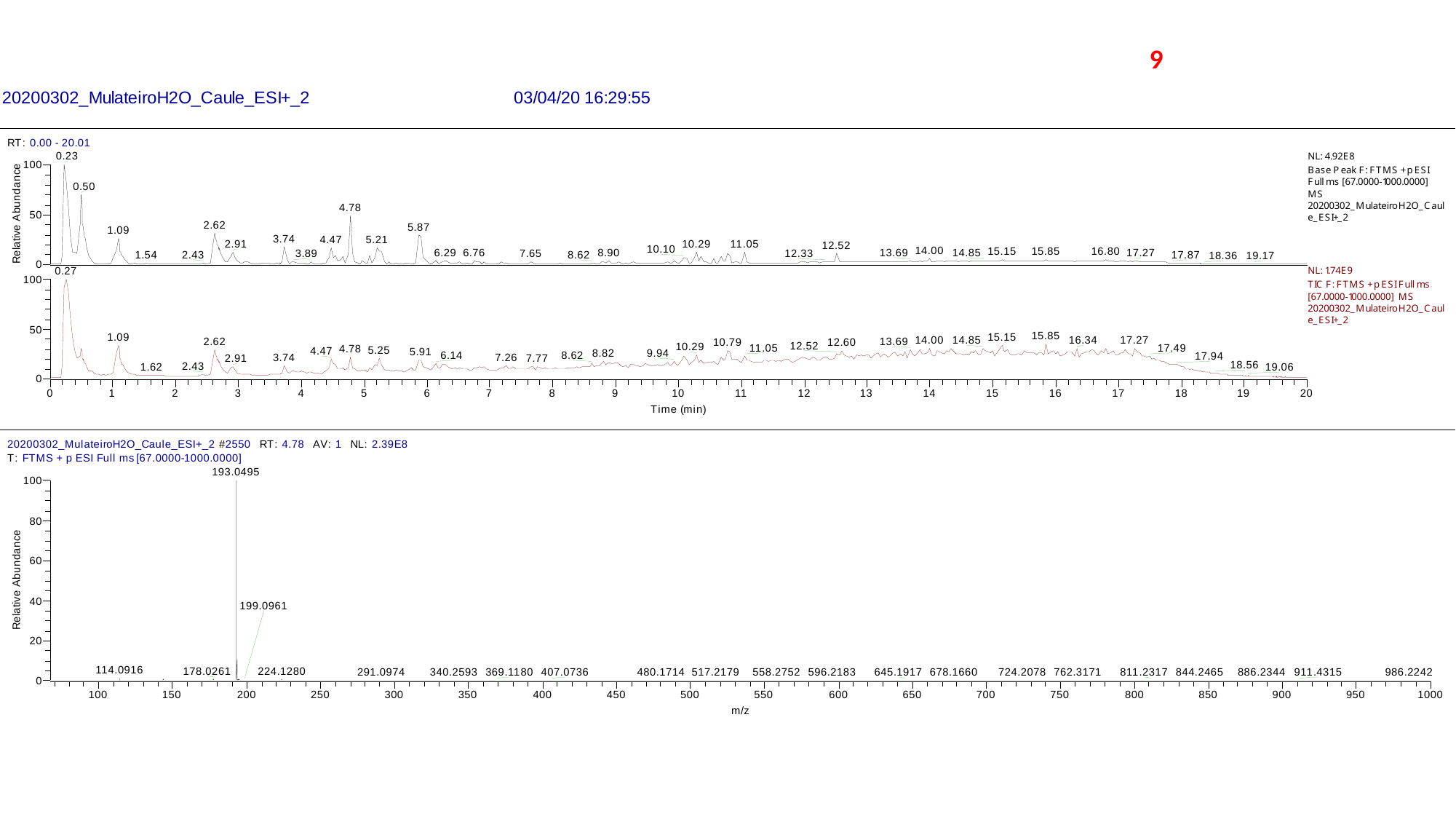

9

## Slide 24
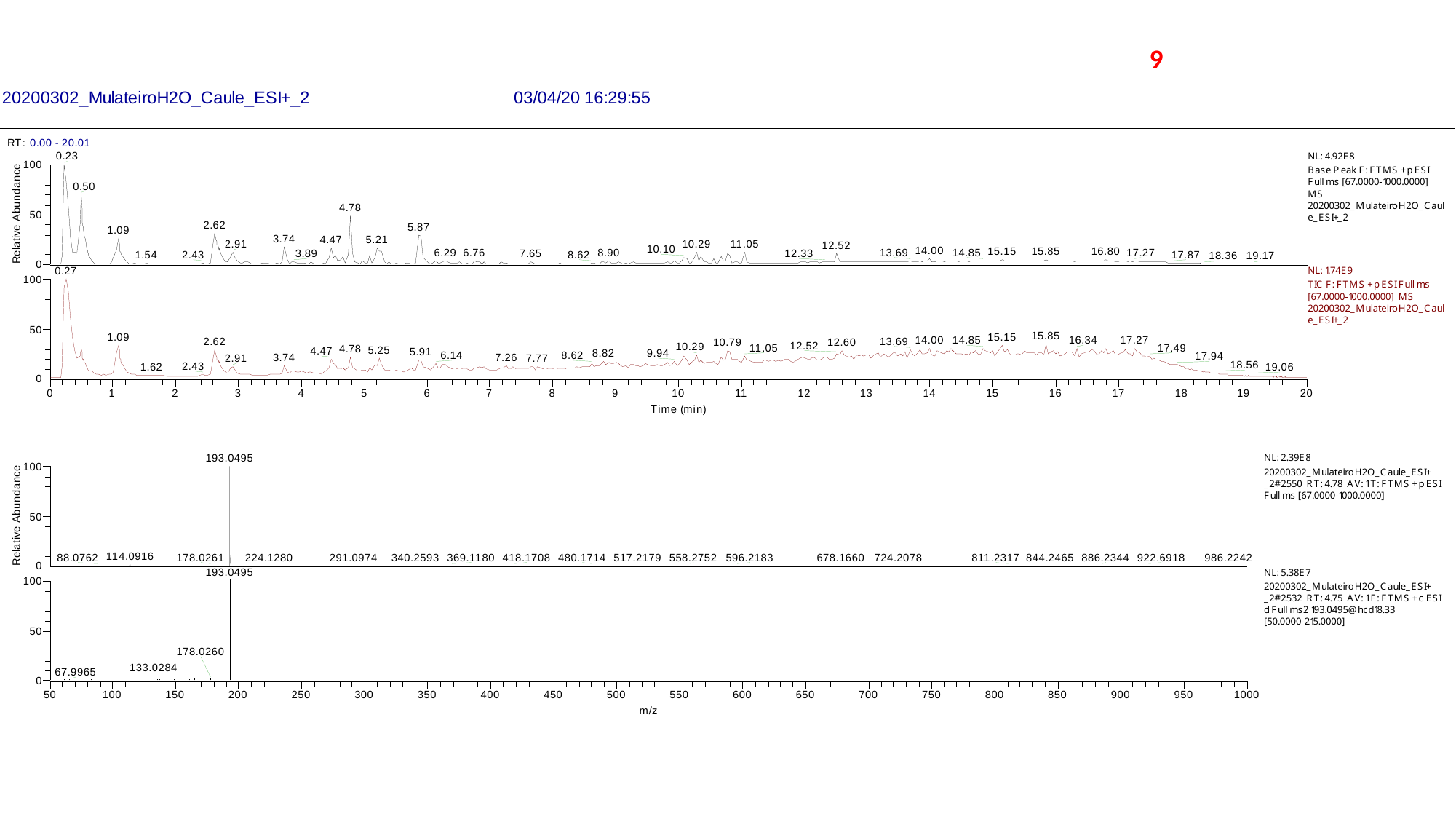

9

## Slide 25
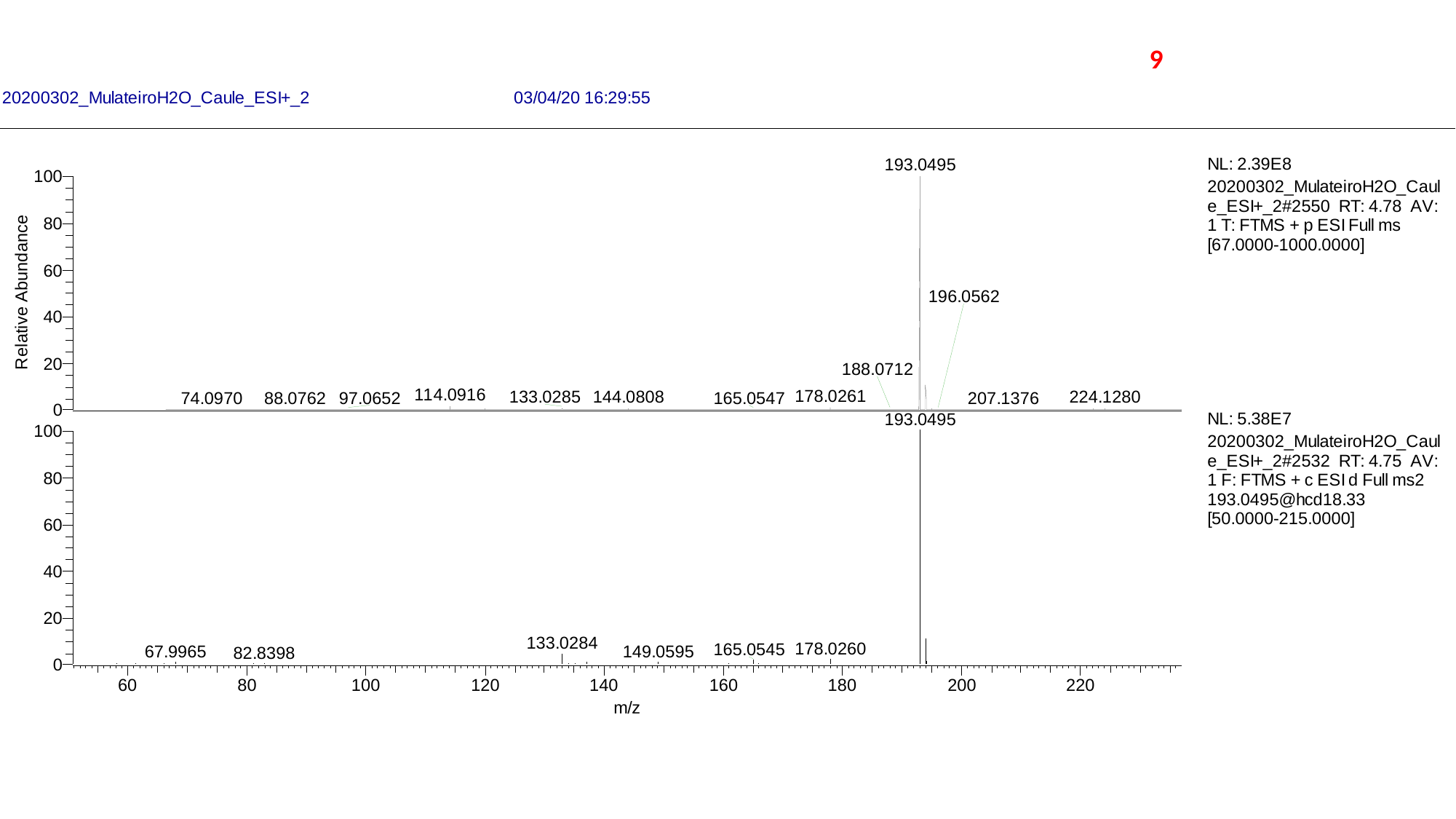

9

## Slide 26
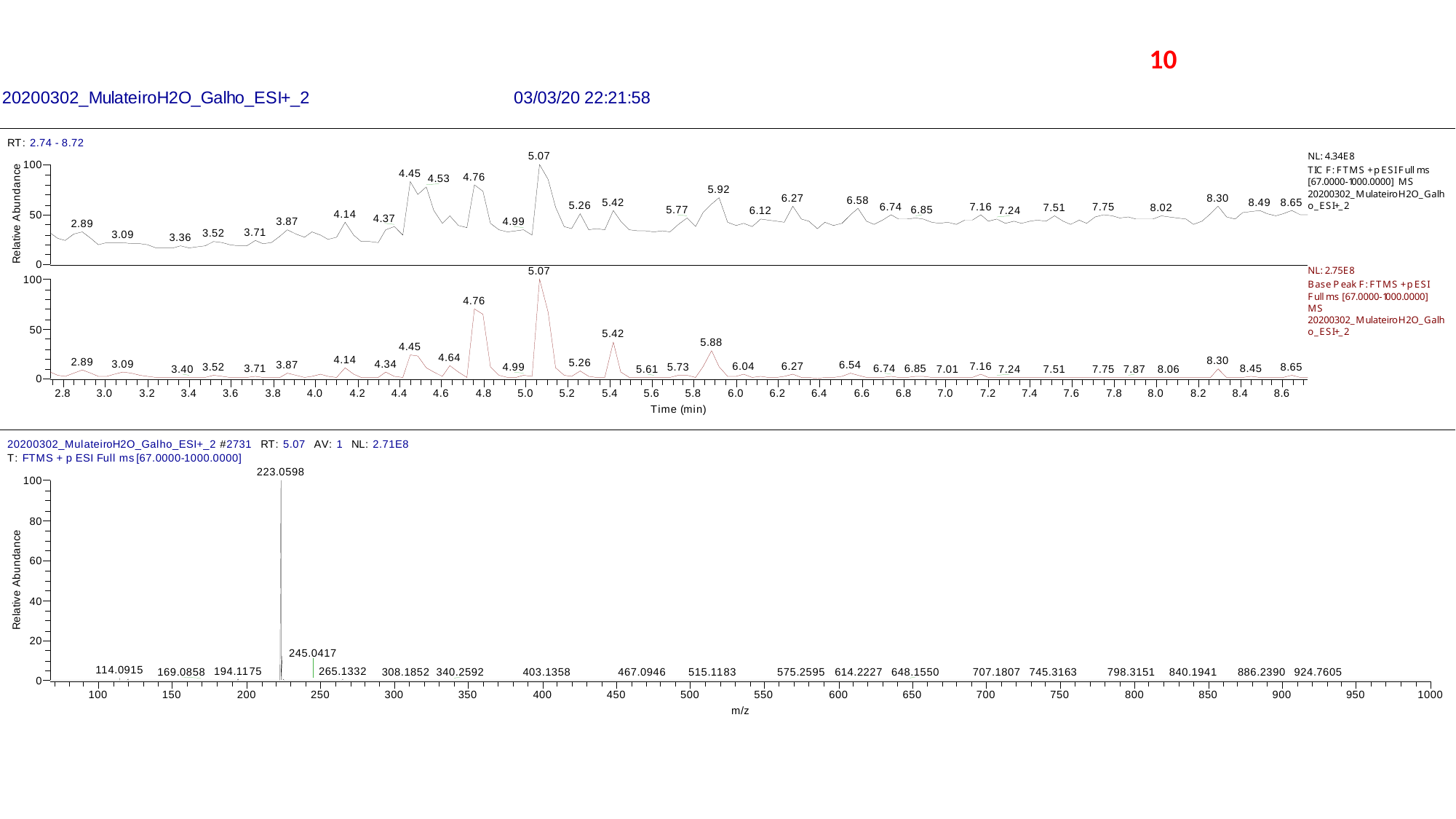

10

## Slide 27
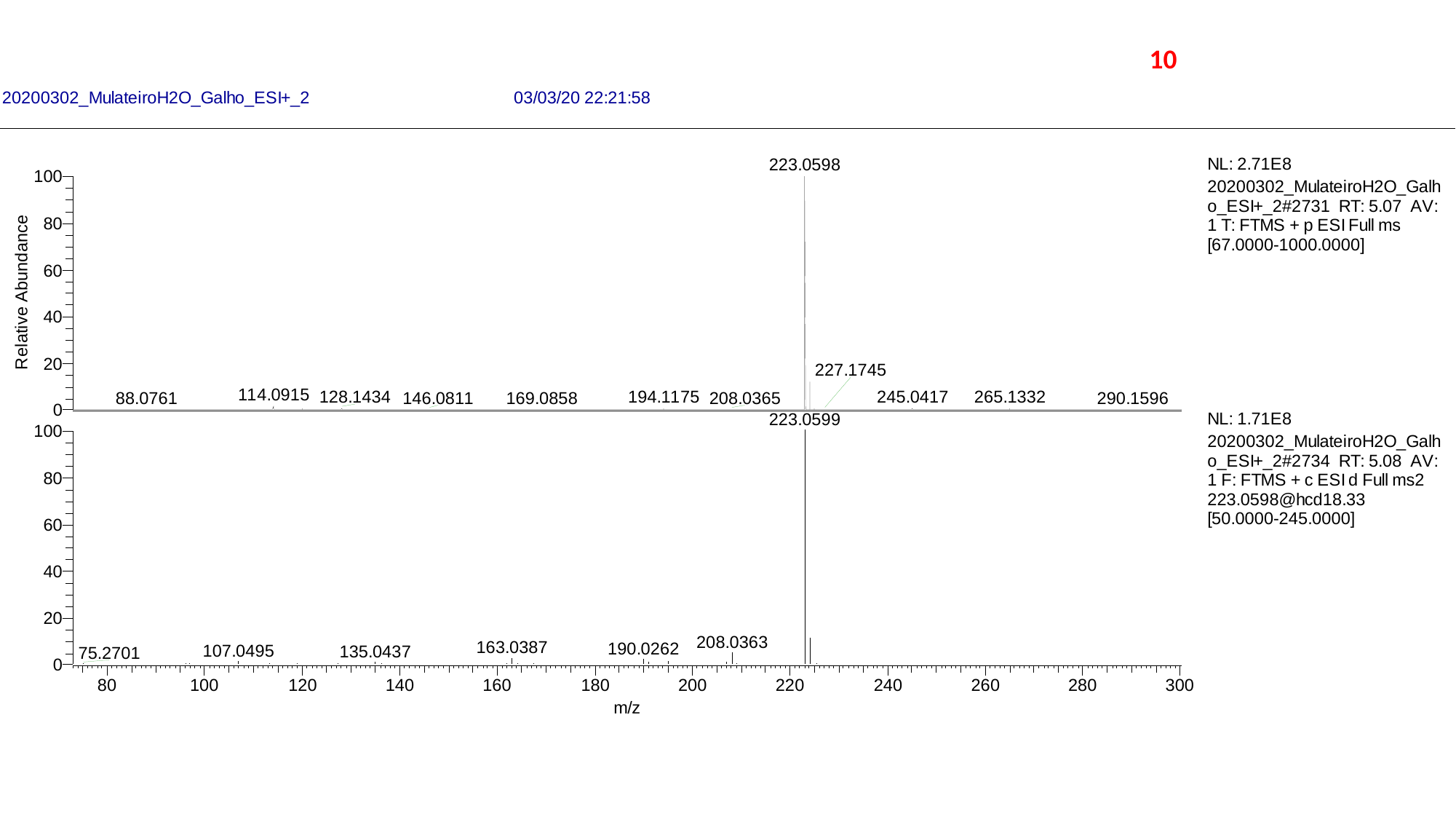

10

## Slide 28
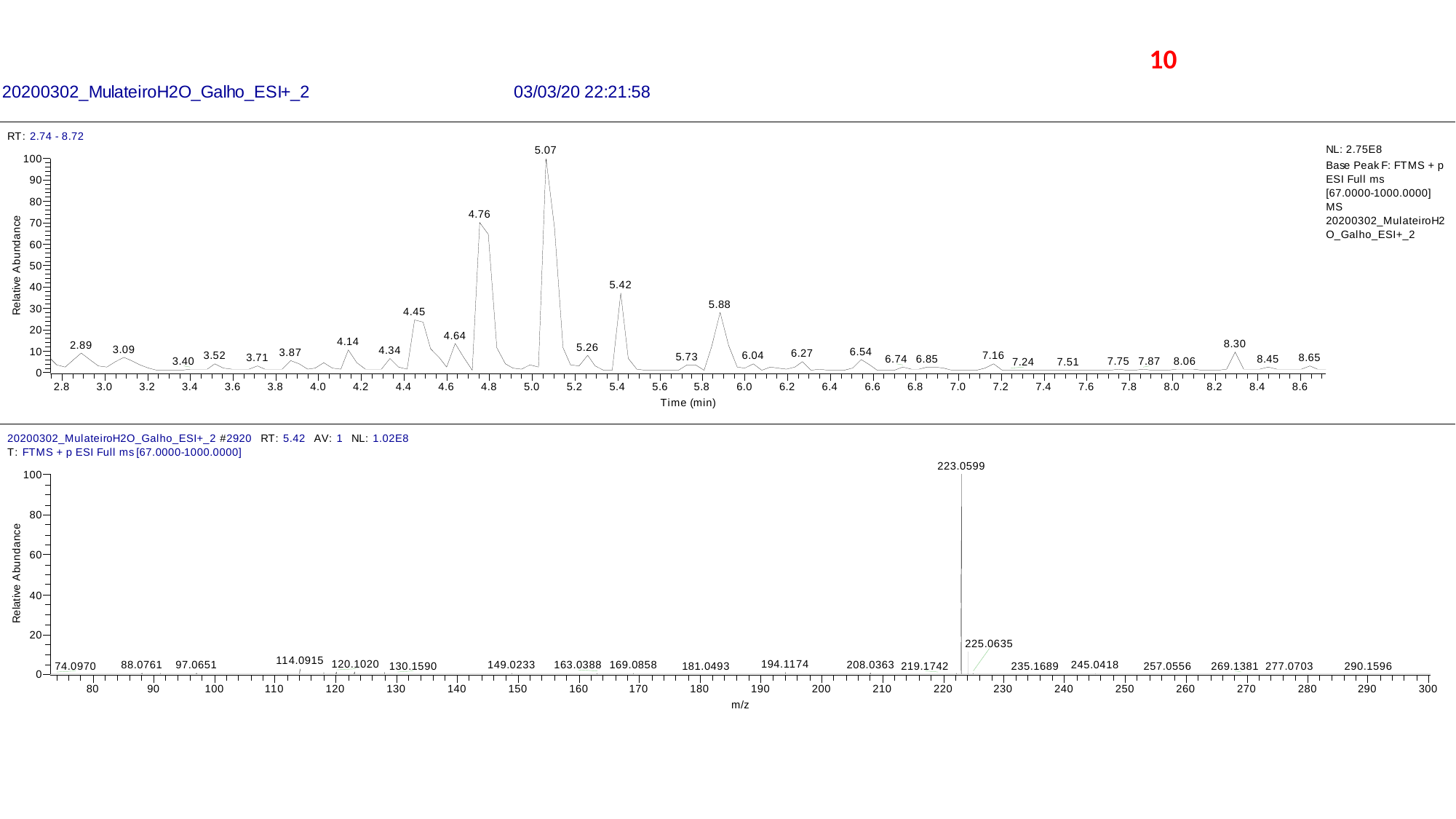

10

## Slide 29
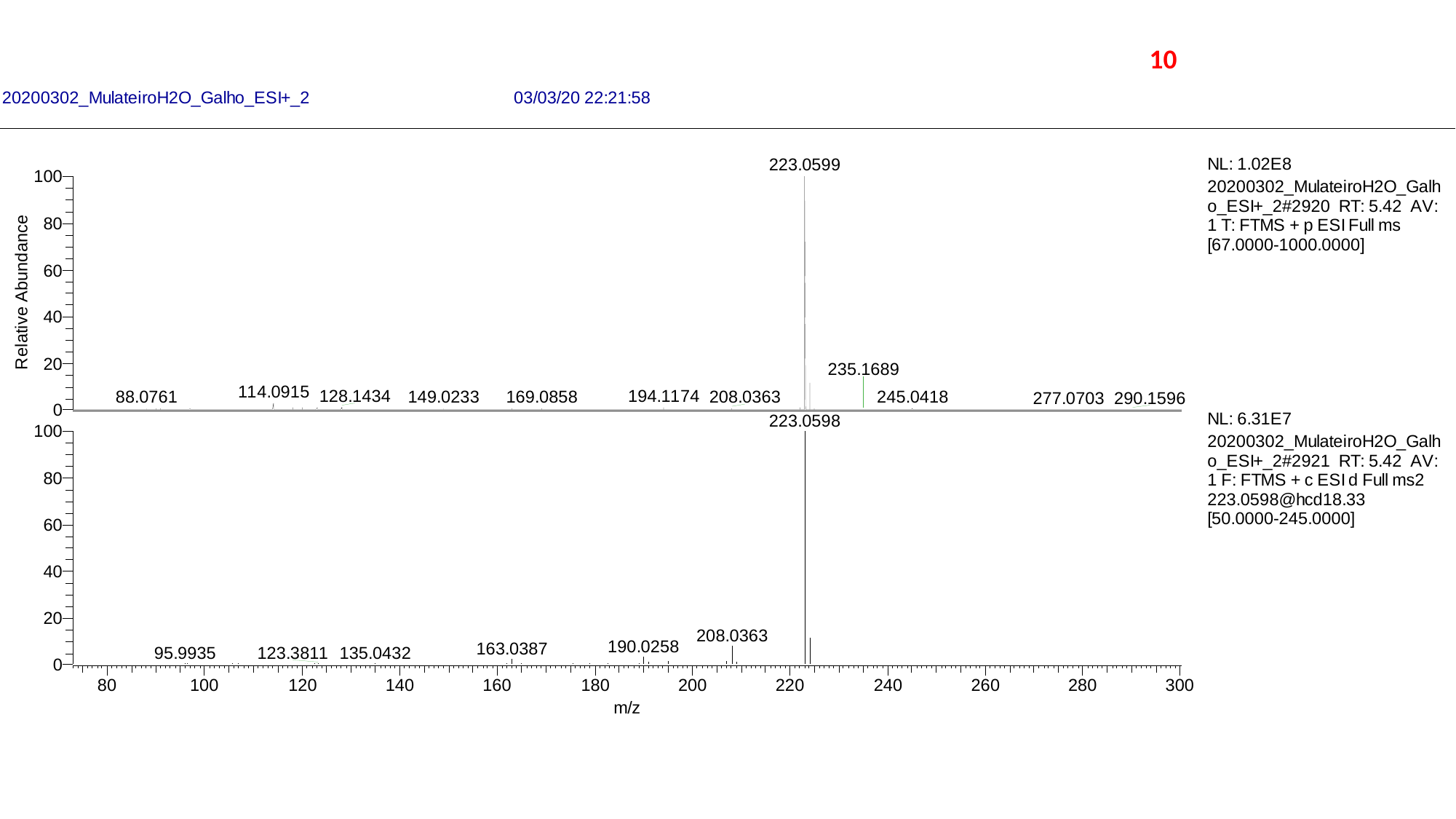

10

## Slide 30
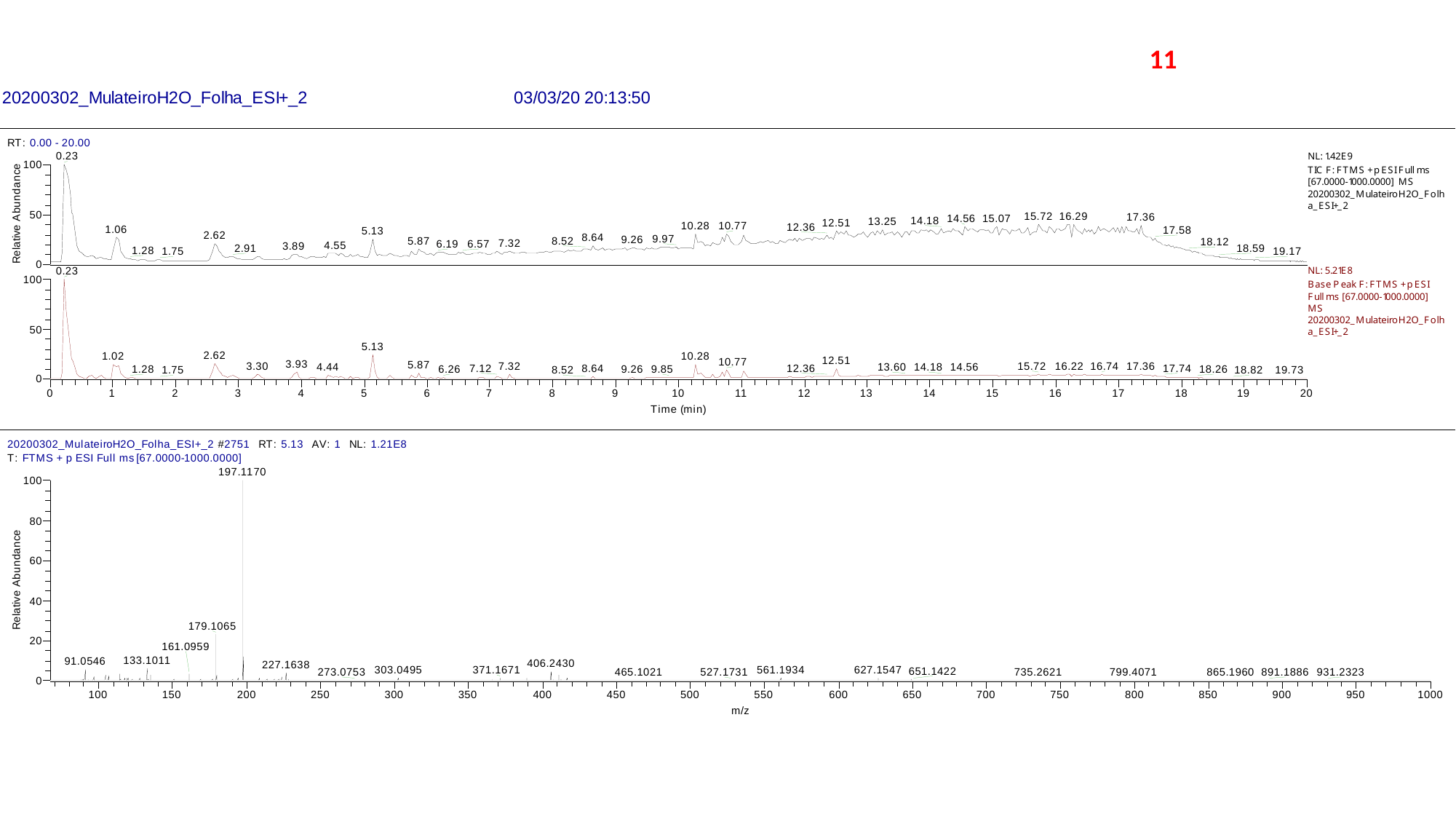

11
 3

## Slide 31
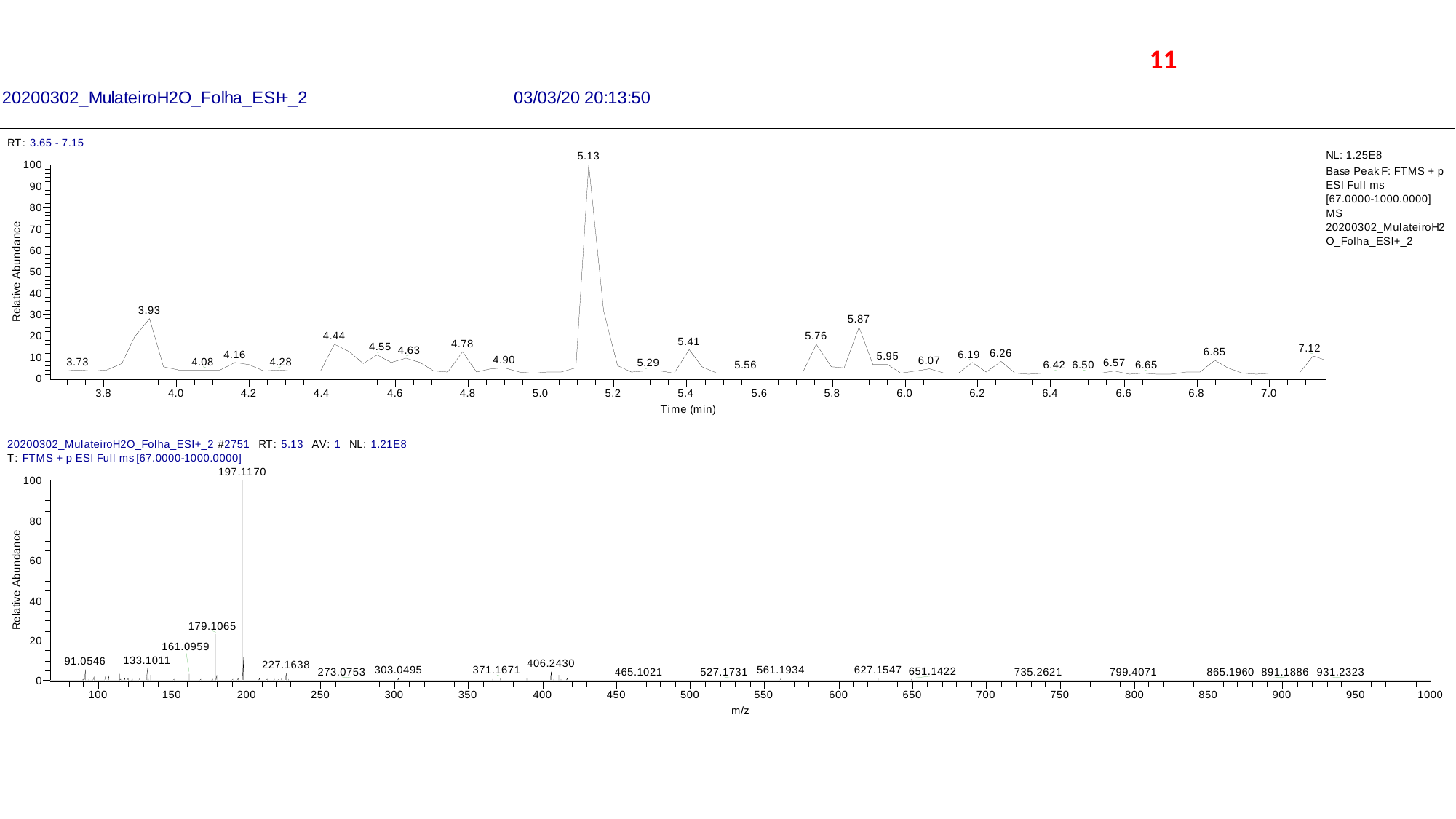

11

## Slide 32
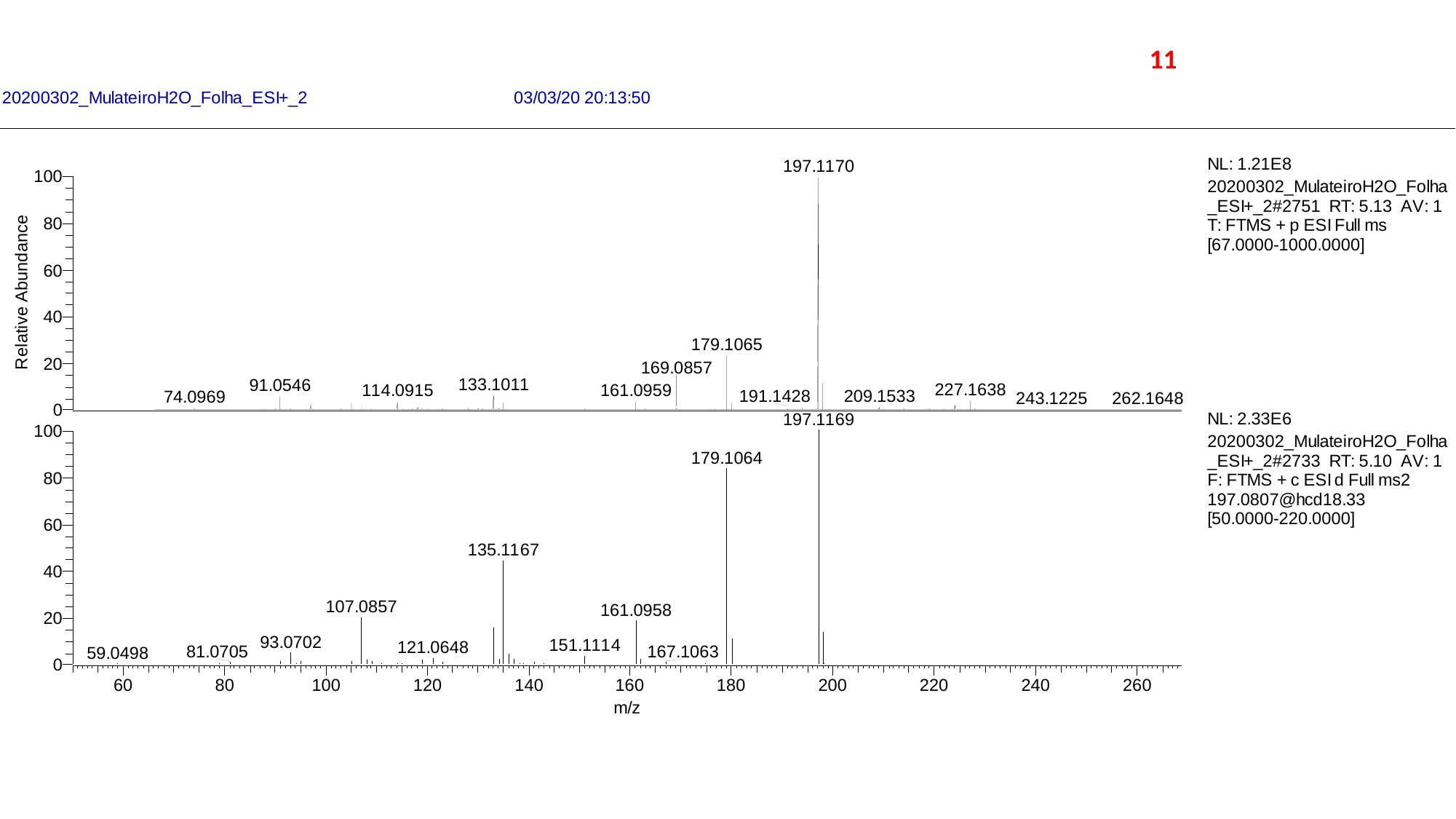

11

## Slide 33
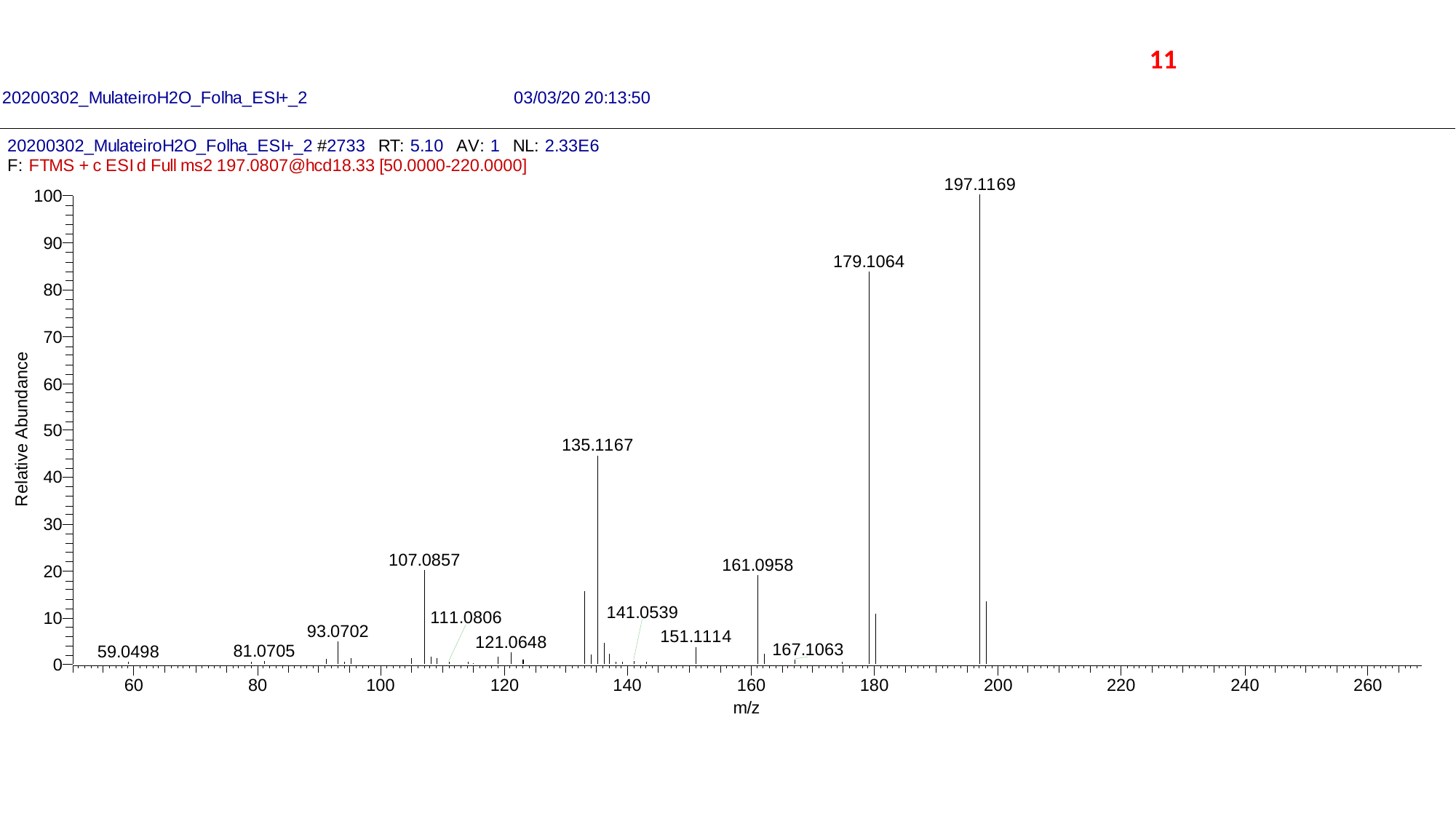

11

## Slide 34
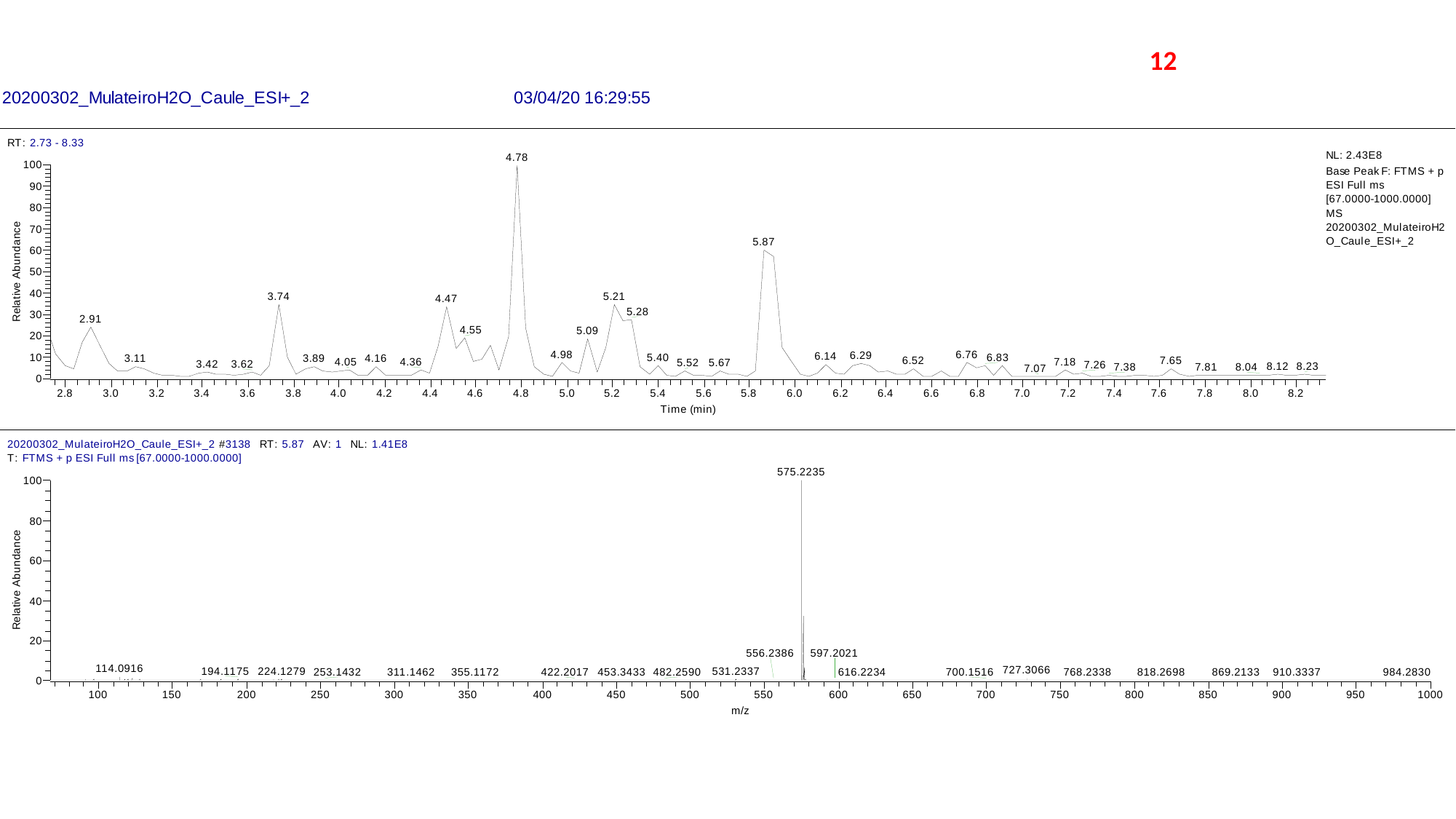

12

## Slide 35
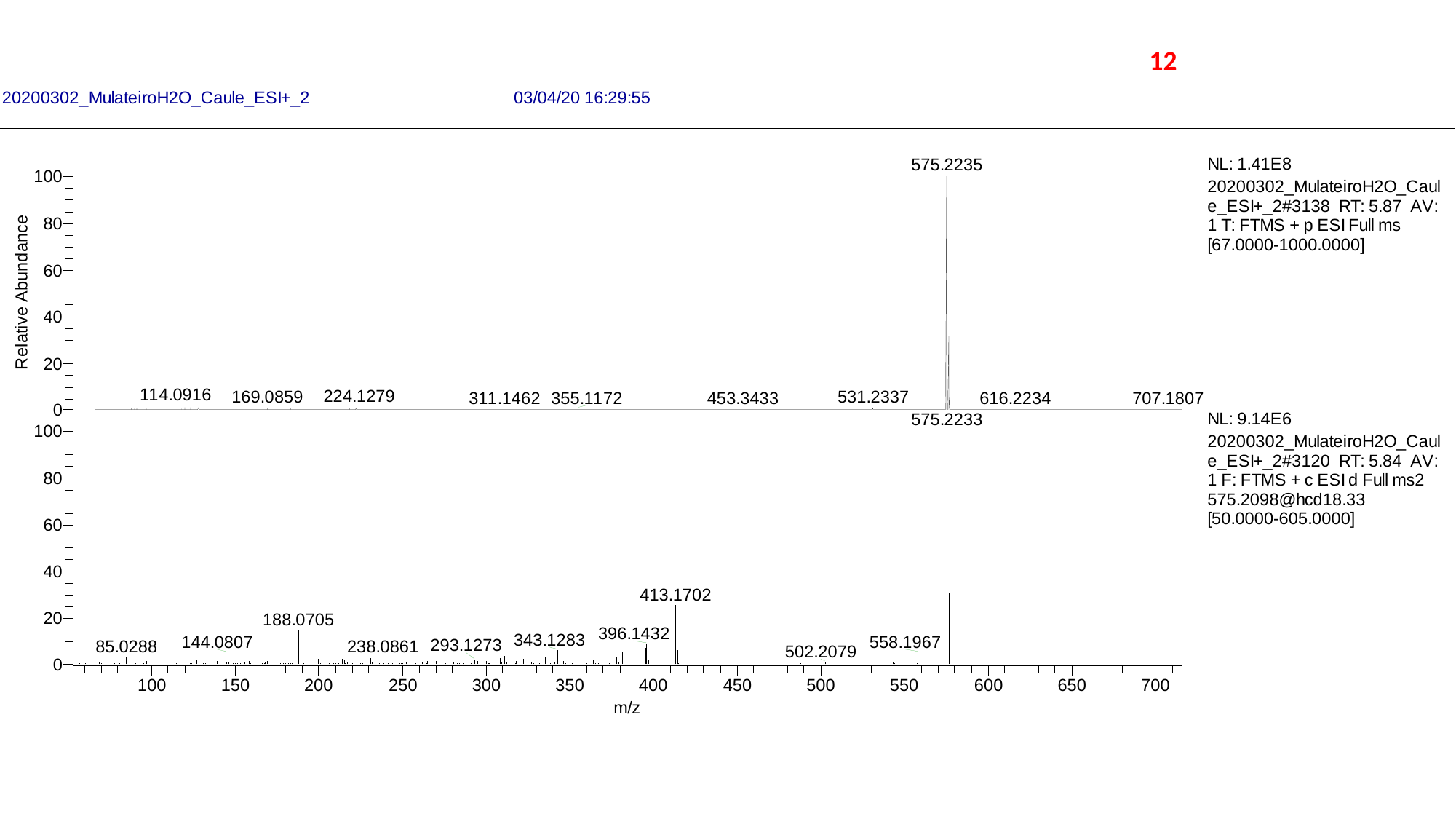

12

## Slide 36
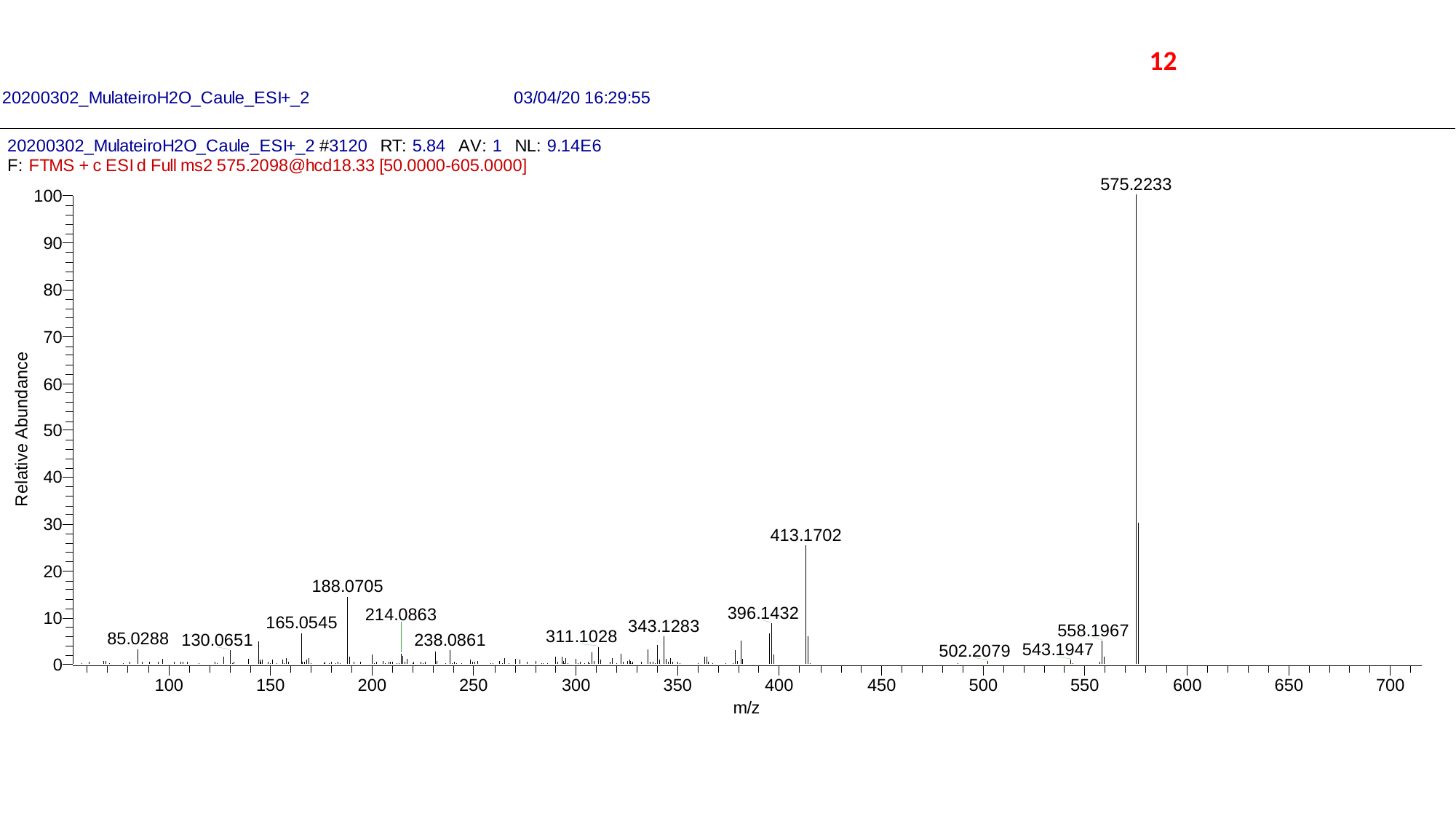

12

## Slide 37
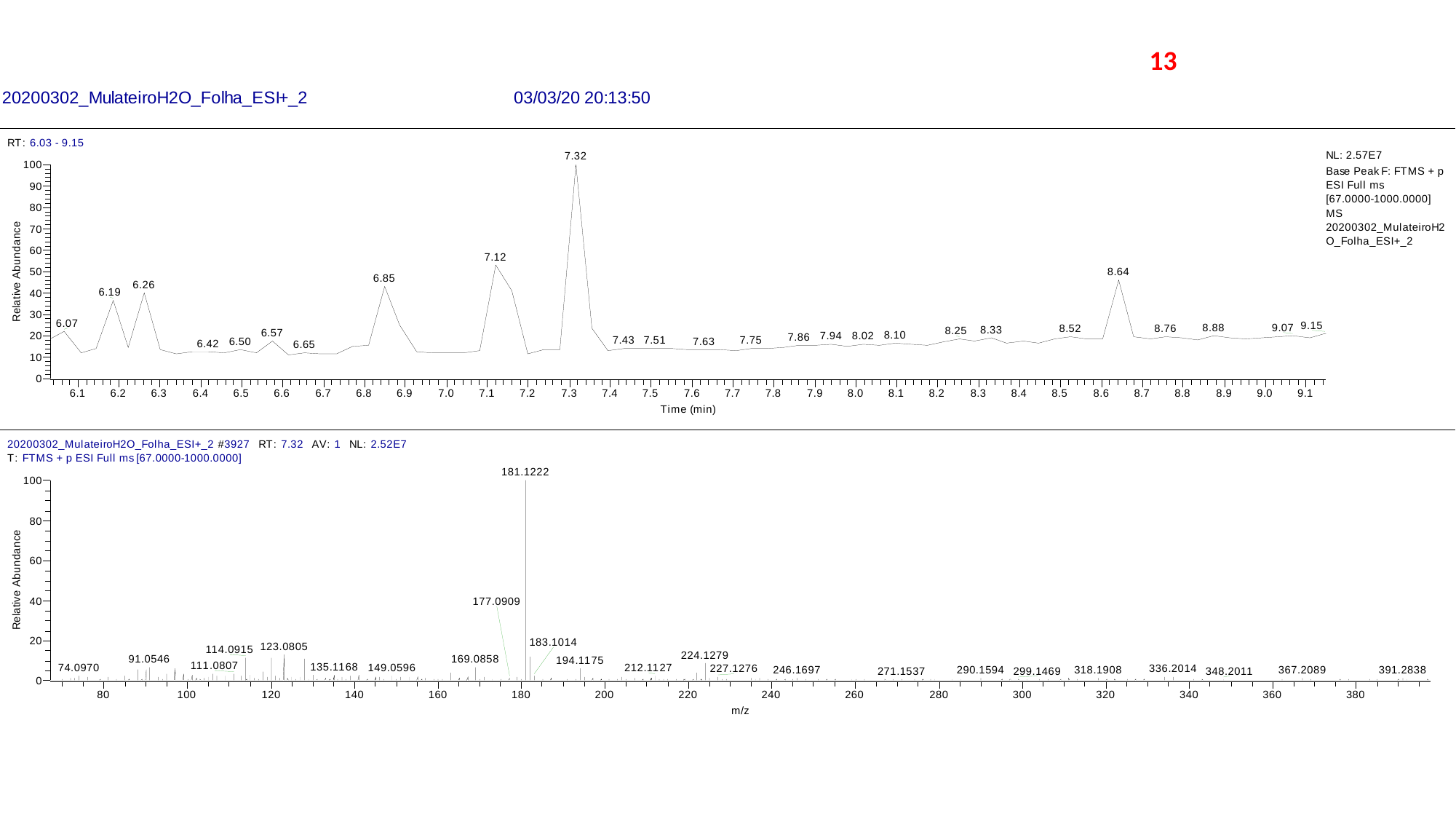

13

## Slide 38
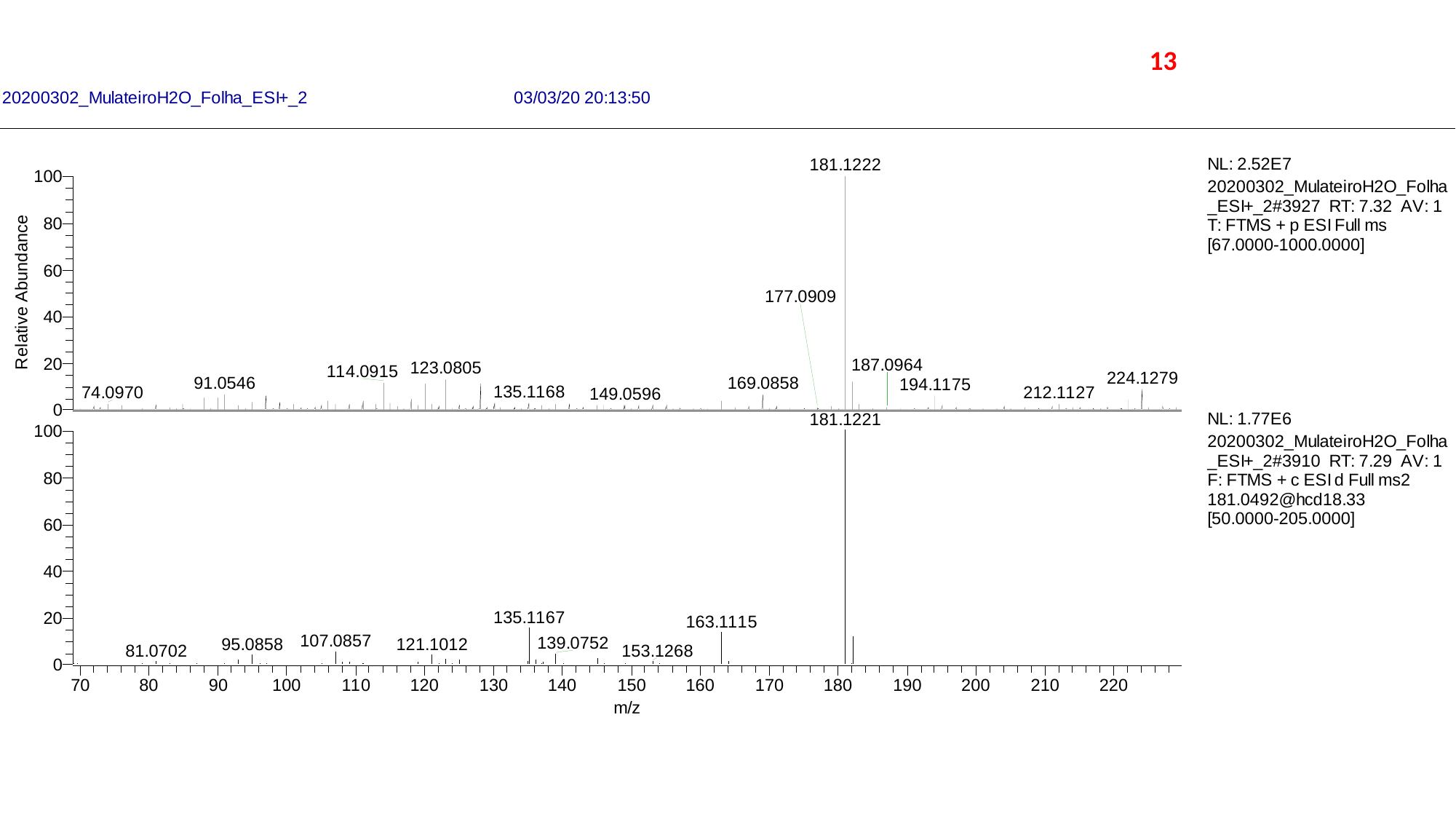

13

## Slide 39
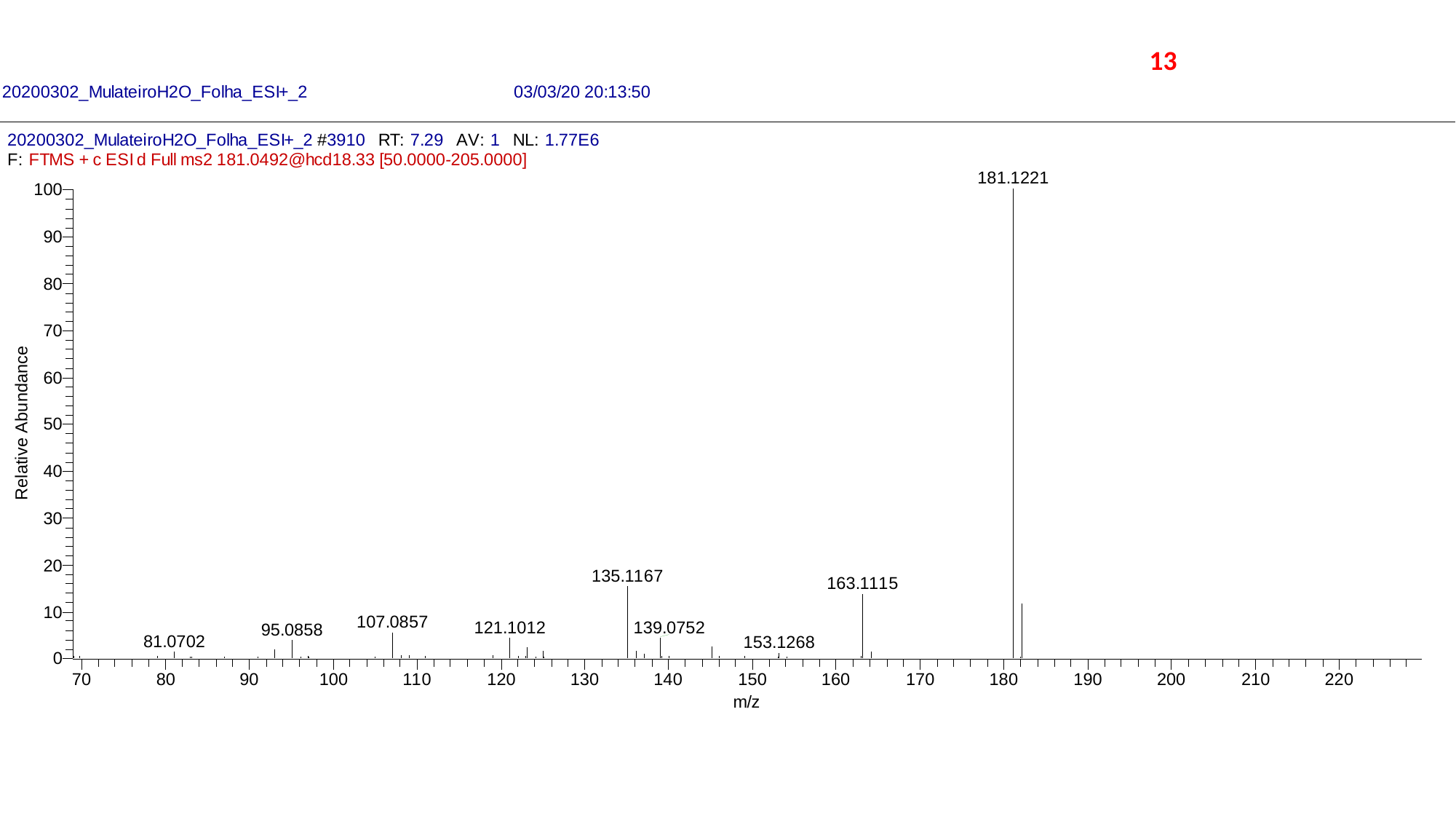

13

## Slide 40
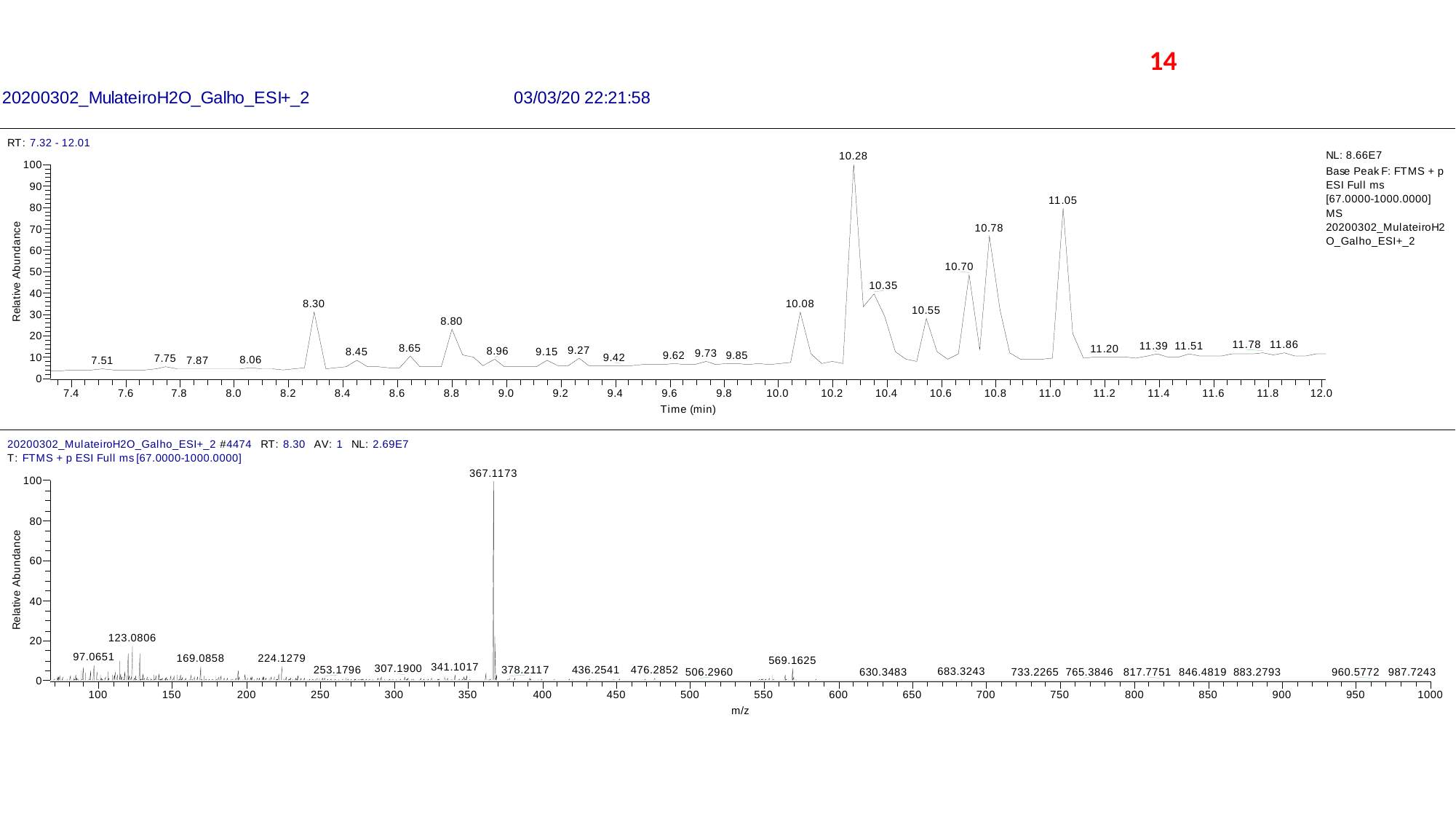

14

## Slide 41
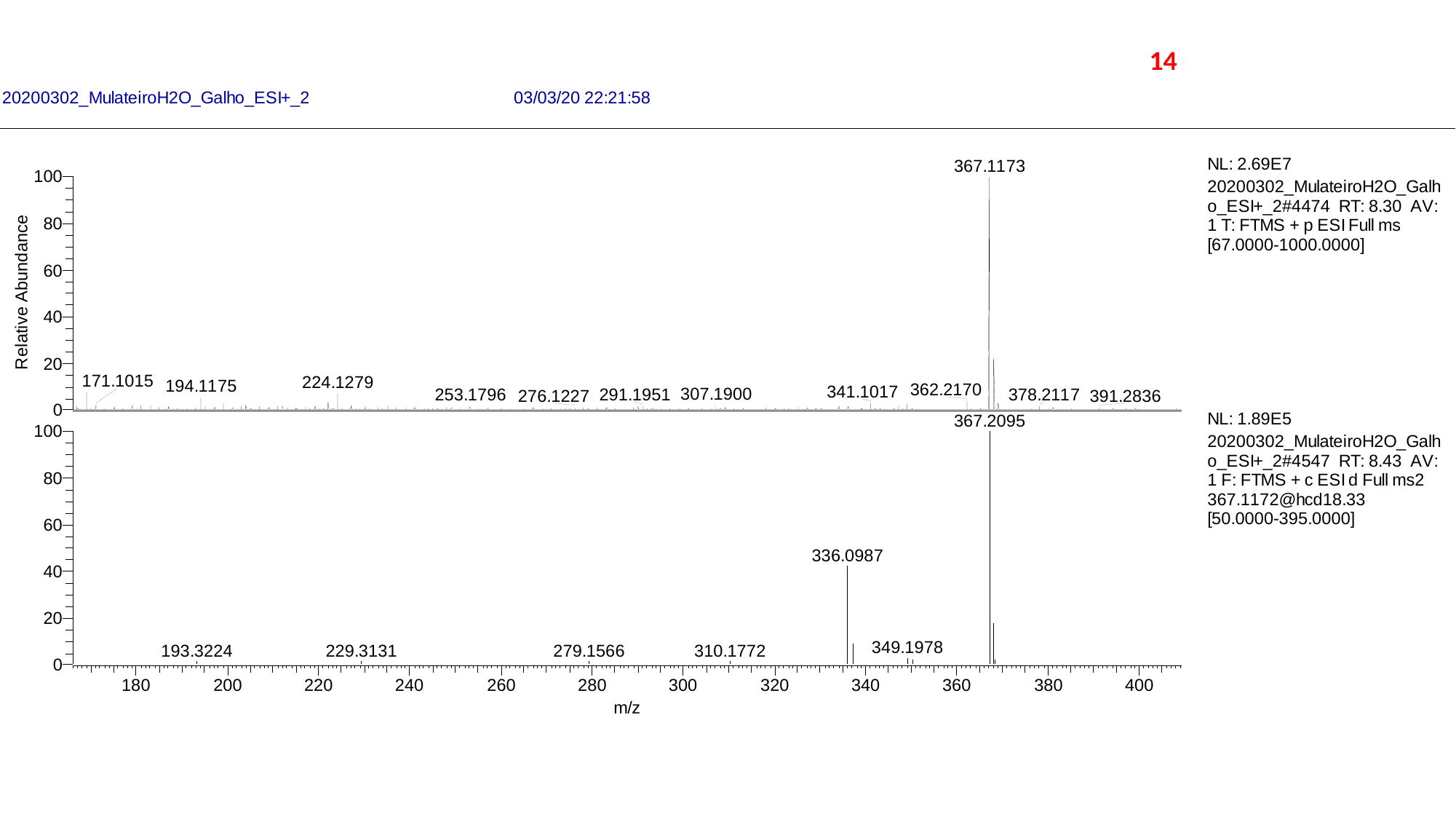

14

## Slide 42
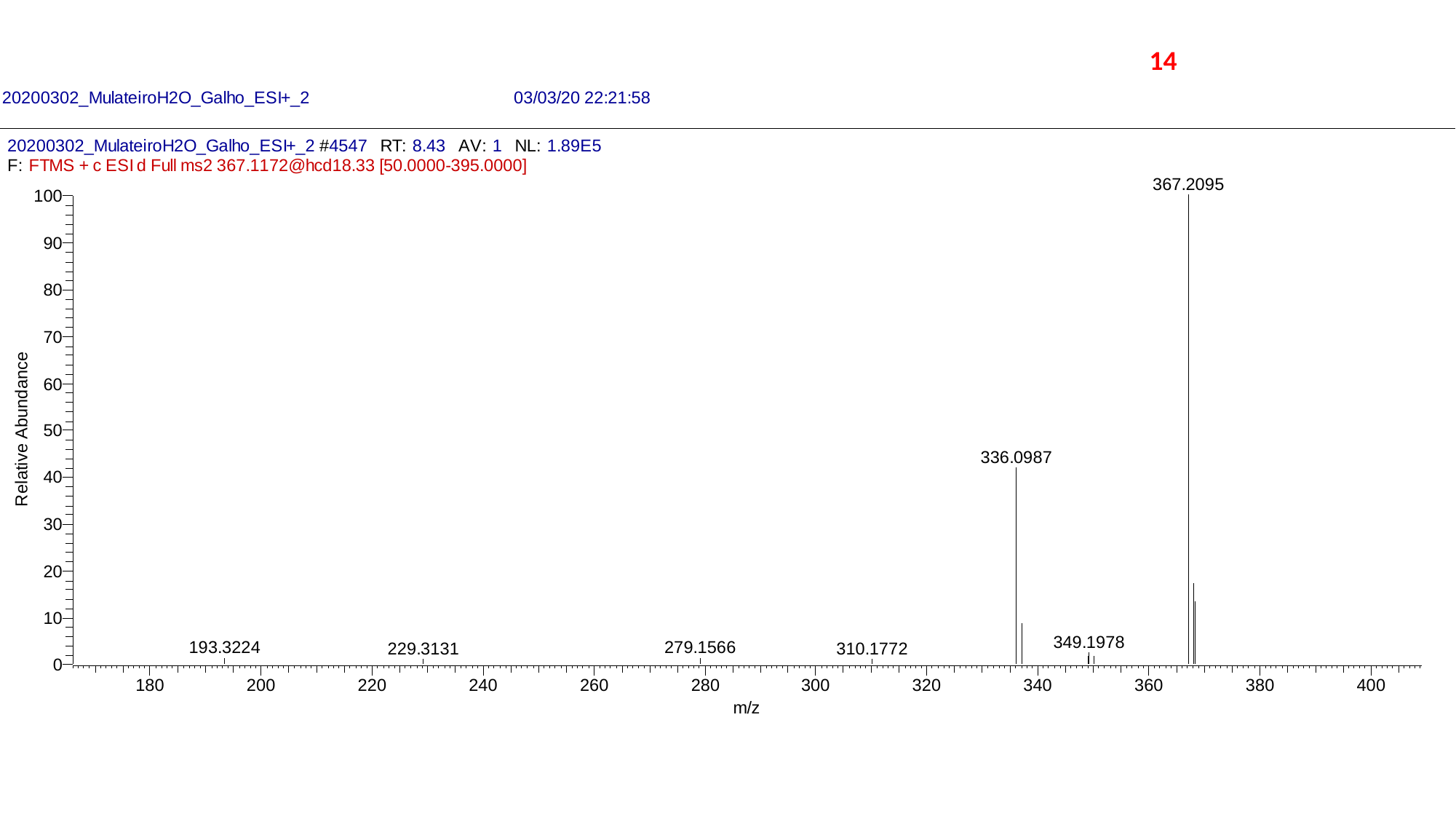

14

## Slide 43
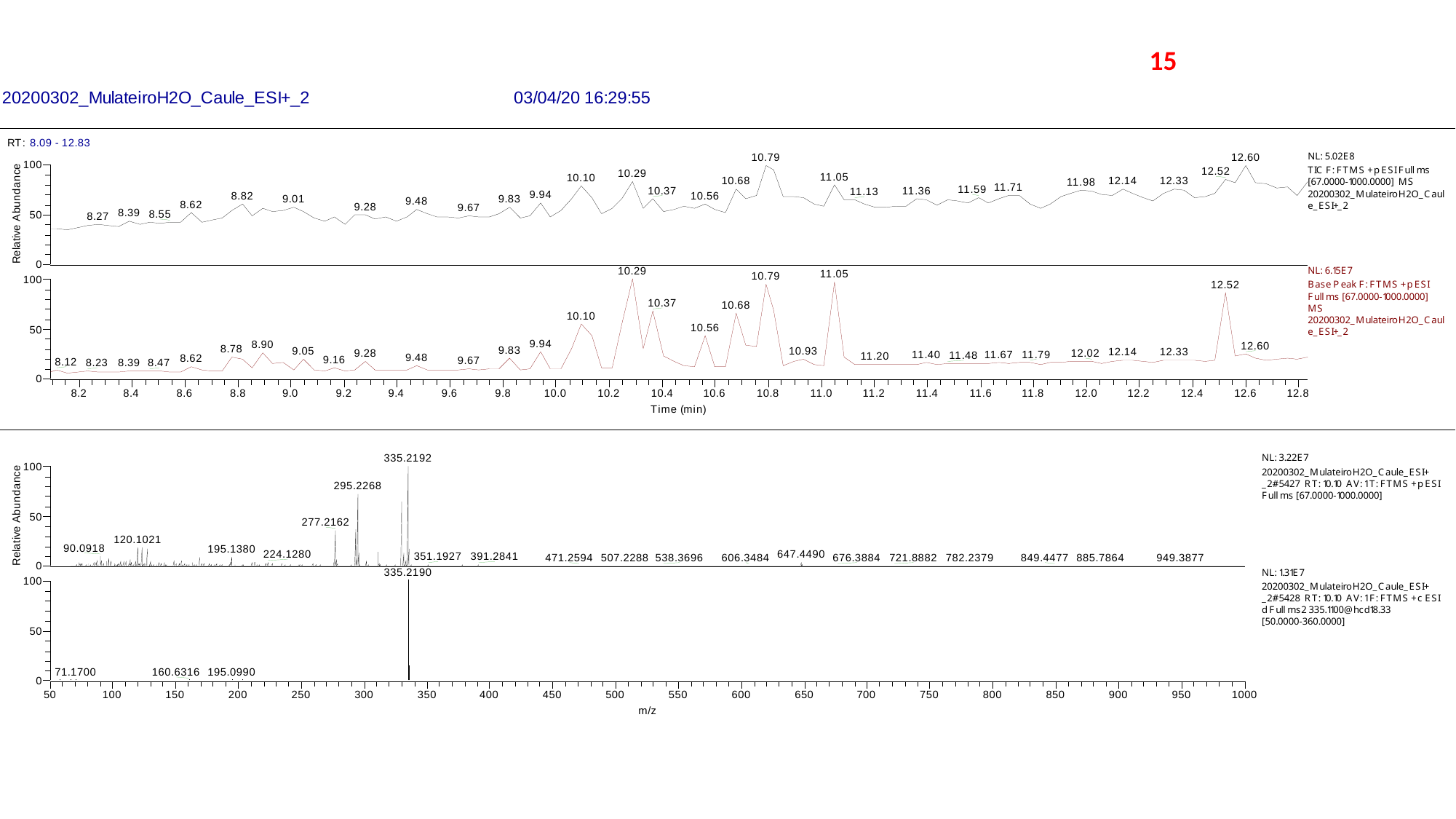

15

## Slide 44
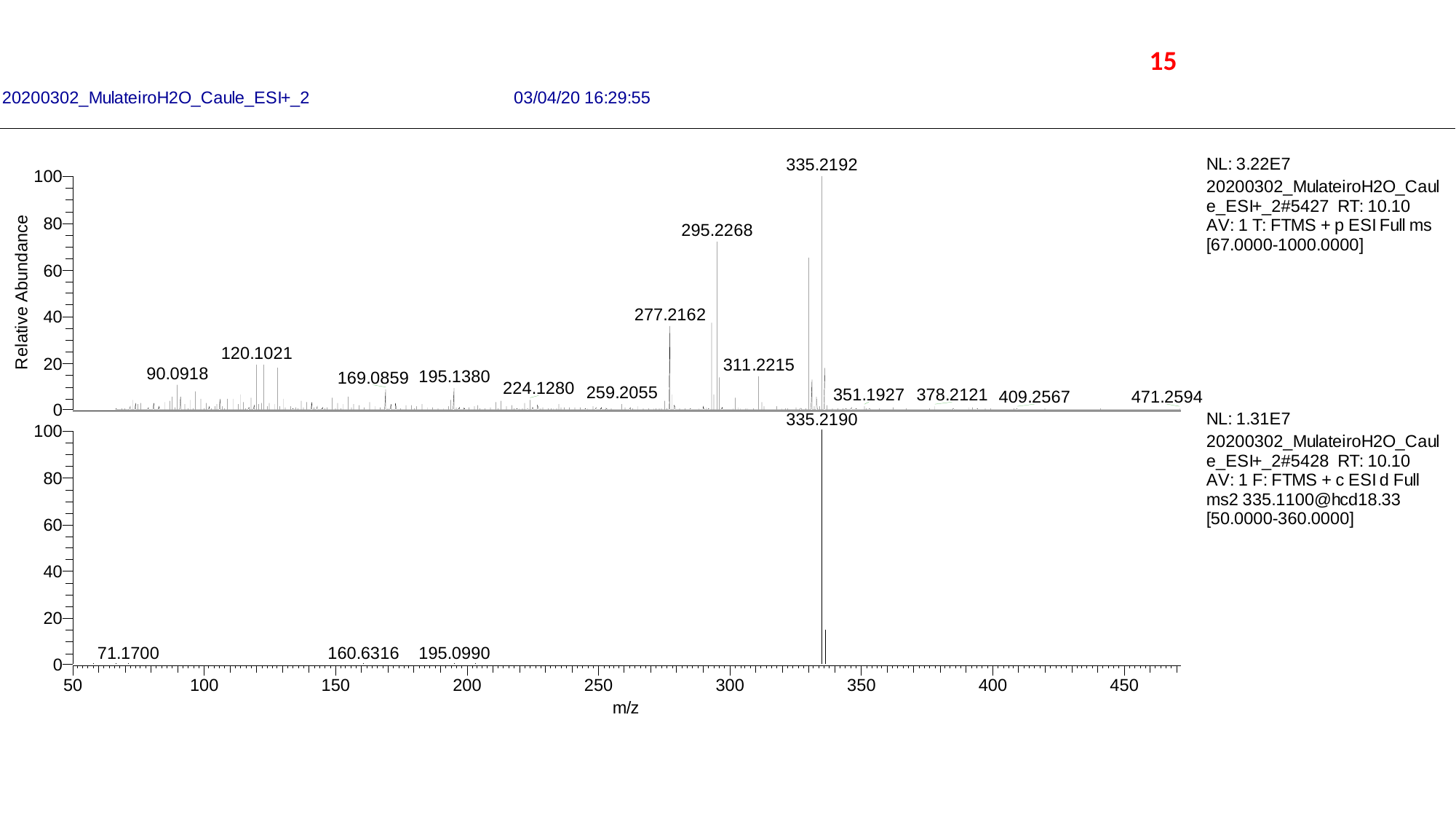

15

## Slide 45
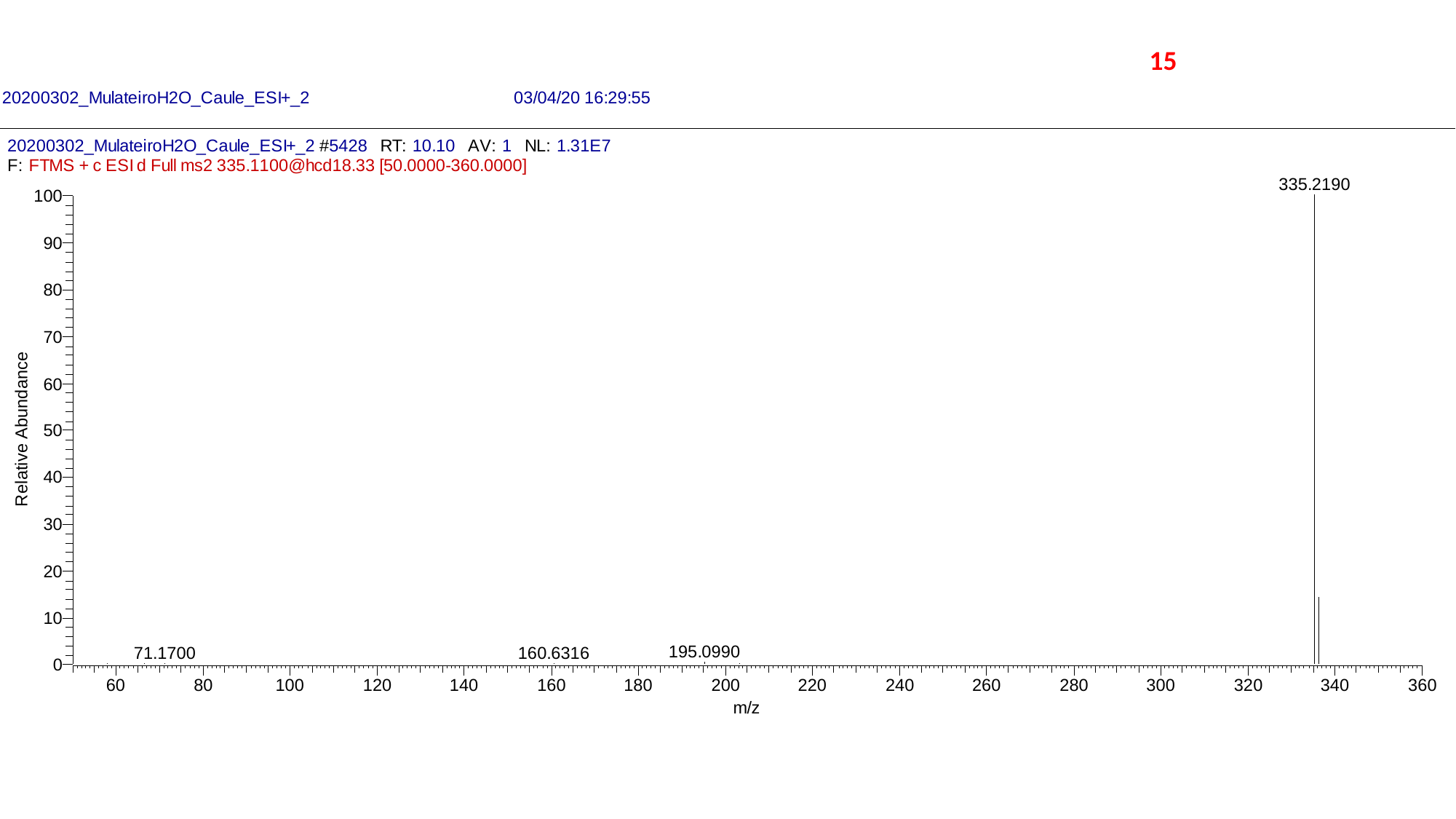

15

## Slide 46
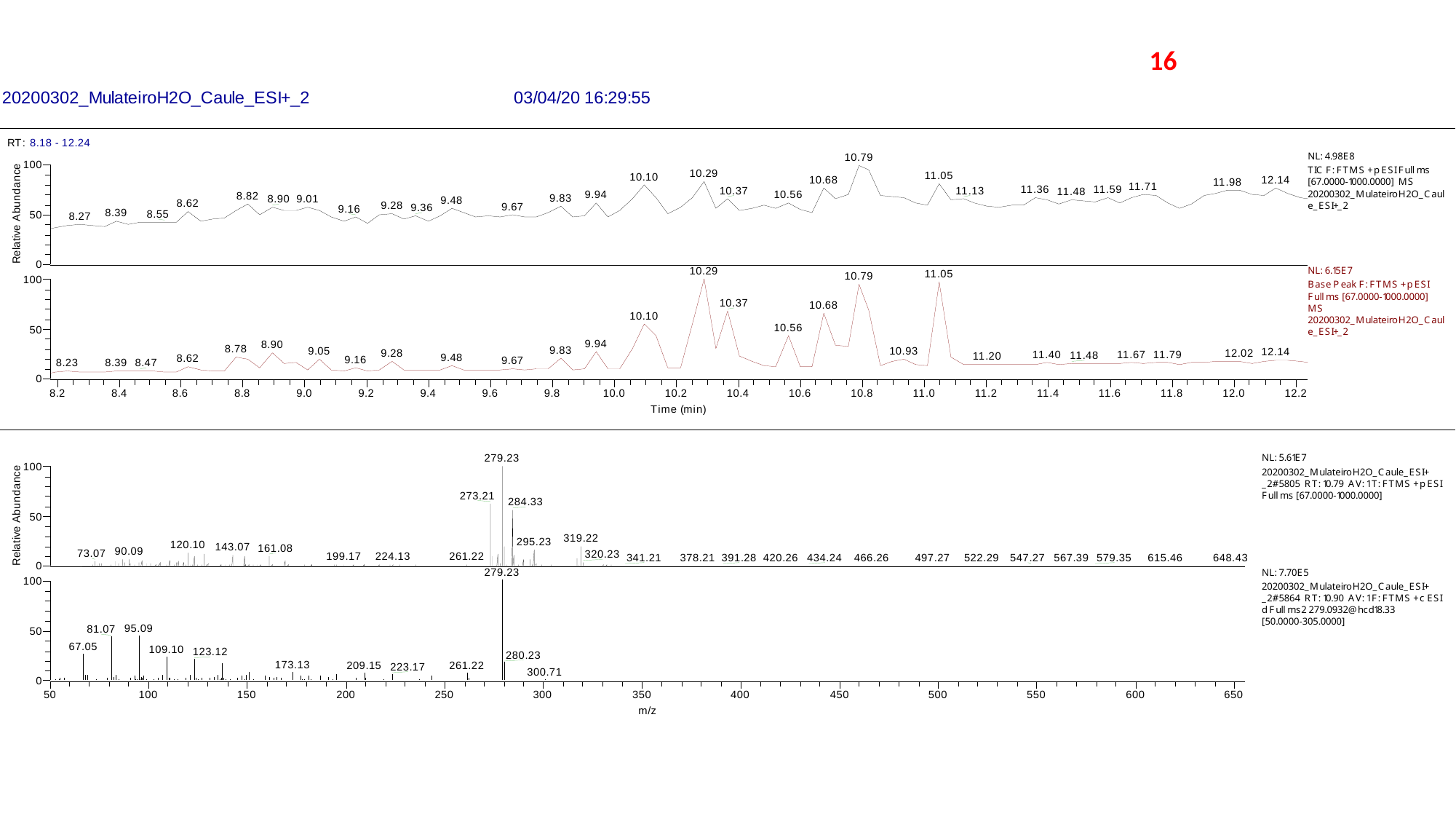

16

## Slide 47
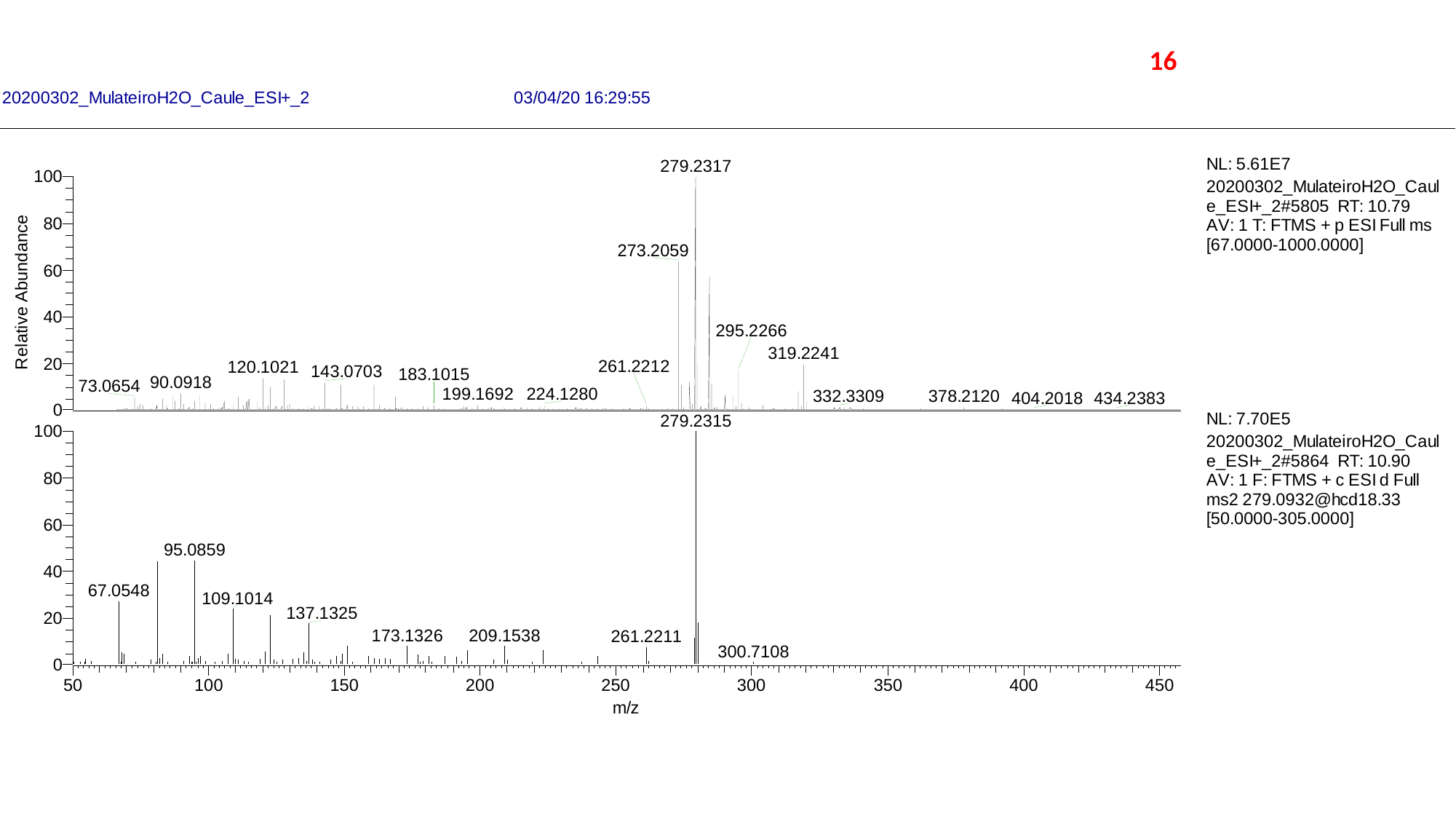

16

## Slide 48
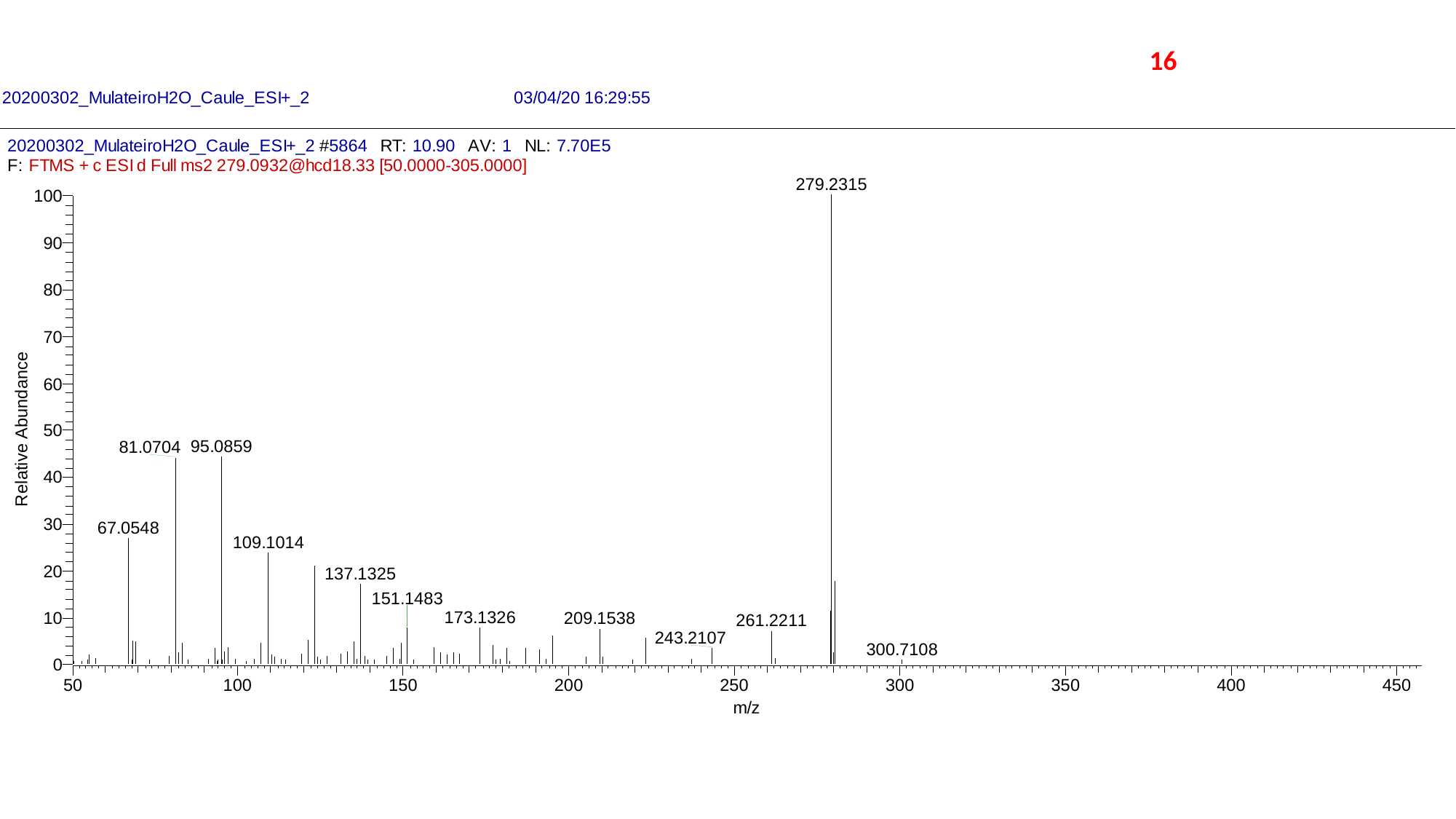

16
