# Supplementary material for: Identification of Bioactive Metabolites of Capirona macrophylla by Metabolomic Analysis, Molecular Docking, and In Vitro Antiparasitic Assays
Source: Metabolites. 2025 Feb 26;15(3):157. doi: 10.3390/metabo15030157 (PMC11943490; doi:10.3390/metabo15030157)
Supplement: Supplementary file 1 [file metabolites-15-00157-s001.zip › Supplementary material S2.pptx]

## Slide 1
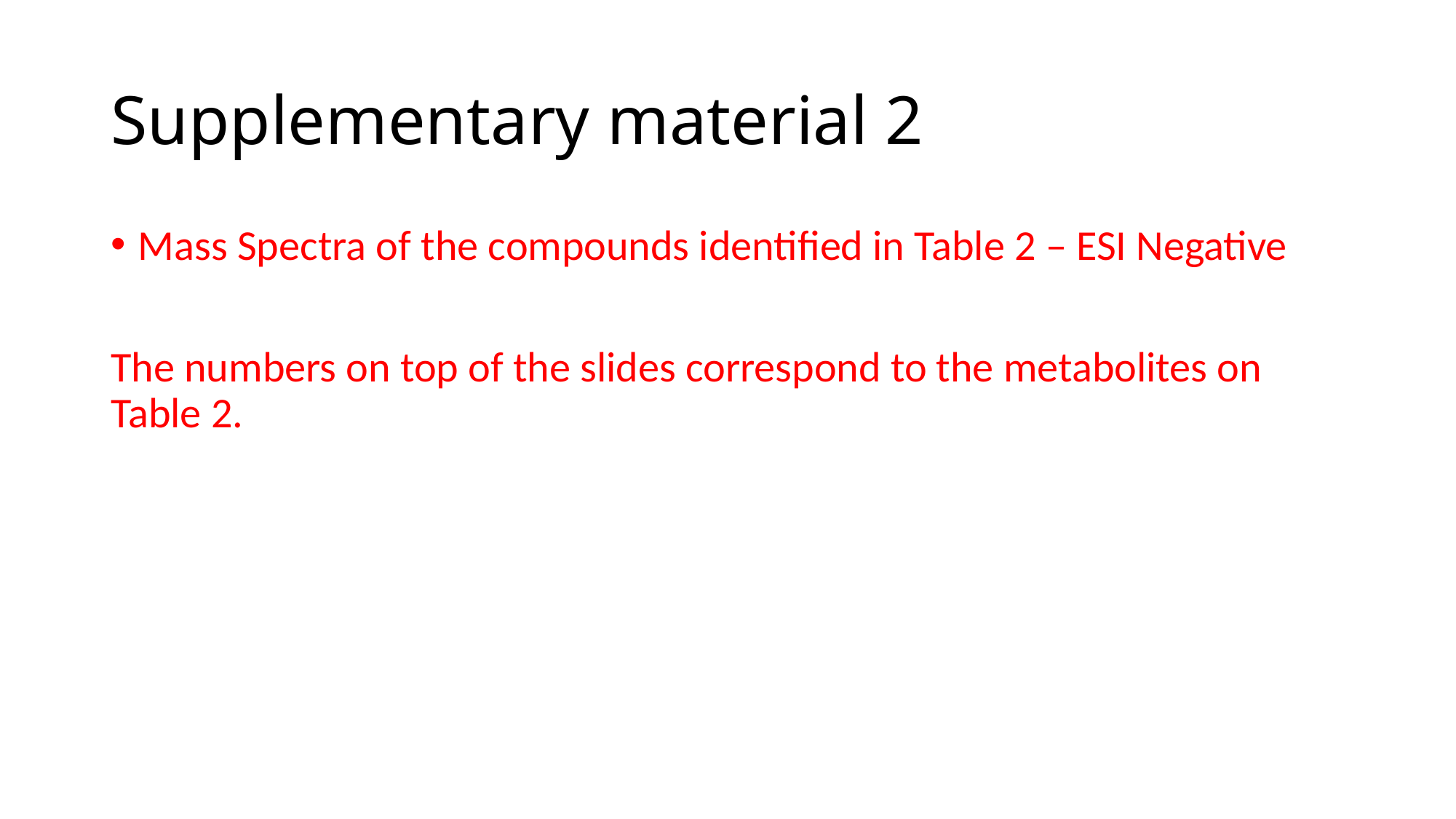

# Supplementary material 2
Mass Spectra of the compounds identified in Table 2 – ESI Negative
The numbers on top of the slides correspond to the metabolites on Table 2.

## Slide 2
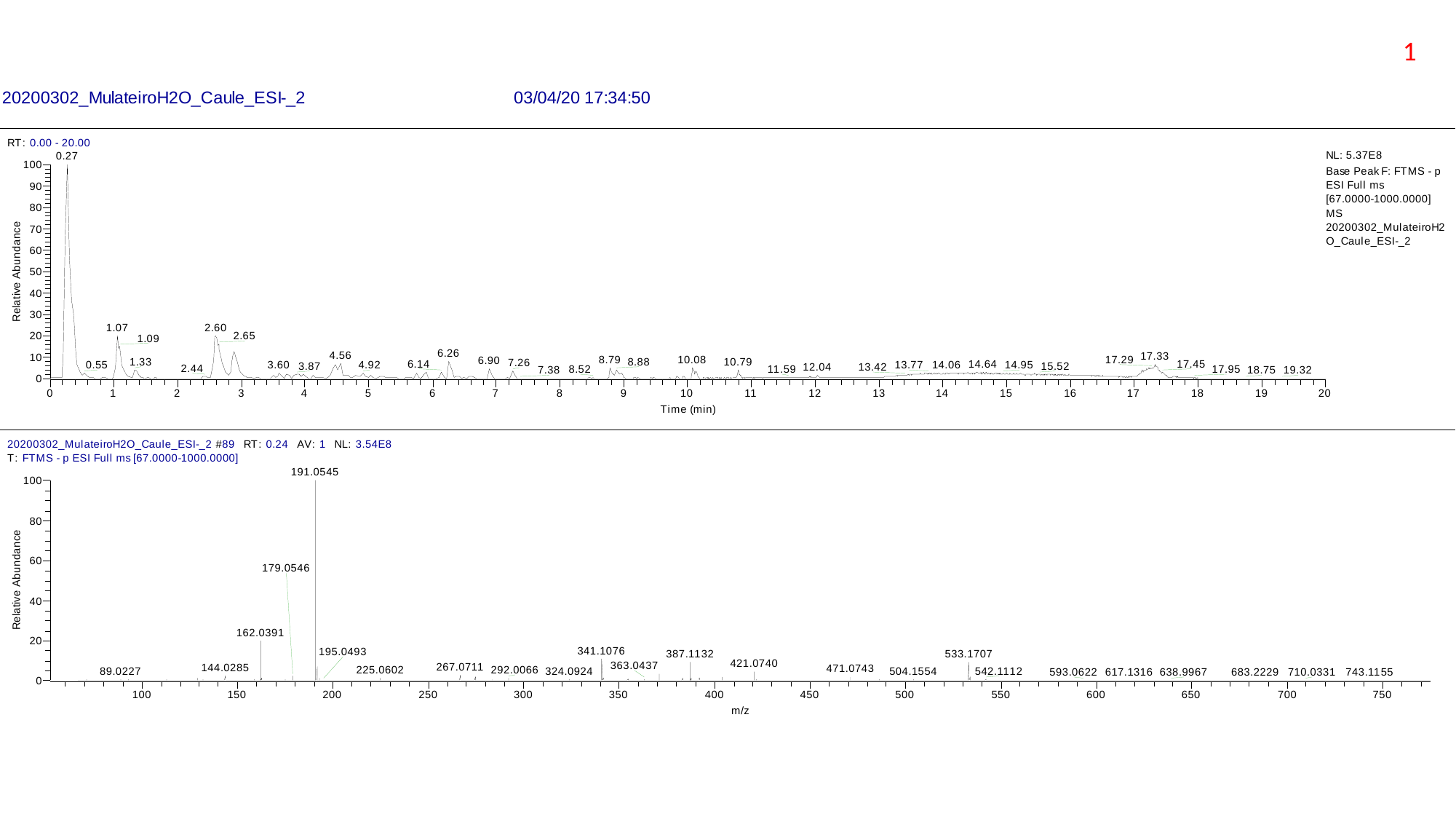

1

## Slide 3
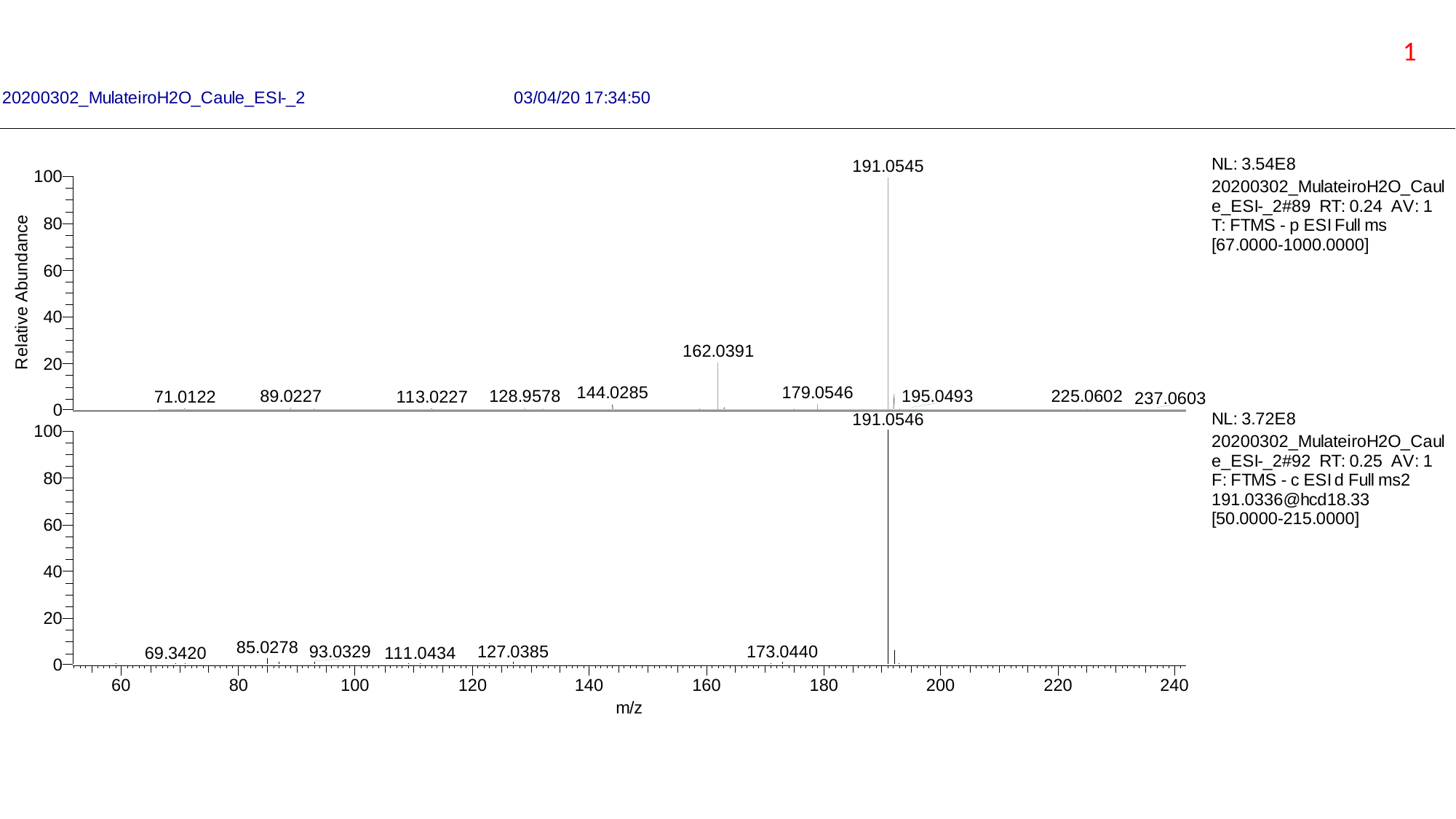

1

## Slide 4
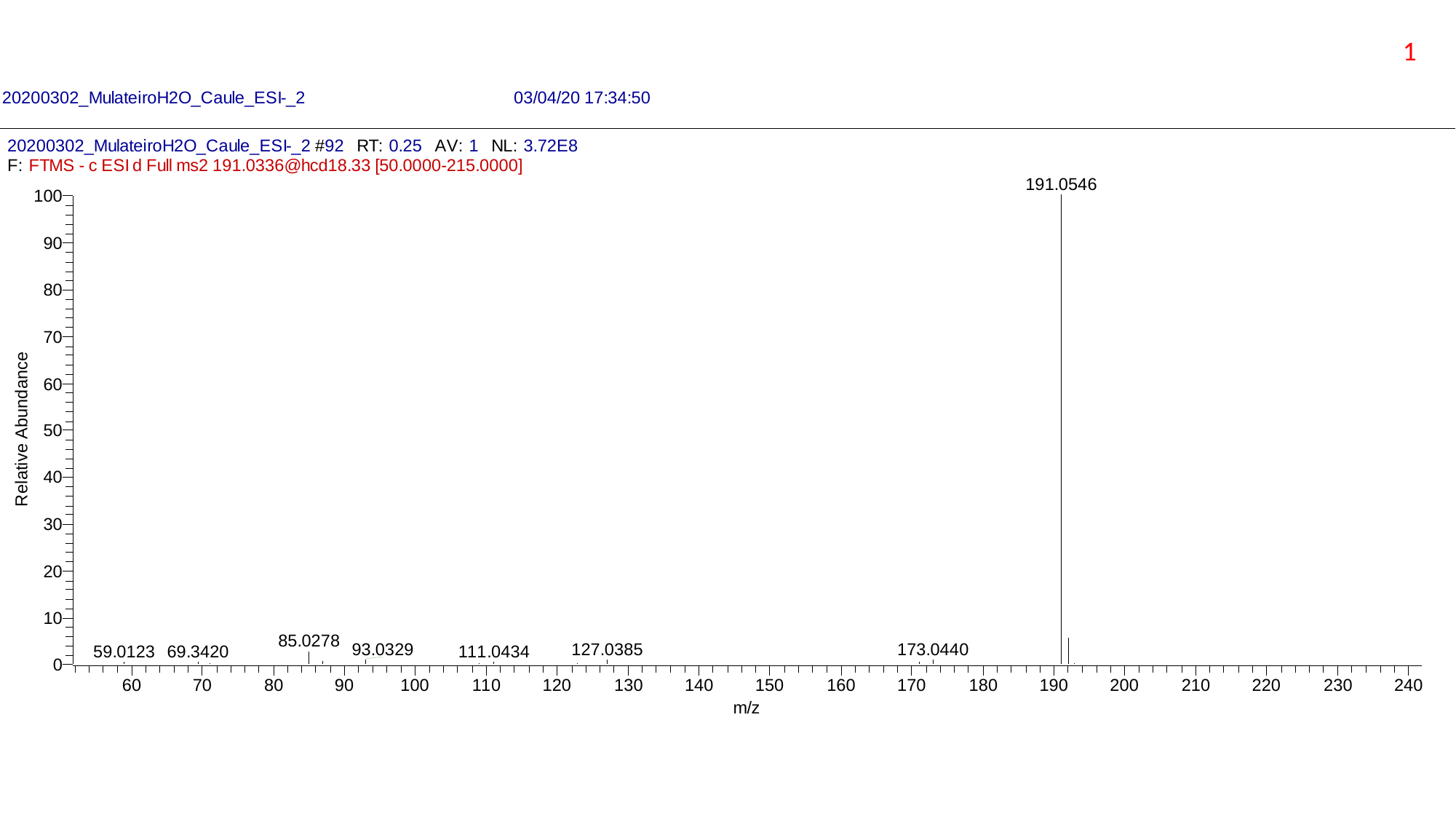

1

## Slide 5
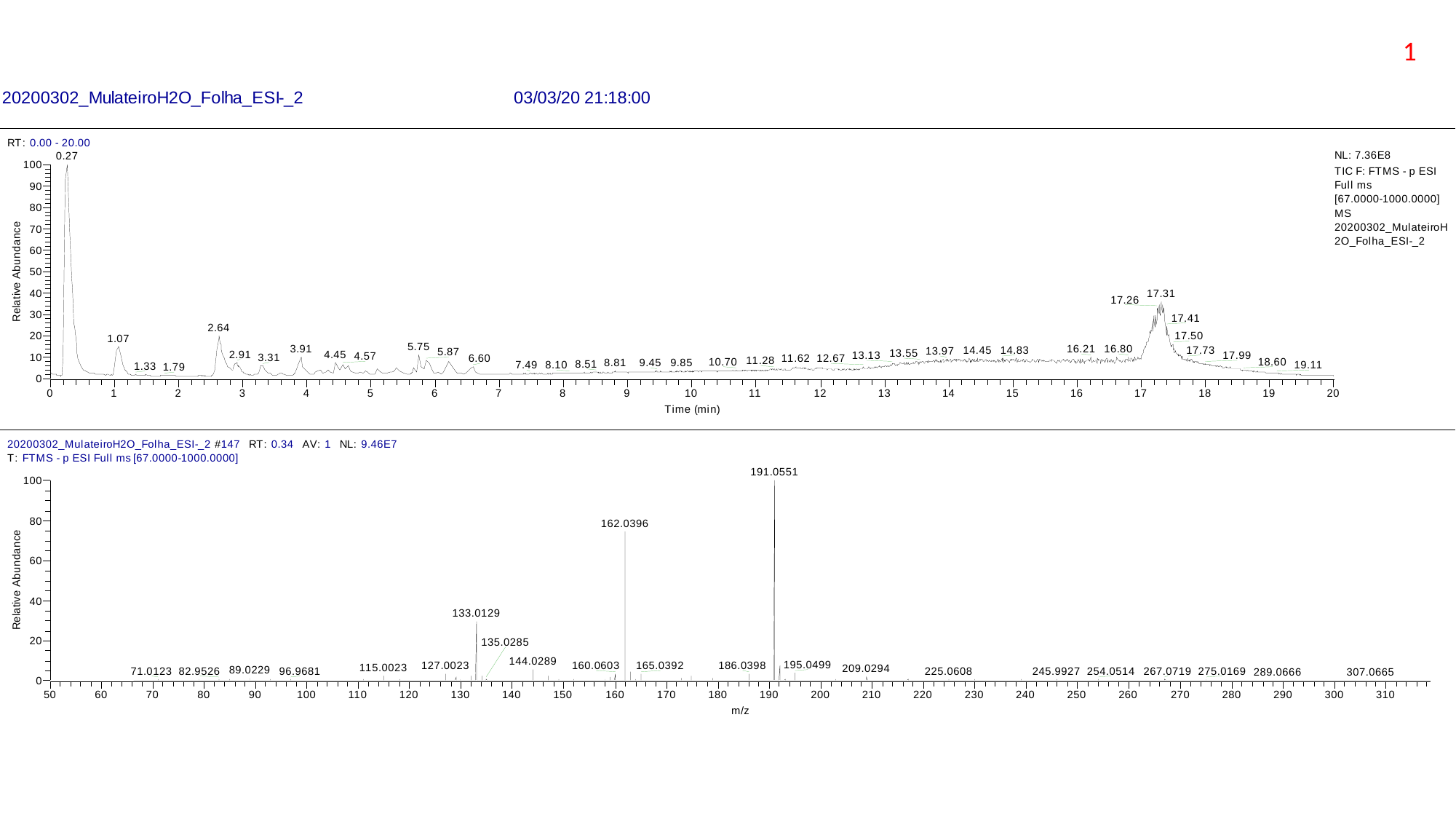

1

## Slide 6
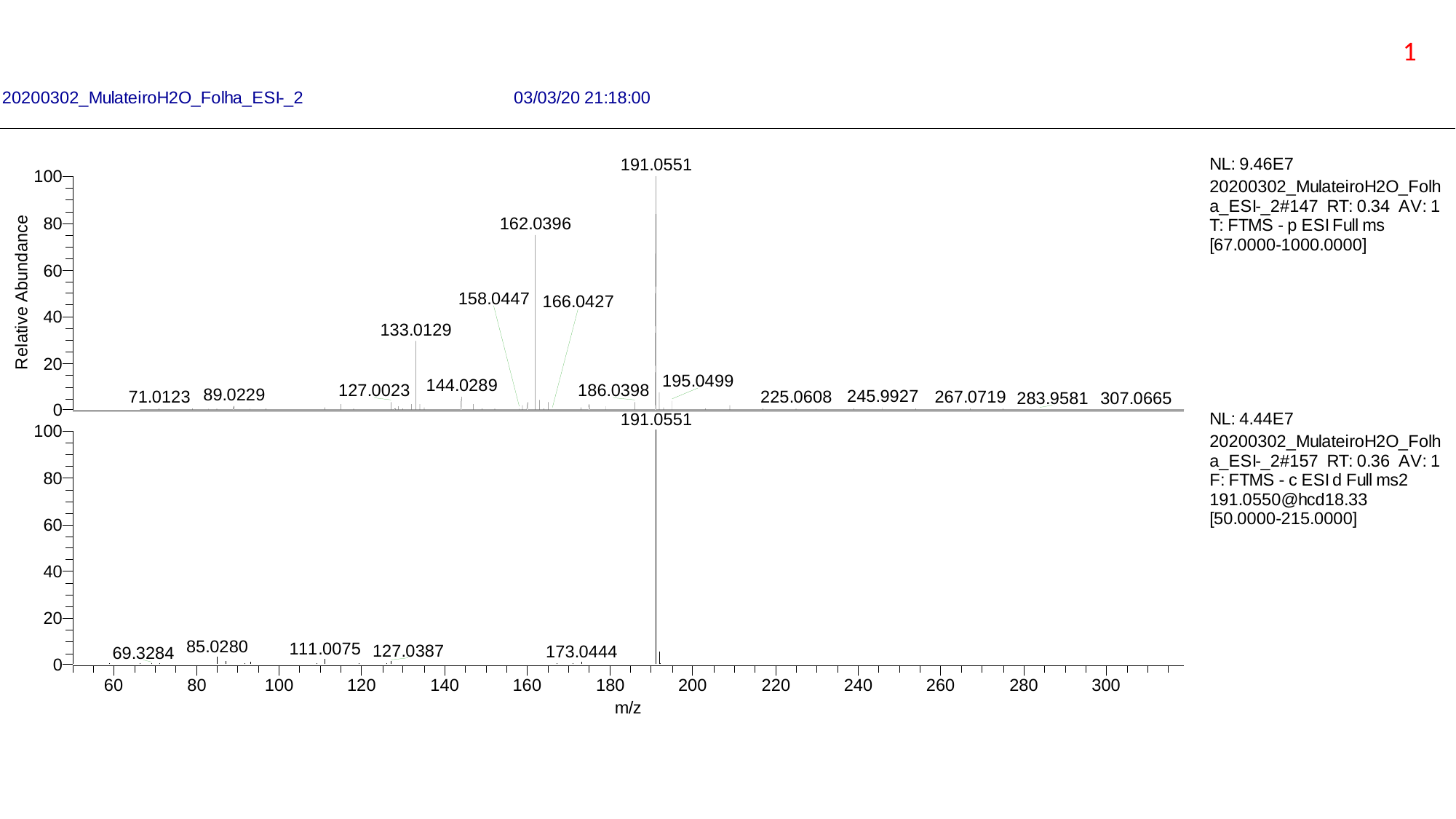

1

## Slide 7
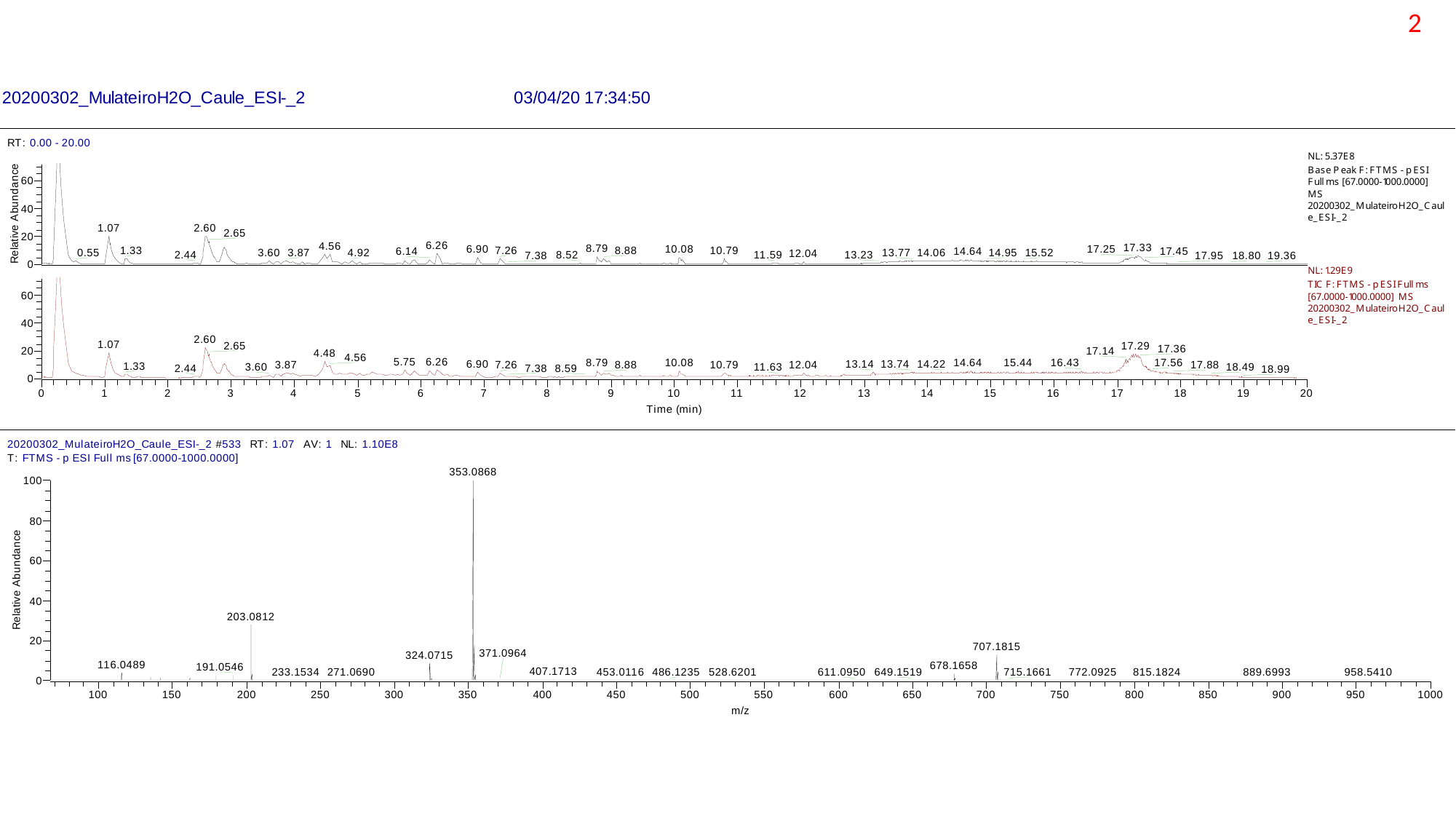

2

## Slide 8
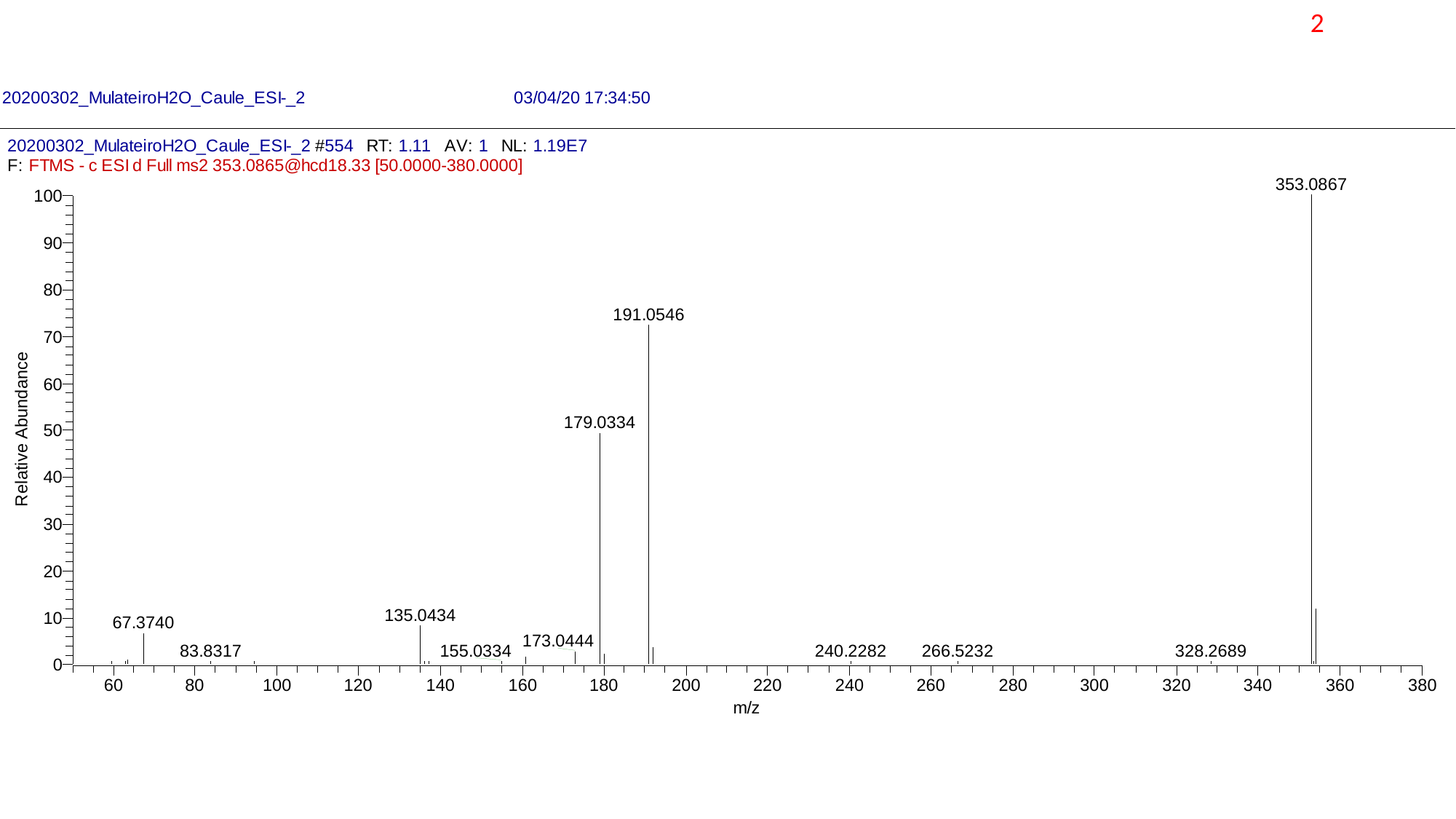

2

## Slide 9
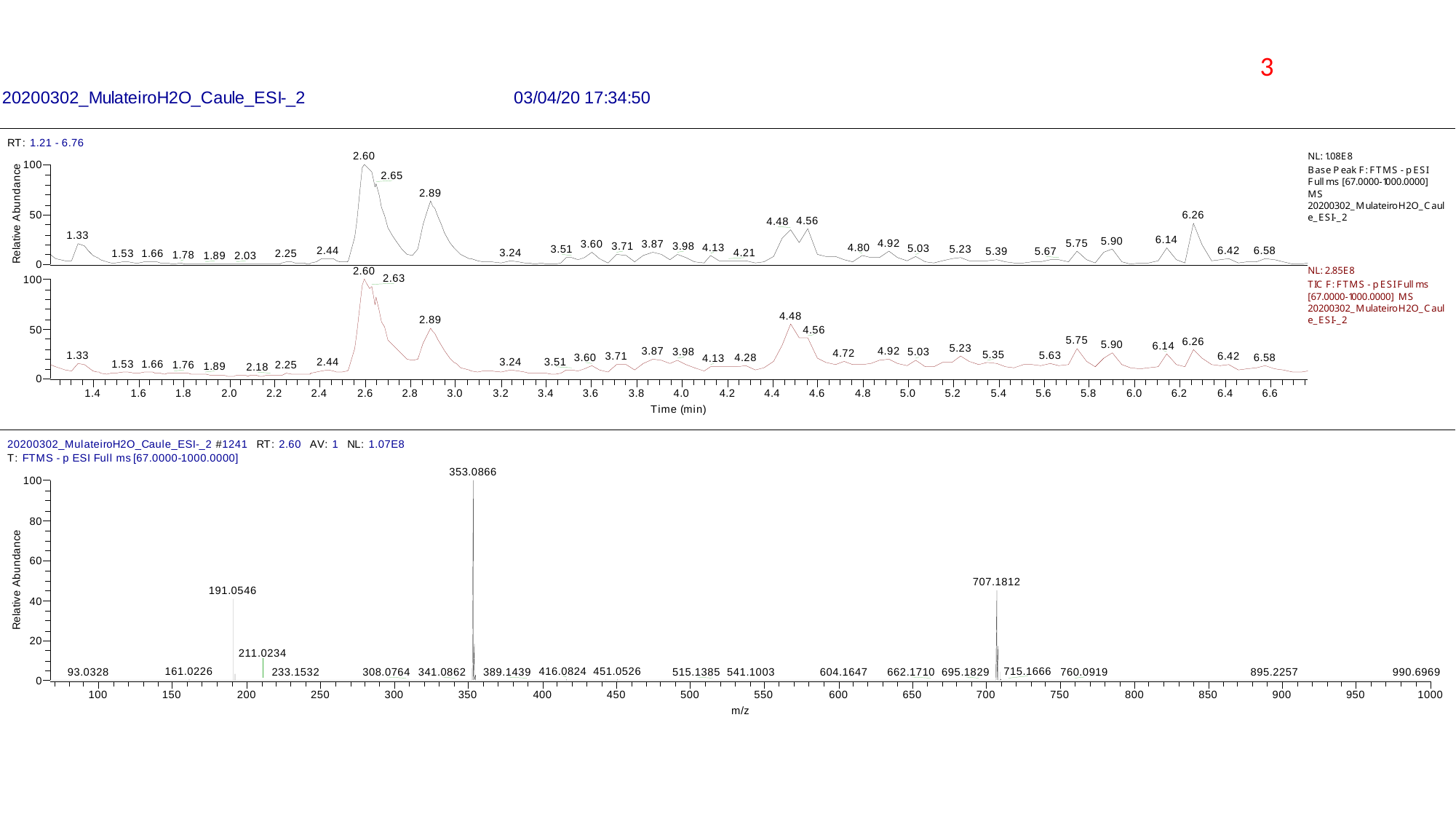

3

## Slide 10
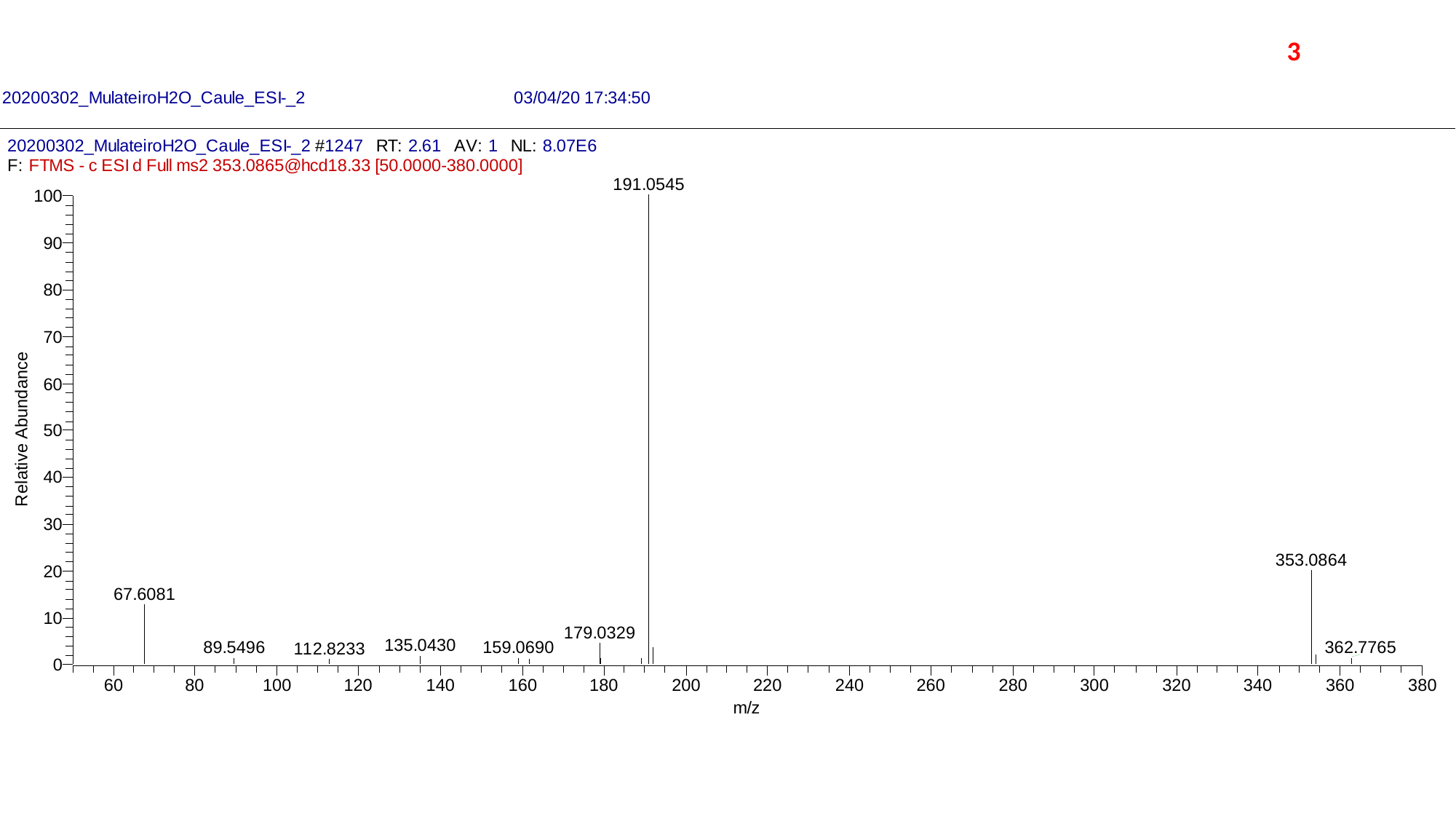

3

## Slide 11
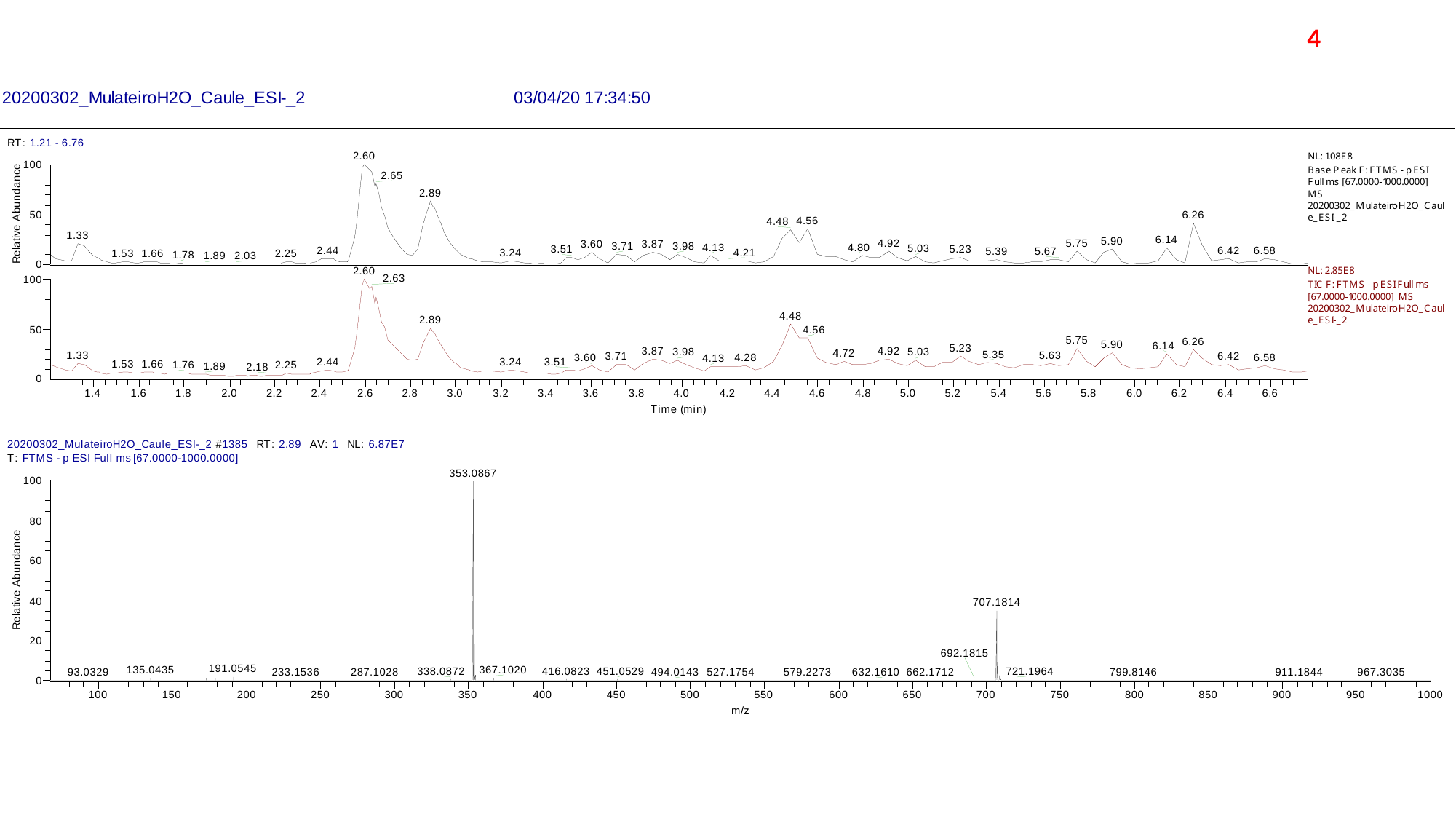

4

## Slide 12
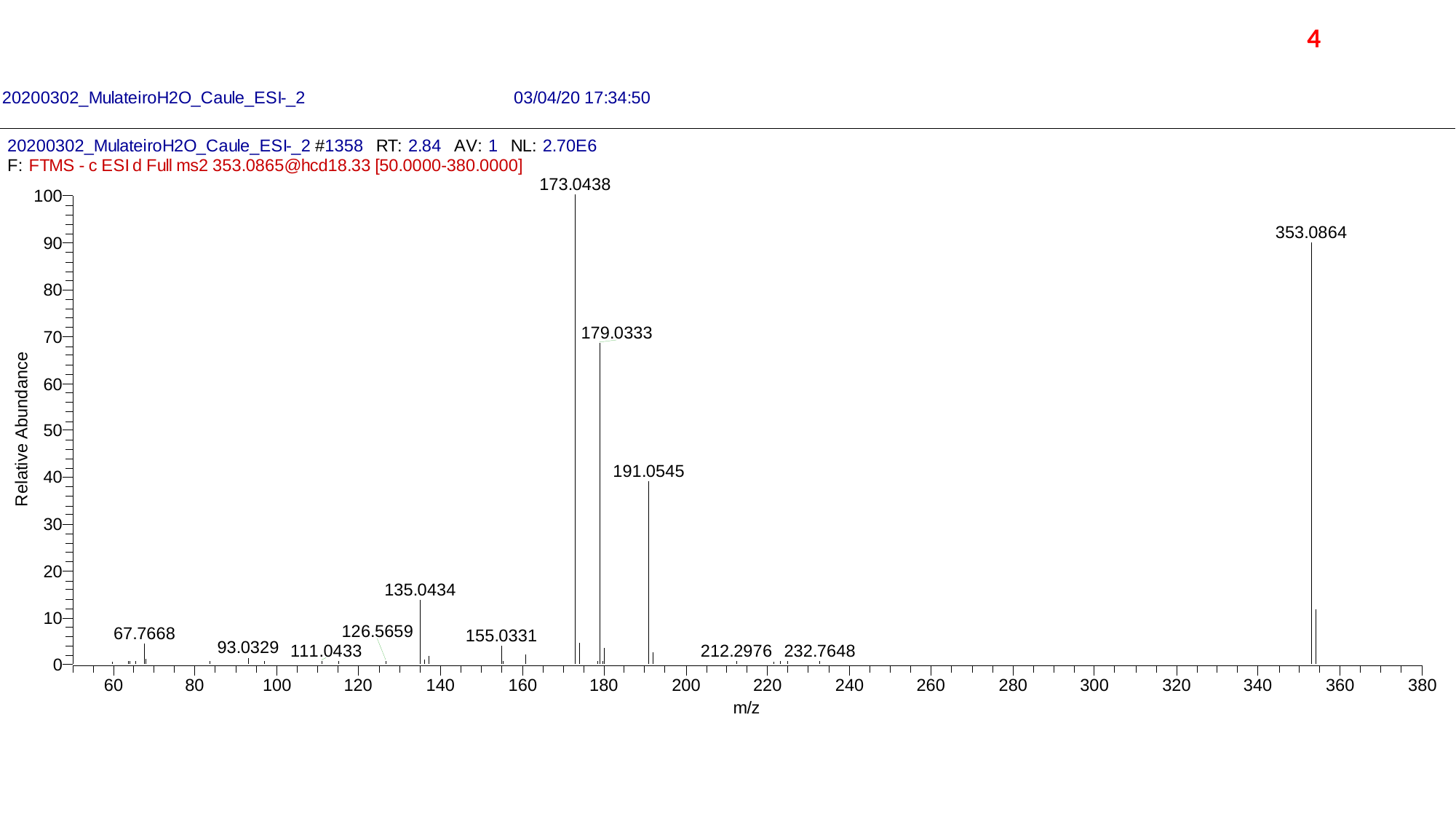

4

## Slide 13
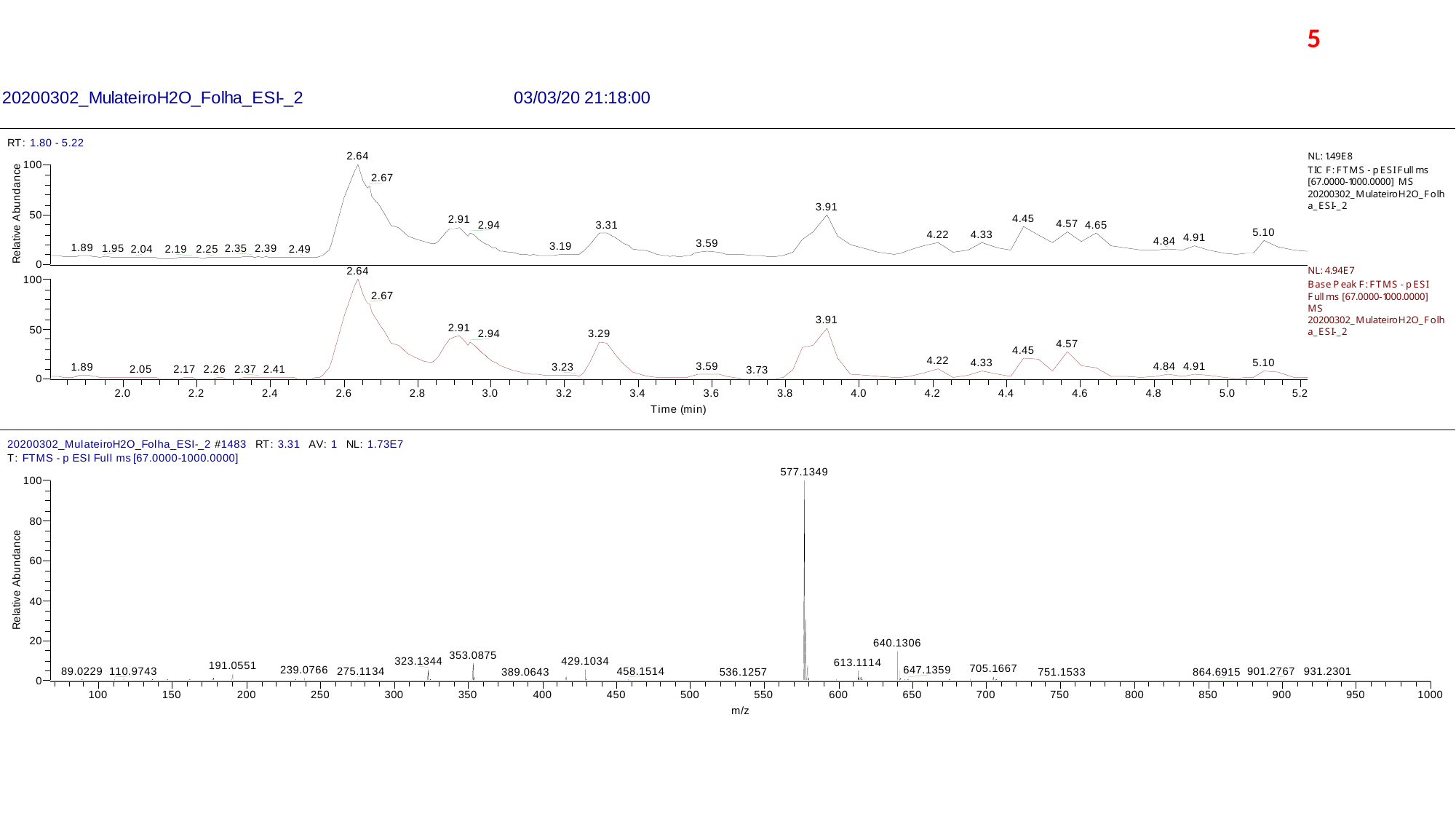

5

## Slide 14
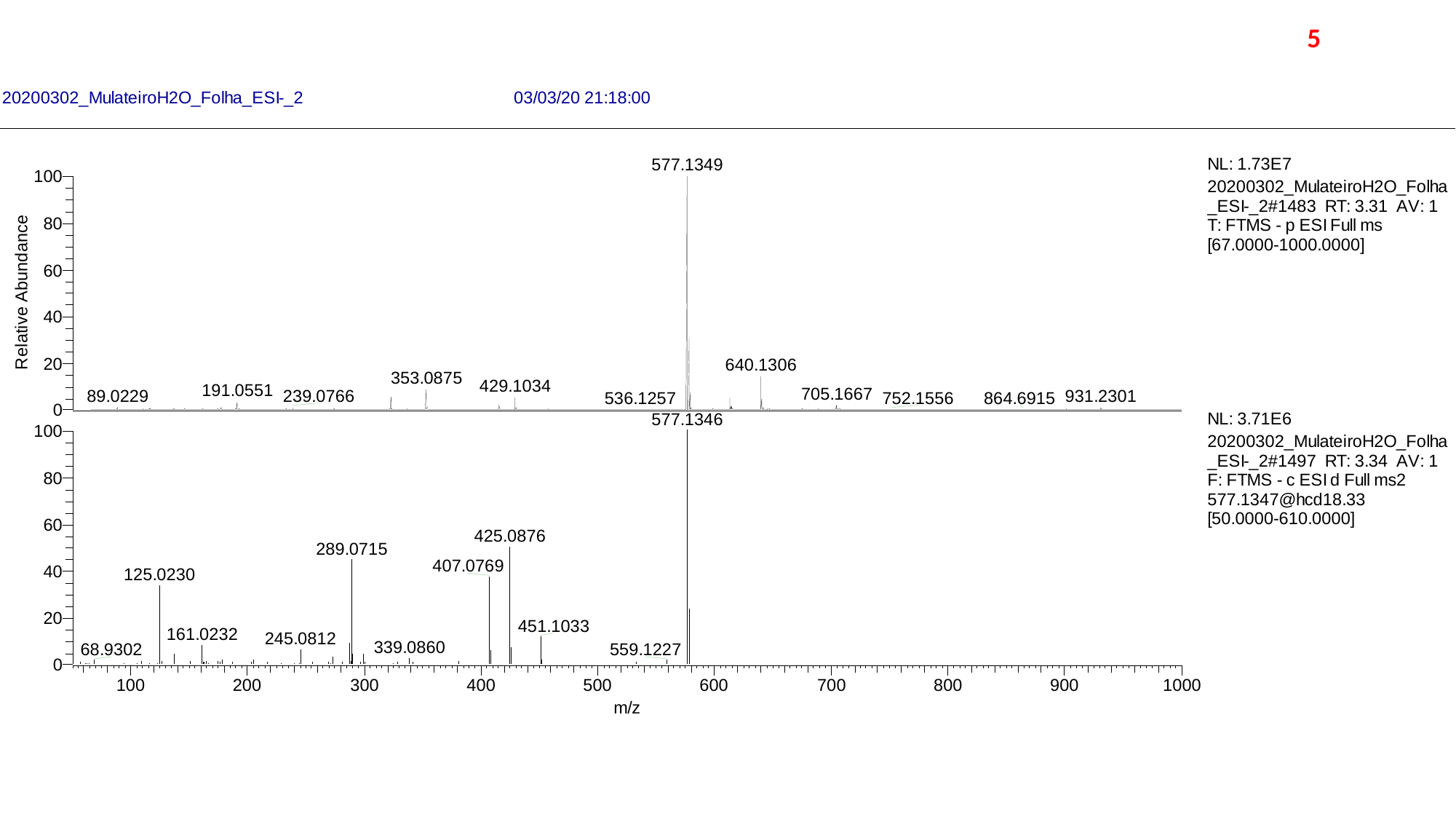

5

## Slide 15
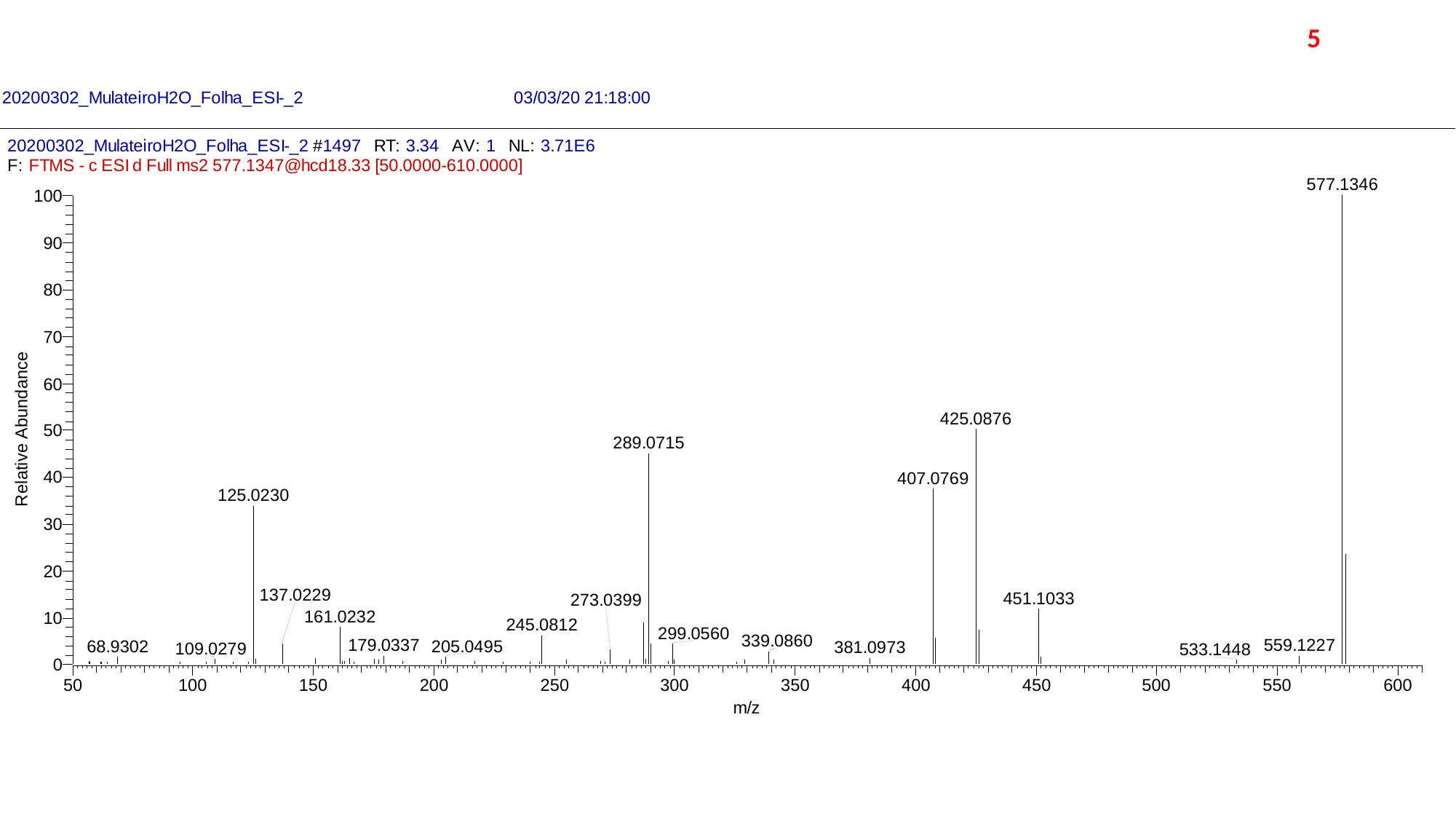

5

## Slide 16
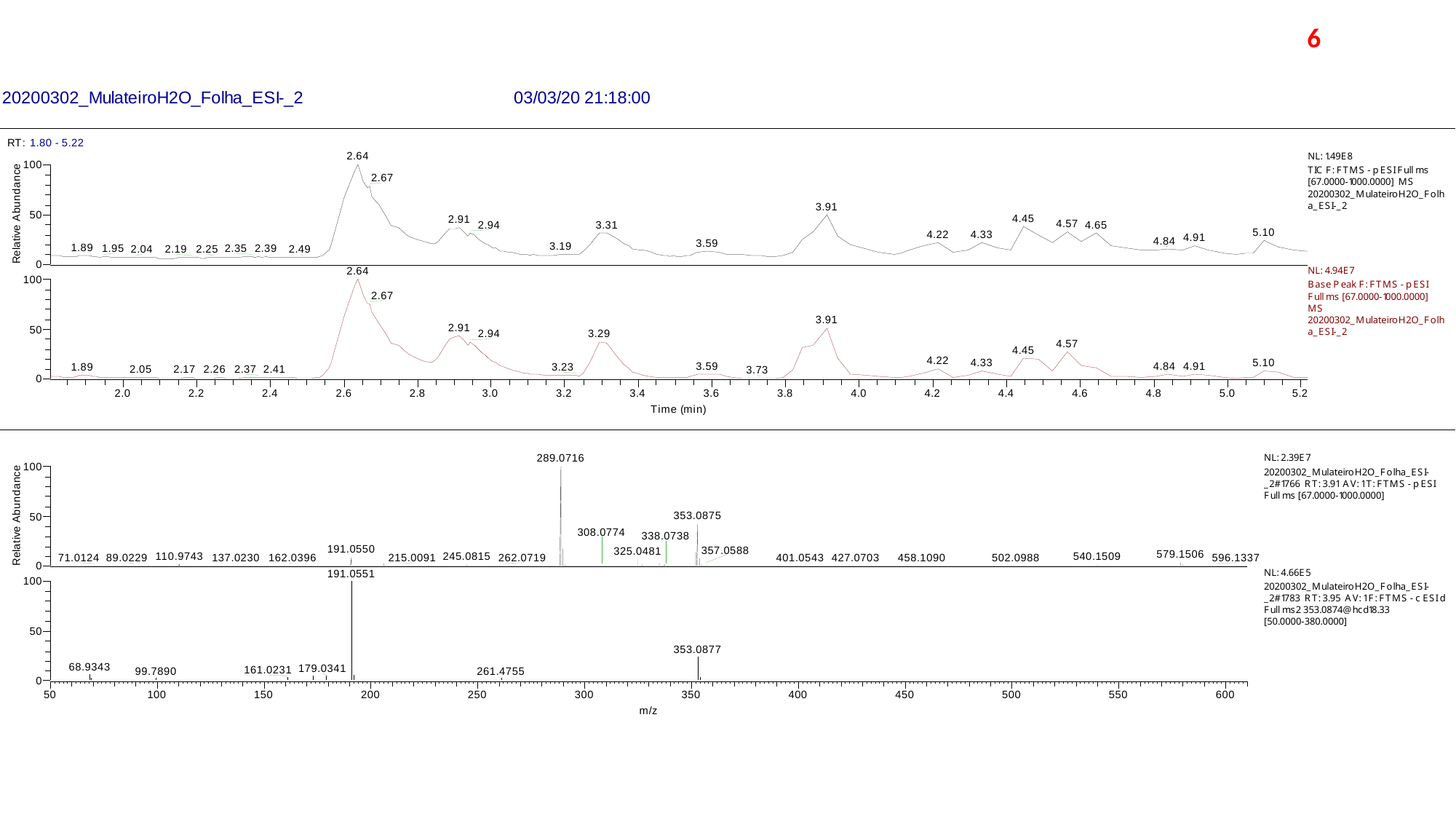

6

## Slide 17
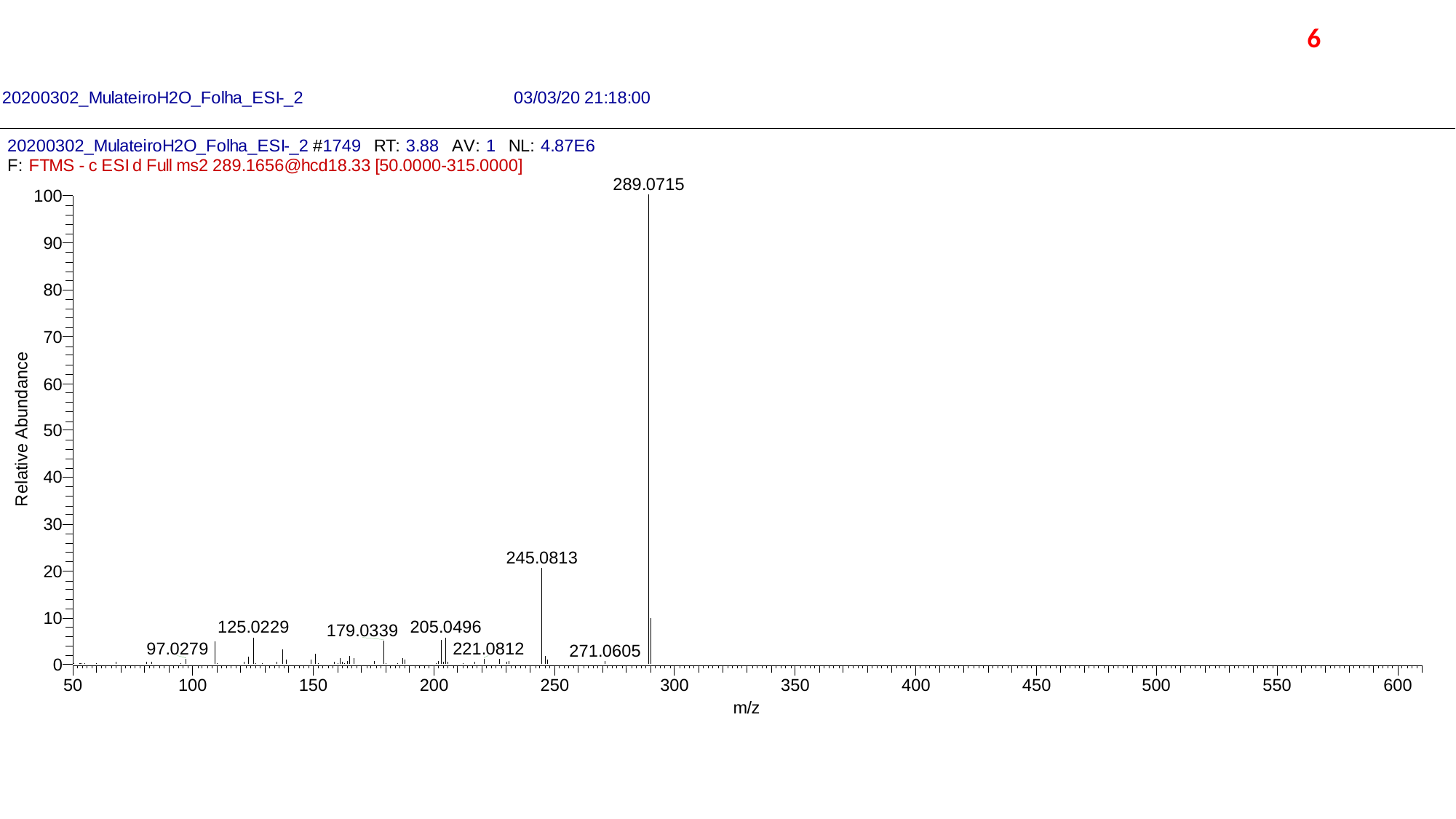

6

## Slide 18
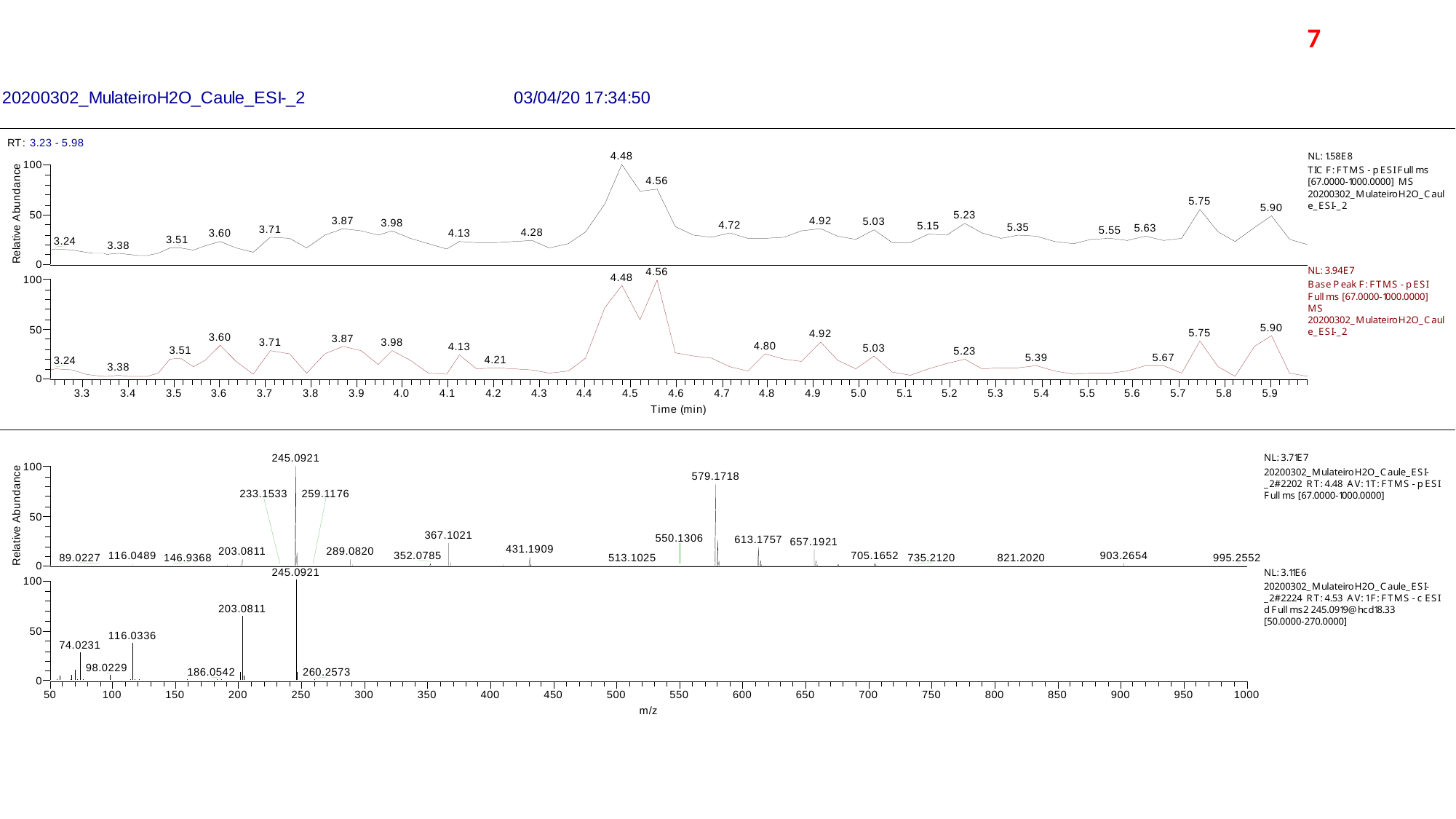

7

## Slide 19
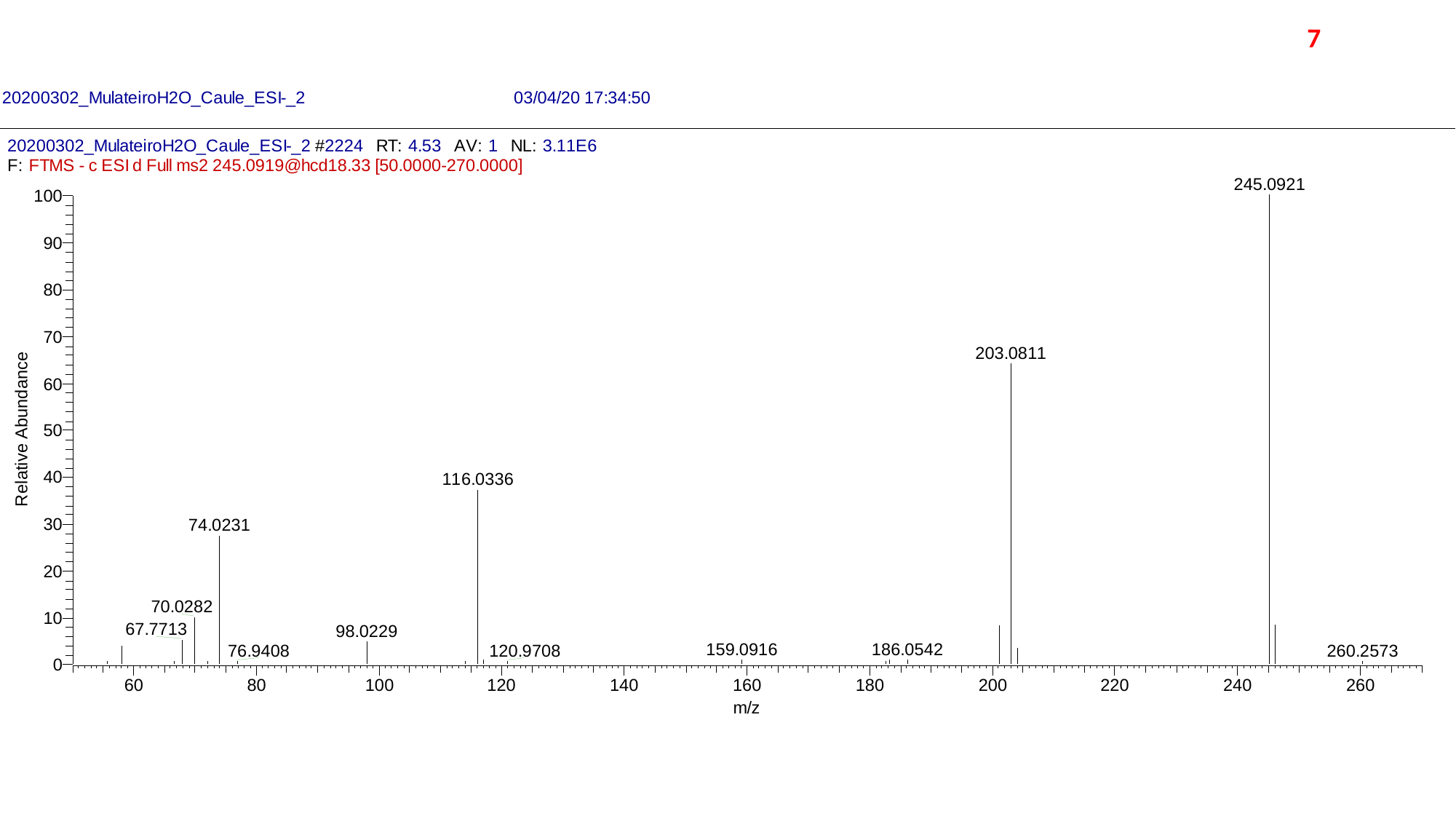

7

## Slide 20
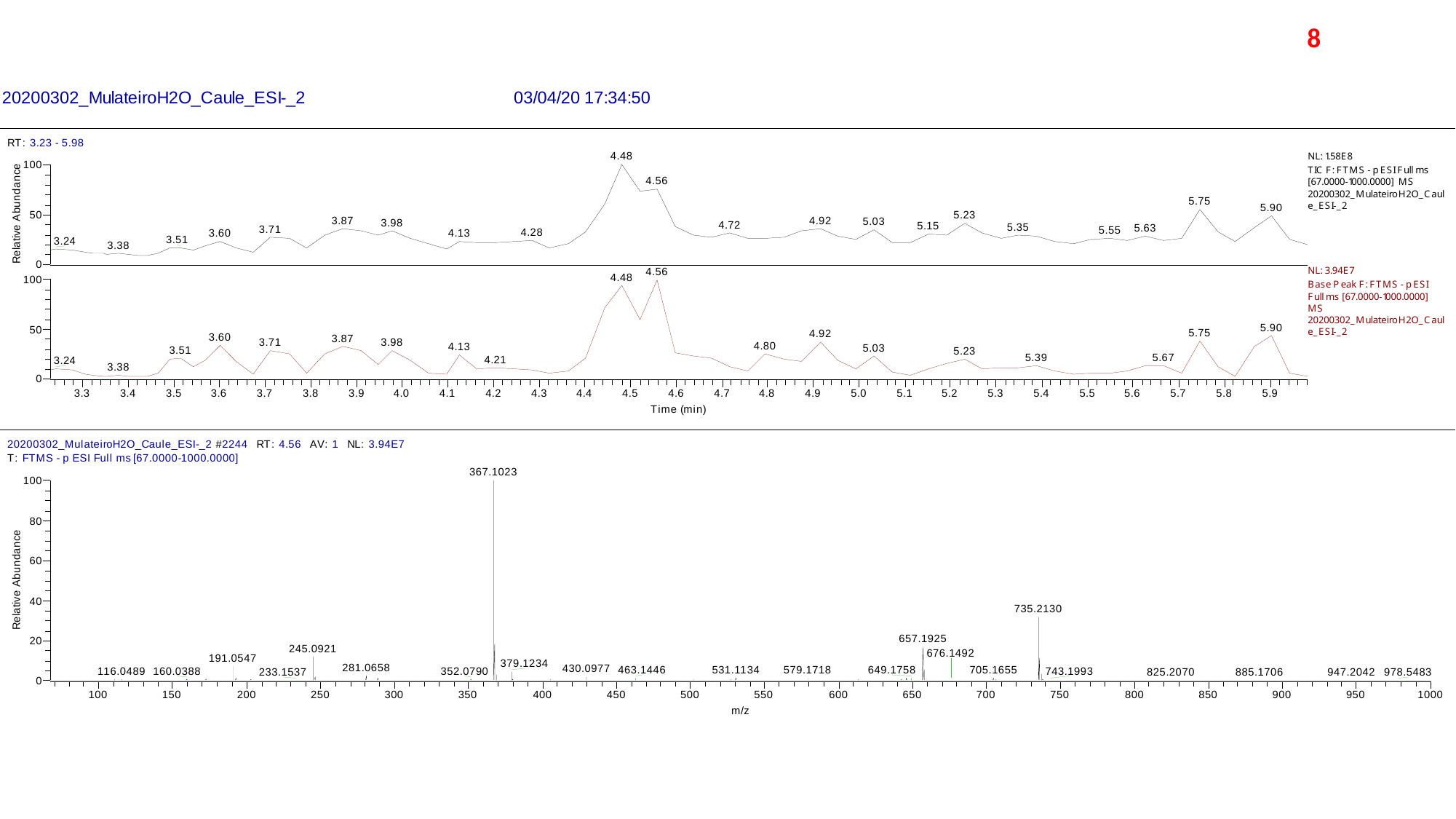

8

## Slide 21
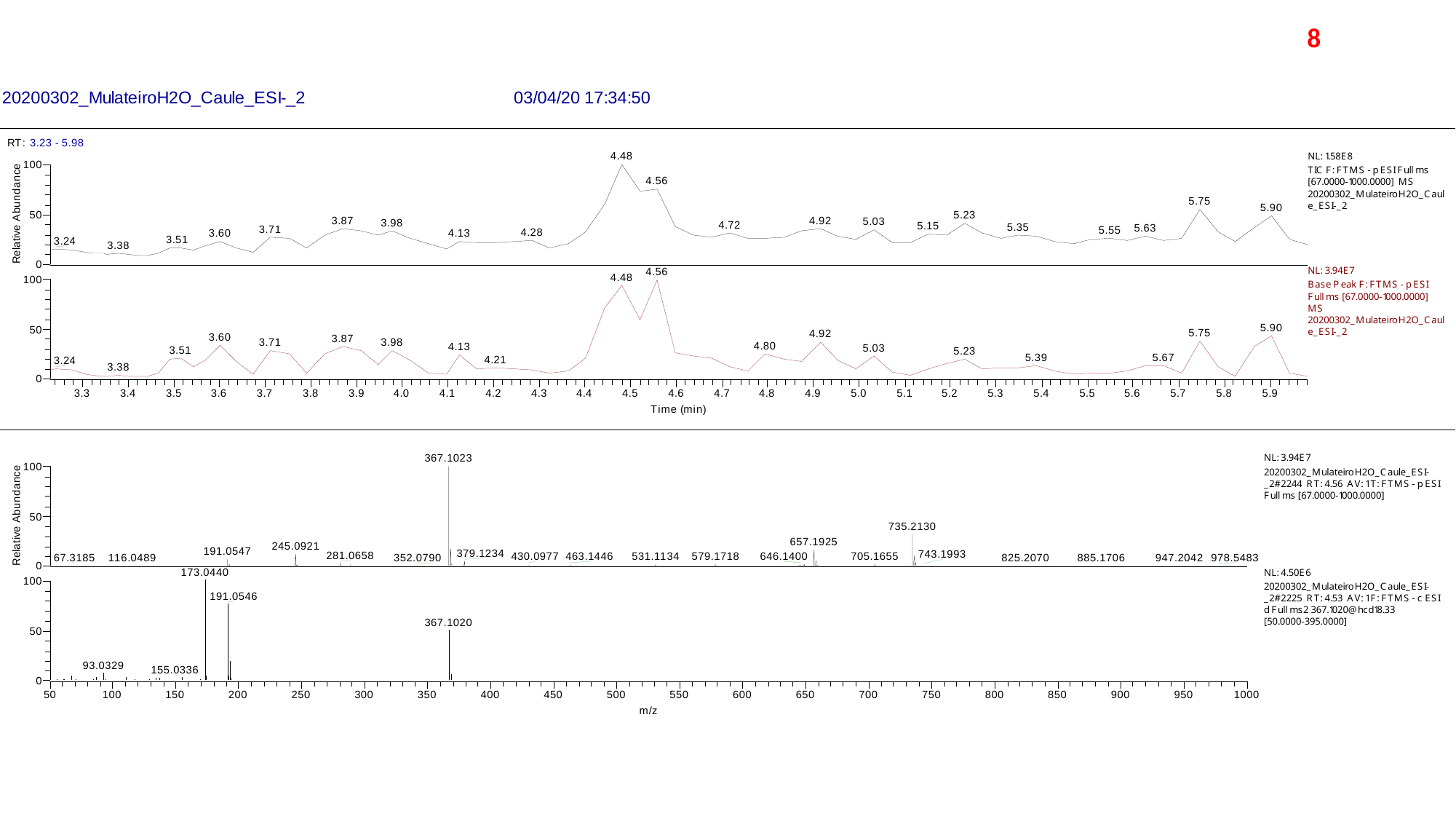

8

## Slide 22
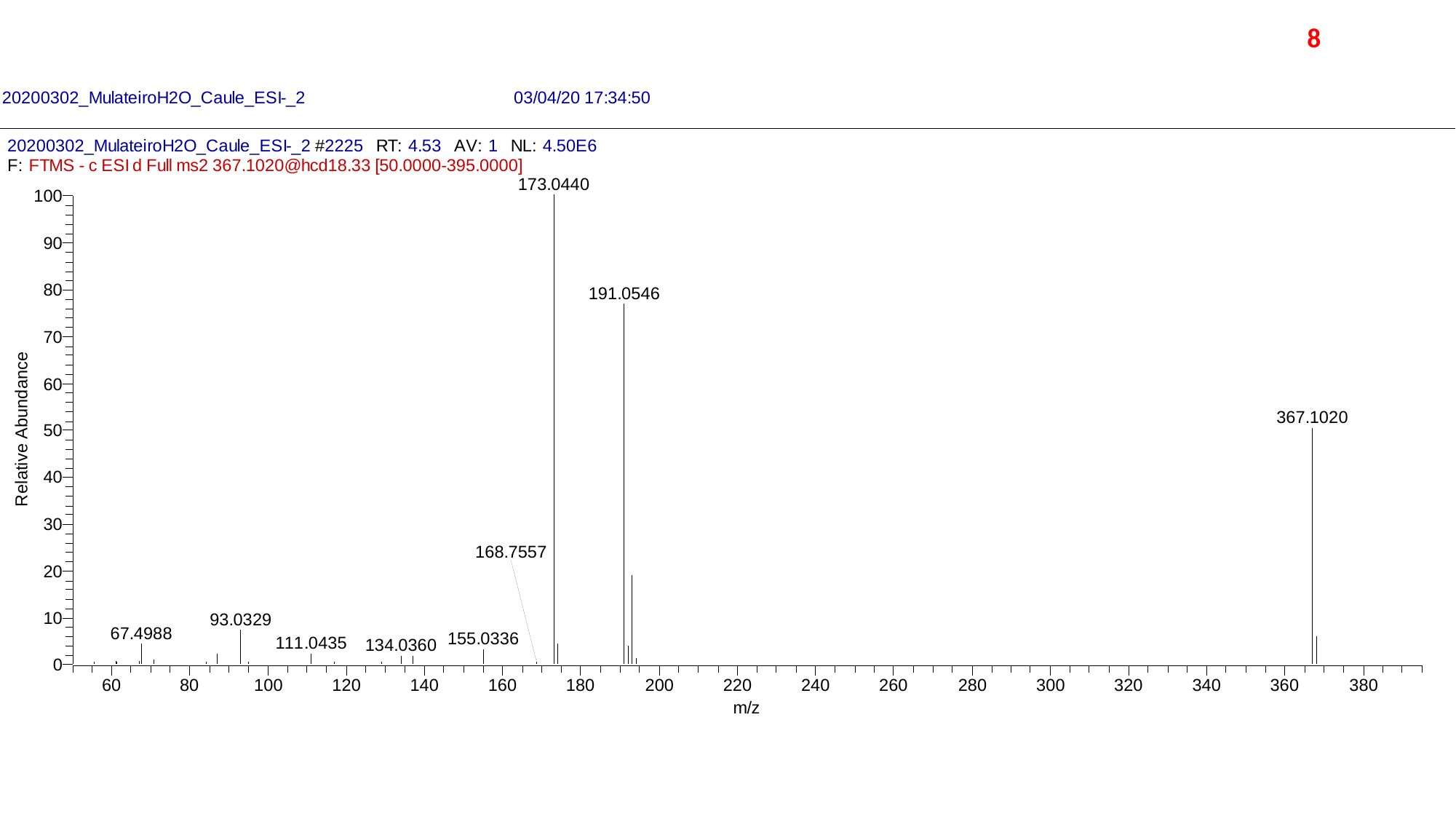

8

## Slide 23
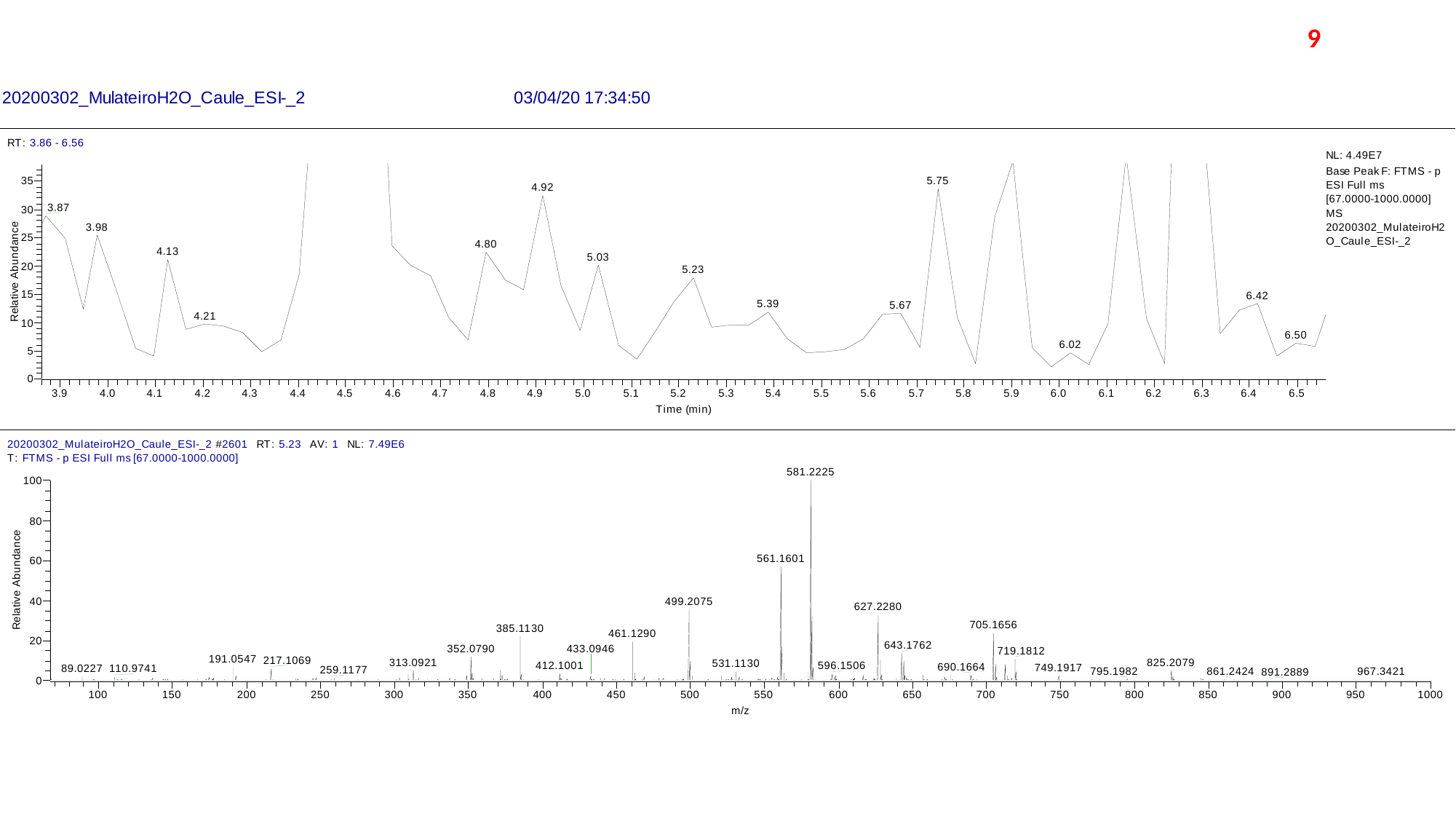

9

## Slide 24
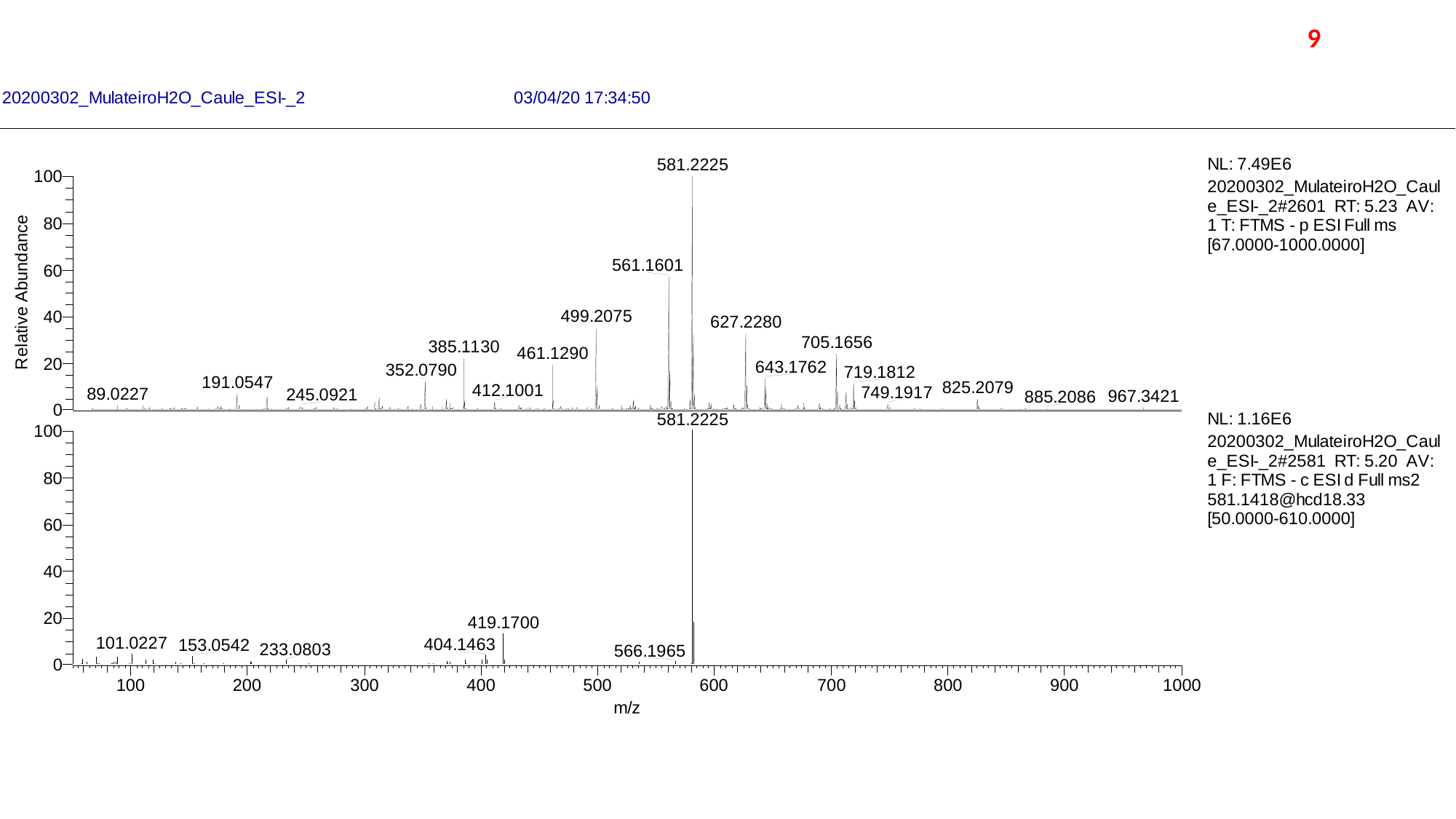

9

## Slide 25
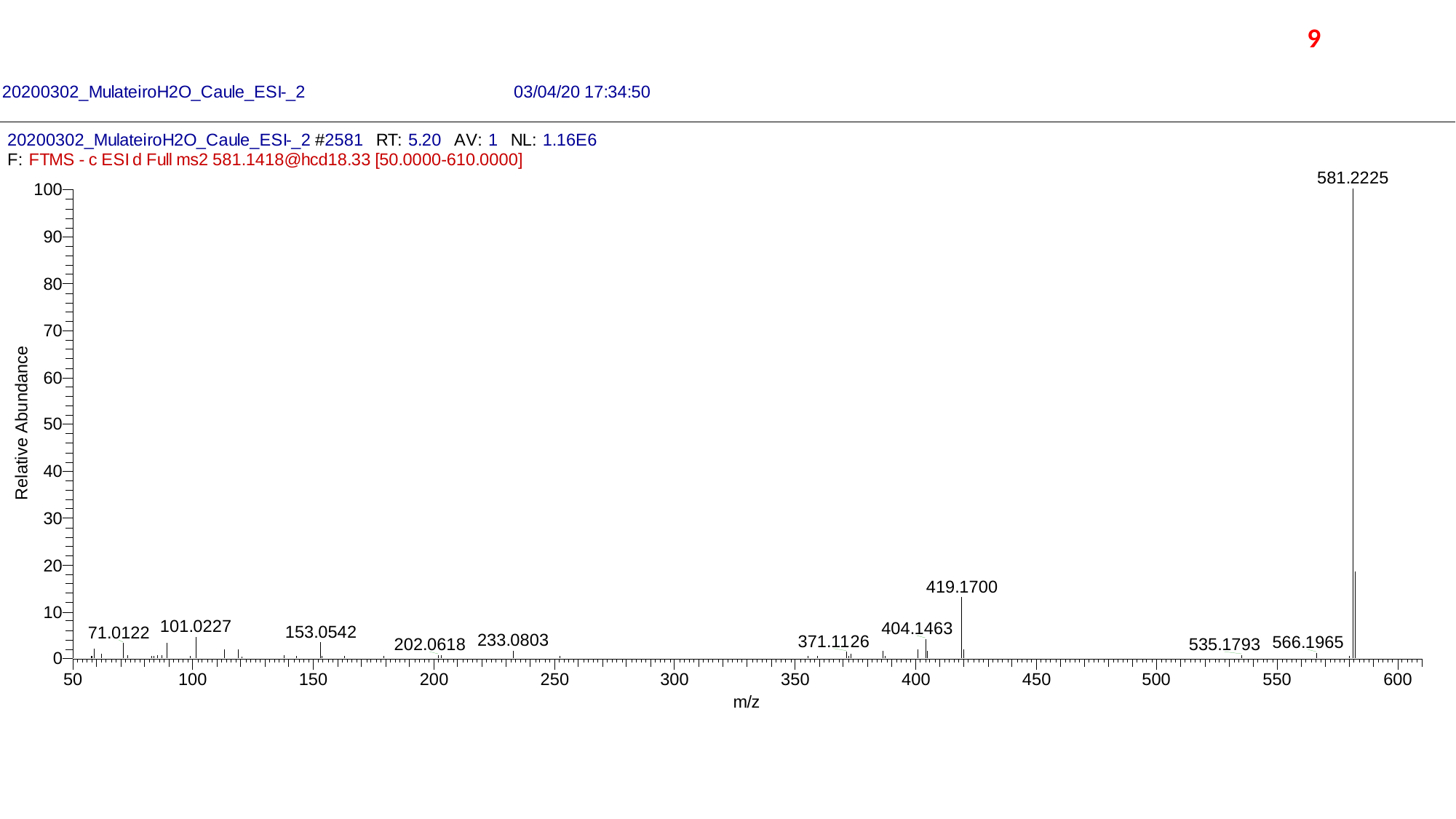

9

## Slide 26
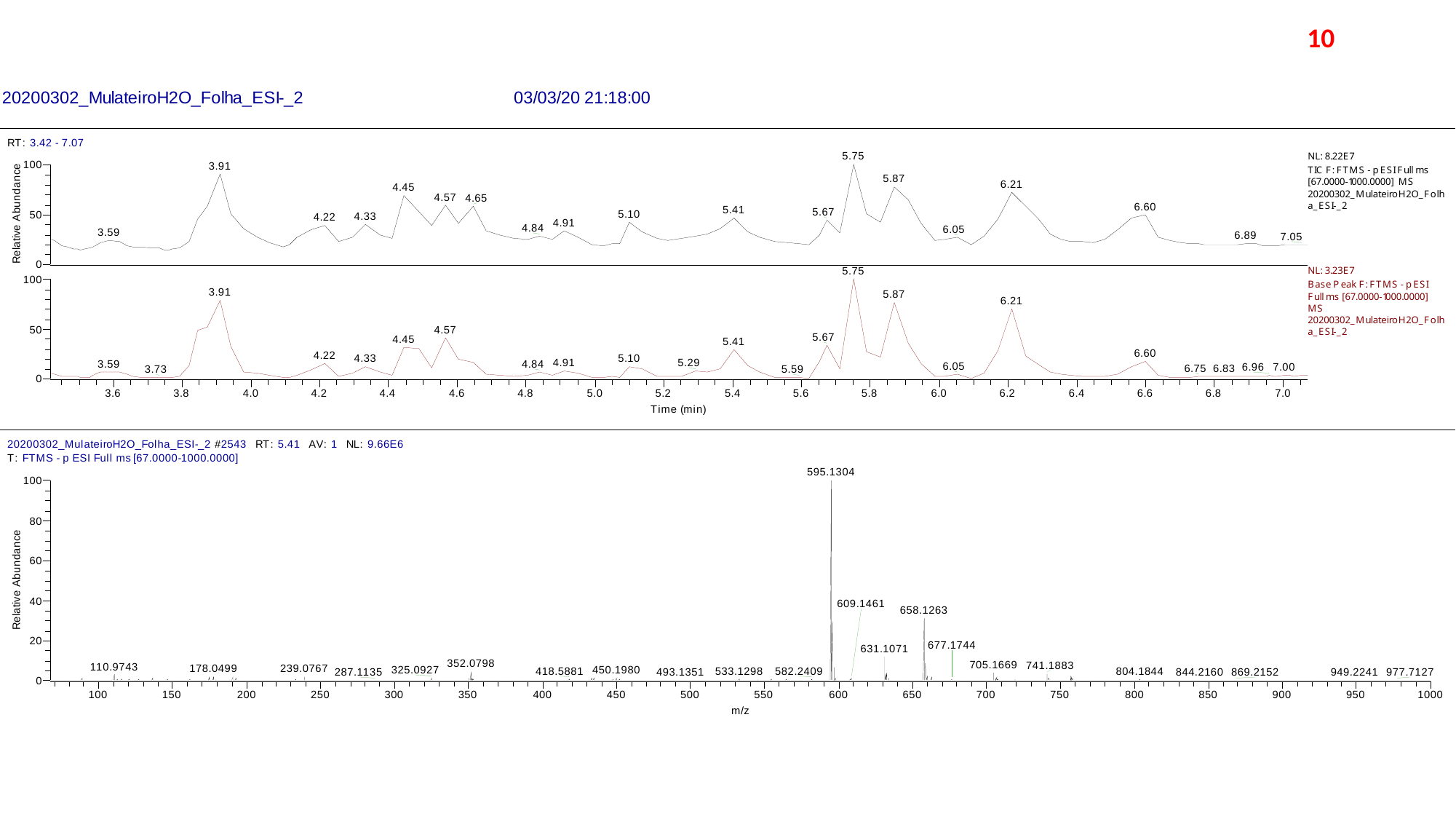

10

## Slide 27
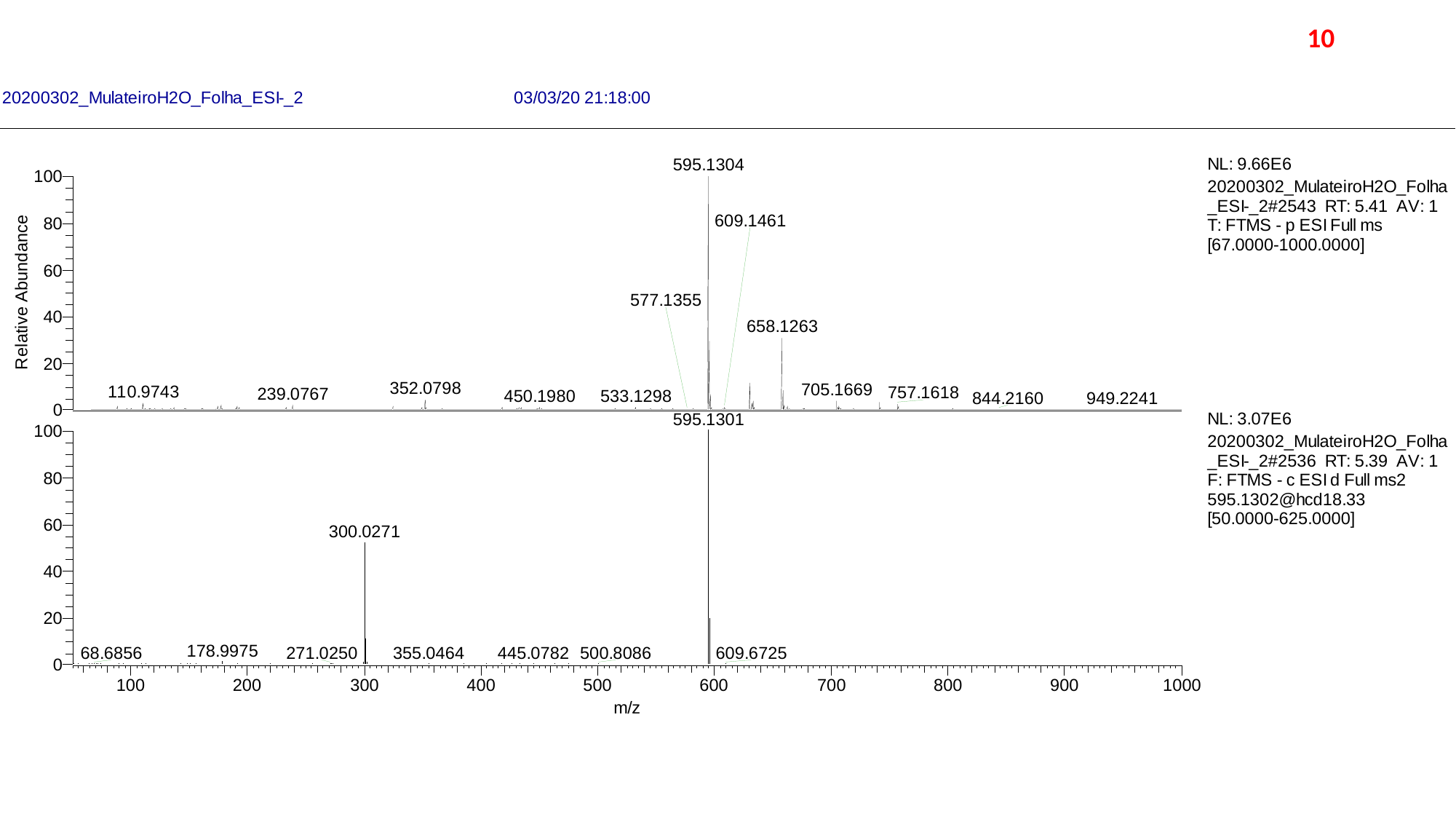

10

## Slide 28
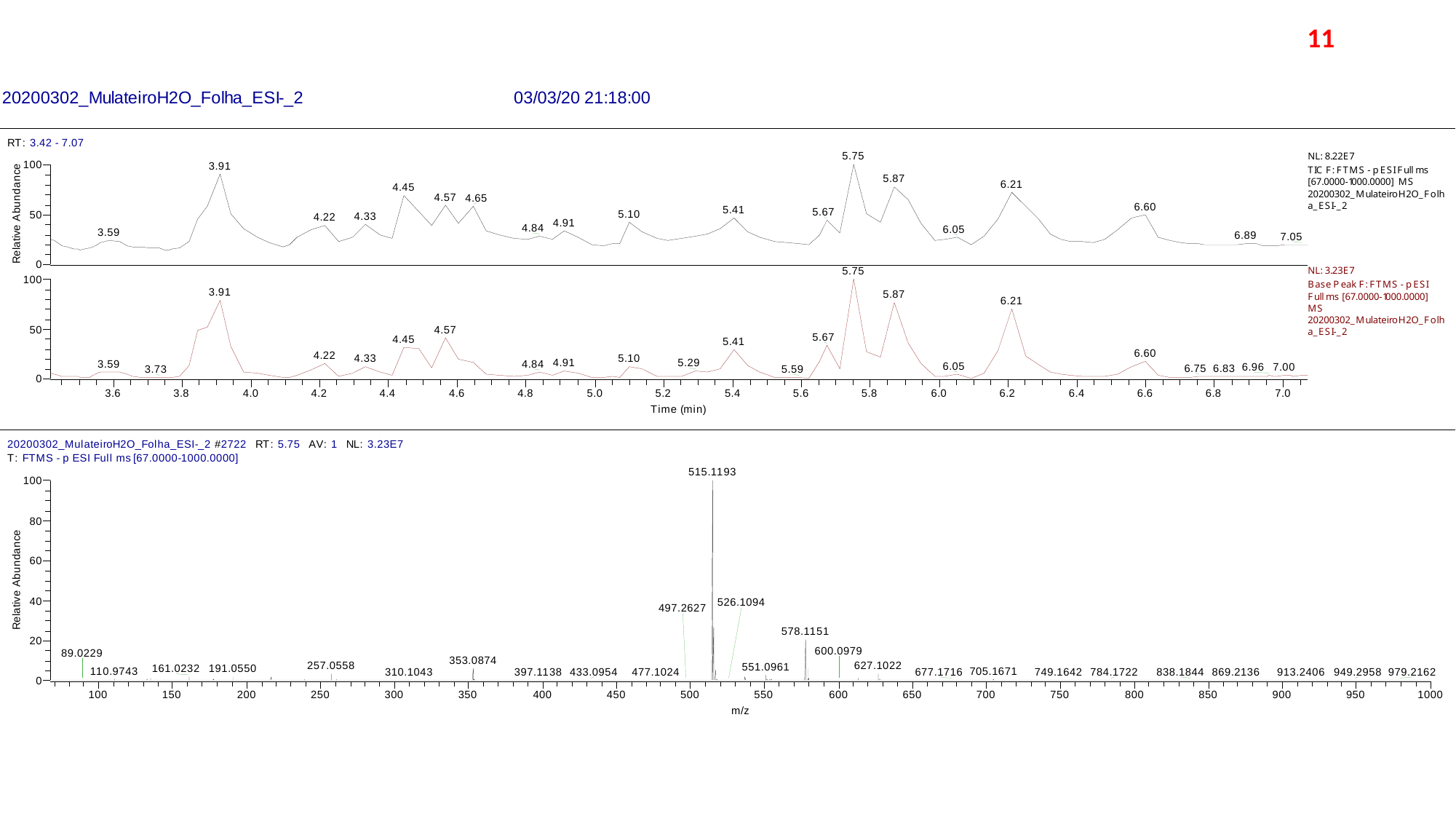

11

## Slide 29
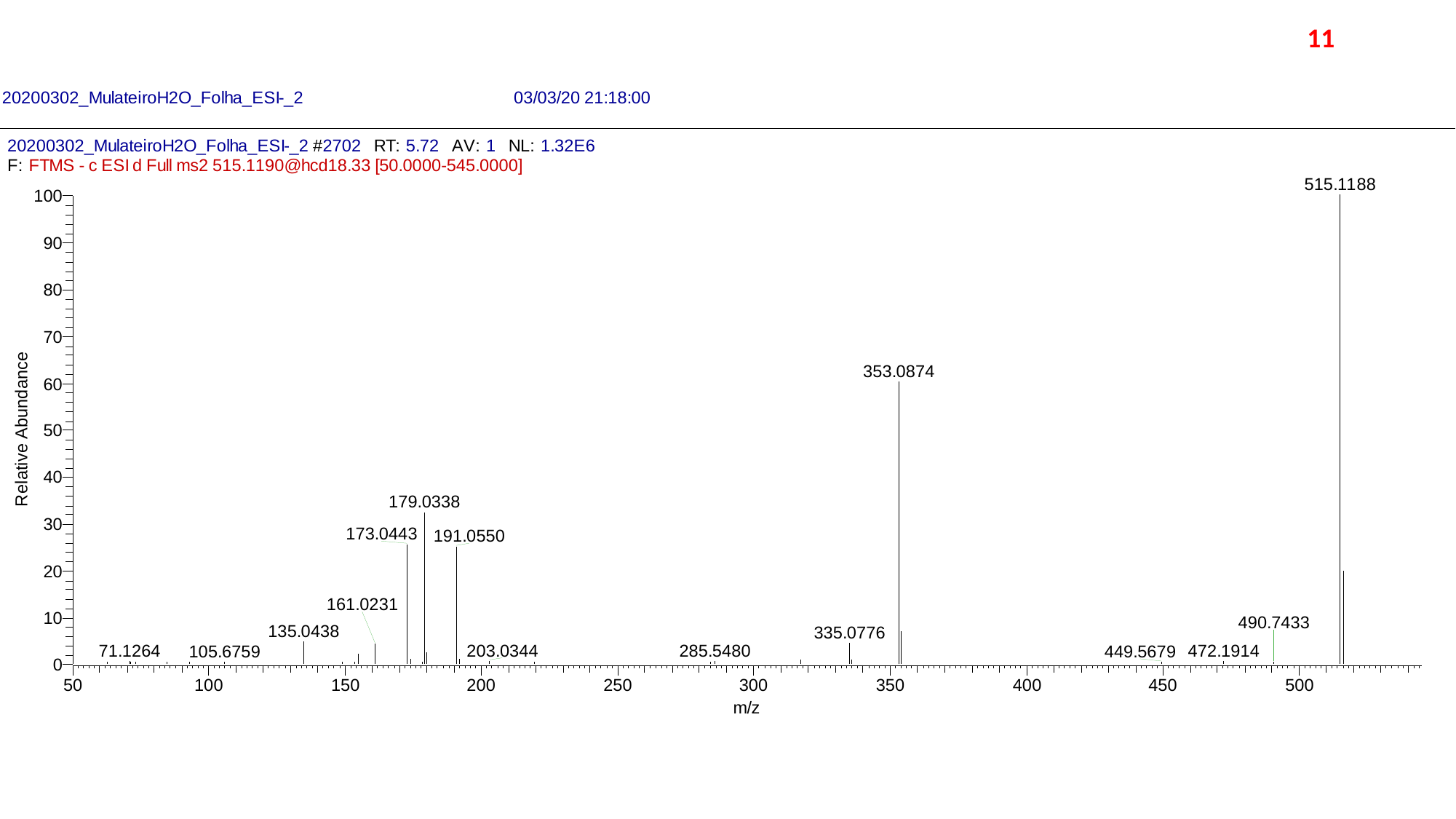

11

## Slide 30
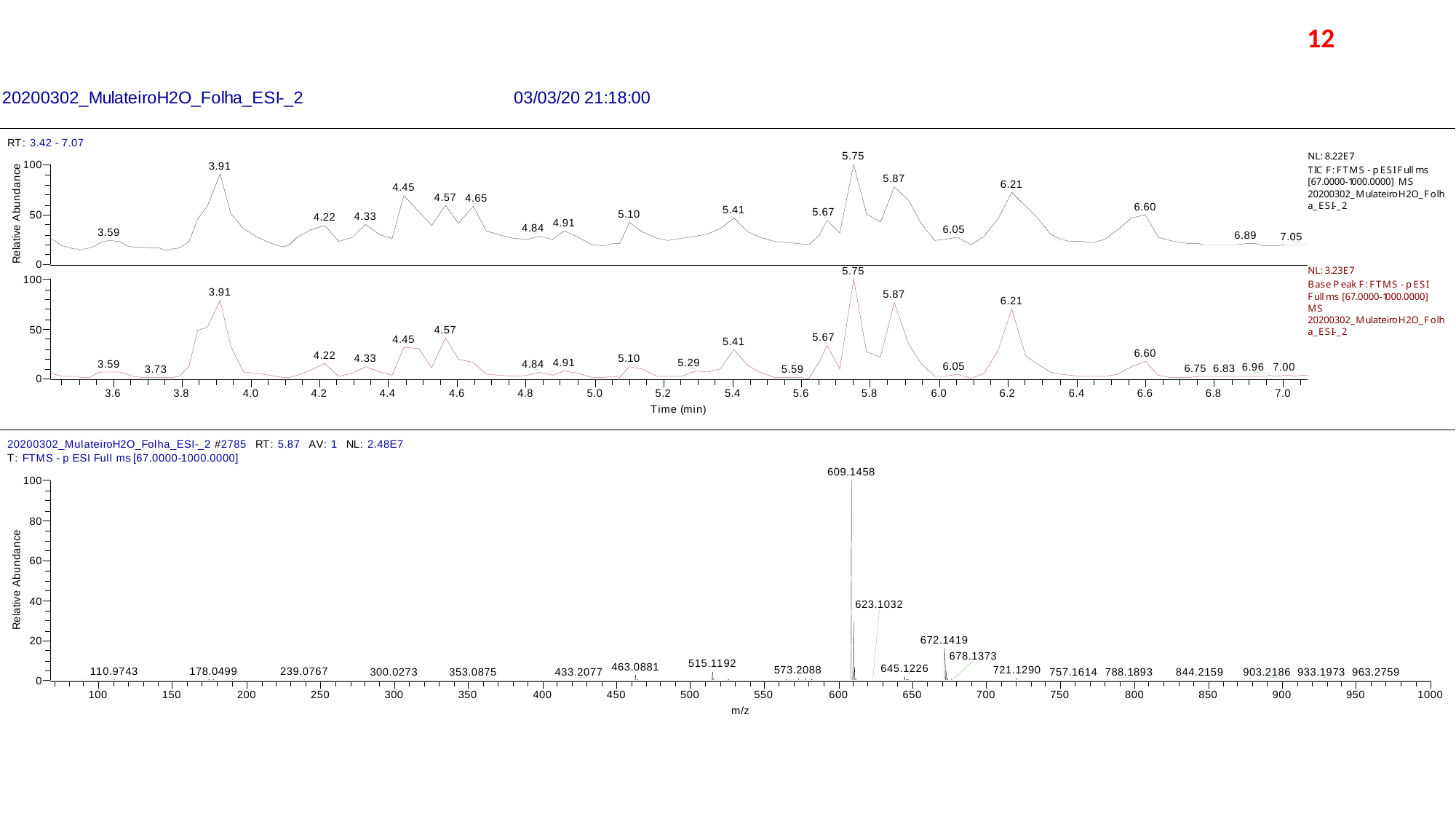

12

## Slide 31
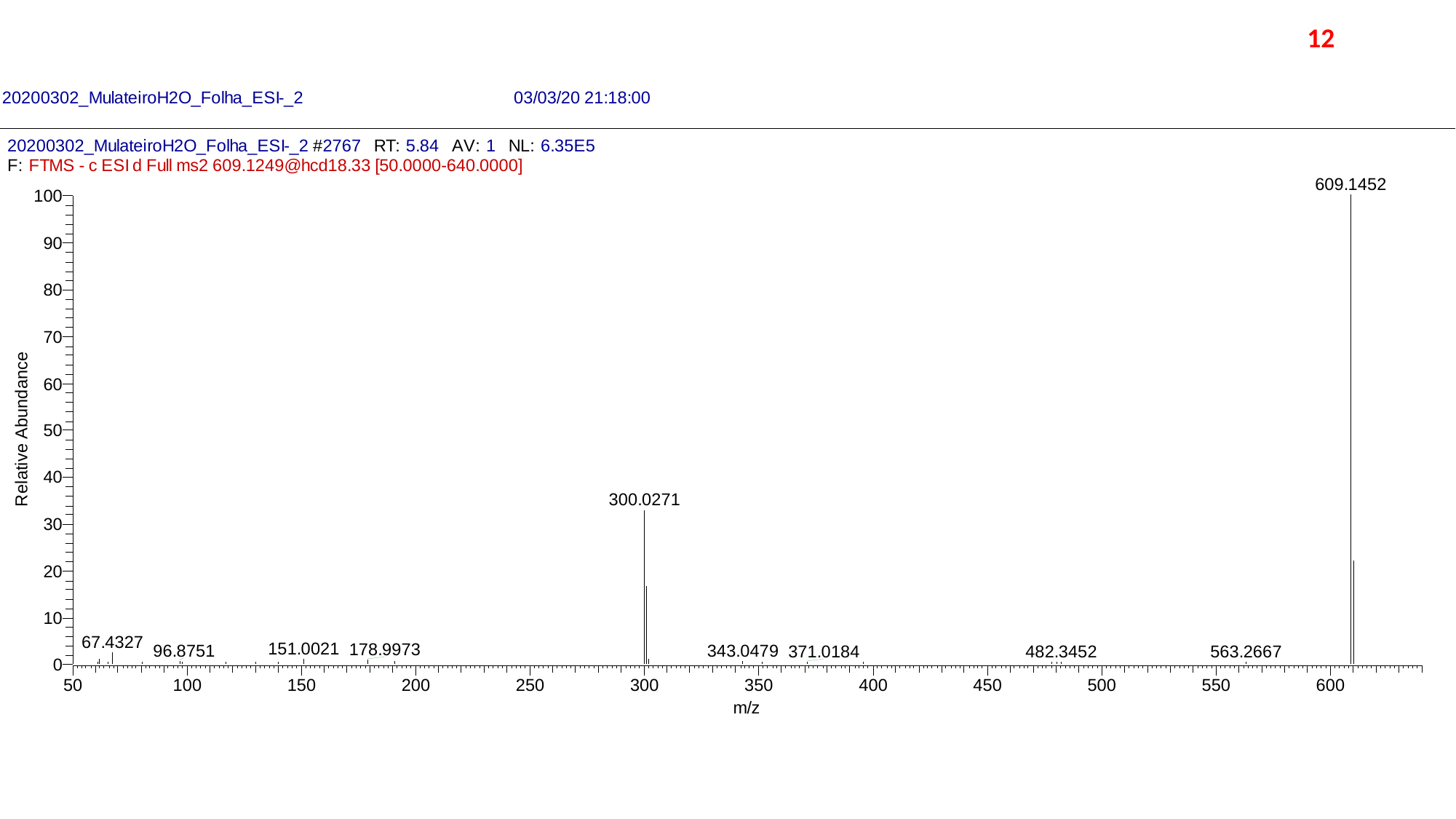

12

## Slide 32
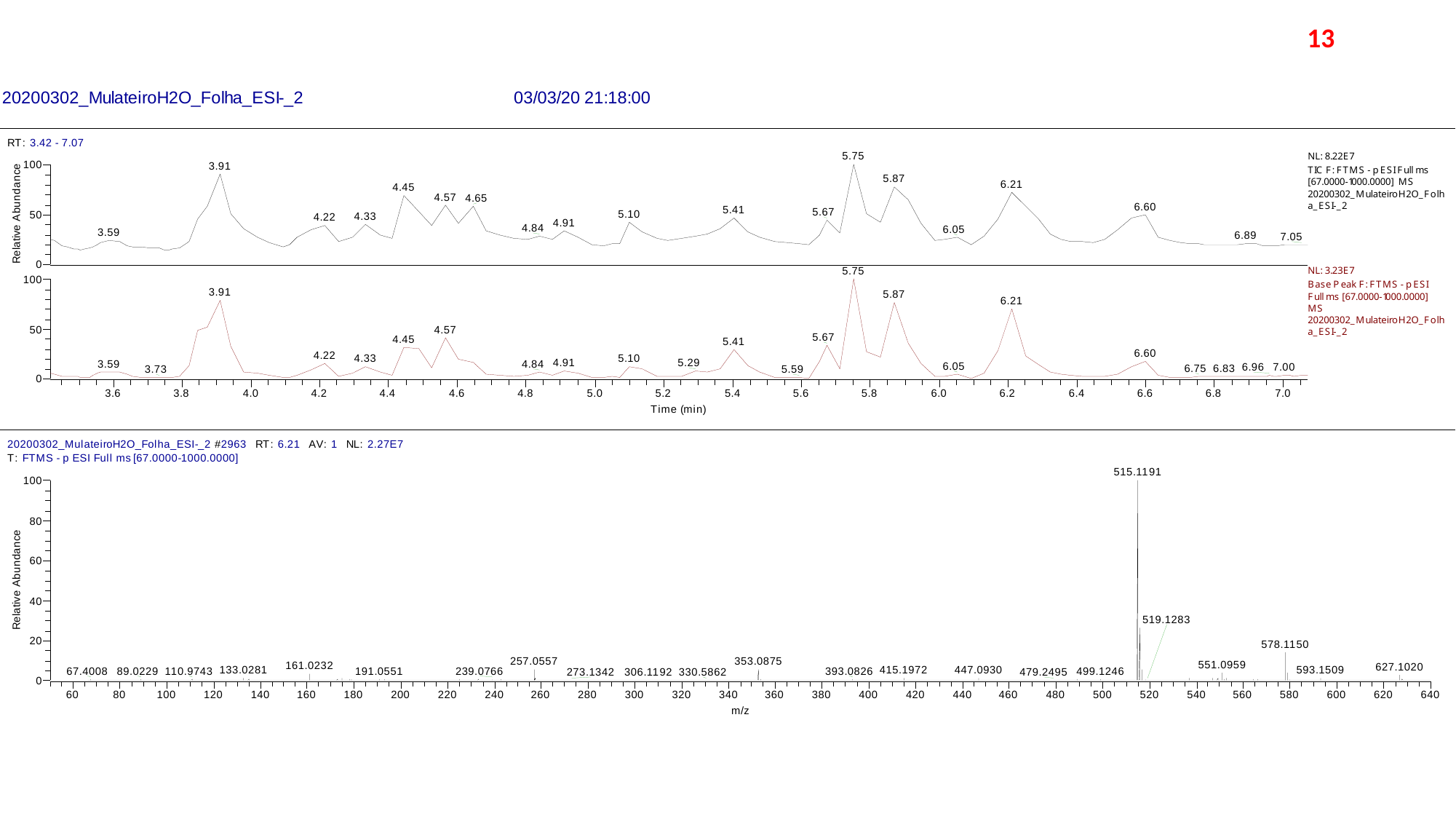

13

## Slide 33
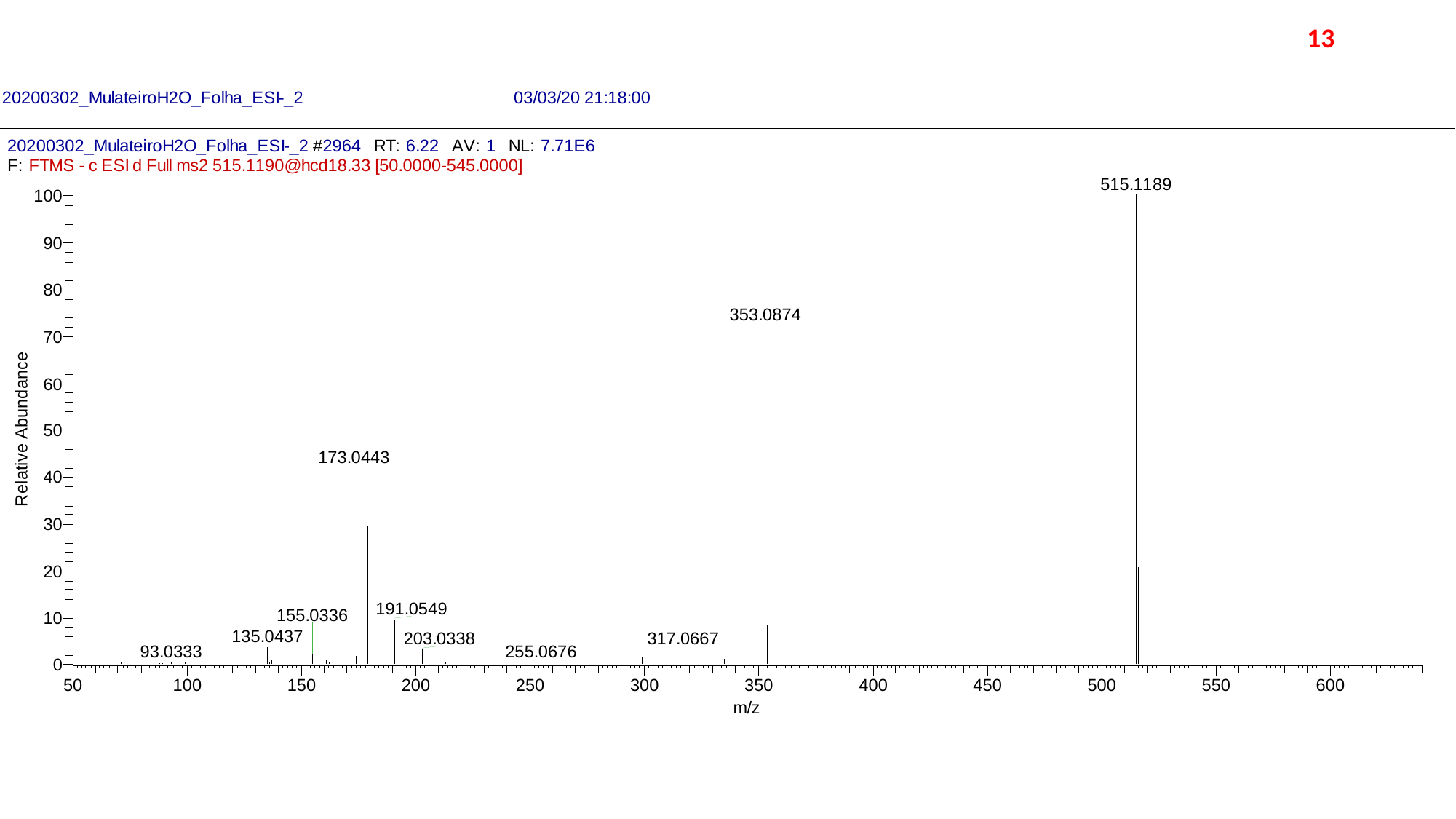

13

## Slide 34
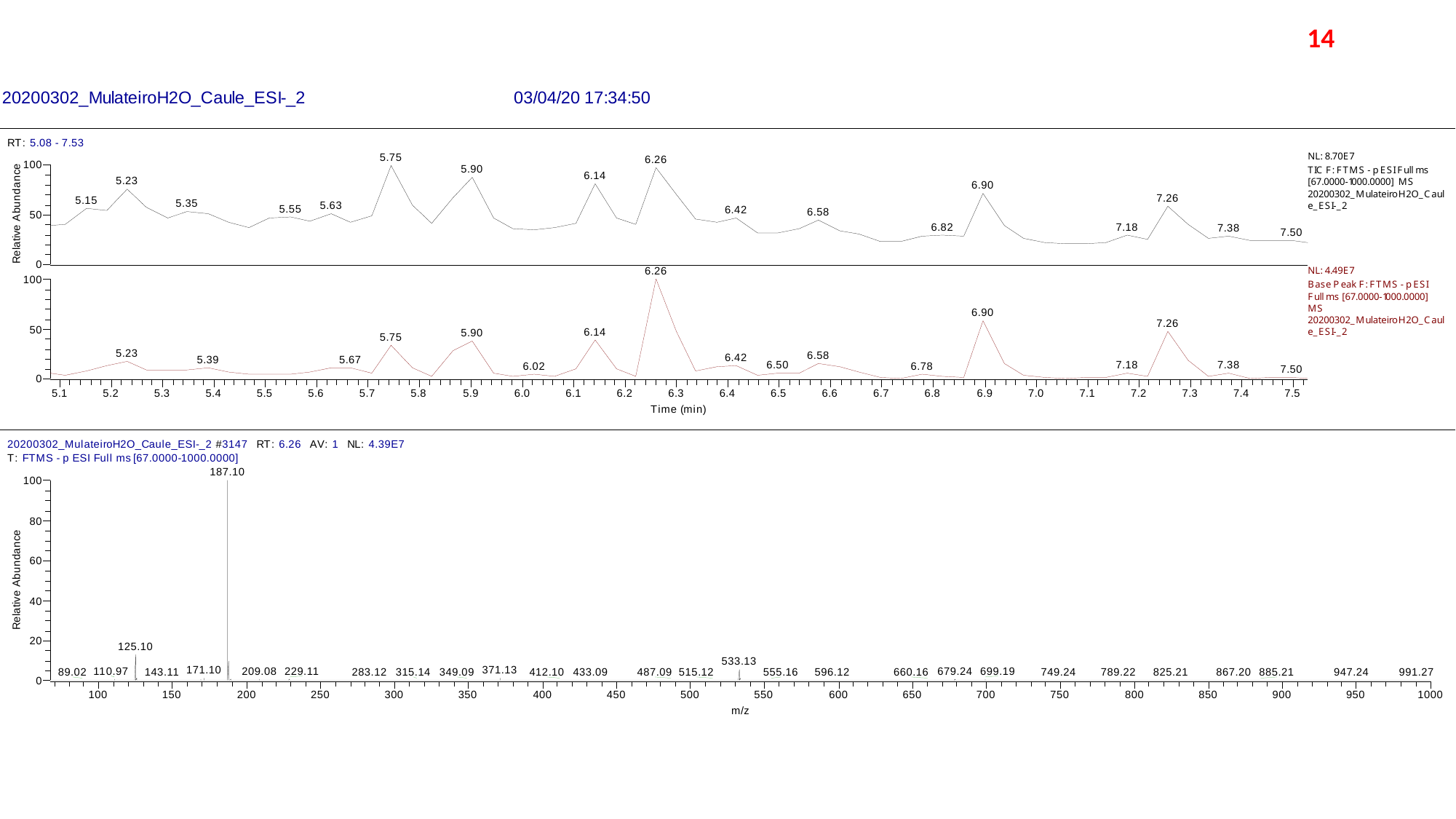

14

## Slide 35
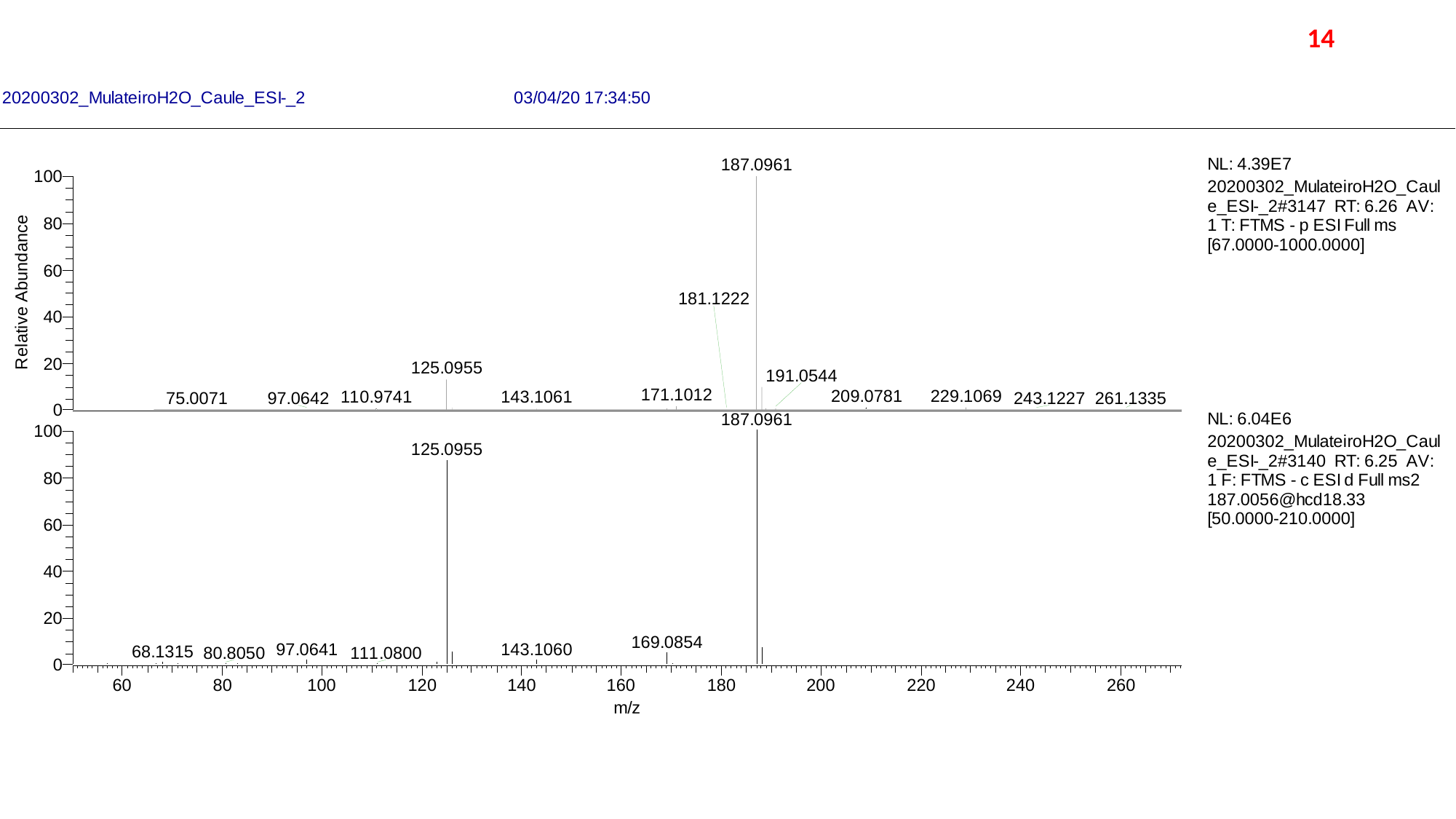

14

## Slide 36
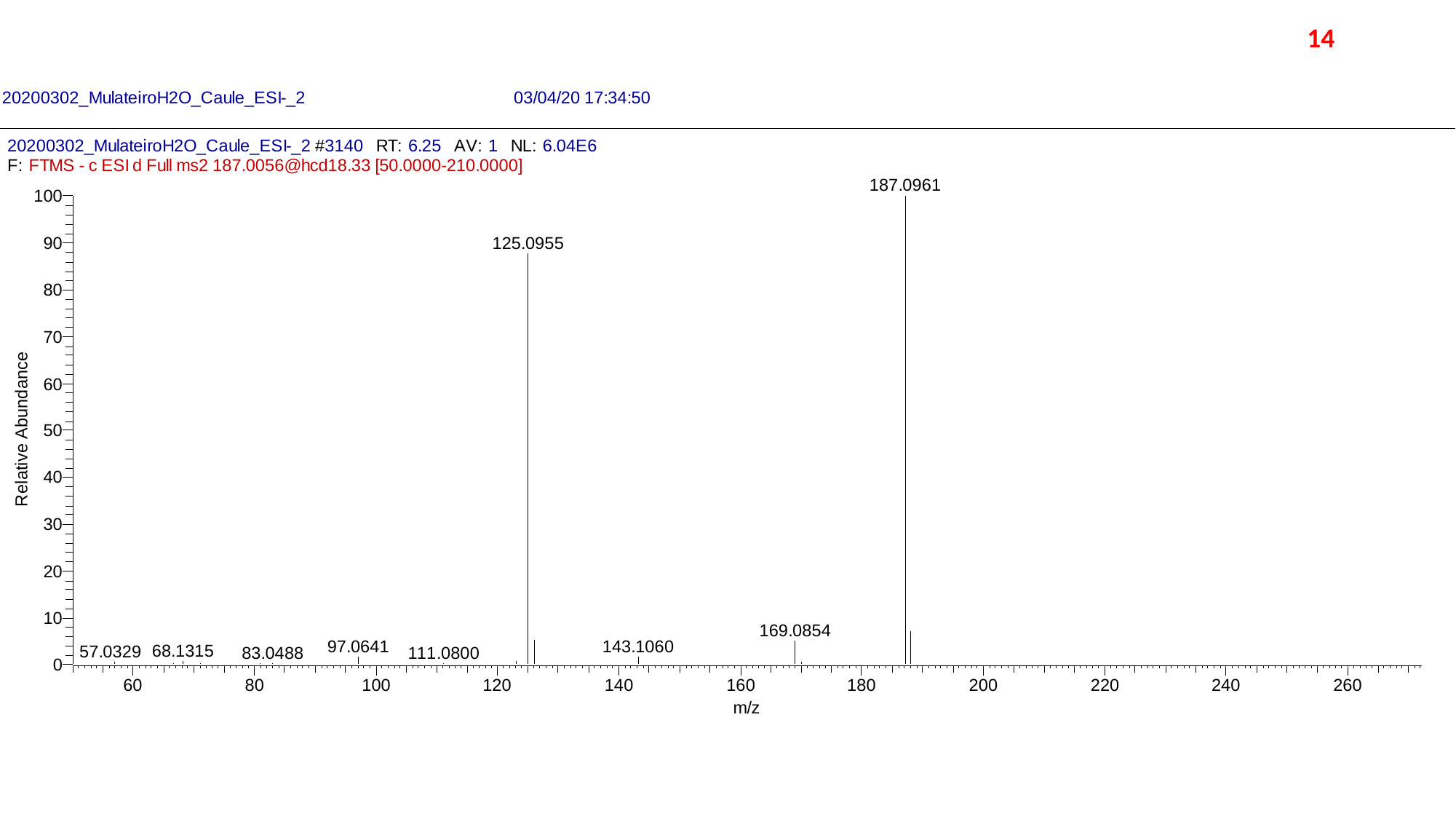

14

## Slide 37
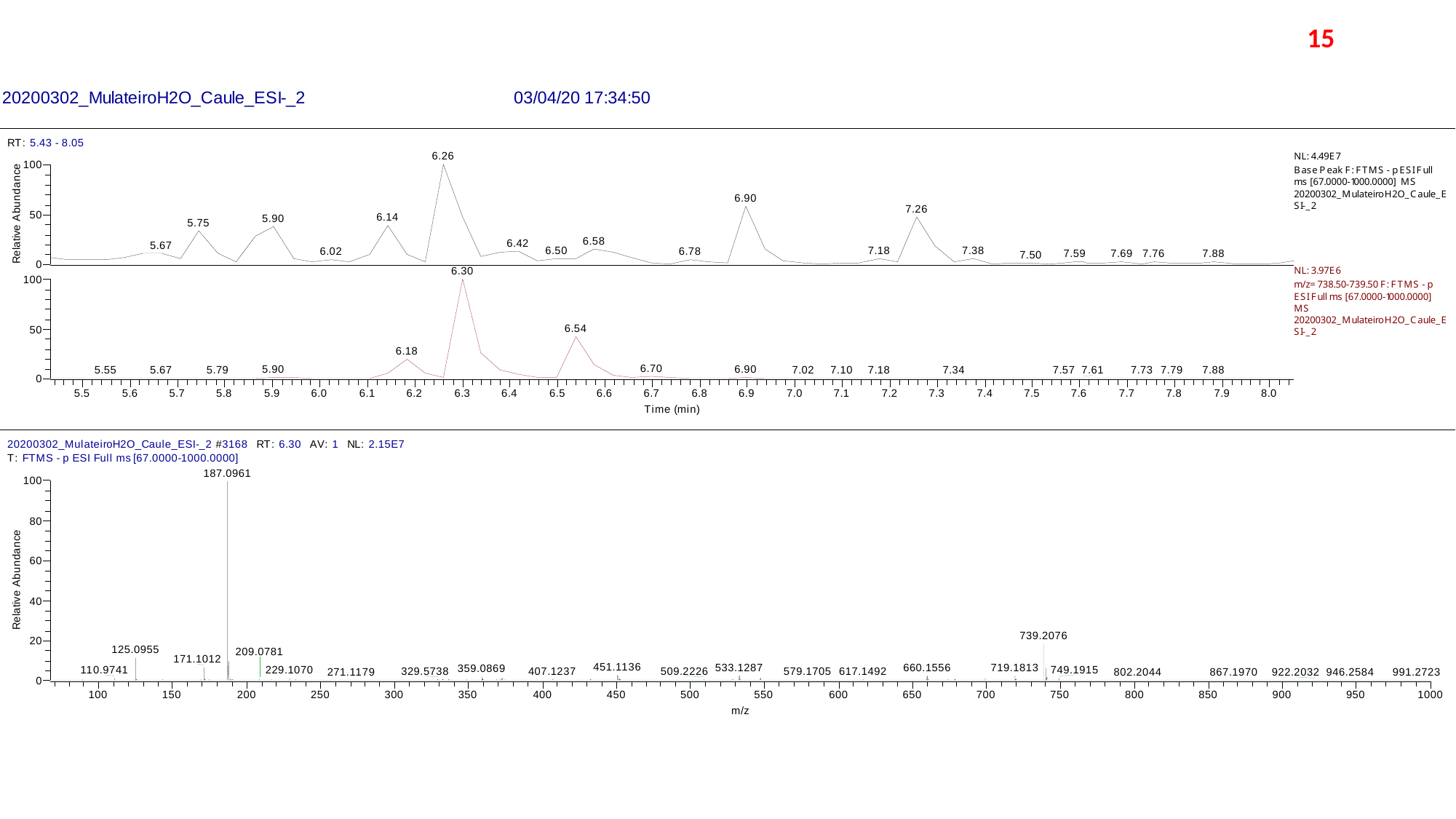

15

## Slide 38
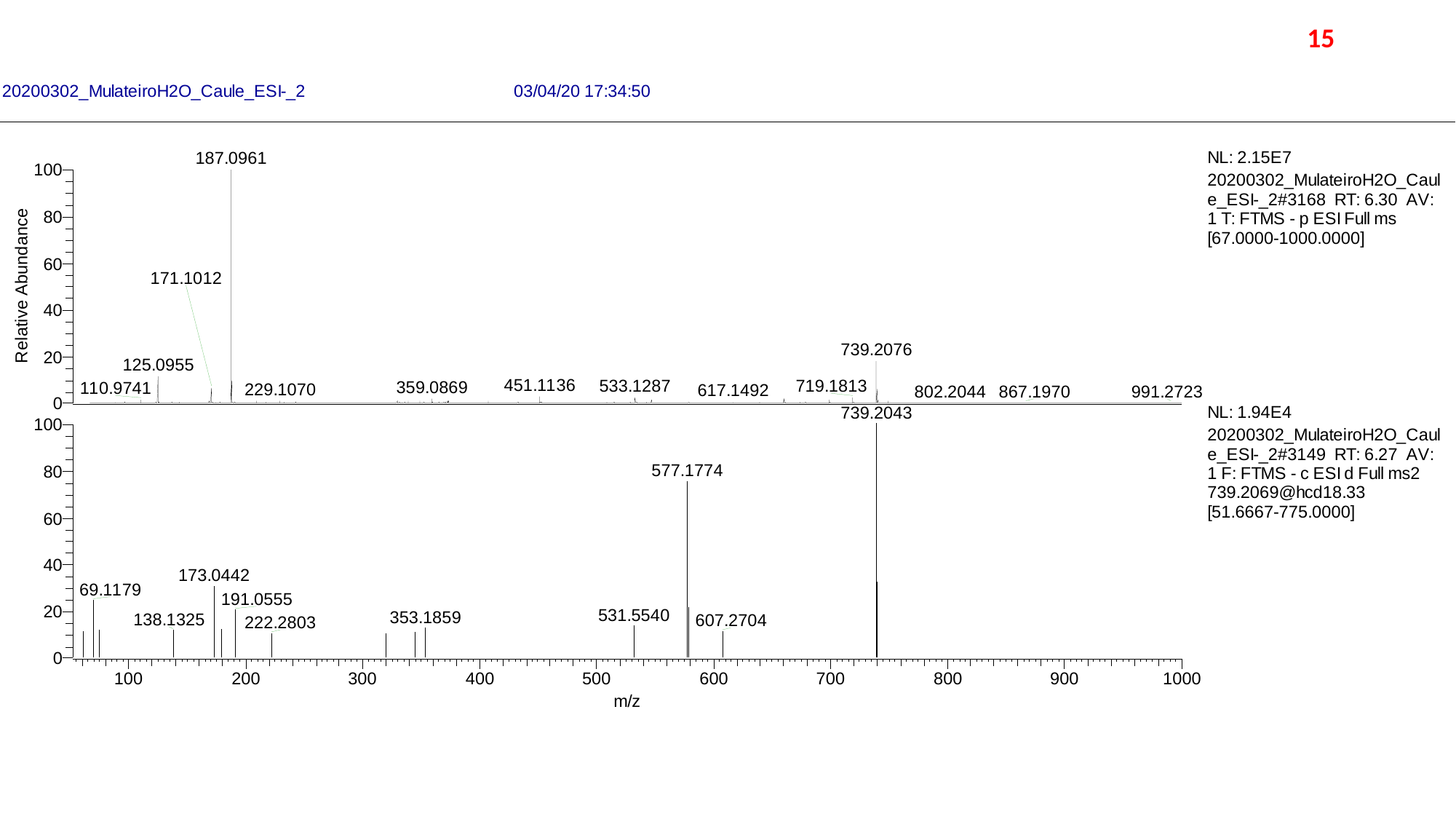

15

## Slide 39
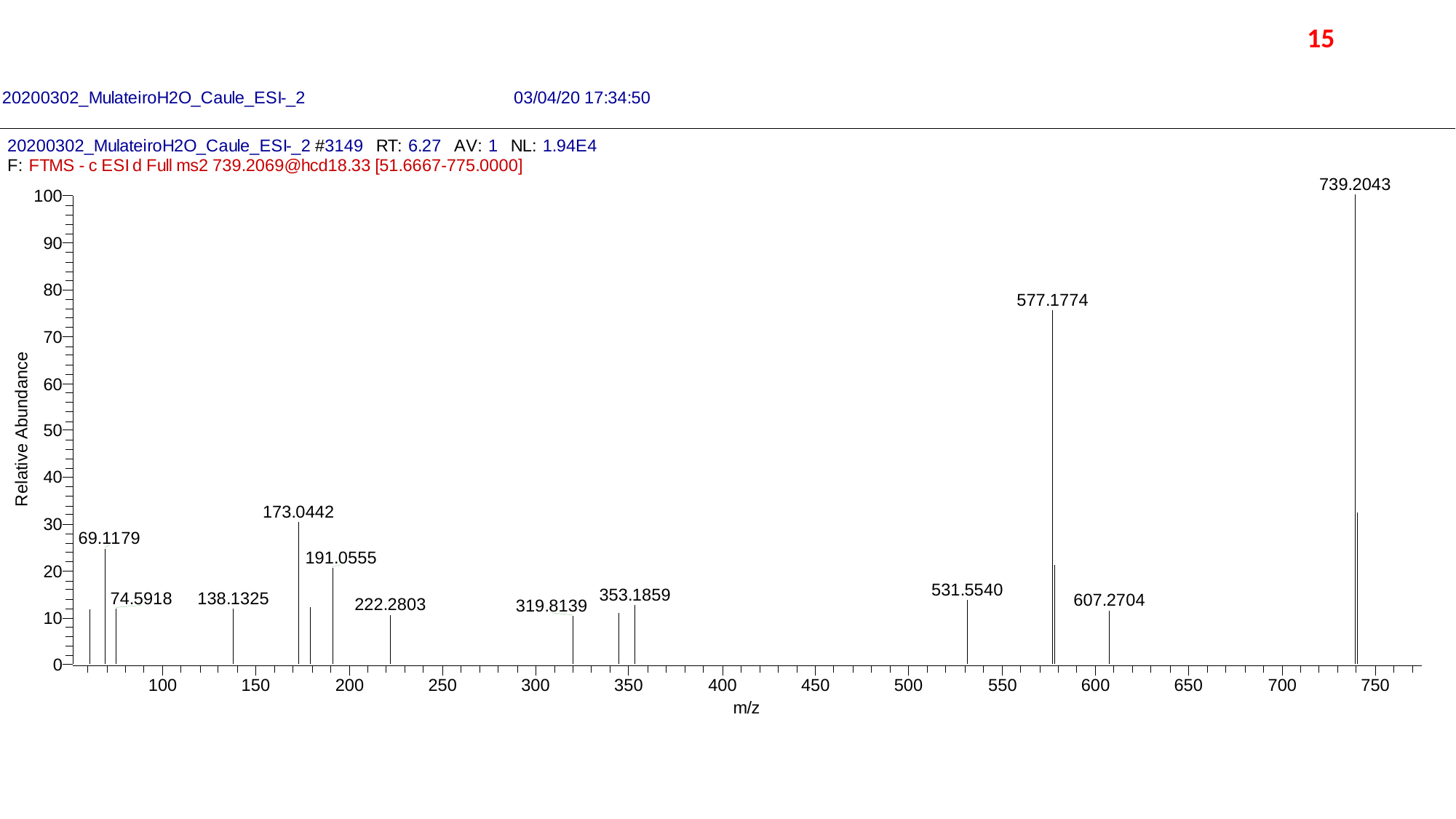

15

## Slide 40
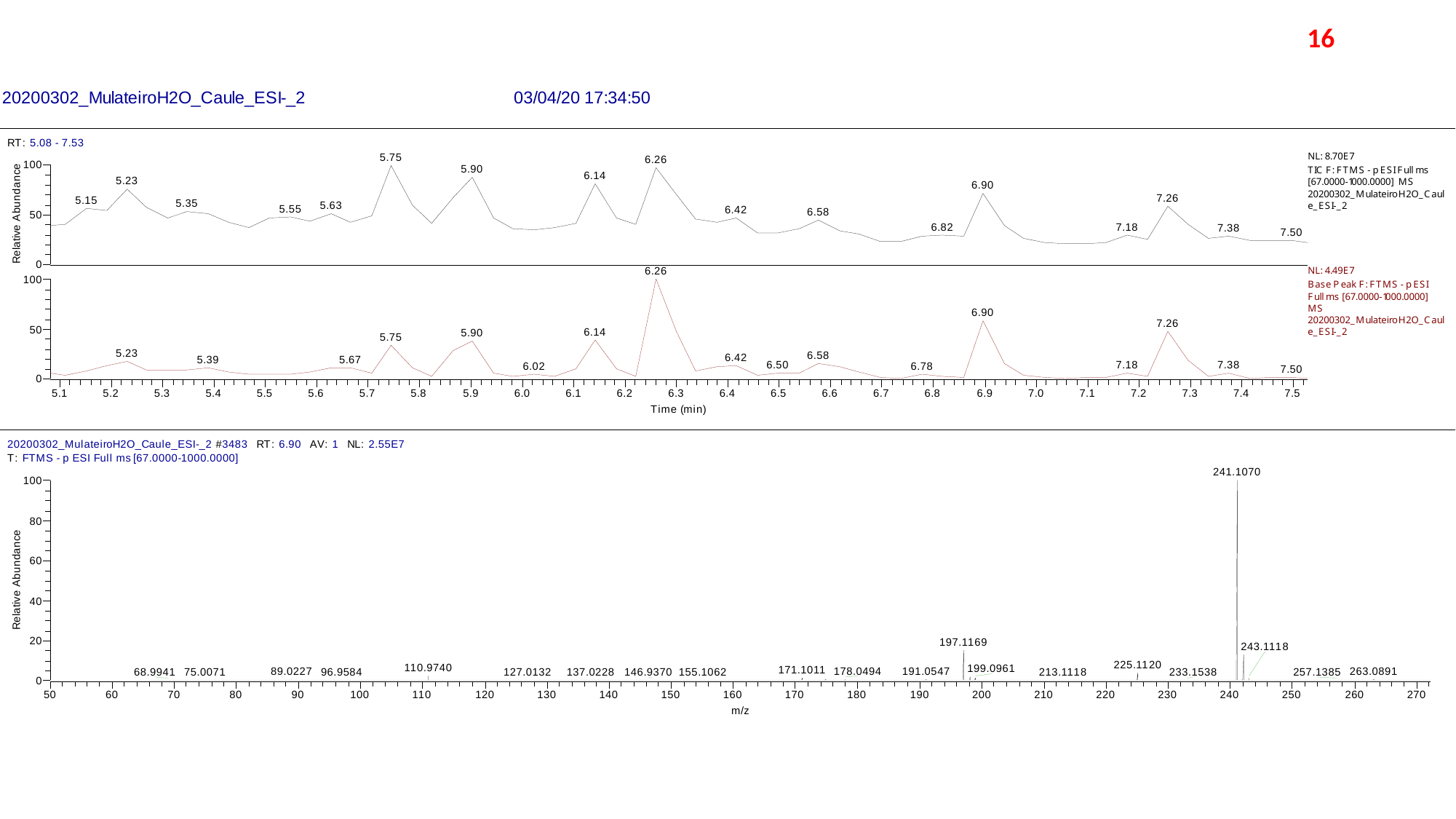

16

## Slide 41
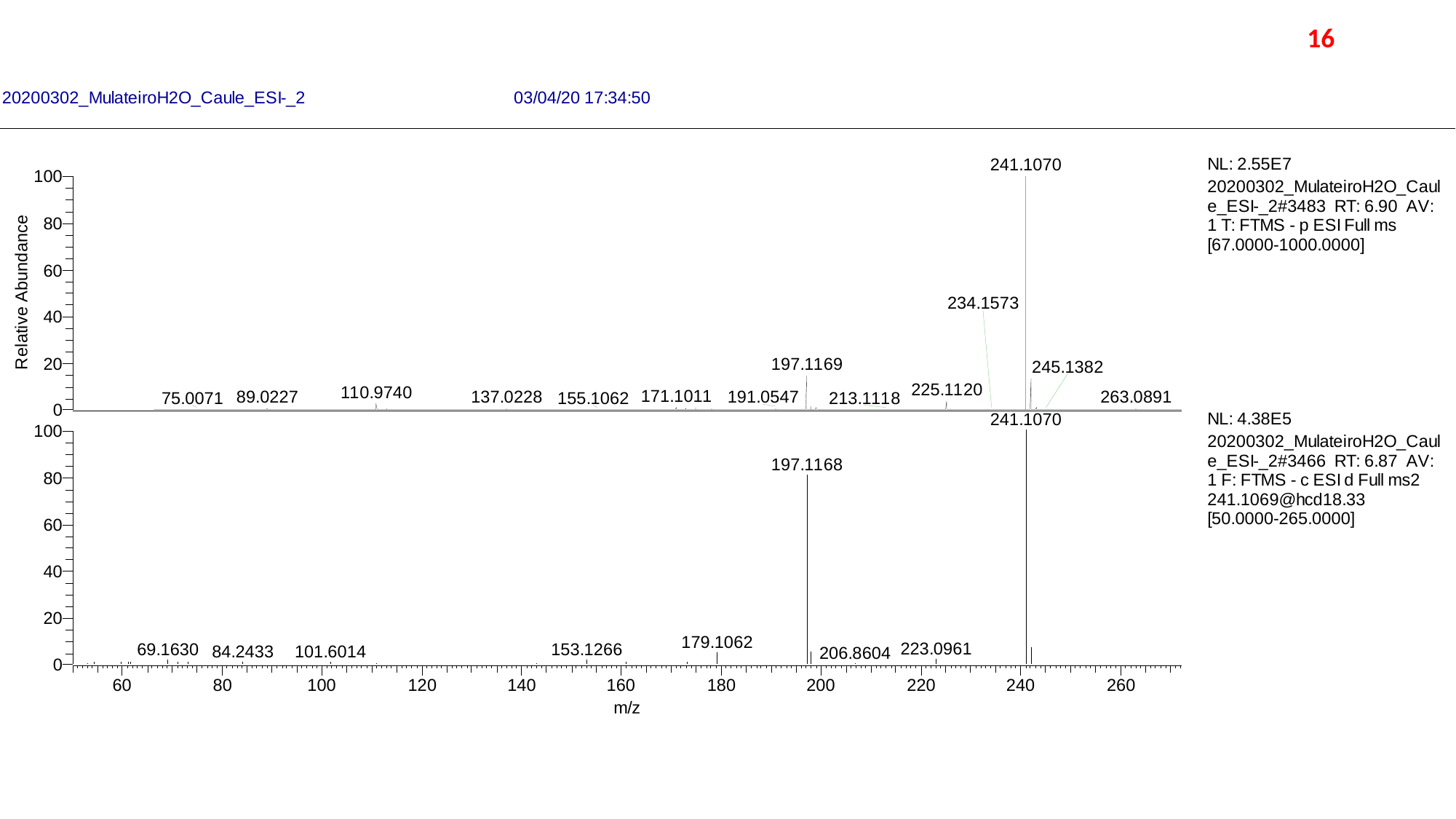

16

## Slide 42
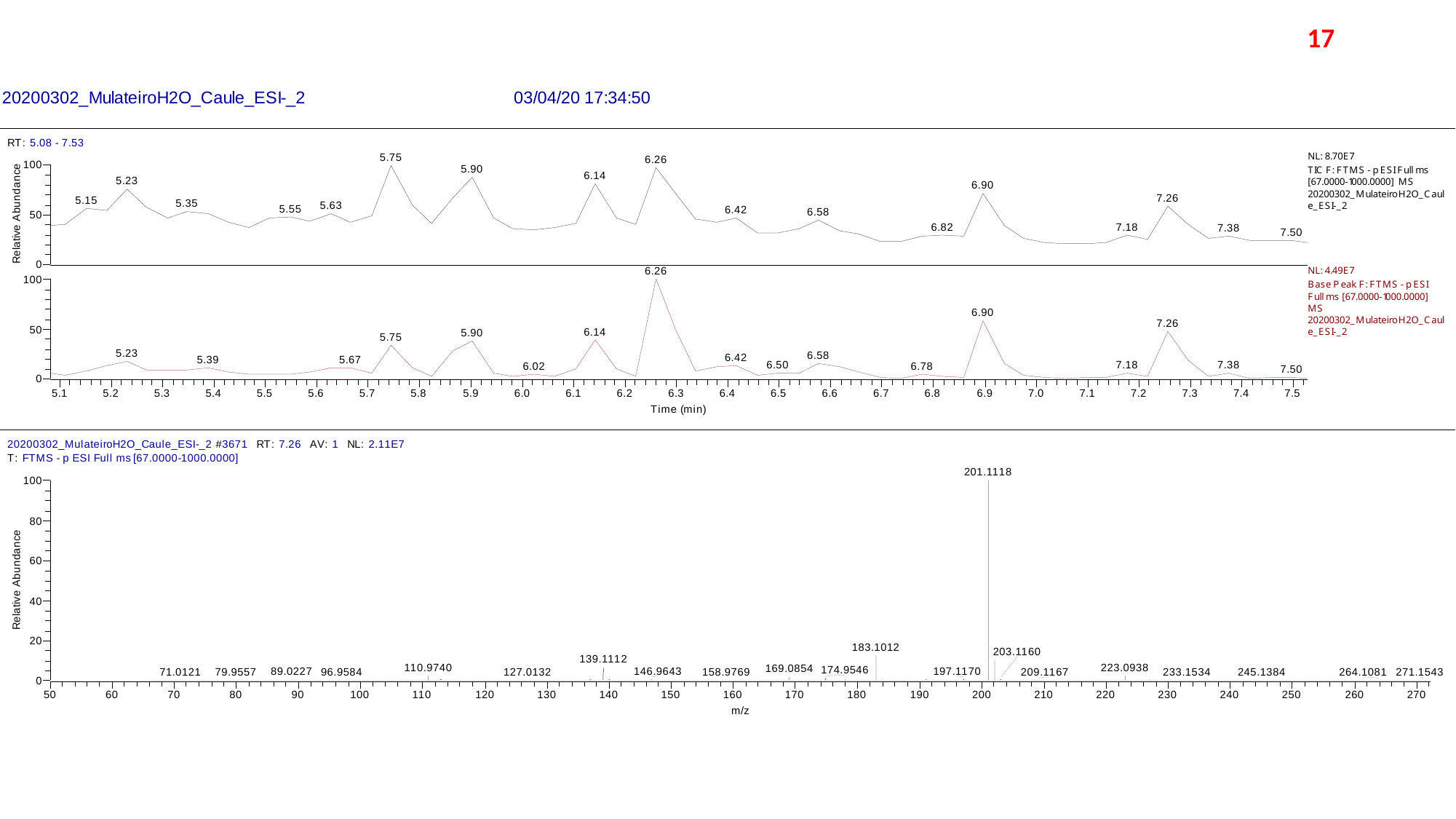

17

## Slide 43
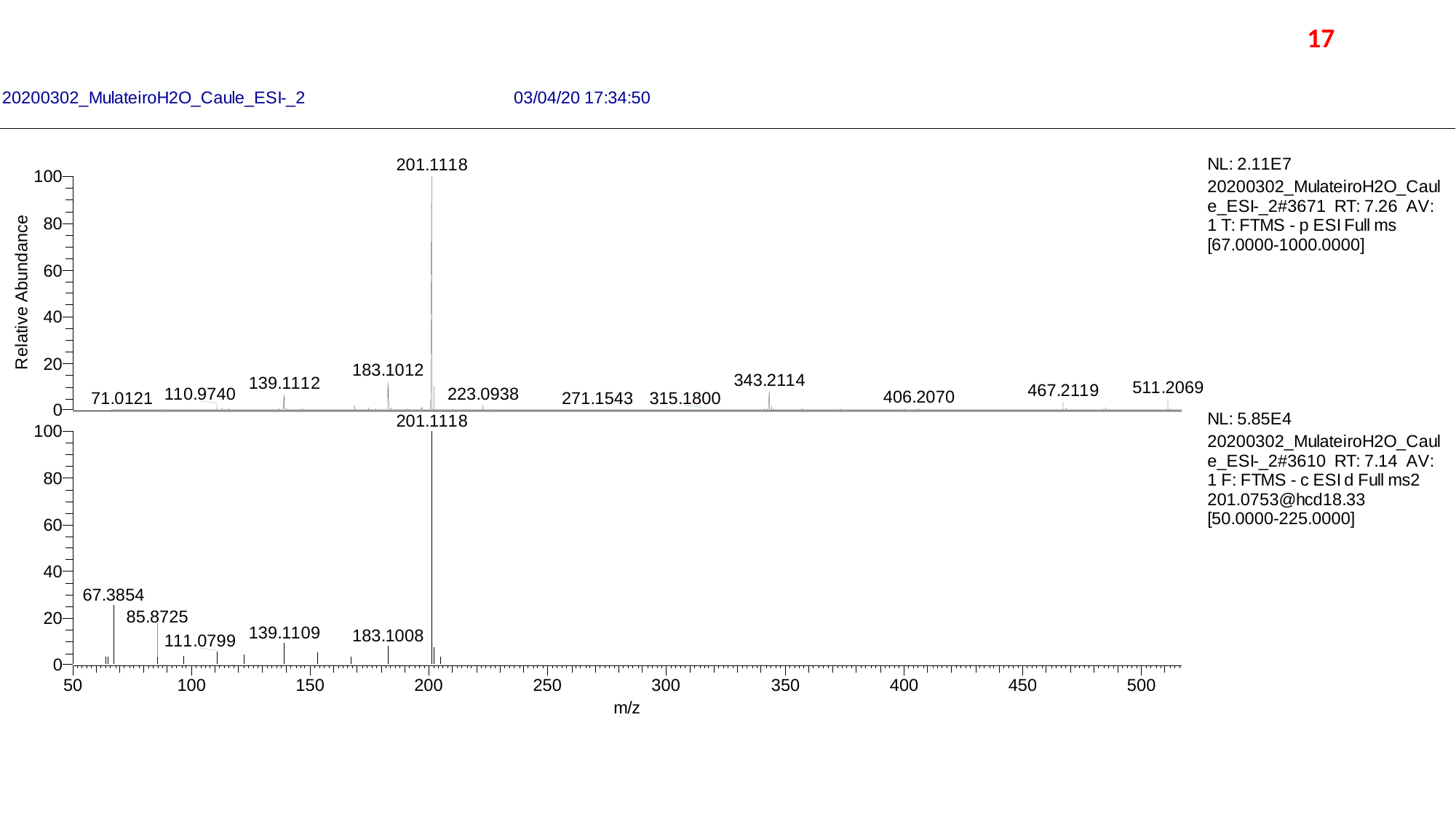

17

## Slide 44
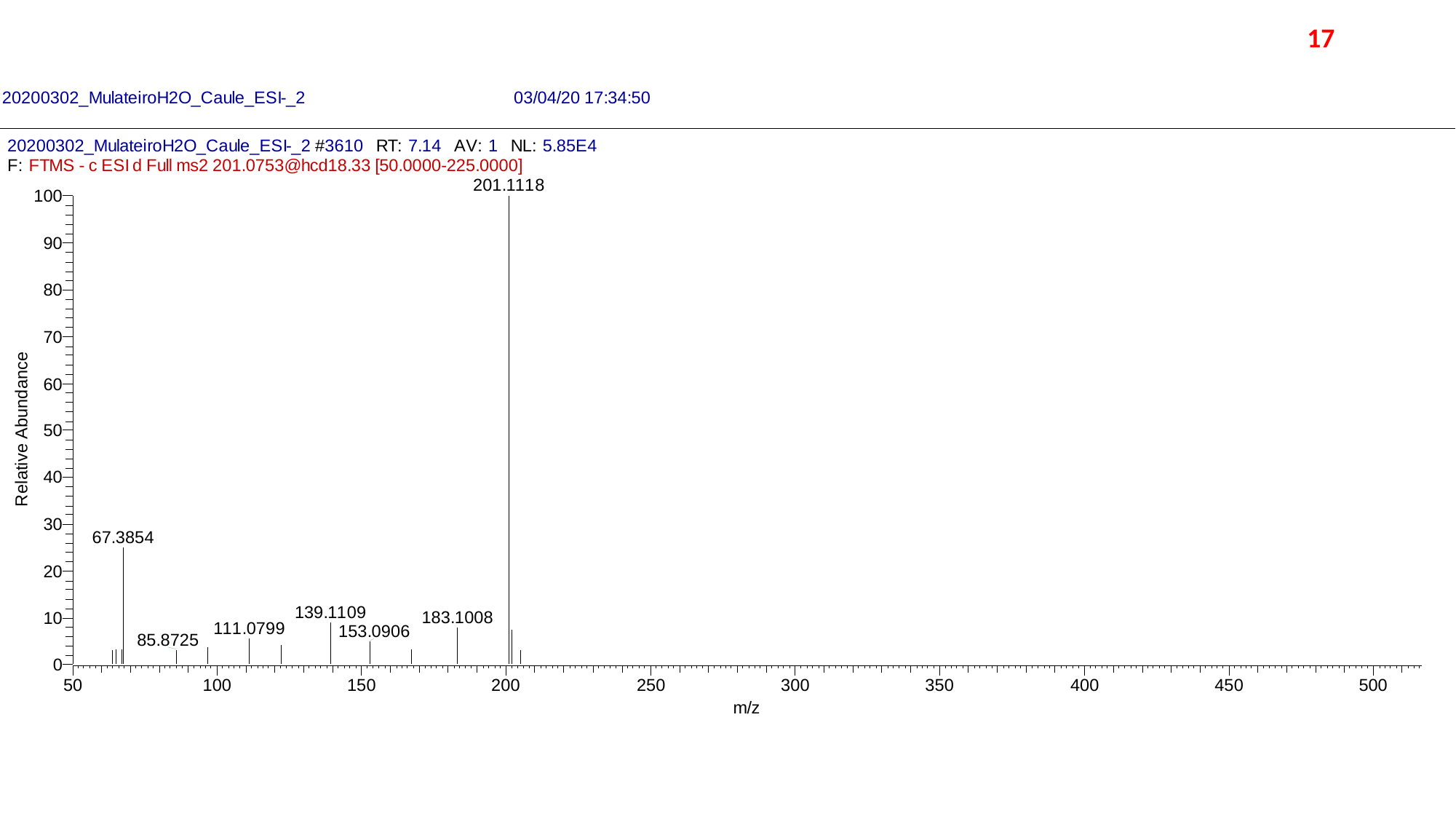

17

## Slide 45
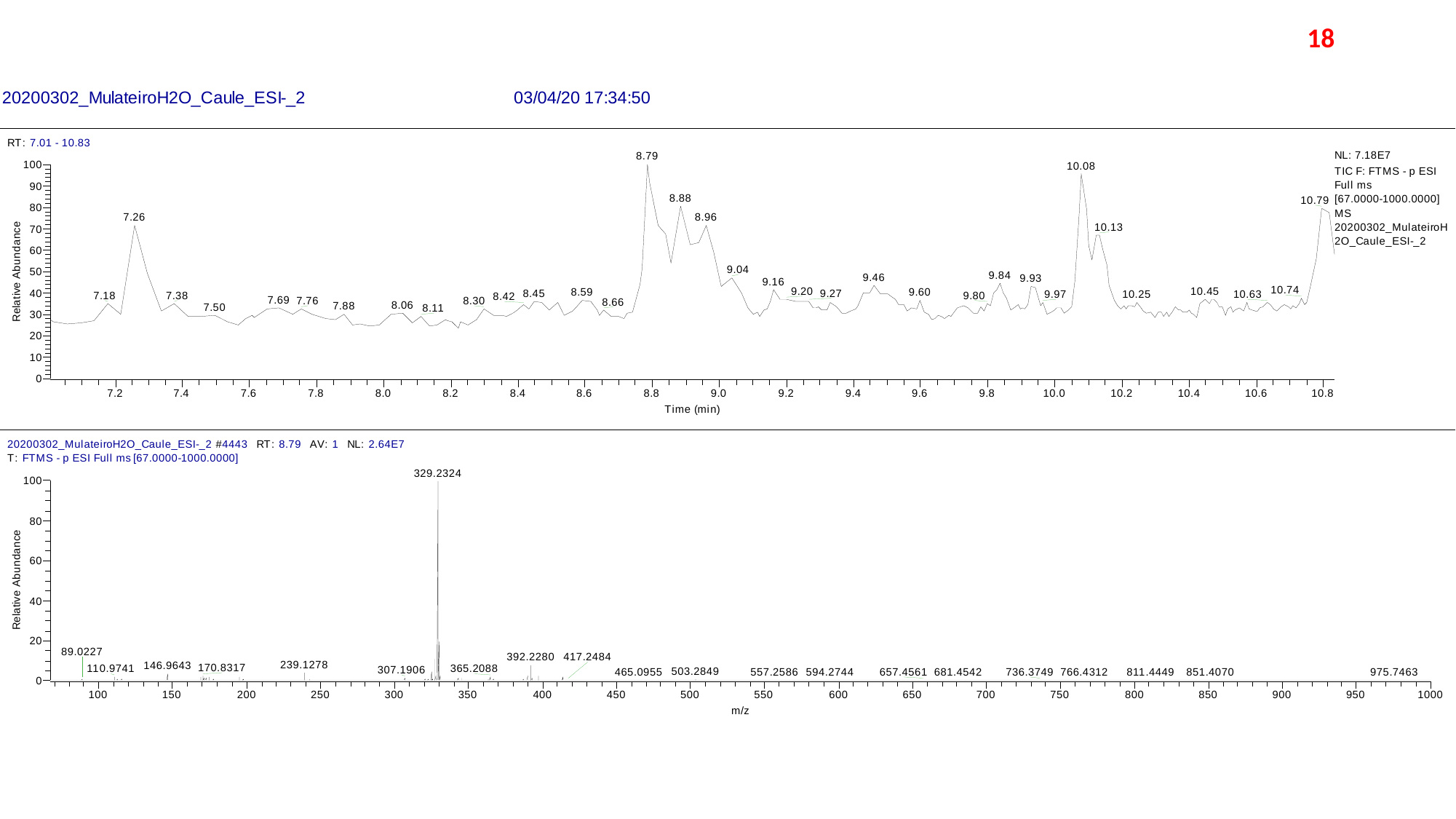

18

## Slide 46
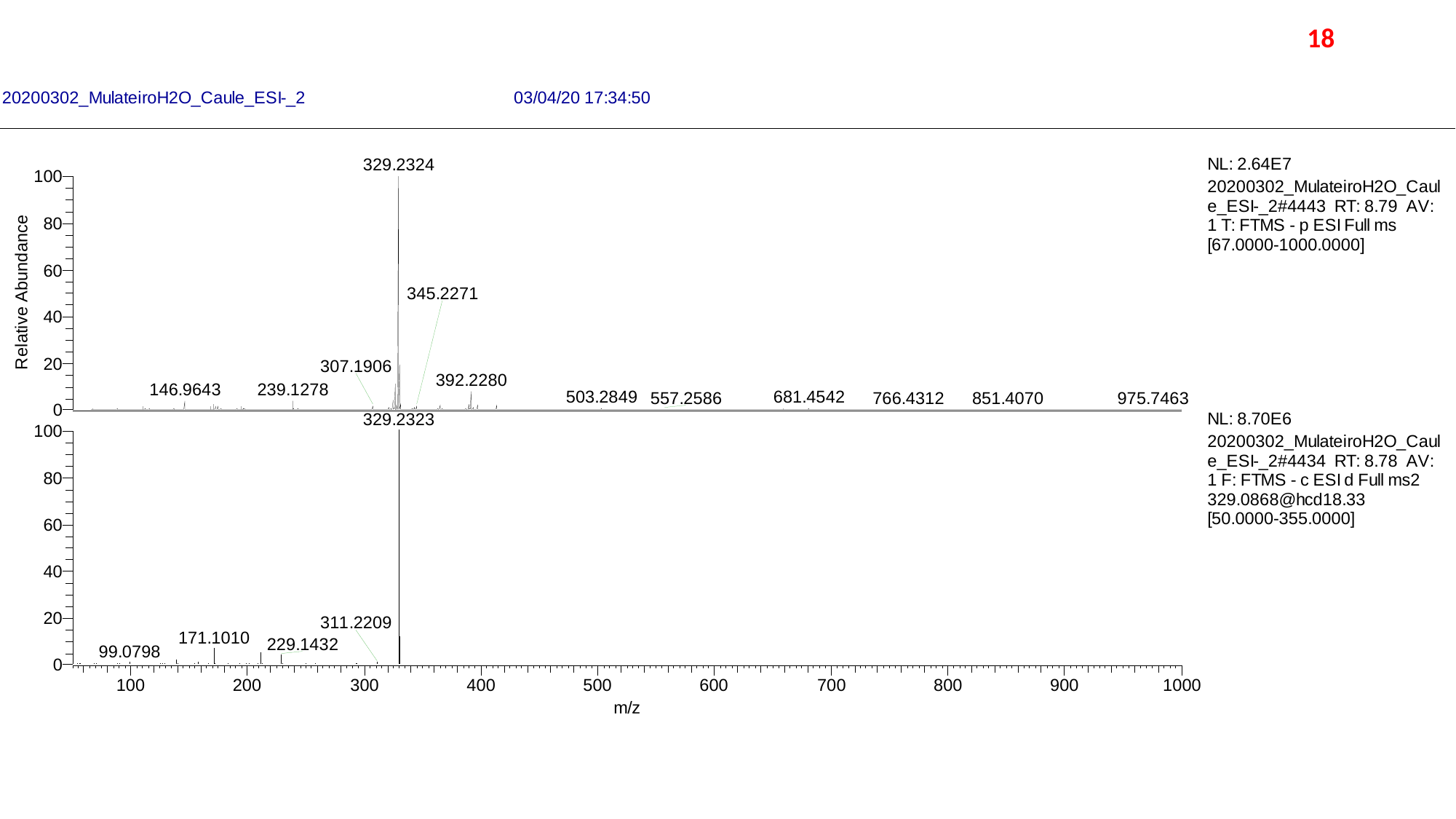

18

## Slide 47
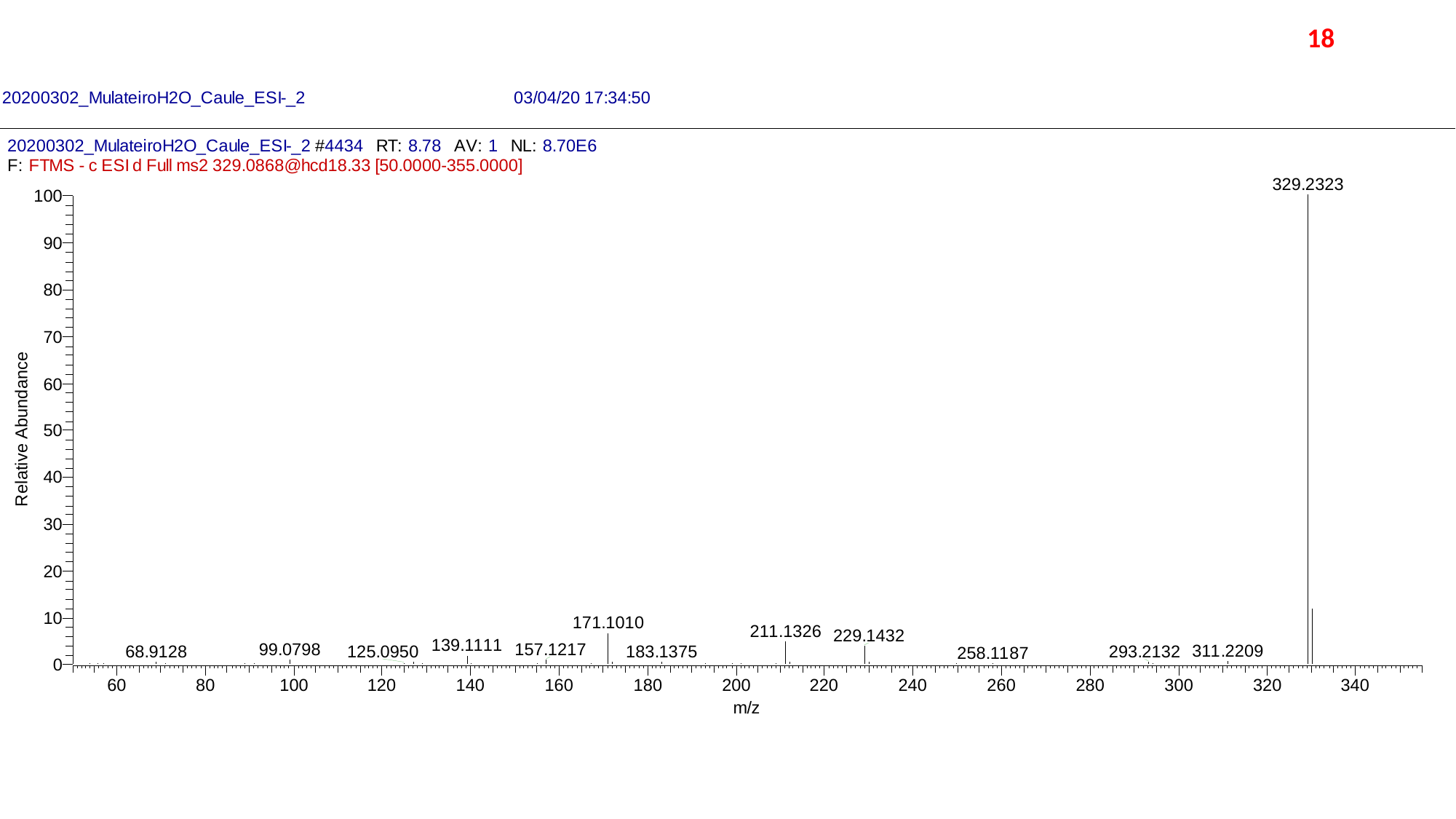

18

## Slide 48
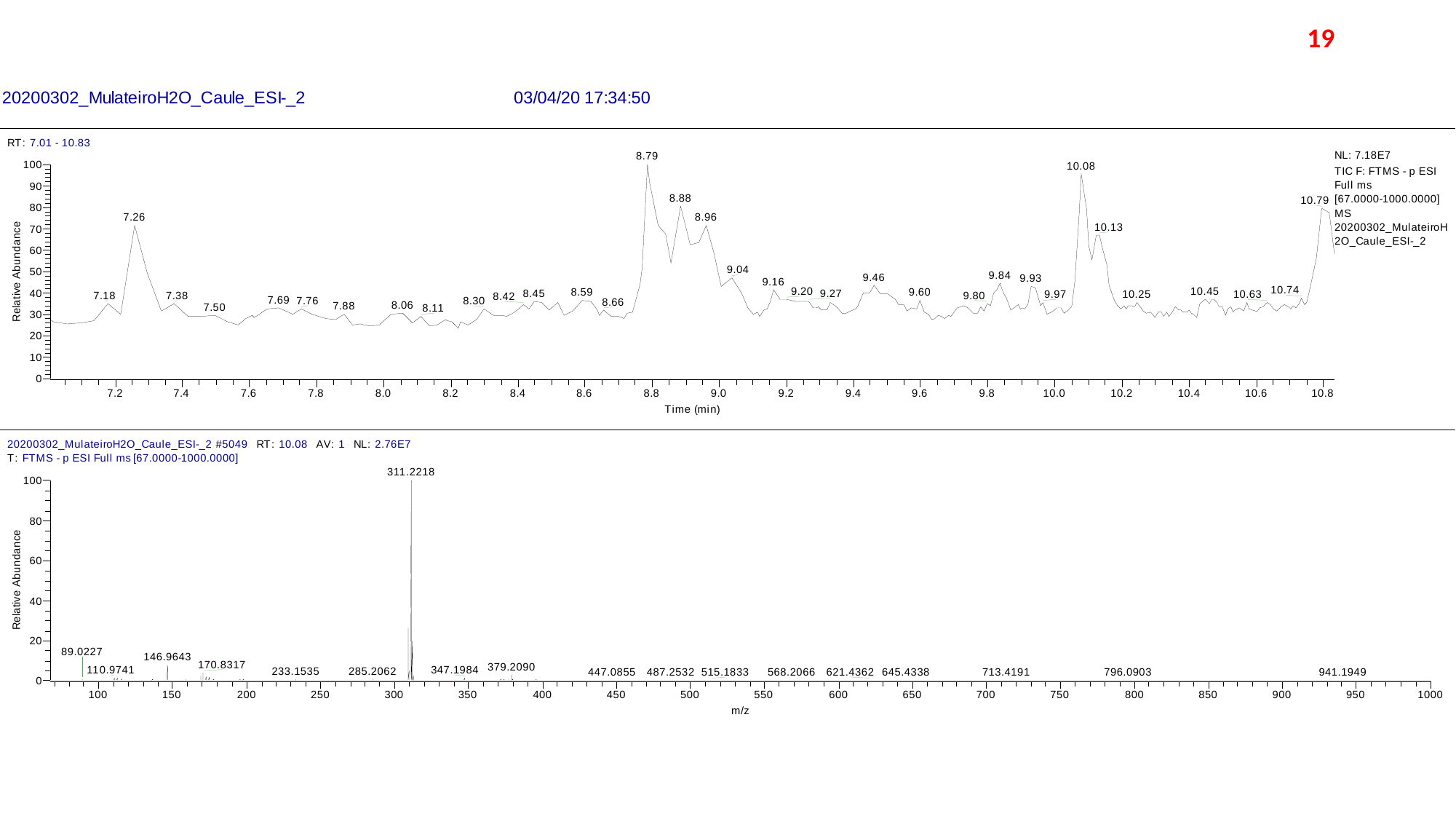

19

## Slide 49
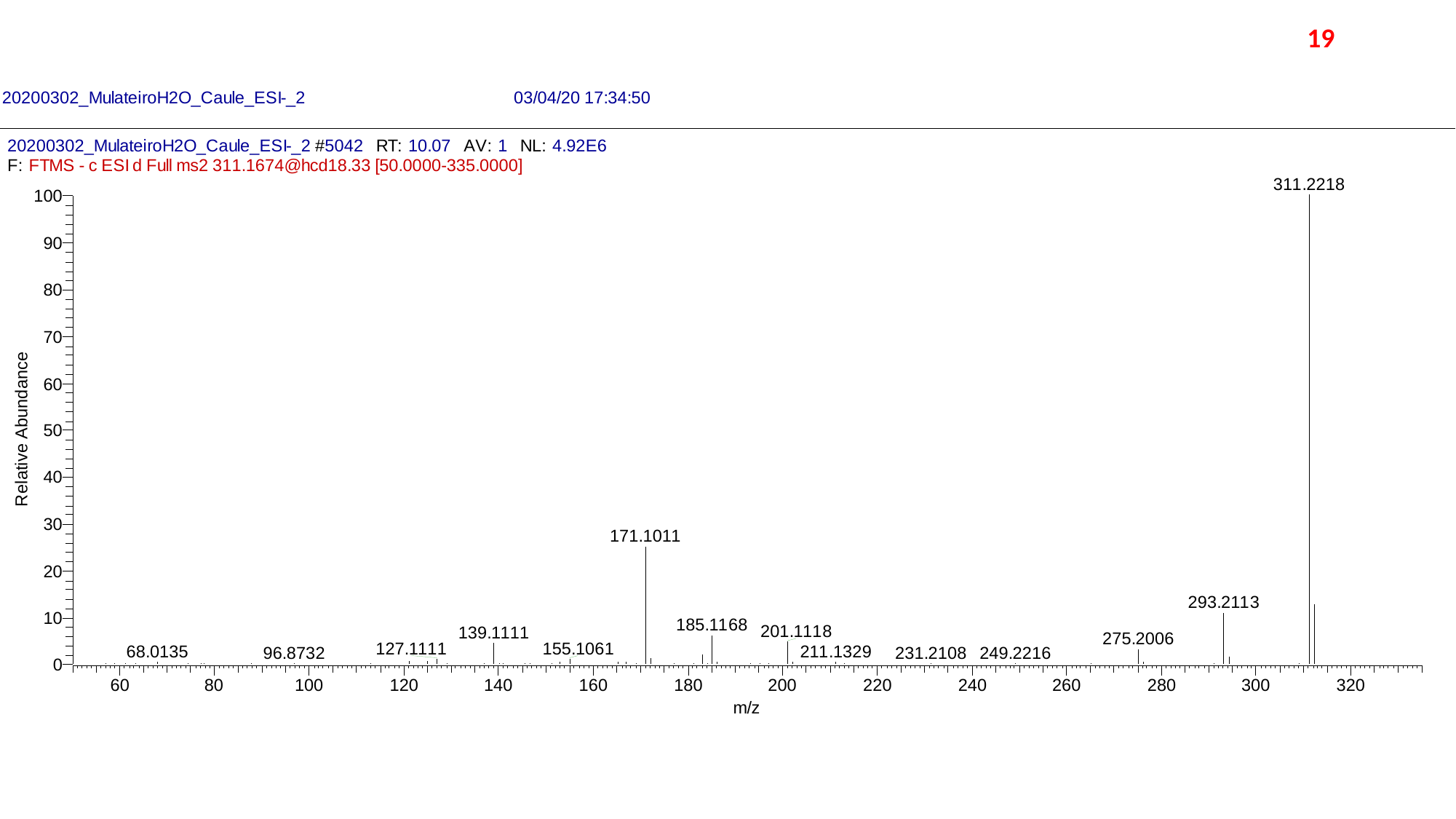

19

## Slide 50
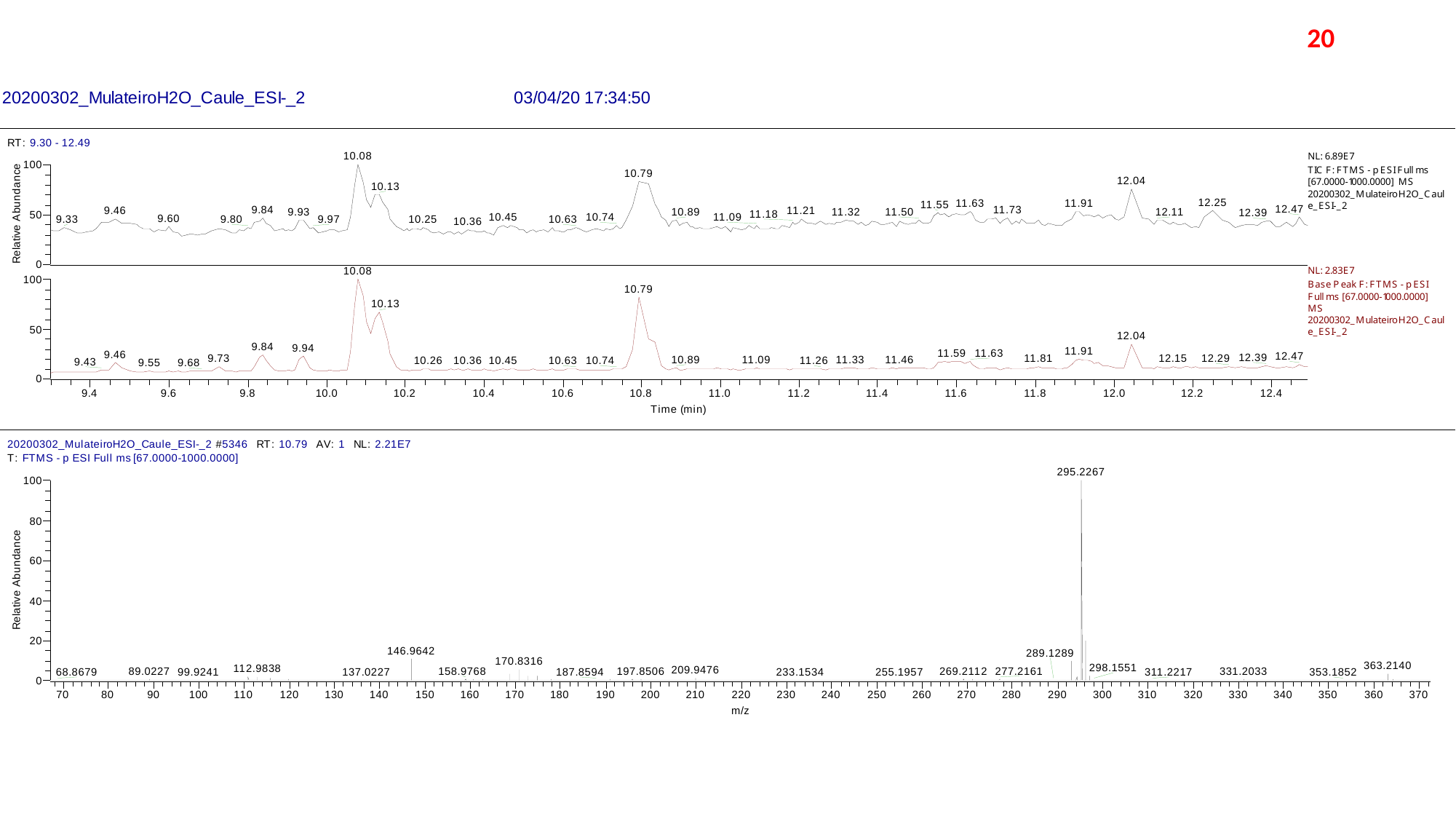

20

## Slide 51
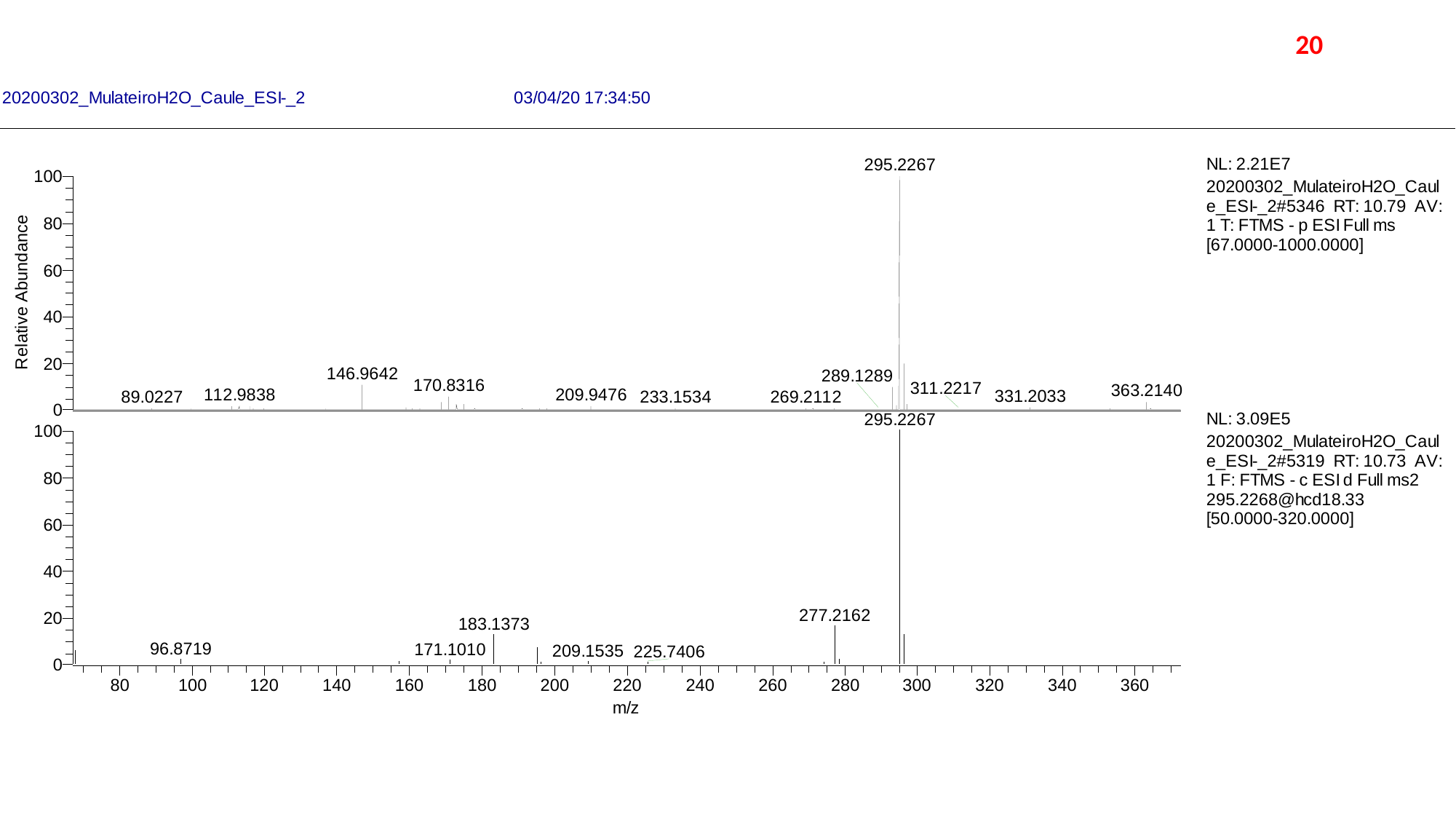

20

## Slide 52
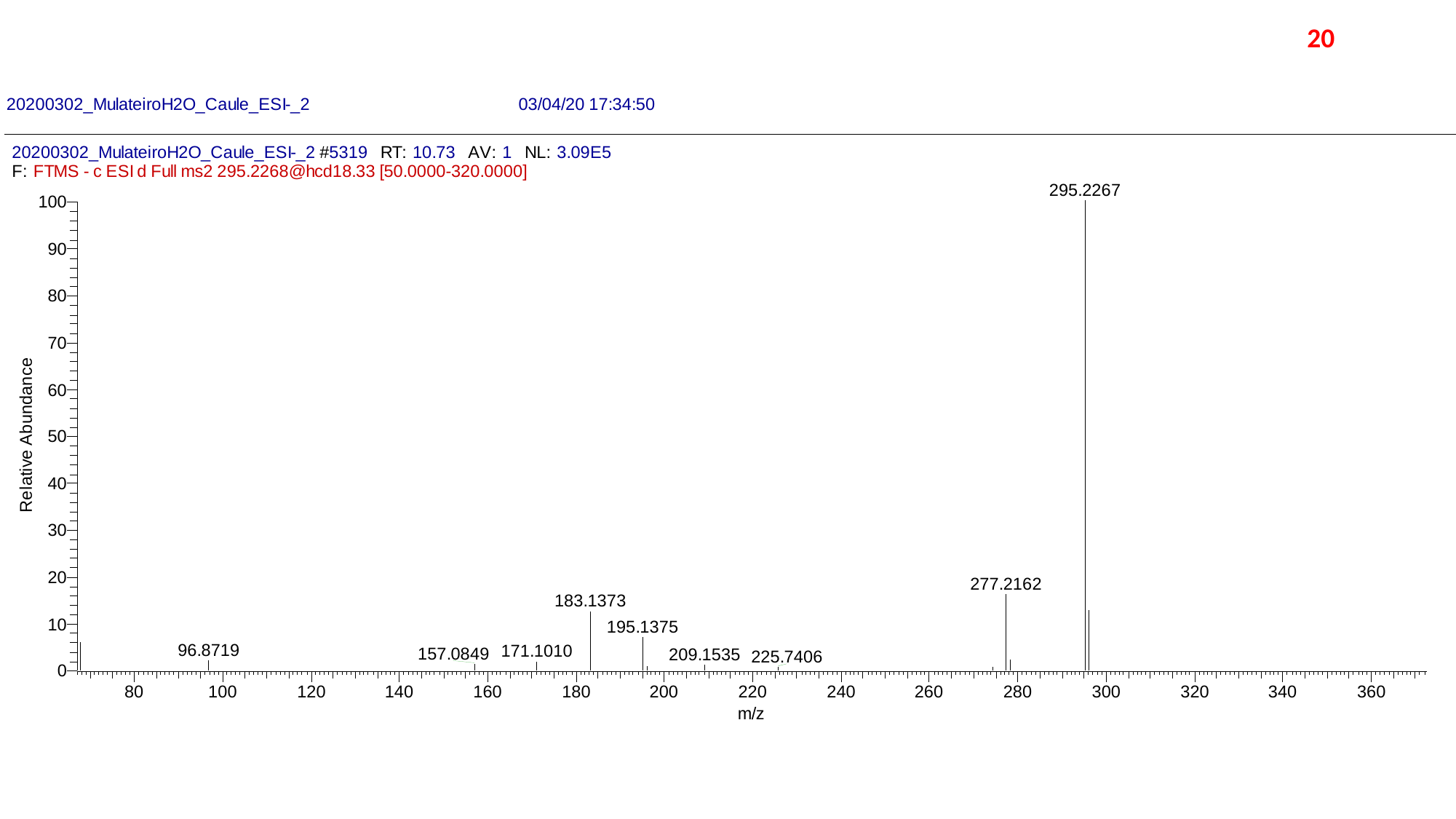

20
