# Supplementary figures and images for: Identification of Bioactive Metabolites of Capirona macrophylla by Metabolomic Analysis, Molecular Docking, and In Vitro Antiparasitic Assays
Source: Metabolites. 2025 Feb 26;15(3):157. doi: 10.3390/metabo15030157 (PMC11943490; doi:10.3390/metabo15030157)

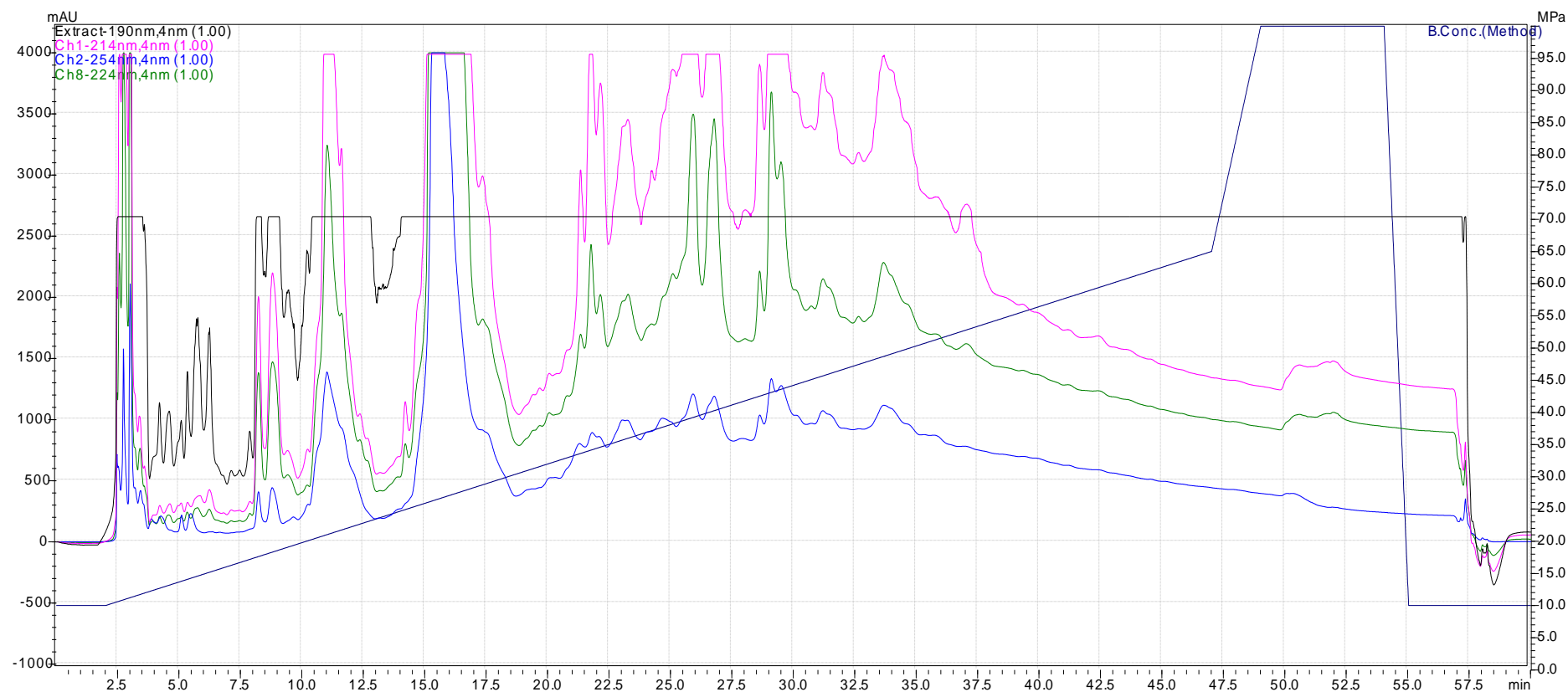

Supplement: Supplementary file 1 [file metabolites-15-00157-s001.zip › Supplementary material S5.pdf]

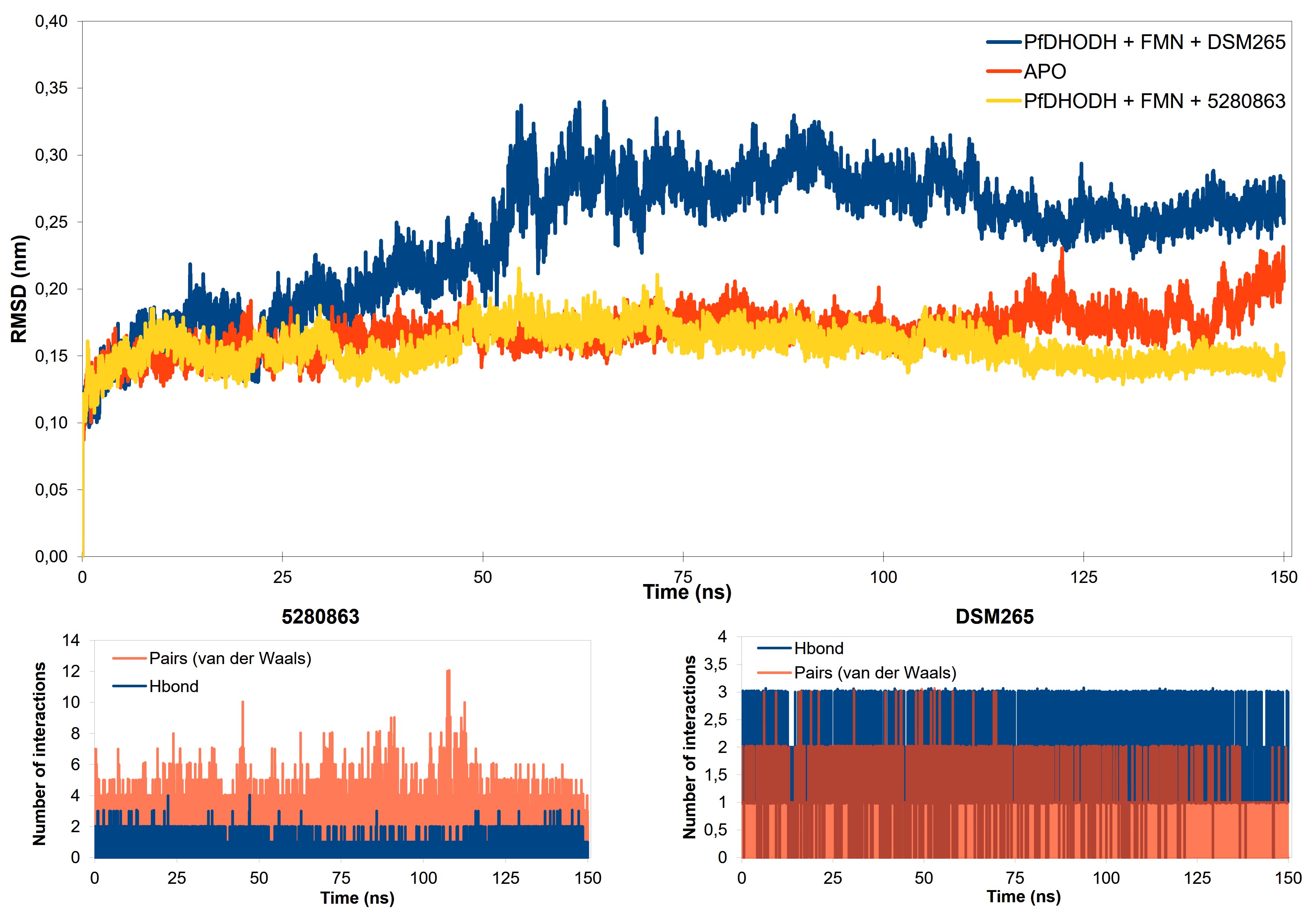

Supplement: Supplementary file 1 [file metabolites-15-00157-s001.zip › Supplementary material S6.jpg]

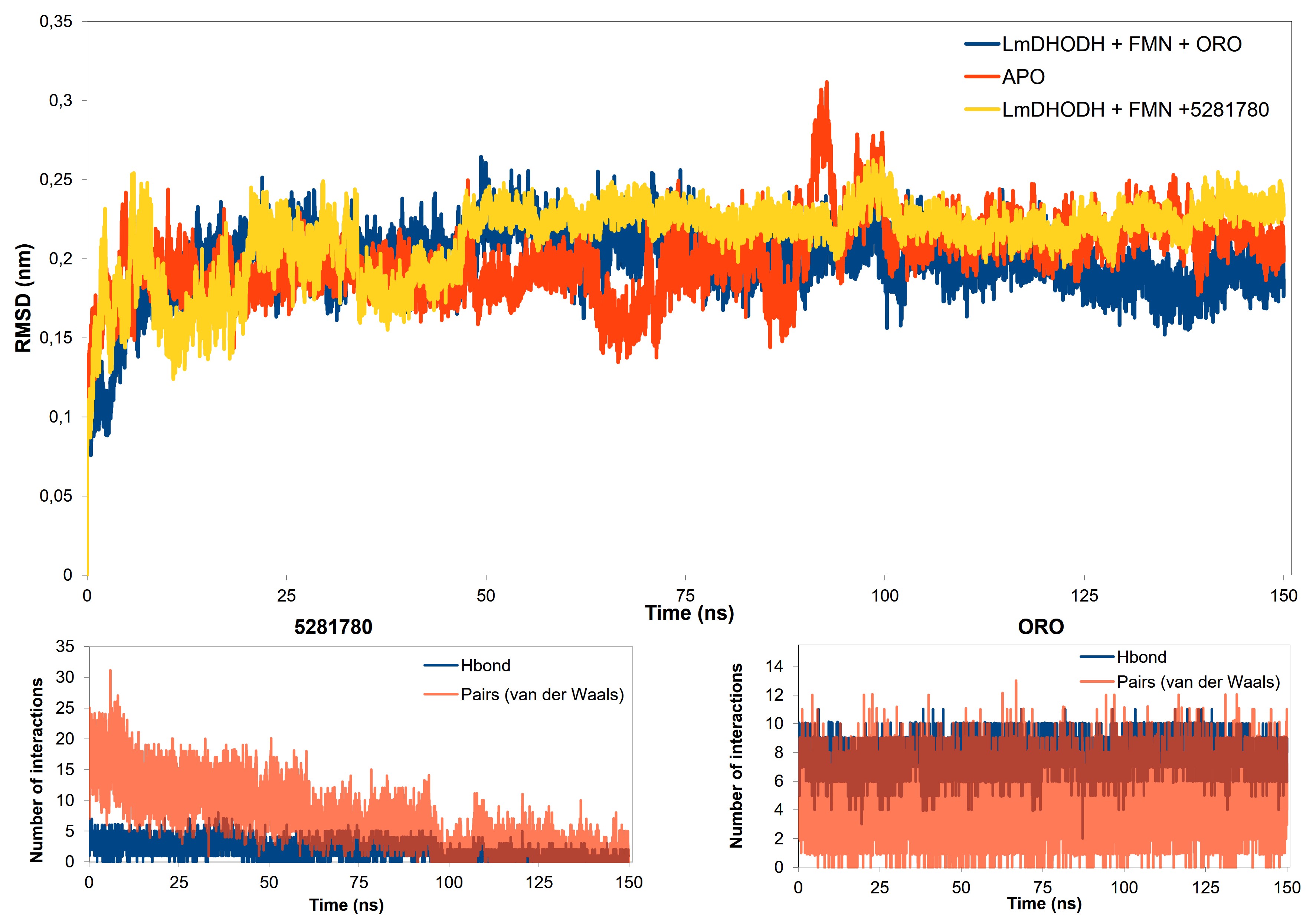

Supplement: Supplementary file 1 [file metabolites-15-00157-s001.zip › Supplementary material S7.jpg]
